# Beyond Histone Demethylation: Mechanisms of *N*‑Alkyl Consecutive Oxidations by the Non-Heme Fe(II)/2-Oxoglutarate Oxygenase KDM6B

**DOI:** 10.1021/jacsau.6c00575

**Published:** 2026-06-06

**Authors:** Simahudeen Bathir Jaber Sathik Rifayee, Sudheesh Devadas, Midhun George Thomas, Bhargav Varada, Ethan Sommer, Cassandra Talaba, Christopher Schofield, Christo Z. Christov

**Affiliations:** † Department of Chemistry, 3968 Michigan Technological University, Houghton, Michigan 49931, United States; ‡ Department of Biomedical Engineering, Michigan Technological University, Houghton, Michigan 49931, United States; § Department of Biochemistry and Molecular Biology, Michigan Technological University, Houghton, Michigan 49931, United States; ∥ Chemistry Research Laboratory, Department of Chemistry, and the Ineos Oxford Institute for Antimicrobial Research, 6396 University of Oxford, Oxford OX1 3TA, U.K.

**Keywords:** KDM6B, methyl, ethyl, isopropyl, MD, QM/MM

## Abstract

The nonheme Fe­(II)/2-oxoglutarate (2OG)-dependent histone demethylase KDM6B (JMJD3) has demonstrated a capacity for diversity in the oxidative transformations of N^ε^-alkylated lysine residues in histone H3 peptides; however, the mechanisms of such dealkylations, compared with standard KDM-catalyzed demethylations, remain unexplored. We implemented molecular dynamics and quantum mechanics/molecular mechanics to investigate the catalytic strategies for the sequential oxidation reactions of KDM6B with different *N*-alkylated forms of lysine K27 in the H3 peptide chain, that is *N* ^ε^, *N* ^ε^-methyl ethyl lysine (**Lys­(Me/Eth)**) and *N* ^ε^-isopropyl lysine (**Lys­(iPr)**). The results for sequential oxidations, which yield alcohol, aldehyde, and then carboxylic acid products, reveal that variations in the conformational positioning of different *N*-alkylated groups are enabled by second coordination sphere (SCS) interactions and long-range correlated motions. Specifically, access of the different *N*-alkylated groups to the reactive Fe­(IV)=O intermediate, leading to hydroxylation, is controlled by a network of SCS interactions, in particular involving N344 and Y239, which was also demonstrated by MD and QM/MM calculations on N344A and Y239A mutants. Subsequent oxidations of the alcohols to aldehyde and acid derivatives are also guided by the conformational positioning of the hydroxylated/aldehyde substituent. QM/MM calculations predicted regio- and chemo-selective oxidation can be initiated through hydrogen atom transfer involving σ- or π-mechanisms. The insights would guide experimental efforts to design Fe­(II)/2OG enzymes with non-native catalytic activities and altered substrate selectivity. Furthermore, the results reveal mechanistic features that can be leveraged to design biocatalytic platforms for the selective functionalization of peptide-based drugs.

## Graphical abstract

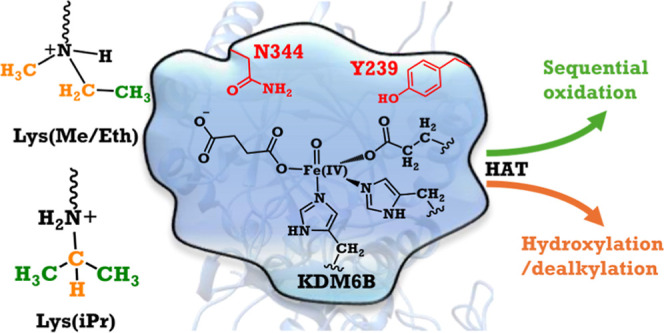

## 1 Introduction

The activation of C–H bonds represents a fundamental challenge for biological and synthetic catalysis.^1,2^ C–H bonds are among the least reactive due to their high dissociation energies and nonpolar character;^3^ therefore, methods to selectively break and functionalize C–H bonds are an active area of research.^2,4–6^ Transition metal catalysts have been widely implemented to achieve selective C–H bond activation.^7^ However, these catalyst systems often employ harsh reaction conditions, prefunctionalized substrates (directing groups), and/or rare metals like palladium, ruthenium, rhodium, etc., which limit their usage and scalability.^1^ In contrast, transition metal-containing enzymes have evolved to perform similar transformations with high selectivity under physiological conditions, providing metal centers and a preorganized protein environment that enables C–H oxidation.^1,8–10^ Unlike synthetic catalysts, metalloenzymes achieve regio- and stereoselectivity through substrate positioning, electrostatic preorganization, and dynamics of the local environment surrounding the metal center.^11^

Nonheme Fe­(II)/2-oxoglutarate (2OG)-dependent oxygenases are among the most versatile metalloenzymes being capable of performing highly selective oxidation reactions via C–H activation, including hydroxylation, halogenation, desaturation, demethylation, ring openings/closures, epoxidation, and electrophilic aromatic substitution reactions, among others.^9,12–14^ Fe­(II)/2OG-dependent oxygenases play roles in diverse biological processes, including fatty acid metabolism, the hypoxic response, epigenetic regulation, collagen biosynthesis, and transcriptional regulation.^15–19^ In their resting state, the Fe­(II)-center is octahedrally coordinated by a facial triad and water molecules. The binding of 2OG displaces two waters, and substrate binding promotes the removal of the third, creating a site for O_2_ binding.^20–22^ This enables the formation of an Fe­(III)-superoxo species, which reacts with 2OG to produce CO_2_ and an Fe­(II)-peroxide intermediate. O–O bond cleavage then generates the ferryl (Fe­(IV)=O) species and succinate. The ferryl intermediate abstracts a hydrogen atom from the substrate, forming Fe­(III)–OH and a substrate radical, followed by hydroxyl rebound to yield the hydroxylated product and regenerate Fe­(II) (Scheme S1).^10,12^ Both active site residues and second coordination sphere (SCS) residues control the redox properties, spin-state energetics, and the reactivity of the Fe­(IV)=O species, enabling efficient C–H bond activation.^23–27^

Among the nonheme Fe­(II)/2OG-dependent oxygenases, the Jumonji C (JmjC) domain-containing histone demethylases (KDMs) are of particular interest due to their role in epigenetic regulation.^28–32^ The JmjC KDMs catalyze the removal of methylation marks through C–H activation from various lysine residues on histone H3 tails, notably at positions K4 and K36; marks typically associated with transcriptional activation, as well as K9 and K27, which are typically linked to transcriptional repression.^13,14^ Humans contain the KDM2-KDM9 subfamilies of JmjC KDMs with varying methylation state (mono-, di-, or tri-*N* ^ε^-methylated lysines and positional selectivities (including at histone H3 K4, K9, K27, and K36)); some JmjC KDMs also catalyze demethylation of N-methylated arginine residues.^28,33–37^ The human KDM6 subfamily, in particular, is specific toward trimethylated lysine-27 of histone H3.^38–41^

The human KDM6 subfamily comprises KDM6A (UTX), KDM6B (JMJD3) and KDM6C (UTY).^40^ Of these, the observed demethylation activity of KDM6C is low, at least with the substrates reported so far.^41^ KDM6A and KDM6B catalyze the demethylation of both tri- and dimethylated H3K27.^40^ They share substantial sequence similarity (84%), but have differing catalytic activities;^38,39^ the reasons for this have been explored in a reported computational study.^42^ The fold of the KDM6B contains a JmjC domain, a linker domain, and a Zn­(II)-binding domain (Figure 1a).^38^ The JmjC domain of KDM6B contains the Fe­(II)-binding (H250, E252, H330) and substrate binding sites. The Zn­(II)-binding domain is reported to be crucial for the H3-substrate binding to the KDM6B protein.^38^ Apart from its established trimethylated H3 lysine 27 (H3K27) (**Lys­(Me3)**) substrate, studies with isolated KDM6B have shown it can catalyze oxidative dealkylation of a variety of N^ε^-alkylated H3K27 derivatives, including *N* ^ε^, *N* ^ε^-methyl ethyl lysine (**Lys­(Me/Eth)**), *N* ^ε^-isopropyl lysine (**Lys­(iPr)**), *N* ^ε^, *N* ^ε^-methyl isopropyl lysine (**Lys­(Me/iPr**)), and *N* ^ε^, *N* ^ε^-diethylated lysine-derivatives (**Lys­(Eth2)**) (Figure 1b).^43^ KDM6B can remove both methyl and ethyl groups from the **Lys­(Me/Eth)** substrate, with the methyl group being removed more readily. In addition, KDM6B was reported to catalyze, albeit less efficiently, subsequent oxidations of the ethyl group of **Lys­(Me/Eth)**, leading to alcohol, then aldehyde, and carboxylic acid products (Figure 1c).^43^ Similar reactivity was observed with the **Lys­(iPr)** substrate, with deisopropylation being the major reaction, with minor reactions leading to consecutive oxidation products of the isopropyl mark. The demonstrated ability for consecutive oxidations catalyzed by KDM6B suggests a mechanistic similarity with Fe­(II)/2OG oxygenases acting on nucleic acids and those involved in small molecule biosynthesis, where they catalyze a very wide range of oxidative reactions.^44–51^ A notable example of the latter is the Fe­(II)/2OG oxygenase-catalyzed sequential oxidation of an alkyl group to an alcohol, then an aldehyde, and further oxidation during gibberellin biosynthesis.^52,53^ Related reactions can also occur during the biosynthesis of cephalosporins.^54^ In the context of nucleic acid modification, TET2 exemplifies similar oxidative versatility by catalyzing stepwise oxidation of 5-methylcytosine to 5-hydroxymethylcytosine, 5-formylcytosine, and 5-carboxylcytosine, demonstrating its ability to process alkylated substrates through sequential oxidation.^47,49^ Likewise, the AlkB family of Fe­(II)/2OG oxygenases further expands this mechanistic paradigm, as these enzymes can oxidatively repair alkylated and exocyclic nucleobases.^17,48,50^ Together, these examples highlight that KDM6B fits within a broader mechanistic framework in which Fe­(II)/2OG oxygenases carry out diverse, often consecutive, oxidative transformations across proteins, nucleic acids, and small-molecule substrates. Extensive computational studies have elucidated the catalytic strategies of TET and AlkB enzymes,^49,50,55–57^ particularly their ability to oxidize alkylated bases and exocyclic nucleobase adducts. Although sequential oxidation by TET2 has been modeled in detail,^56^ comparable mechanistic insight for the histone demethylase KDM6B remains limited, underscoring the need for deeper computational investigation of its catalytic versatility.

**Figure 1:**
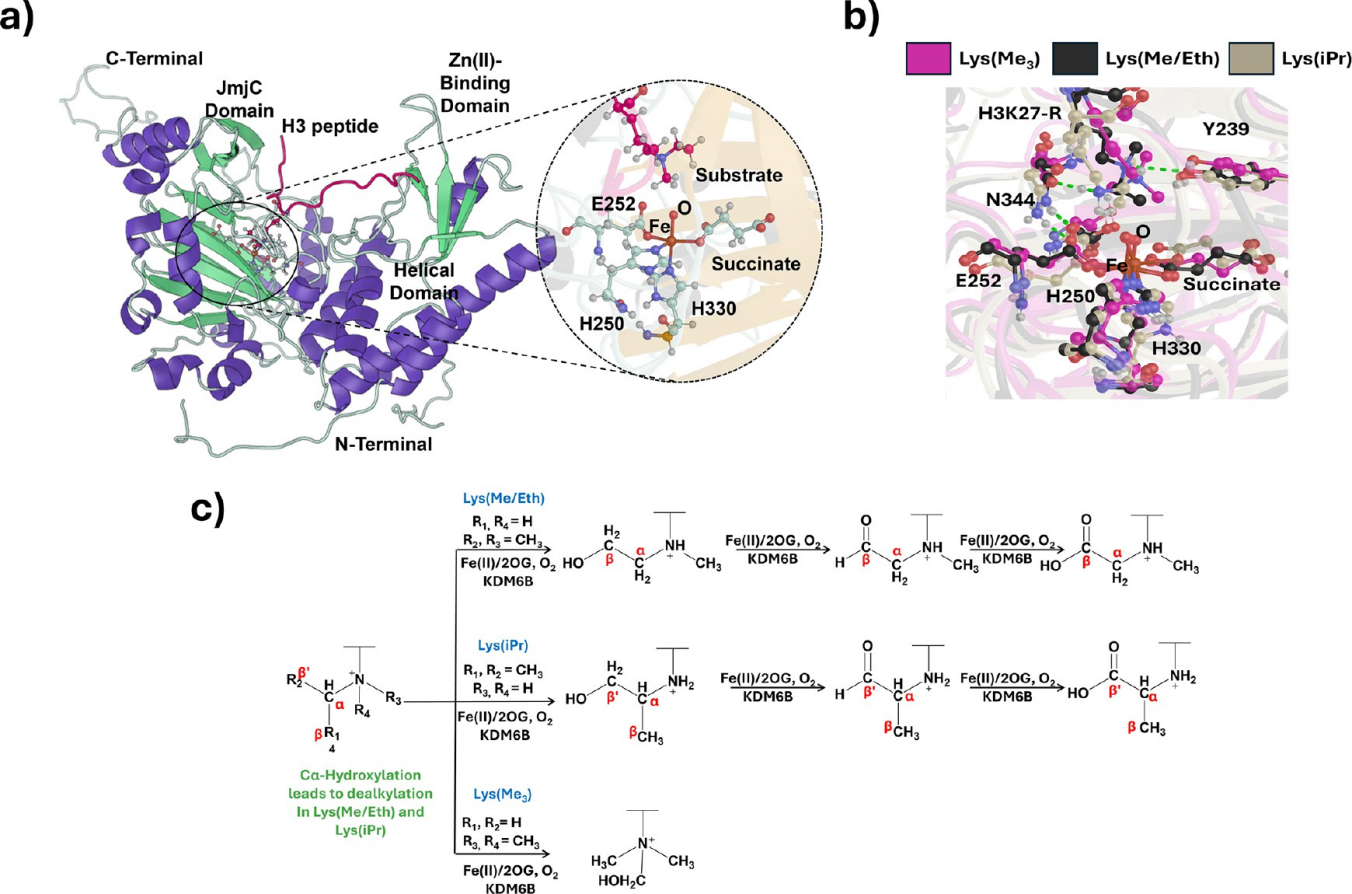
KDM6B catalyzes reactions beyond *N*-methyl demethylation. (a) View derived from a crystal structure of KDM6B (PDB ID: 5oy3) modeled with Fe­(IV)=O, succinate, and an H3 peptide substrate with **Lys­(Me/Eth)**. The three domains of the KDM6B protein are labeled. The inset shows an enlarged view of the KDM6B active site showing the Fe­(IV)=O group, Fe-chelating succinate, and the facial triad. (b) Overlaid structures of KDM6B with Lys­(Me_3_), **Lys­(Me/Eth)**, and **Lys­(iPr)**. (c) Enzymatic conversions catalyzed by KDM6B with different *N*-alkylated Lys substrates.^43^

The *N*-methyl group removal, as catalyzed by KDMs, follows the consensus catalytic mechanism of nonheme Fe­(II)/2OG oxygenases, as established by spectroscopic, kinetic, and computational studies (as described above).^10,20,58–69^ In the subsequent step, where a hydroxylated alkyl group is converted to an aldehydic group, Fe­(IV)=O species formed through the canonical O_2_-activation reaction pathway might abstract two hydrogens; one from the hydroxo group and one from the adjacent carbon to form the aldehyde group (Figure 1c). The initial hydrogen atom transfer (HAT) from the hydroxyl hydrogen might yield a substrate-based oxygen-centered radical, which is then followed by a second HAT from the Cβ–H bond (Figure 1c). This could result in the formation of a carbonyl (aldehyde) group with the reduction of Fe­(IV)=O to Fe­(II)–OH_2_. The subsequent oxidation of the aldehyde group to carboxylic acid involves HAT from the aldehyde hydrogen by the Fe­(IV)=O to form Fe­(III)–OH, followed by rebound of the hydroxo group to the carbonyl-based radical.

We are interested in understanding the mechanisms that enable Fe­(II)/2OG oxygenases, such as KDM6B, to catalyze consecutive oxidations, in particular by integrating molecular dynamics (MD) with quantum mechanics/molecular mechanics (QM/MM) calculations. By investigating how different *N*-alkylated lysine substrates, such as **Lys­(Me/Eth)** and **Lys­(iPr)**, interact with the enzyme’s active site and SCS, we aimed to define the determinants of substrate selectivity, product distribution, and the role of active-site dynamics in controlling reactivity. In addition to probing the initial hydroxylation step, we also explored the sequential oxidations catalyzed by KDM6B, including the conversion of hydroxyalkyl intermediates to the corresponding aldehyde and acid products. Understanding of KDM6B catalysis with non-natural alkylations of H3K27 would help the efforts for selective functionalization of peptides via introducing non-natural side chain modifications, which is an important technique for modulating the potency, proteolytic stability, and target specificity of peptide-based drugs.^70,71^ Overall, such mechanistic insights would deepen our fundamental understanding of KDM catalysis and may ultimately aid future efforts in enzyme engineering to design biocatalytic tools for selective functionalization of peptide-based drugs.

## 2 Methods

### 2.1 System Preparation

The initial structure for the KDM6B protein was obtained from the RCSB database (PDB ID: 5oy3).^38^ The structure obtained contains Fe coordinated by 2OG, H250, E252, and H330, with the sixth coordination site occupied by a water molecule. Necessary modifications were made, including replacing 2OG with succinate and the water molecule with oxygen to model the ferryl intermediate (Fe­(IV)=O) (Figure 1a). The H3K27me2/3 substrate residue was modified to represent the different *N* ^ε^-alkylated substrates studied in this work by replacing hydrogens attached to *N* ^ε^ with methyl (Me), ethyl (Eth), or isopropyl (iPr) groups, dependent on the combination of interest, like **Lys­(Me/Eth)** and **Lys­(iPr)**. The protonation states of the system were determined using Amber routines.^72^ The missing residues in the crystal structure were modeled using the MODELER suite^73^ implemented in Chimera.^74^ The parameters for the different unnatural lysine substrates with *N*-alkyl modifications were obtained using the generalized Amber force field (GAFF)^75^ in the Antechamber tools from Amber 20.^72^ The metal center parameters for the Fe­(IV)=O group bonded to H250, E252, H330, succinate, and oxygen were obtained using the Metal Center Parameter Builder (MCPB.py)^76^ module in AmberTools. We used the high-spin (HS) quintet state of Fe as the ground state, as studies have shown that nonheme Fe­(II)/2OG oxygenases prefer this spin state for reaction.^21,36,49,55,62,67,77–79^ The parameters for Zn­(II) bonded to four cysteines in the Zn­(II)-binding domain were modeled using Amber’s Zinc Amber Force Field (ZAFF) method.^80^ The modeled proteins with different substrates were then individually solvated with a rectangular box of TIP3P^81^ water molecules with a cutoff of 10 Å from the farthest atom on the protein surface. Modeling and subsequent MD and QM/MM simulations followed the same protocol as used in our previous studies on the mechanism of WT KDM6B to ensure consistency.^42^

### 2.2 Molecular Dynamics Simulations

To remove unfavorable contacts between the solvent and the solvated systems, we implemented minimizations in two steps: first, only the solvent molecules were minimized with a strong restraint of 500 kcal mol^–1^ Å^–2^ on the protein; then, the whole system was minimized without any constraints. The minimizations were divided into 5000 steps of the steepest descent algorithm, followed by 5000 steps of the conjugate gradient algorithm. The SANDER (CPU version of Amber20) code was used for minimization simulations. The systems were then gently heated from 0 to 300 K in an *NVT* ensemble using a Langevin thermostat^82,83^ with a collision frequency of 1 ps^–1^ for 250 ps with a small harmonic potential restraint of 50 kcal mol^–1^ Å^–2^. Following heating, the systems were equilibrated. The particle mesh Ewald method^84,85^ was used to calculate long-range electrostatic interactions with a direct space and van der Waals cutoff of 10 Å, and the bonds involving hydrogens were constrained using the SHAKE algorithm.^86^ Subsequently, systems underwent MD simulations for 1 ns to attain a target pressure of 1 atm with a weak harmonic potential restraint of 5 kcal mol^–1^ Å^–2^ on the protein. The systems were then equilibrated for 3 ns without restraints at 300 K in an *NPT* ensemble. During the simulations, the pressure was maintained at 1 bar using a Berendsen barostat.^87^ All simulations were carried out with periodic boundary conditions. The production simulations were performed for 2 μs for **Lys­(Me/Eth)** and **Lys­(Me/Eth–OH)** in order to ensure proper equilibration of these systems (Figures S3, S33) in an *NPT* ensemble with a time step of 2 fs, with a pressure set at 1 bar and a constant pressure coupling of 2 ps. All other MD simulations were performed for 1 μs under the same conditions. The GPU version of Amber20 was utilized for production MD simulations.^72^ Hydrogen bonds, interatomic distances, and angle data of the MD simulations were analyzed using the CPPTRAJ^88^ module of Amber20. Kernel density estimate (KDE) analysis of the distance vs angle was calculated using a Python script. Principal component analysis (PCA) and Dynamic Cross Correlation Analysis (DCCA) were implemented using the Bio3D^89,90^ package in the R programming language on the equilibrated portion of the production MD simulation.

### 2.3 QM/MM Simulations

We extracted snapshots from well-equilibrated regions of the trajectory using K-means clustering^91^ based on distance metrics and the protein backbone RMSD of all backbone atoms, as implemented in cpptraj,^88^ and selected representative structures from the most populated clusters. Additional details of the clustering procedure are provided in the Supporting Information (page S9, Figures S1 and S2). Water molecules beyond 12 Å from each atom on the protein surface were truncated. The ChemShell program^92^ was used for QM/MM simulations, utilizing Turbomole^93^ for QM calculations and DL-POLY^94,95^ for MM implementation. The electrostatic embedding scheme^96^ was utilized to account for the polarizing effect of the MM region on the QM region. The QM/MM boundaries were capped using hydrogen link atoms by implementing the charge shift^96^ method. The facial triad residues with Fe­(IV), along with succinate, oxygen, and the alkylated lysine substrate truncated along the Cγ-Cδ bond, were included in the QM region (Figure 1a). Protein residues and water molecules within 8 Å were included in the flexible MM region, and the system beyond 8 Å was fixed. The Amber ff14SB force field^97^ was used to define the MM region. Experimental and computational studies have shown that the reaction proceeds through the quintet state for the nonheme Fe­(II)/2OG oxygenase systems. Hence, we used the HS quintet state to represent the QM region for our reaction path calculations. Def2-SVP^98^ basis set was used for the QM/MM geometry optimizations using the DFT method with the B3LYP functional (QM­(B1)/MM). The potential energy scans (PES) were carried out using a specific reaction coordinate with a step size of 0.1 Å starting from the optimized reaction complexes (RCs) to obtain transition states (TSs), intermediates (IMs), and product complexes (PCs). The DL-FIND optimizer was used for geometry optimizations. The TSs were optimized without any constraints using the dimer method^99^ implemented in the DL-FIND optimizer.^100^ Further refinement of energies was obtained by calculating single point (SP) energy calculations on the optimized geometry using a higher all-electron basis set def2-TZVP (QM­(B2)/MM).^98^ The zero-point energies obtained from frequency calculations on the optimized geometries were added to the B2 energy to obtain zero-point corrected QM­(B3)/MM energies. All the reaction path calculations in the subsequent sections were discussed at the QM­(B3)/MM level of energy. Spin Natural Orbital (SNO) analysis was carried out using the Gaussian 16 package^101^ from the electronic structure obtained from QM/MM optimizations.

## 3 Results and Discussion

### 3.1 How Conformational Dynamics Facilitate the Formation of Reactive Complex between KDM6B–Fe­(IV)=O and *N*-Alkylated H3-Lysine Substrates?

The catalytic machinery of Fe­(II)/2OG-dependent oxygenases depends on the different states of the nonheme iron center.^12^ The important C–H activation step is carried out by the reactive Fe­(IV)=O intermediate. To investigate the role of substrate conformational flexibility in dictating the C–H activation followed by oxidation of different alkyl groups, we performed comparative MD simulations for KDM6B at the Fe­(IV)=O intermediate level with the **Lys­(Me/Eth)** and **Lys­(iPr)** substrates.

#### 3.1.1 Methyl Ethyl Lysine (Lys­(Me/Eth)) Substrate

Molecular dynamics simulations were conducted for 2 μs to obtain a well-equilibrated trajectory of KDM6B–Fe­(IV)=O•Lys­(Me/Eth) (Figure S3). The **Lys­(Me/Eth)** substrate simulations predicted that access to the alkyl groups was less dynamic than the **Lys­(Me**_**3**_ **)** substrate. The methyl group was consistently closer to the Fe­(IV)=O group than the ethyl group. The histogram analysis of O^......^C (methyl carbon or ethyl carbons) indicates a clear preference for the methyl group over the ethyl group (Figure S4). However, the ethyl group also has conformations with a closer approach to the Fe­(IV)=O in a significant portion of the trajectory. This observation aligns with the experimental finding that methyl groups are more readily removed from the **Lys­(Me/Eth)** substrate than the ethyl group.^43^ The residues that define the preference of the alkyl groups in the active site include N344 and Y239. N344 forms hydrogen bonds with the amino nitrogen of the **Lys­(Me/Eth)** substrate when the methyl group is closer to the Fe­(IV)=O oxygen. When the N^ε^-methyl group becomes close to the Fe center, the N^ε^-H group forms hydrogen bonds with one oxygen of the iron-binding E252 side chain. Conversely, Y239 hydrogen bonds with the N^ε^-H group of the **Lys­(Me/Eth)** substrate when the ethyl group is closer to the Fe center. It can therefore be argued that these residues function as gatekeeper residues, facilitating the chemo-selectivity of the KDM6B in hydroxylating methyl and ethyl groups from the **Lys­(Me/Eth)** substrate.

Furthermore, the C4 carboxylate of succinate was observed to form a consistent salt–bridge interaction with K241 and a hydrogen bonding interaction with the hydroxo group of T247 in most parts of the KDM6B–Fe­(IV)=O•Lys­(Me/Eth) trajectory, consistent with the calculation on **Lys­(Me3)** dynamics at the same intermediate state.^42^ However, the hydrogen bond between iron-bound E252 and the hydroxo group of S258 observed in the **Lys­(Me3)** dynamics was lost entirely in the KDM6B–Fe­(IV)=O•Lys­(Me/Eth) dynamics, due to the bulkier ethyl group in the case of the **Lys­(Me/Eth)** substrate.

Experimental studies have demonstrated that the position of C–H activation in the alkyl group determines the product distribution in histone demethylases. For example, the hydroxylation at the methylene carbon (Cα) of the ethyl group of **Lys­(Me/Eth)** leads to its decomposition to acetaldehyde,^102^ while the C–H activation at the methyl carbon (Cβ) of the ethyl might lead to consecutive oxidations.^43^ Consequently, it is crucial to comprehend the preference of the KDM6B enzyme in accessing the methylene or methyl carbons of the ethyl group of the **Lys­(Me/Eth)** substrate for C–H activation. The population distribution of O^. . ..^Cα and O^. . ..^Cβ distances of ethyl indicates that both the carbons are equally accessible to the Fe­(IV)=O, with a slight preference for the Cα (Figure S4). This observation is consistent with the experimental observation that product distribution with **Lys­(Me/Eth)** substrate favors dealkylated lysine (**Lys**) followed by demethylated **(Lys­(Eth))** and de-ethylated lysine **(Lys­(Me))**.

We performed PCA to investigate the dynamic behavior of KDM6B in the presence of the **Lys­(Me/Eth)** substrate. PCA revealed three significant regions of KDM6B/**Lys­(Me/Eth)** complex, which showed distinct, flexible motions: (i) the residues that form β16, β17 and the loop connecting them (447–461), (ii) the residues that form β15 and the loop connecting it with β16 (424–433) and (iii) the disordered region from residues 152 to 184 which surrounds the double-stranded beta helix (DSBH) fold of the active site (Figure 2a). Regions (i) and (ii) showed motions moving away from the H3 substrate, a motion that was not observed in the **Lys­(Me3)**system (Figure S5). Notably, these regions surround the Zn­(II)-binding region, which was experimentally demonstrated to be involved in H3 substrate recognition.^43^ Given that **Lys­(Me/Eth)** is a less efficient substrate than **Lys­(Me3)** with respect to dealkylation,^43^ but uniquely undergoes sequential oxidation by KDM6B, the increased flexibility of these regions may reflect altered dynamics that both reduce dealkylation efficiency relative to **Lys­(Me3)** and enable the sequential oxidation pathway that is absent in the **Lys­(Me3)** (Figure S5).^43^

**Figure 2:**
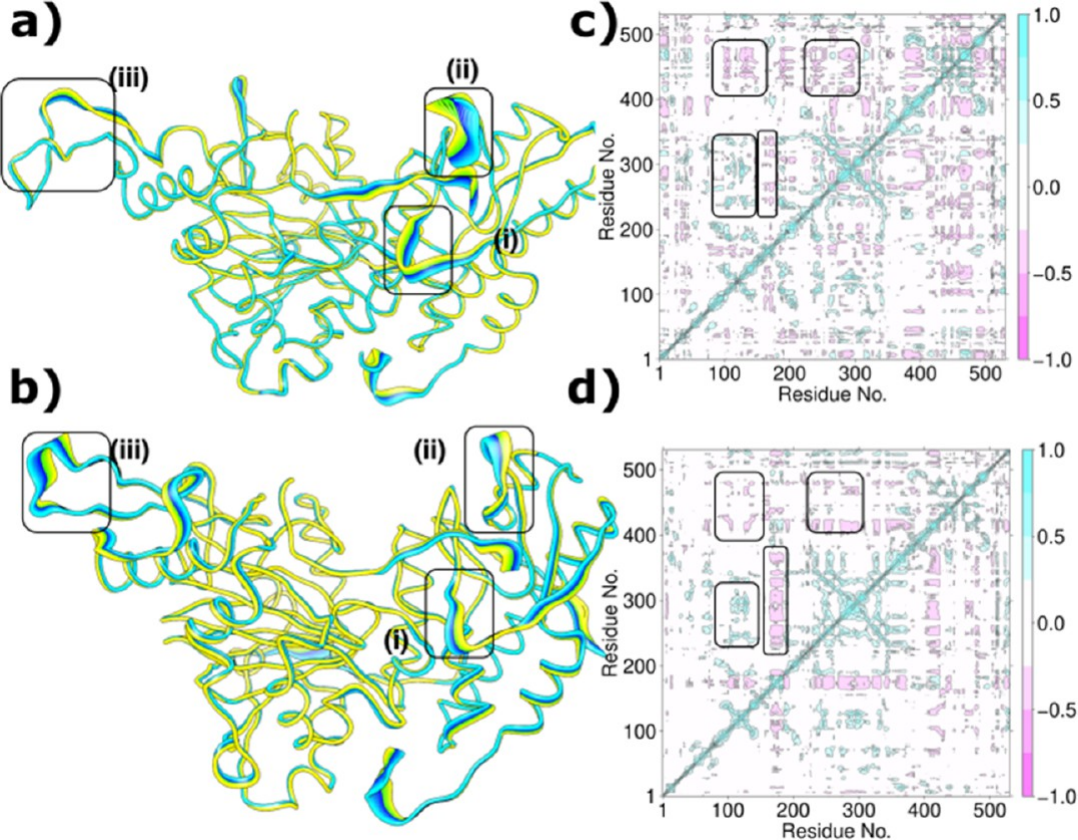
Conformational behavior of KDM6B–Fe­(IV)=O intermediate with **Lys­(Me/Eth)**, and **Lys­(iPr)** substrates. PCA showing the dominant motions of the KDM6B enzyme with (a) **Lys­(Me/Eth)** and (b) **Lys­(iPr)** substrates. DCCA plot showing the correlated/anticorrelated motions involved in the KDM6B with (c) **Lys­(Me/Eth)** and (d) **Lys­(iPr)** substrates. Boxed areas show correlated and anticorrelated motions of the flexible regions.

Further, we explored the interdependent correlated/anticorrelated motions exhibited by the flexible regions of the KDM6B protein using DCCA. The regions (i) and (ii) exhibited anticorrelated motions with the loop connecting β6 and β7, which contains Y239, which is directly involved in substrate binding (Figure 2b). Region (iii) exhibited correlated motions with the residues that form α14, α15, (274–290), and β10 (268–273). The H3 substrate residues are anticorrelated with regions (i) and (ii) and correlated with region (iii); this follows what was observed in **Lys­(Me3)** dynamics (Figure S5). Notably, the disordered region in KDM6B (residues 152–184), which was predicted to have an anticorrelated motion with the DSBH fold in the **Lys­(Me3)** system,^42^ has reduced anticorrelated motion in the **Lys­(Me/Eth)** system (Figure 2b). Overall, the DCCA analysis showed less intense correlated/anticorrelated motions in the KDM6B–Fe­(IV)=O•Lys­(Me/Eth) system compared to the KDM6B–Fe­(IV)=O•Lys­(Me3) system, which could, at least in part, contribute to both the reduced dealkylation efficiency of **Lys­(Me/Eth)** and its ability to undergo sequential oxidation, which is absent in the **Lys­(Me3)** system.

Overall, the conformational analysis of KDM6B–Fe­(IV)=O•Lys­(Me/Eth) provided insights into the substrate binding interactions, highlighting flexible regions of the protein and the correlated motions associated with them, which are likely involved in substrate recognition and reactivity.

#### 3.1.2 Isopropyl Lysine (Lys­(iPr)) Substrate

Biochemical studies on KDM6B with the **Lys­(iPr)** substrate have demonstrated that the isopropyl group is readily removed. Apart from dealkylation, the enzyme also catalyzes consecutive oxidations on the methyl groups of the isopropyl group, leading to alcohol, aldehyde, and carboxylic acid products.^43^ It has been demonstrated that the hydroxylation at the tertiary isopropyl Ca leads to dealkylation, while C–H activation at a methyl group leads to consecutive oxidations.^43^ To understand the **Lys­(iPr)** substrate dynamics bound to the KDM6B enzyme at the Fe­(IV)=O intermediate state, we conducted MD simulations to obtain a well-equilibrated one μs trajectory of KDM6B–Fe­(IV)=O•Lys­(iPr) (Figure S6). In contrast to the KDM6B-**Lys­(Me**_**3**_ **)**, with **Lys­(iPr)**, the Fe­(IV)=O could consistently access the sterically hindered isopropyl Ca and one of the two methyl groups of the isopropyl group. The histogram analysis of O^. . ..^Cα, O^. . ..^Cβ, and O^. . ..^Cβ′ distances of the isopropyl group revealed a clear preference for Cα oxidation followed by either of the two β carbons (Figures S7 and S8). Thus, the conformational dynamics of the **Lys­(iPr)** substrate align with the experimental product distribution obtained, which indicates a predominant dealkylation, followed by the sequential formation of alcohol, aldehyde, and acid products of the isopropyl group.^43^ As with **Lys­(Me/Eth)**, the SCS residue N344 forms hydrogen bonds with the N^ε^-H group of the **Lys­(iPr)**, which determines the conformational stability of the substrate with a clear preference for the Cα of isopropyl, followed by the methyl carbons (Figure S7). Atomistic analysis revealed that A342 and Y239 stabilized the isopropyl group of **Lys­(iPr)** through hydrophobic interactions. In contrast to the **Lys­(Me/Eth)** dynamics, there were no interactions of the N^ε^-H group of **Lys­(iPr)** with Y239. However, consistent with the **Lys­(Me3)** ^42^ and **Lys­(Me/Eth)** dynamics, the C4 carboxylate of succinate was stabilized by salt bridge interactions with K241 and hydrogen bonding interactions with the hydroxo group of T247.

Similar to the **Lys­(Me/Eth)** dynamics, PCA analysis with KDM6B–Fe­(IV)=O•Lys­(iPr) showed regions (i) and (ii) were the most dominant flexible regions; however, region (iii) showed less flexibility; instead, the disordered region from residues 152–184 gained more flexibility (Figure 2b). The changes in the flexibility were also reflected in the interdependent correlated/anticorrelated motions of these regions. The negatively correlated motion exhibited between regions (iii) with the regions (i) and (ii) was lost, while the positively correlated motion existed between region (iii) and the regions that form α14, α15, (274–290), and β10 (268–273) showed less intensity in **Lys­(iPr)** system compared to **Lys­(Me/Eth)** dynamics (Figure 2d). The negatively correlated motion that existed between regions (i) and (ii) with the α14, α15, (274–290), and β10 (268–273) in the **Lys­(Me/Eth)** system was absent in the **Lys­(iPr)** dynamics. In contrast to the **Lys­(Me/Eth)** dynamics, the disordered region 152–184 showed intense anticorrelated motion with the DSBH folds in the **Lys­(iPr)** system, similar to what was predicted in **Lys­(Me3)** dynamics.^42^ Overall, the **Lys­(iPr)** has caused significant changes in the correlated/anticorrelated motions involved with the KDM6B enzyme, which could help in the substrate binding and the orientation for effective catalysis.

Overall, the conformational dynamics of the **Lys­(Me/Eth)** and **Lys­(iPr)** revealed substrate-specific SCS interactions and long-range (LR)-correlated motions with KDM6B. The overlaid structures in Figure 1b clearly demonstrate the role of N344 and Y239 in defining the substrate binding orientation.

### 3.2 Mechanism of KDM6B-Catalyzed Hydroxylation

MD simulations have predicted differential access of the alkyl groups of the N^ε^-substituted lysines **Lys­(Me/Eth)** and **Lys­(iPr)** to the Fe­(IV)=O intermediate. To investigate this, we carried out QM/MM simulations on the Fe­(IV)=O intermediate obtaining complex snapshots obtained from different parts of the trajectory, considering the substrate orientation-for example, methyl closer or ethyl closer to the Fe­(IV)=O in **Lys­(Me/Eth)** substrate.

#### 3.2.1 KDM6B-Catalyzed Hydroxylation of Lys­(Me/Eth)

Experimental studies have shown that KDM6B can catalyze the demethylation and de-ethylation of **Lys­(Me/Eth).** ^43^ Mass spectrometric analyses also demonstrated that the methyl group of the ethyl group undergoes sequential oxidations, leading to alcoholic, aldehydic, and acid products.^43^ To investigate the mechanism of hydroxylation, we obtained snapshots from various portions of the MD trajectory of the KDM6B–Fe­(IV)=O•Lys­(Me/Eth) and optimized them using the QM/MM method. The reactive Fe­(IV)=O species abstracts the hydrogen from the methyl group with a barrier of 23.0 kcal mol^–1^ through MEL1-TS1, leading to Fe­(III)–OH intermediate (MEL1-IM1-meth) (Figure 3a). In MEL1-RC, the methyl group was closer compared to the ethyl group to the Fe­(IV)=O center, with an O–H (methyl) distance of 2.14 Å and a Fe–O–H angle of 106.7° (Figure 4). At MEL1-TS1, the O–H distance was 1.14 Å, and the C–H distance was 1.38 Å, whereas at MEL1-TS2-meth, the O–H distance reduced to 0.98 Å, and the C–H distance increased to 2.99 Å. At MEL1-IM1-meth, the O–C (methyl) distance was 2.80 Å (Figure 4). OH-rebound takes place with a barrier of 5.2 kcal mol^–1^ to form a hydroxylated methyl group in **Lys­(Me/Eth)** (MEL1-PC-meth) (Figure 3a). During the entire methyl hydroxylation process, the N^ε^ of **Lys­(Me/Eth)** forms a hydrogen bond with the nonbonded oxygen of iron-binding E252, which also forms a hydrogen bond with N344 (Figure 4). Furthermore, from the same initial structure, MEL1-RC, we tried HAT from the β-carbon (Cβ) of the ethyl group of the **Lys­(Me/Eth)** substrate. The reaction proceeds with a barrier of 28.1 kcal mol^–1^ to form MEL1-IM1-eth, which is higher than that of methyl HAT. During ethyl Cβ HAT, the substrate undergoes a conformational change to access the Fe­(IV)=O species, which leads to the loss of the hydrogen bond between N^ε^ of **Lys­(Me/Eth)** and the nonbonded oxygen of E252 (Figure 4). However, the Cβ of ethyl returns to the original conformation after hydrogen transfer to the oxygen with a Cβ-O distance of 5.53 Å (Figure 4).

**Figure 3:**
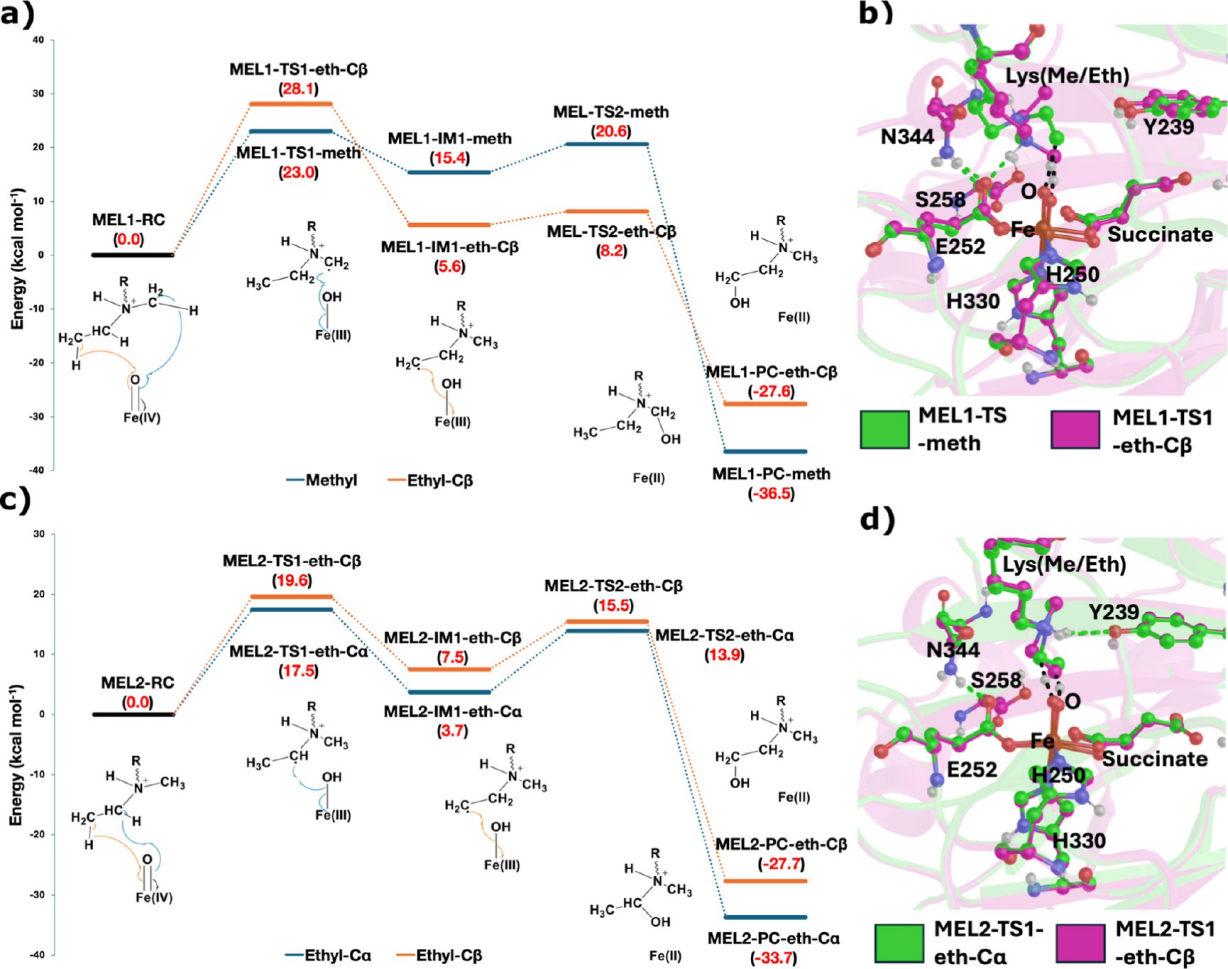
(a) Reaction profile of the hydroxylation of methyl and ethyl groups from WT-MEL1-RC snapshot (b) Overlaid TSs obtained during methyl HAT and ethyl Cβ HAT, (c) reaction profile of hydroxylation of Cα and Cβ carbons of ethyl from MEL2-RC snapshot, and (d) Overlaid TSs obtained during ethyl Cα and Cβ HAT. Relative energies are presented at the QM­(B3)/MM level. Hydrogen bonds are represented in green dashed lines. TS partial bonds are represented in black dashed lines. Nonpolar hydrogens are hidden for clarity.

**Figure 4:**
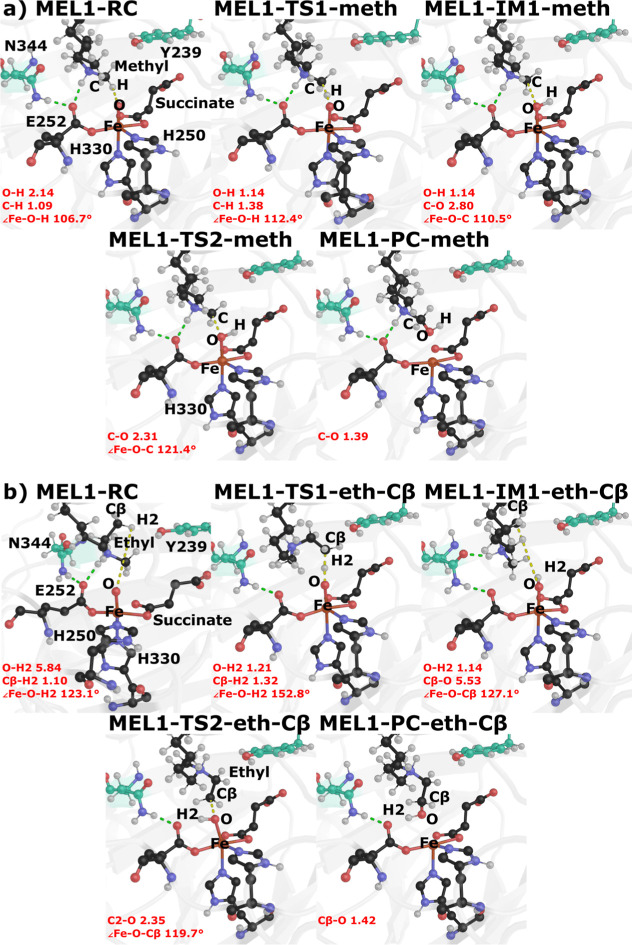
QM/MM optimized stationary points obtained during PES simulations from MEL1-RC during hydroxylation of: (a) methyl, and (b) ethyl Cβ carbons. Distances are given in Å and represented by yellow dashed lines. Hydrogen bonding interactions: green dashed lines.

Despite the more considerable Cβ-O distance at MEL1-IM1-eth-Cβ, the rebound hydroxylation required only 2.5 kcal mol^–1^ activation energy (Figure 3). Our simulations on MEL1-RC predicted that the hydrogen bond between N^ε^ of **Lys­(Me/Eth)** and the nonbonded oxygen of E252 plays a significant role in the regioselectivity of hydroxylation. Overlaid TS states obtained during the hydroxylation of methyl and ethyl groups, depicted in Figure 3b, show the difference in the SCS interactions during different oxidations. The results indicate that the hydrogen bond between N^ε^ of **Lys­(Me/Eth)** and the nonbonded oxygen of E252 should be disturbed for hydroxylation in the ethyl group. To explore hydroxylation when the ethyl group was closer to the Fe­(IV)=O intermediate, we optimized another snapshot to obtain MEL2-RC. In this snapshot, the ethyl group is closer to Fe­(IV)=O species with O–H1 (Ethyl Cα) and O–H2 (Ethyl Cβ) distances of 2.49 Å and 3.21 Å, respectively (Figure 5). As HAT from Cα could lead to hydroxylation at the Cα carbon, which would lead to the removal of the ethyl group, forming acetaldehyde, as suggested by experimental studies,^43^ we simulated hydroxylation at Cα. The Cα-HAT proceeded with a low barrier of 17.5 kcal mol^–1^, leading to MEL2-IM1-eth-Cα with a radical on the Cα carbon (Figure 3c). Following HAT, the OH group rebounds with a barrier of 10.1 kcal mol^–1^ to form a Cα-hydroxylated ethyl group (Figure 3c). We performed HAT from the Cβ in MEL2-RC as in MEL1-RC. The reaction required a 19.5 kcal mol^–1^ barrier through a MEL2-TS1-eth-Cβ to form MEL2-IM1-eth-Cβ (Figure 3c). Subsequent rebound hydroxylation proceeded with a barrier of 7.9 kcal mol^–1^ to form a Cβ-hydroxylated ethyl group (Figure 3c). During ethyl hydroxylations, the N^ε^-H group of **Lys­(Me/Eth)** forms a hydrogen bond with Y239 (Figures 3d and 4b). The barriers obtained from our simulations align well with the experimental observations, as Cα-hydroxylation requires a lower barrier than Cβ-hydroxylation, which suggests that Cα-hydroxylation followed by de-ethylation is more favored over Cβ-hydroxylation followed by subsequent oxidations.

**Figure 5:**
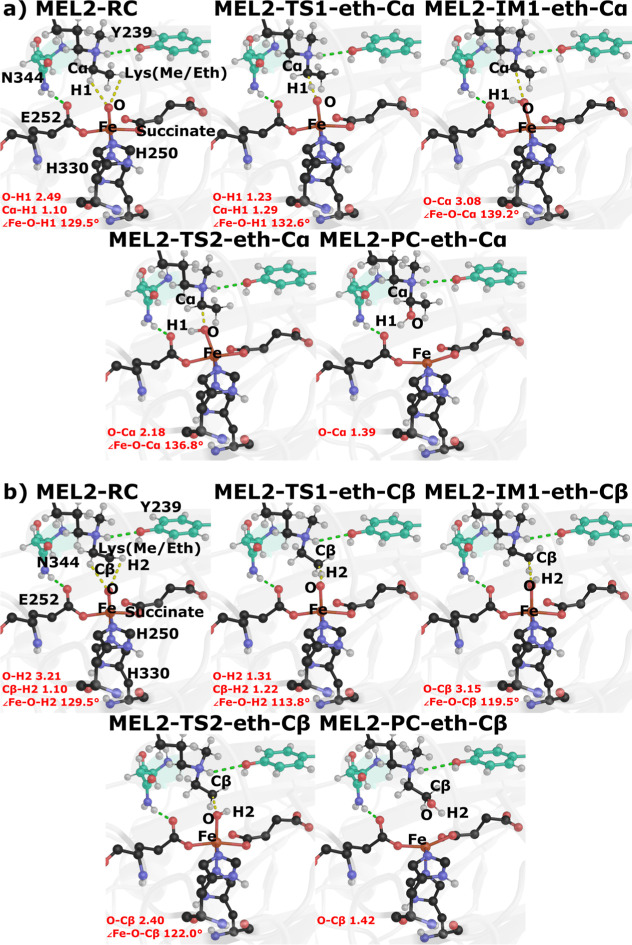
QM/MM optimized structures of stationary points obtained from the MEL2-RC snapshot during hydroxylations of (a) ethyl Cα, and (b) ethyl Cβ carbons. Distances are given in Å and represented by yellow dashed lines. Hydrogen bonding interactions: green dashed lines.

##### 3.2.1.1 Y239A and N344A Reshape Substrate Orientation and Predicted Reaction Preference in KDM6B

To test how the observed SCS interactions stabilize the substrate during the HAT reaction pathway, we examined two SCS mutants, Y239A and N344A, using the **Lys­(Me/Eth)** substrate. In the WT enzyme, the methyl group was closer to the ferryl oxygen for 65% of the MD trajectory, whereas the ethyl group was closer for 35% of the simulation time, consistent with the experimentally observed preference for demethylation (Figure S4). In Y239A, this preference was reversed relative to WT. The ethyl group was closer to the ferryl oxygen for 88% of the trajectory, and 95% of these ethyl-proximal conformations favored Cα positioning (Figures S9 and S10). In these conformations, the substrate N^ε^-H group formed a strong hydrogen bond with the side-chain carbonyl oxygen of Q237 (Figure S11), suggesting that Q237 adopts a gatekeeping role in the absence of Y239. Together, these results indicate that removal of the Y239 side chain reshapes the active-site environment to favor substrate orientations associated with de-ethylation. By contrast, N344A further strengthened the WT-like preference for methyl-directed positioning. In this mutant, the methyl group was closer to the ferryl oxygen for 95% of the trajectory, whereas the ethyl group was closer in only 5% of sampled conformations (Figures S12 and S13). No strong hydrogen bonds between the substrate N^ε^-H group and enzyme residues were observed when the methyl group was proximal to the ferryl oxygen. However, ethyl-proximal conformations in N344A showed a strong hydrogen bond between the substrate N^ε^-H group and the side-chain carbonyl oxygen of Q237 (Figure S14), as in Y239A (Figure S11). These results suggest that alanine substitution at N344 reinforces the preference for demethylation.

To assess the mechanistic consequences of these altered substrate orientations, we carried out QM/MM reaction-path calculations of the catalytically relevant HAT step in both mutants, using the same protocol as for the WT enzyme. For each mutant, two representative snapshots were selected from well-equilibrated regions of the MD trajectories: one in which the methyl group was closer to the ferryl oxygen, denoted MEL1-RC_N344A_ or MEL1-RC_Y239A_, and one in which the ethyl group was closer, denoted MEL2-RC_N344A_ or MEL2-RC_Y239A_. These structures were then used to evaluate the methyl and ethyl HAT pathways.

For N344A, HAT scans from MEL1-RC_N344A_ were carried out for the methyl-group hydrogen and the nearest ethyl-group hydrogen, whereas scans from MEL2-RC_N__344A_ were performed for methyl hydrogen, the ethyl Cα- and Cβ-hydrogens. In MEL1-RC_N__344A_, the methyl HAT barrier was 21.1 kcal/mol (Figure S15), lower than that in the WT enzyme (23.0 kcal/mol) (Figure S15), indicating improved efficiency of demethylation in this mutant. In contrast, the ethyl HAT barriers in MEL2-RC_N344A_ were comparatively unfavorable, with barriers of 26.4 kcal/mol for Cα and 25.2 kcal/mol for Cβ (Figure S16), relative to the WT enzyme, where the corresponding barriers were 17.5 and 19.6 kcal/mol, respectively (Figure 3c). In MEL2-RC_N344A_, the methyl HAT barrier was 28.7 kcal/mol, compared with 25.4 kcal/mol in WT MEL2-RC (Figure S16). Altogether, these results indicate that, in N344A, even when the ethyl group approaches ferryl oxygen closer than the methyl group, de-ethylation is likely less efficient than in the WT enzyme. In the WT active site, the methyl and ethyl HAT pathways are more clearly distinguished by their activation energies, whereas in the N344A mutant, the methyl and ethyl HAT barriers are more closely aligned. This suggests that, although in the ethyl-proximal frames of N344A, de-ethylation remains less favorable than in the respective ethyl-proximal snapshots of the WT enzyme, it is still slightly more favorable than demethylation. Taken together with the MD result that the methyl group is productively oriented for 95% of the trajectory, these findings indicate that the N344A active site is better tuned for demethylation than the WT enzyme.

For Y239A, calculations on MEL1-RC_Y239A_ gave a methyl HAT barrier of 24.5 kcal/mol (Figure S17), whereas the corresponding ethyl HAT barrier was much higher at 39.0 kcal/mol (B1) (Figure S17), indicating that in conformations where the methyl group is closer to the ferryl oxygen, demethylation remains preferred, similar to the WT enzyme. However, in MEL2-RC_Y239A_, evaluation of methyl, ethyl Cα-, and ethyl Cβ-HAT pathways showed that the methyl HAT barrier was 30.2 kcal/mol (Figure S18), whereas the ethyl Cα-HAT barrier was 22.7 kcal/mol (Figure S18). Thus, the ethyl Cα-HAT pathway is more favorable than methyl abstraction in the ethyl-proximal Y239A reactant complex and is also lower in energy than the methyl HAT barriers computed for the Y239A reactant complexes. Together with the MD observation that ethyl-proximal conformations dominate the Y239A ensemble, these results indicate that the Y239A active site is better tuned for de-ethylation than for demethylation, in contrast to the WT enzyme.

Overall, the MD and QM/MM results suggest that Y239 and N344 modulate reaction outcome by controlling the population of substrate orientations that place either the methyl or ethyl group in a productive geometry for HAT. These findings provide an initial computational demonstration that perturbation of SCS interactions can be used to tune reaction preference in KDM6B.

#### 3.2.2 KDM6B-Catalyzed Hydroxylation of Lys­(iPr)

MD simulations of KDM6B–Fe­(IV)=O•Lys­(iPr) predicted that the sterically hindered Cα isopropyl carbon consistently positioned itself closer to the Fe­(IV)=O species compared to the methyl groups (Cβ and Cβ′) of the iPr substituent. To ascertain if this preference manifested in the regioselectivity of hydroxylation at isopropyl or methyl carbons of **Lys­(iPr)** substrate, we conducted QM/MM simulations on snapshots obtained from the equilibrated portion of the MD trajectory.

In the optimized IL1-RC, the O–H1 (isopropyl Cα hydrogen) distance was 2.61 Å, and O–H2 (methyl group Cβ hydrogen of iPr) was 2.95 Å (Figure 6). We explored HAT from both positions of the iPr group. Consistent with the O–H distance, the HAT from Cα hydrogen (H1) required a lower barrier of 20.2 kcal mol^–1^, while the HAT from Cβ hydrogen (H2) required a barrier of 26.6 kcal mol^–1^ (Figure 7). At IL1-TS1-Cα and IL1-TS1-Cβ, the O–H1 and O–H2 distances are 1.31 and 1.18, respectively, with corresponding Fe–O–H1 and Fe–O–H2 angles measured at 125.0° and 105.2° (Figure 6). Furthermore, the IL1-RC-IM1-Cα thus obtained from Cα-HAT is less endothermic with 4.1 kcal mol^–1^ reaction energy relative to the initial IL1-RC compared to the 19.9 kcal mol^–1^ reaction energy of IL1-IM1-Cβ because the secondary carbon radical in IL1-IM1-Cα was more stabilized by the inductive effect due to two methyl groups attached to the Cα-carbon compared to the primary carbon radical in IL1-IM1-Cβ with a single adjacent carbon group. Subsequent rebound hydroxylation at both positions led to IL1-PC-Cα and IL1-PC-Cβ, of which the former, with −36.4 kcal mol^–1^ energy, was more stable compared to −31.5 kcal mol^–1^ energy of the latter (Figure 7a). Thus, we suggest that the Cα-hydroxylation is thermodynamically and kinetically favored over the Cβ-hydroxylation. The preference of Cα-hydroxylation is consistent with the experimental observation, which showed a preference for deisopropylation (Cα-hydroxylation leads to dealkylation) over hydroxylation at methyl groups of the **Lys­(iPr)** substrate.^43^ The SCS residue N344 forms hydrogen bonding interactions with the amino group of the **Lys­(iPr)** substrate and the nonbonded oxygen of iron-binding E252. The interactions were stable throughout the Cα- and Cβ-hydroxylation reaction paths (Figures 6 and 7b). Hence, it could be suggested that this particular SCS residue was crucial in effective substrate positioning and subsequent reaction in **Lys­(iPr)**. Overall, the SCS interactions, kinetic, and thermodynamic factors help the hydroxylation of the sterically hindered Cα position of the **Lys­(iPr)**.

**Figure 6:**
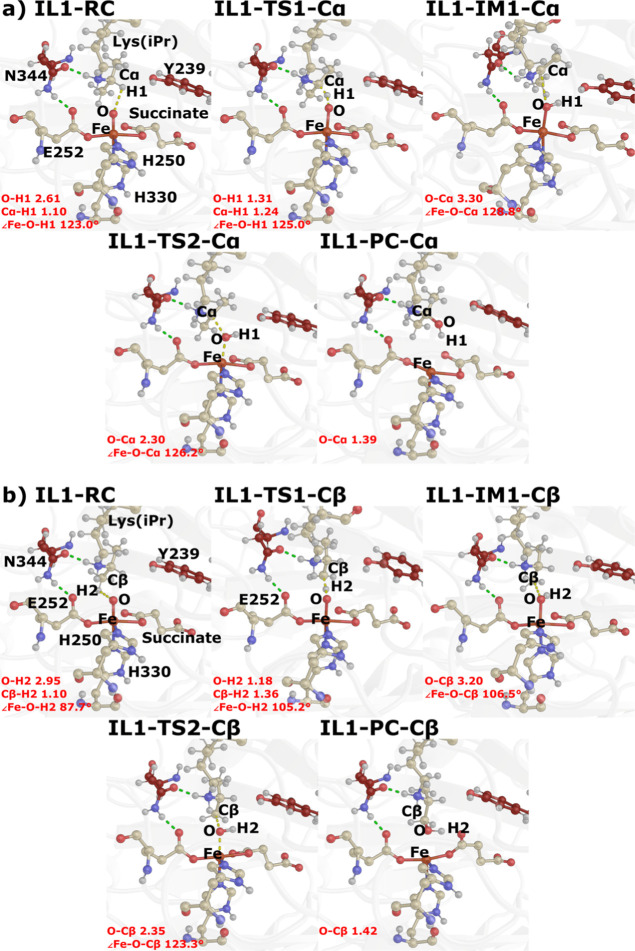
QM/MM optimized stationary points obtained during PES simulations of hydroxylation reactions of (a) Cα and (b) Cβ carbons of the isopropyl group from the IL1-RC snapshot. Distances are in Å and represented by yellow dashed lines. Hydrogen bonding interactions: green dashed lines.

**Figure 7:**
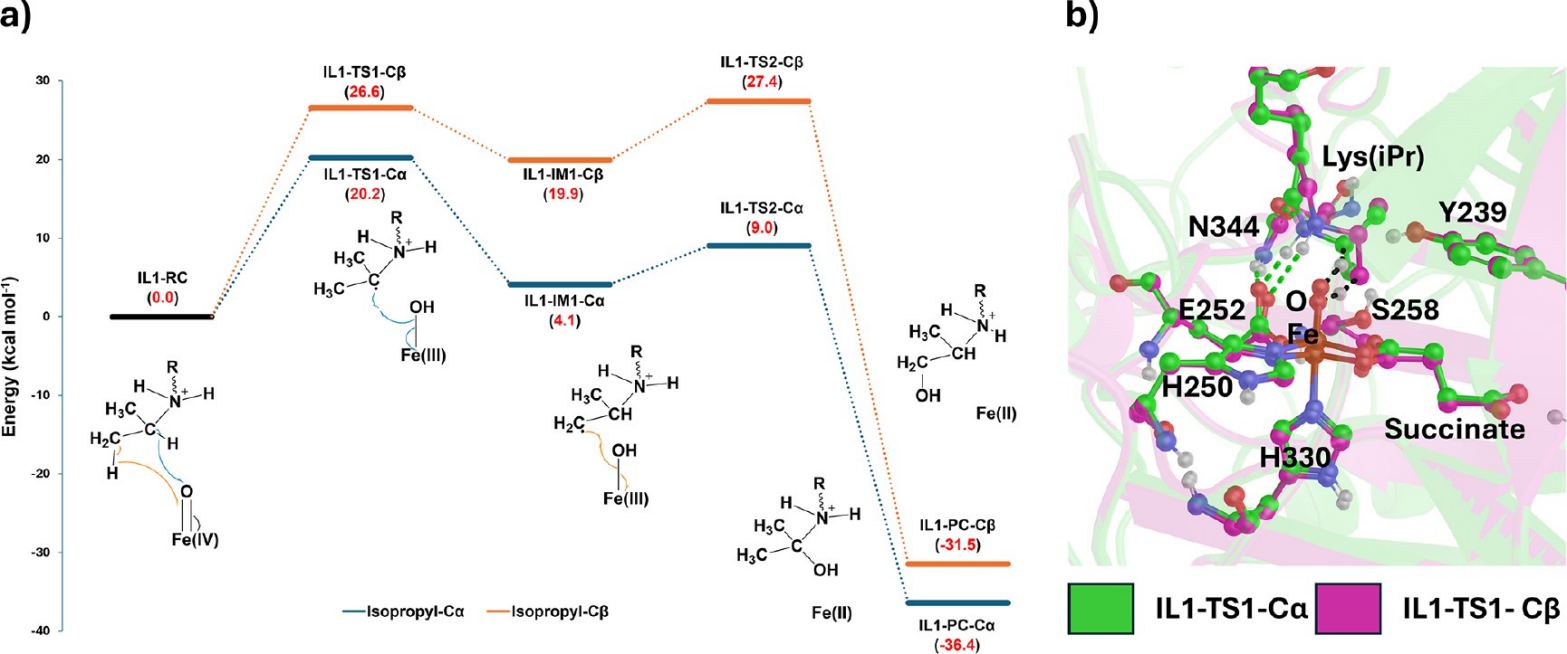
(a) Reaction profile of the hydroxylations of the **Lys­(iPr)**, (b) Overlaid TSs of Cα and Cβ HATs in **Lys­(iPr)**. Relative energies are presented at the QM­(B3)/MM level. Nonpolar hydrogens are hidden for clarity.

#### 3.2.3 Electronic and Conformational Features Responsible for Hydroxylation of Lys­(Me/Eth) and Lys­(iPr)

Electronic and conformational features significantly influence the outcomes of nonheme Fe­(II)/2OG-dependent oxygenase reactions.^23,67,103–106^ Computational studies on nonheme Fe­(II)/2OG enzymes have proposed two possible pathways for electron transfer during HAT from substrate σ_C–H_ bond to Fe center, one in which an α electron moves to the unoccupied antibonding σ*_z_^2^ orbital of the Fe center, requiring a ∠Fe–O–H of ∼180°, leaving a β electron on the substrate,^107–110^ and in the other, a β electron from the σ_C–H_ bond is transferred to one of the singly occupied antibonding π* orbitals (π*_xz_ or π*_yz_), leaving an α electron on the substrate, requiring a more horizontal approach with ∠Fe–O–H of ∼120°. Angles <120° generally favor the π-pathway, while angles >120° favor the σ-pathway. A modified π-pathway has also been proposed in which the α electron enters a π* orbital, followed by excitation to the σ*z ^2^ orbital. Understanding this is important, as substrate dynamics may affect substrate disposition, which in turn affects the ∠Fe–O–H angle and reactivity. Hence, we explored the electronic and conformational features associated with the hydroxylation reactions of **Lys­(Me/Eth)** and **Lys­(iPr)** substrates.

##### 3.2.3.1 Methyl Ethyl Lysine (Lys­(Me/Eth))

Consequently, the electronic structure analysis of the HAT TSs of MEL1-RC revealed that, where the methyl was closer than ethyl, the methyl HAT proceeds through a π-pathway, while the ethyl HAT follows a σ-pathway. The SNO analysis revealed that the electron transfer happens from the σ_C–H_ bond to the π*_yz_ orbital of the Fe center in MEL1-TS1-meth, while in MEL1-TS1-eth, the electron transfers to the σ*_z_^2^ of the Fe center (Figure S19). The spin density analysis of MEL1-IM1-meth and MEL1-IM1-eth-Cβ showed the presence of positive spin density on the methyl carbon with 1.05 and negative spin density of −1.05 on the Cβ carbon of ethyl, respectively (Figure S20). Conversely, in the MEL2-RC, where the ethyl was closer, both the HAT from the Cα and Cβ carbons of ethyl follow the σ-pathway. Similar σ-pathway preference was shown by the hTET2 enzyme in the oxidation of Cα and Cβ carbons of ethylated cytosine residues of the DNA.^111^ The spin density analysis of MEL2-IM1-eth-Cα and MEL2-IM1-eth-Cβ confirms the same, with spin densities of −1.01 and −1.05 on Cα and Cβ, respectively (Figure S21). SNO analyses corroborate the σ-pathway in MEL2-RC, showing electron transfer from the σ_C–H_ bond to the σ*_z_2 of the Fe center in both Cα and Cβ HATs (Figure S22). The FMO calculations show that the energy splitting between the two acceptor orbitals σ_*z*_2 and π*_xz_ was 0.453 eV (10.4 kcal/mol) and 0.493 eV (11.3 kcal/mol) at the MEL1-RC and MEL2-RC, respectively (Figures S23, S24). The energy gap between the acceptor orbitals is reflected in the activation barrier and electron transfer of HAT, as a slight difference between MEL1-RC and MEL2-RC led to the π-pathway and a higher barrier (23.0) for methyl HAT in the former and the σ-pathway and a lower barrier (17.5) for ethyl Cα HAT in the latter. We analyzed the **Lys­(Me/Eth)** MD using a Kernel density estimate (KDE) plot of the O–C distance versus ∠Fe–O–C angle. Based on KDE analysis, the methyl carbon spans a wide range of ∠Fe–O–C angles from 90°–170°, suggesting that both σ and π pathways are feasible, while the ethyl carbons (Cα and Cβ) access only a narrow range from 120°–130°, which predominantly prefers the σ-pathway (Figure S25). We further employed energy decomposition analysis (EDA) to elucidate the energetic contributions of SCS and LR residues to HAT and rebound reactivity in MEL1-RC and MEL2-RC. The analysis predicted that N344, Q237, and Y239 were involved in destabilizing HAT TS during the hydroxylation of the methyl group in MEL1-RC, while N191, R199, and D291 were involved in stabilizing HAT TS. Of these, only D291 was observed in the TS stabilization of HAT in the **Lys­(Me**_**3**_ **)** substrate.^42^ For the case of ethyl Cβ oxidation in MEL1-RC, N191, G232, and Q237 were predicted to be involved in stabilizing the HAT TS, while T190, Y239, and W343 were involved in destabilizing it (Figure S26). D291 was found to be unique for the methyl oxidation in MEL1-RC, while Q237 was found to be unique in ethyl oxidation. Furthermore, S258, and D291 were predicted to be involved in the HAT TS stabilization of oxidation of both Cα and Cβ carbons of the ethyl group in MEL2-RC (Figure S27). K241 was predicted to destabilize MEL2-TS1-eth-Cα, while the same was suggested to stabilize in the case of MEL2-TS1-eth-Cβ. Thus, we suggest that the K241 residues might be crucial in the regioselectivity of hydroxylation in the case of the ethyl group in MEL2-RC. Based on DCCA analysis of the MD trajectory of **Lys­(Me/Eth)** dynamics, the residues N191 and K241 were found to be correlated with the DSBH fold while showing anticorrelated motion with the Zn-binding domain of the KDM6B protein, including regions (i) and (ii) discussed in the MD section (Figure 2b). Thus, QM/MM simulations suggest that the motions observed in the MD were crucial in the catalysis of hydroxylation involved in the KDM6B enzyme with the **Lys­(Me/Eth)** substrate.

##### 3.2.3.2 Isopropyl Lysine (Lys­(iPr))

Similarly, we analyzed the electronic structure of TSs of the HAT involved in the Cα and Cβ carbons of the isopropyl group in IL1-RC. Based on SNO analysis, the Cα HAT proceeds through the σ-pathway with the electron transfer from the σ_C–H_ bond to the σ*_z_2 orbital of the Fe center, leaving a radical on the Cα carbon (Figure S28). However, the Cβ HAT proceeds through a π-pathway with the electron transfer from the σ_C–H_ bond to the π*_yz_ of the Fe center. Spin densities of −0.96 and 1.07 on the Cα and Cβ carbons in IL1-IM1-Cα and IL1-IM1-Cβ, respectively, corroborate the electron transfer pathways predicted in SNO analyses (Figure S29). The FMO energy difference between σ*_z_2 and π*_xz_ was calculated to be 0.441 eV (10.1 kcal/mol) in IL1-RC (Figure S30). Due to this energy difference, we observed a lower barrier for Cα HAT, which follows the σ-pathway, while the Cβ HAT required a higher barrier as it follows the π-pathway. KDE analysis of the MD trajectory of **Lys­(iPr)** also shows that the Cα carbon of **Lys­(iPr)** accesses conformations with ∠Fe–O–Cα of 120°–140°, consistent with the σ-pathway, while the Cβ carbon accesses conformations with ∠Fe–O–Cβ of 90°–110°, consistent with the π-pathway (Figure S31). EDA analysis on the oxidations of the Cα carbon of the isopropyl group predicted that N191, K241, and N253 are involved in HAT TS stabilization (Figure S32). However, for Cβ HAT TS, E104, D193, and S258 are involved in stabilization. Among these residues, K241 and S258 are involved in TS stabilization of the ethyl oxidation of **Lys­(Me/Eth)** substrate, while only N191 is observed in TS stabilization of methyl oxidation of **Lys­(Me/Eth)**. All the residues that surround the **Lys­(iPr)** substrate are predicted to be correlated with the H3 substrate residues and anticorrelated with the Zn-binding domain residues based on DCCA analysis on the **Lys­(iPr)** substrate MD simulations (Figure 2d).

In summary, our computational studies reveal that substrate-dependent electronic and conformational features distinctly influence the HAT mechanism and regioselectivity in KDM6B catalysis. The ∠Fe–O–C geometries and specific SCS/LR residue interactions govern the reaction outcome.

### 3.3 How Do Conformational Dynamics Orient Hydroxylated Alkyl Substrates at Fe­(IV)=O Intermediate State of KDM6B?

To understand the substrate dynamics at the hydroxylated states of ethyl and isopropyl, respectively, of the **Lys­(Me/Eth)** and **Lys­(iPr)** substrates with the KDM6B–Fe­(IV)=O intermediate, we implemented MD simulations on KDM6B–Fe­(IV)=O·Lys­(Me/Eth–OH) and KDM6B–Fe­(IV)=O·Lys­(iPr–OH) to obtain equilibrated trajectories (Figures S33, S34). In contrast to **Lys­(Me/Eth)** and **Lys­(iPr)** dynamics, the hydroxylated ethyl and isopropyl groups were consistently closer to the Fe­(IV)=O species based on the histogram analysis, with the Cβ of both ethyl and isopropyl invariably closer to the iron center (Figures S35, S36). The hydroxyl group of Y239 showed interactions with the amino group of Lys­(Me/Eth–OH) when the hydroxylated ethyl group was closer to the Fe­(IV)=O species. The methyl group accessed the Fe­(IV)=O species the least in the **Lys­(Me/Eth–OH)** trajectory. The SCS residue N344, which formed a stable hydrogen bonding interaction with the amino group of **Lys­(Me/Eth)**, showed limited interactions with the amino group of **Lys­(Me/Eth–OH).** The loss of this critical SCS interaction might hinder the approach of methyl to the Fe­(IV)=O species. On the contrary, the amino group of **Lys­(iPr–OH)** showed stable hydrogen bonds with N344 when the hydroxylated Cβ is closer to the Fe center. Further atomistic analysis revealed that the Fe-binding residues, H250 and H330, maintained stable hydrogen bonding interactions with each other in both systems. The backbone of E252 exhibited hydrogen bonding interactions with N255, while the carboxylate groups of succinate formed stable hydrogen bonding interactions with T247, N260, and N340. The simulations revealed that substrate binding and orientation were influenced by critical interactions with SCS residues, particularly N344 and Y239, similar to the other substrates explored in this study.

We further explored the dominant motions of KDM6B in the presence of **Lys­(Me/Eth–OH)** substrate using PCA. The analysis revealed that the regions (i), (ii), and (iii) are flexible in the **Lys­(Me/Eth–OH)** and **Lys­(iPr–OH)**; however, they are less flexible than the **Lys­(Me/Eth)** and **Lys­(iPr)** system (Figure 8). Contrary to the **Lys­(Me/Eth) and Lys­(iPr)** system, the **Lys­(Me/Eth–OH)** and **Lys­(iPr–OH)** systems showed more flexibility in the disordered region (152–184). This motion might be helpful in the favorable binding of the H3 substrate with **Lys­(Me/Eth–OH)** and **Lys­(iPr–OH)** modification. The change in flexibility of the region (iii) was also reflected in the correlated/anticorrelated motions of the KDM6B protein in both systems. DCCA analysis showed that the region (iii) was highly anticorrelated with the DSBH fold in both systems (Figure 8). However, the magnitude and extent of correlation/anticorrelation were higher in **Lys­(Me/Eth–OH)** compared to **Lys­(iPr–OH)**.

**Figure 8:**
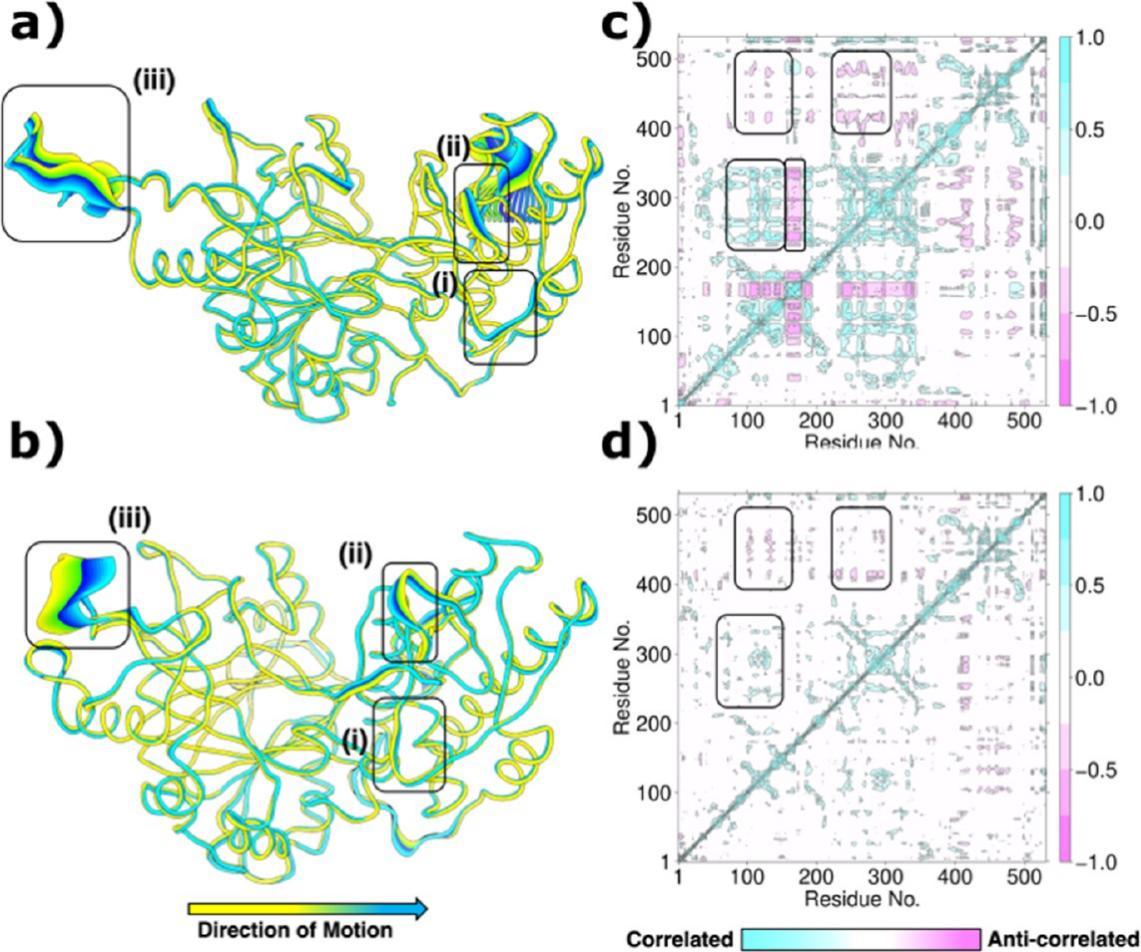
Conformational behavior of KDM6B–Fe­(IV)=O species with **Lys­(Me/Eth–OH)**, and **Lys­(iPr–OH)**. PCA showing the dominant motions of the KDM6B enzyme with (a) **Lys­(Me/Eth–OH)** and (b) **Lys­(iPr–OH)** substrates. DCCA plot showing the correlated/anticorrelated motions involved in the KDM6B with (c) **Lys­(Me/Eth–OH)** and (d) **Lys­(iPr–OH)** substrates. Boxed areas show correlated and anticorrelated motions of the flexible regions.

Overall, the conformational analysis of the **Lys­(Me/Eth–OH)** and **Lys­(iPr–OH)** substrate systems revealed intriguing motions in different regions of the KDM6B enzyme, which could be modulated to alter activity.

### 3.4 Mechanism of Subsequent Oxidation of Hydroxylated Alkyl Substrates in KDM6B

Experimental studies have shown that KDM6B can catalyze the oxidation of **Lys­(Me/Eth–OH)** and **Lys­(iPr–OH)** to form aldehyde derivatives of the substrates. To understand the catalytic mechanism of this oxidation, we employed QM/MM reaction path calculations on the snapshots obtained from different portions of the equilibrated MD trajectory, considering the various orientations of the substrate. Three principal mechanistic pathways were examined in the reaction path calculations: (1) HAT from the hydroxo group to generate an Fe­(III)–OH species, followed by a second HAT from the Cβ carbon of the substrate, or the reverse sequence; (2) the corresponding HAT steps proceeding via water mediation; and (3) HAT from the Cβ carbon, followed by hydroxyl rebound at Cβ to form a gem-diol intermediate,^112^ which subsequently undergoes dehydration to yield the aldehyde product.

#### 3.4.1 Mechanism of Oxidation of Lys­(Me/Eth–OH) in KDM6B

We obtained a snapshot from the MD trajectory of KDM6B–Fe­(IV)=O•Lys­(Me/Eth–OH) and optimized it using QM/MM to obtain MEL–OH-RC. First, we explored HAT from the hydroxo group using a PES scan, which gave a barrier of 20.9 kcal mol^–1^ (Figure 9a).

**Figure 9:**
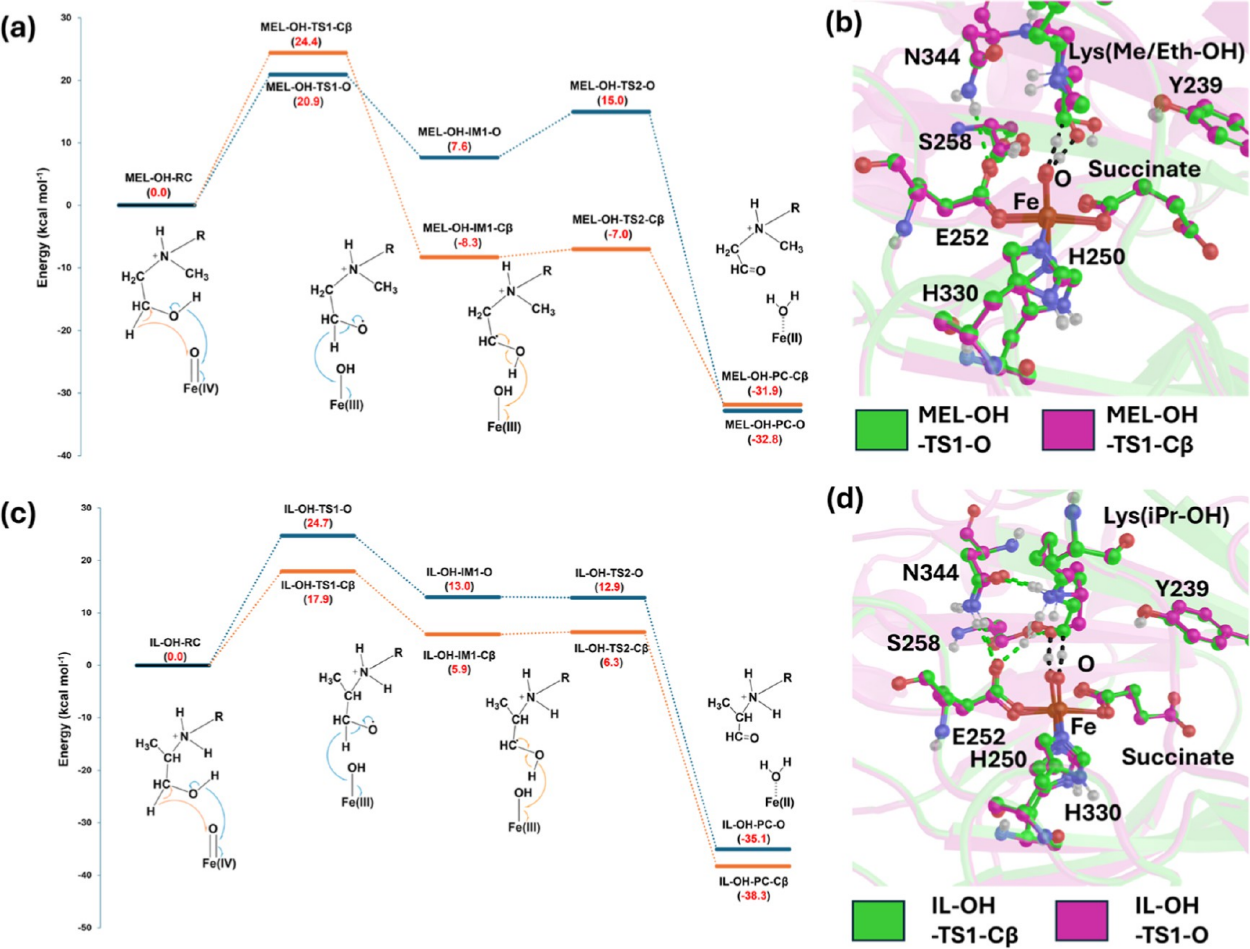
(a) Reaction Profile of the oxidation of **Lys­(Me/Eth–OH)** (b) Overlaid TSs obtained during O-HAT and ethyl Cβ HAT in **Lys­(Me/Eth–OH)**, (c) reaction profile of oxidation of **Lys­(iPr–OH)**, and (d) Overlaid TSs obtained during O-HAT and Cβ-HAT in **Lys­(iPr–OH)**. Relative energies are presented at the QM­(B3)/MM level. Hydrogen bonds are represented in green dashed lines. TS partial bonds are represented in black dashed lines.

The reaction led to the formation of Fe­(III)–OH group with a radical on the oxygen atom of the substrate at MEL–OH-IM1 through MEL–OH-TS1. At the MEL–OH-RC state, the distance between the ferryl oxygen (O) and the hydrogen of the hydroxo group (H_o_) was 4.36 Å, and the O–H2 (hydrogen of the Cβ of ethyl) was 4.18 Å (Figure 10). While at MEL–OH-TS1, the O–H_oh_ and O_oh_-H_oh_ distances were 1.13 Å and 1.26 Å, with a ∠Fe–O–H_oh_ of 116.4°. The distance between O and H2 at MEL–OH-IM1 was 2.71 Å, with ∠Fe–O–H2 of 102.4 Å (Figure 10). Further, the second HAT from the Cβ by the Fe­(III)–OH group required a 7.3 kcal/mol barrier, which leads to the formation of an aldehyde group at the Lys­(Me/Eth–OH) substrate, with a water molecule formed and bound to the Fe center (MEL–OH-PC).

**Figure 10:**
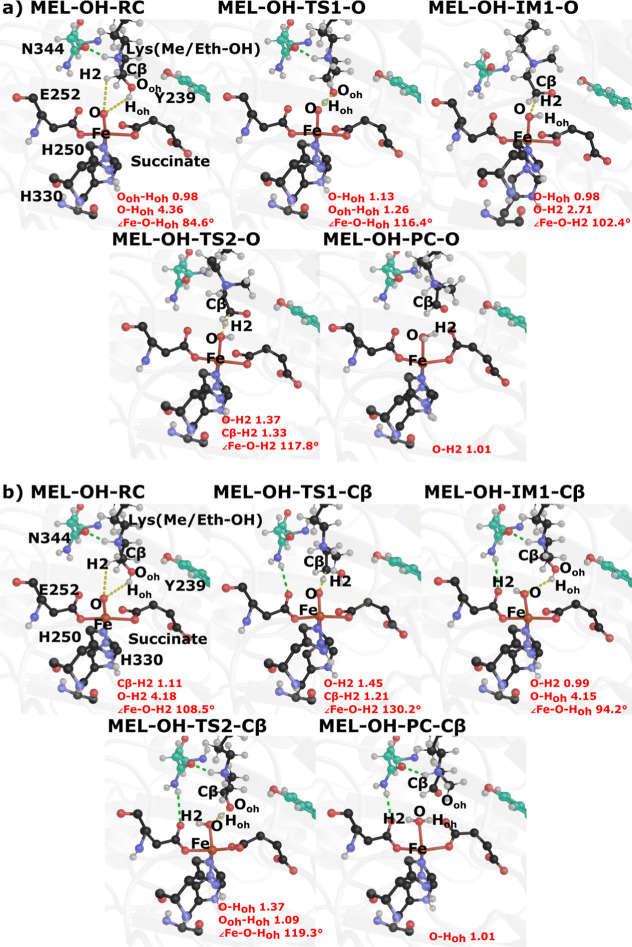
QM/MM optimized structures for the stationary points obtained from the MEL–OH-RC snapshot initiating with (a) O-HAT and (b) Cβ–HAT. Distances are in Å and represented by yellow dashed lines. Hydrogen bonding interactions: green dashed lines.

We also explored the initiation of oxidation through HAT from the Cβ carbon of the Eth–OH, as the MD simulations of **Lys­(Me/Eth–OH)** suggested that the Cβ carbon was consistently closer to the Fe­(IV)=O species. However, the favorable conformation of the Cβ carbon does not influence the reactivity, as the Cβ HAT proceeded with a high barrier of 24.4 kcal mol^–1^ (Figure 9a). Although the activation barrier was higher, the intermediate MEL–OH-IM1-Cβ thus formed was much more stabilized with −8.3 kcal mol^–1^ compared to the 7.6 kcal mol^–1^ energy of MEL–OH-IM1-O (Figure 9a). The subsequent second HAT from the O_oh_-H_oh_ group proceeded with a low barrier of 1.3 kcal mol^–1^ to give the aldehyde product (MEL–OH-PC-Cβ), which is almost isoenergetic with MEL–OH-PC-O. Although Cβ HAT, followed by O_oh_-H_oh_ HAT, was a high-energy pathway, the intermediate MEL–OH-IM1-Cβ was more stabilized as it forms a secondary carbon radical. Hence, the initiation of oxidation by Cβ HAT cannot be ruled out completely. A computational study on hTET2 with 5-hydroxymethylcytosine (5hmC) investigated the oxidation of the hydroxo group by benzylic-type (Cβ) HAT, yielding a barrier of 18.7 kcal/mol to form 5-formylcytosine (5 fC).^56^ Another study investigated the O-HAT pathway for hTET2-mediated 5hmC oxidation, yielding a barrier of 20.1 kcal/mol.^57^ Therefore, based on our QM/MM reaction path calculations, we suggest that the **Lys­(Me/Eth–OH)** oxidation reaction proceeds through either of the two pathways proposed for hTET2 enzymes in the oxidation of 5hmC to 5fC.

Furthermore, from MEL–OH-RC, we further examined the possibility of water-mediated HAT for both pathways discussed above. Toward this, MEL–OH-RC was reoptimized with a water molecule near the Fe­(IV)-center in the QM region, where the water is surrounded by the substrate hydroxyl group, and the methyl carbon of the substrate’s ethyl substituent. In the optimized MEL–OH-RC_wat_, the distance between ferryl oxygen and Ow (oxygen of the water molecule) was 2.80 Å. A PES scan for water-mediated HAT from the hydroxyl group gave a barrier of 23.3 kcal/mol (Figure S37), proceeding through MEL–OH-TS1-O_wat_ a Fe­(III)–OH species with a radical centered on the substrate oxygen atom (Figures S37 and 38). The resulting intermediates from direct HAT and water-mediated HAT are very similar in relative energy (7.6 and 7.9 kcal/mol, respectively), and the corresponding barriers are also comparable, indicating that both pathways are favorable, although direct HAT is slightly preferred. From MEL–OH-IM1-O_wat_, we then modeled water-mediated C–H HAT; however, the reaction proceeds with a very high-energy activation energy (MEL–OH-TS2-O_wat_, 40.1 kcal/mol) to give the aldehyde product MEL–OH-PC-O_wat_ (Figures S37 and S38). For the alternative pathway in which substrate oxidation begins with C–H HAT, the initial water-mediated HAT step also showed a very high barrier of 40.5 kcal/mol via MEL–OH-TS1-Cβ_wat_, making this pathway implausible (Figures S37 and S38). However, the subsequent O–H HAT from MEL–OH-IM1-Cβ_wat_ to MEL–OH-PC-Cβ_wat_ occurs almost barrierless in the water-mediated pathway (0.4 kcal/mol) and is slightly lower than the corresponding direct HAT barrier (1.3 kcal/mol) (Figures 9, S37, and S38). These results indicate that while water-mediated HAT is not viable for pathways initiated by C–H abstraction, the conversion from MEL–OH-IM1-Cβ to MEL–OH-PC-Cβ can proceed through either a direct or a water-mediated HAT mechanism.

Additionally, we explored the gem diol pathway from MEL–OH-IM1-Cβ (Figures S39, S40). The OH rebound step was found to proceed with an activation barrier of 12.7 kcal/mol, leading to a gem-diol intermediate that is 25.6 kcal/mol exothermic (Figure S40). We also investigated the subsequent dehydration of the gem-diol intermediate by considering two possible proton-transfer arrangements in which one hydroxyl group donates a proton to the other. Both dehydration pathways were found to be highly unfavorable, with activation barriers of 42.6 and 32.3 kcal/mol, respectively (Figure S40). These large barriers indicate that the gem-diol dehydration route is not a viable pathway for aldehyde formation from **Lys­(Me/Eth)** substrate. In contrast, the second direct HAT pathway from MEL–OH-IM1-Cβ proceeds with a substantially lower barrier of only 1.3 kcal/mol (Figure 9a) and leads directly to the aldehyde product, which is 31.9 kcal/mol exothermic. Thus, both kinetically and thermodynamically, the direct second-HAT pathway is more favorable than the gem-diol route.

Overall, the mechanistic analysis indicates that although some elementary steps in the alternative routes involving water-mediated pathways or gem-diol formation to form the aldehyde product are energetically accessible, the direct HAT pathway for aldehyde formation remains the preferred pathway in most cases. Water-mediated HAT and direct HAT are close in terms of activation energy during the initial O–H abstraction from MEL–OH-RC, however, it becomes strongly disfavored in pathways that begin with C–H abstraction. Likewise, OH rebound from MEL–OH-IM1-Cβ may produce a gem-diol intermediate, but the following dehydration step faces prohibitively large activation barriers, making this pathway an implausible source of aldehyde formation. In contrast, the direct/water-mediated second-HAT pathways from MEL–OH-IM1-Cβ or MEL–OH-IM1-Cβ_wat_ proceed with a very low activation barrier, making them competing pathways.

#### 3.4.2 Mechanism of Oxidation of Lys­(iPr–OH) in KDM6B

Further to understand the oxidation of the hydroxy group of Lys­(iPr–OH), we obtained a snapshot from the KDM6B–Fe­(IV)=O·Lys­(iPr–OH) MD trajectory and implemented QM/MM reaction path calculations on it. At IL–OH-RC, the distance of the oxygen of Fe­(IV)=O species (O) from the hydrogen of the hydroxo group (H_o_) and the hydrogen of the Cβ (H2) were 3.25 and 2.16, respectively (Figure 11). The initial HAT of the H_oh_ of the Lys­(iPr–OH) required a barrier of 24.1 kcal mol^–1^ to give IL–OH-IM, where the O–H2 distance became 1.85 Å (Figure 9c). The higher activation energy may be attributed to the hydrogen bond between the OH group and E252 (Figure 11). The second HAT spontaneously converts the alcohol to an aldehyde group on the Lys­(iPr–OH) substrate (IL–OH-PC) with an energy of −35.1 kcal mol^–1^. Similar to MEL–OH-RC, we also explored an alternative mechanism involving the initiation of oxidation from the Cβ HAT. Compared to MEL–OH-RC, the reaction proceeded with a lower barrier of 17.9 kcal mol^–1^, leading to an endothermic IL–OH-IM1-Cβ intermediate, with 5.9 kcal mol^–1^ energy (Figure 9c). The barriers are consistent with barriers obtained for hTET2-mediated oxidations of 5hmC to 5fC.^56^ At IL–OH-TS1-Cβ, the O–H2 distance was 1.22 Å, and the Cβ-H2 distance increased to 1.33 Å (Figure 11). Subsequent HAT from the O_oh_-H_oh_ group proceeded with the negligible barrier of 0.4 kcal/mol to give IL–OH-PC-Cβ, which was exothermic with 3.2 kcal mol^–1^ less energy than IL–OH-PC-O. The N^ε^-H groups of **Lys­(iPr–OH)** formed a hydrogen bond with N344 during the secondary oxidation (Figure 9d). Hence, in IL–OH-RC, the second pathway that initiates with Cβ HAT was more favorable than the one that initiates with O_oh_-H_oh_ HAT.

**Figure 11:**
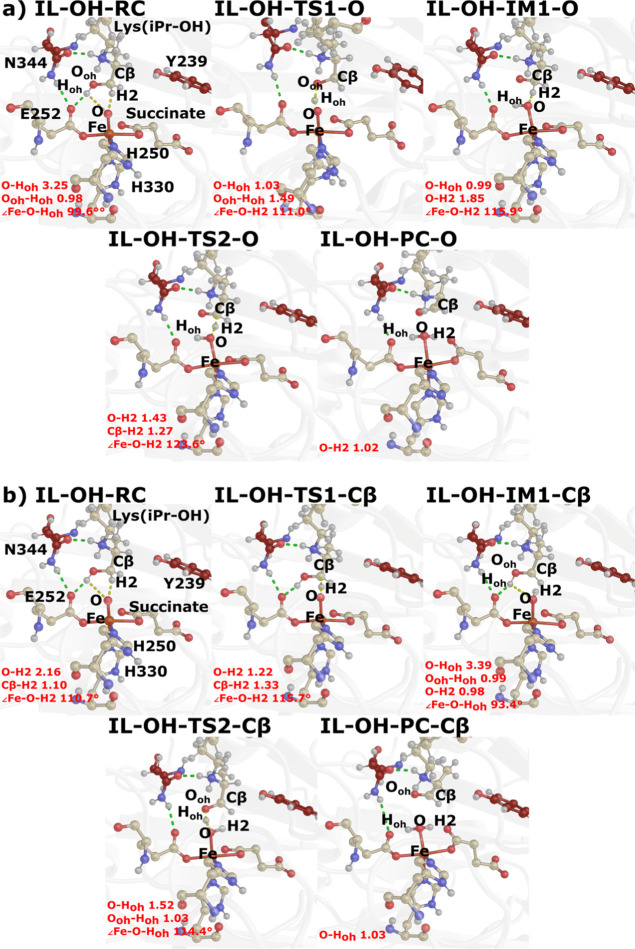
QM/MM optimized structures for the stationary points obtained from the IL–OH-RC snapshot initiating with (a) O-HAT and (b) Cβ–HAT. Distances are in Å and represented by yellow dashed lines. Hydrogen bonding interactions are represented in green dashed lines.

Additionally, a water molecule located near the Fe­(IV) center was included in the QM region of IL–OH-RC and was optimized to generate IL–OH-RC_wat_ to assess water-mediated HAT pathways for the two oxidation routes discussed above. In IL–OH-RC_wat_, the distance between the ferryl oxygen and Ow was 2.79 Å. PES scans for substrate oxidation via water-mediated H_oh_ HAT or water-mediated Cβ HAT showed high activation barriers of 74.0 kcal/mol (B1) and 72.0 kcal/mol (B1), respectively, suggesting that these pathways are unfavorable (Figure S41). Additionally, we examined the second oxidation step via water-mediated HAT from IL–OH-IM1-O and IL–OH-IM1-Cβ by including a water molecule in the QM region, yielding IL–OH-IM1-O_wat_ and IL–OH-IM1-Cβ_wat_. In IL–OH-IM1-O_wat_, water-mediated HAT had a barrier of 10.1 kcal/mol, higher than the direct HAT barrier of 0.1 kcal/mol (Figures 9c and S68). In the case of IL–OH-IM1-Cβ_wat_, the PES scan resulted in a spontaneous transfer of the H_oh_ from the substrate hydroxyl to the nonbonded oxygen of iron-binding E252 at 14.9 kcal/mol from IL–OH-IM1-Cβ_wat_, yielding an aldehyde on the substrate along with a Fe­(II)–OH species (IL–OH-IM2-Cβ_wat_), which was ∼ 23.2 kcal/mol exothermic relative to IL–OH-IM1-Cβ_wat_ (Figure S69). Continuing the PES scan, the proton on E252 was transferred back to the iron-coordinating hydroxyl group, without any energy barrier from IL–OH-IM2-Cβ_wat,_ restoring it as water and completing the reaction. However, the barrier observed for this pathway (14.4 kcal/mol) is also much higher than the direct HAT barrier (0.4 kcal/mol), and thus, this pathway is unfavorable.

Furthermore, we explored the gemdiol pathway from IL–OH-IM1-Cβ, and the rebound scan revealed somewhat different behavior from its MEL counterpart. During the PES scan of the OH rebound process, the proton from the substrate hydroxyl group was found to transfer spontaneously to the iron-coordinating glutamate, forming a relatively stable aldehyde. This proton-transfer step occurs with a barrier of 2.2 kcal/mol and gives an aldehydic intermediate that is 32.4 kcal/mol lower in energy than IL–OH-IM1-Cβ. Continuing the scan along the rebound coordinate led to a rebound transition state with a barrier of 4.1 kcal/mol relative to this aldehydic intermediate, ultimately forming a gem-diol species at 45.9 kcal/mol below IL–OH-IM1-Cβ. We then examined the dehydration of this gem-diol intermediate through two possible pathways to regenerate the aldehydic product. However, both routes were associated with very high activation barriers of 51.84 kcal/mol (B1) and 41.98 kcal/mol (B1), respectively, making them energetically inaccessible. By comparison, the second HAT pathway from IL–OH-IM1-Cβ has a barrier of only 0.4 kcal/mol, indicating that this route is more favorable.

Taken together, these results indicate that although gem-diol formation via OH rebound is energetically accessible in some cases, the subsequent dehydration step is prohibitively unfavorable. Therefore, the direct pathway involving the second C–H activation/HAT step remains the preferred mechanism for aldehyde formation in both substrate systems.

#### 3.4.3 Electronic and Conformational Features Responsible for Oxidation of Lys­(Me/Eth–OH) and Lys­(iPr–OH)

To understand the electronic and conformational features responsible for oxidation of the alcohols to the aldehydes, we implemented SNO, EDA, DCCA, and KDE analyses on Lys­(Me/Eth–OH) and Lys­(iPr–OH) systems.

##### 3.4.3.1 Hydroxy Ethyl Methyl Lysine (Lys­(Me/Eth–OH))

The SNO analysis of the MEL–OH-TS1-O and MEL–OH-TS1-Cβ predicted that an alternate π-pathway was preferred in both cases, where an electron from σ_CH_ transfers to π*_yz_, since the ∠Fe–O–H angle is less than 120° in MEL–OH-TS1-O (Figure S42). The spin density analysis showed a negative spin density of −0.87 at O_oh_ at MEL–OH-IM1-O and delocalized negative spin density along Cβ(−0.80)-O_oh_(−0.18) at MEL–OH-IM1-Cβ (Figure S43). Hence, we suggest that the electron transfer during HAT of secondary oxidation involves an alternative π-pathway, which occurs at the quintet spin state of Fe. FMO analysis revealed that the energy split between σ*_z_2 and π*_xz_ was less, with 0.363 eV (8.3 kcal/mol) in MEL–OH-RC, compared to KDM6B with **Lys­(Me/Eth)** and **Lys­(Me**_**3**_ **)** (Figure S44). Hence, despite taking the π-pathway, the activation barriers were lower with 20.9 kcal/mol. KDE analysis of ∠Fe–O-X versus O-X distance (X denotes HAT targets) in the MD trajectory of **Lys­(Me/Eth–OH)** substrate dynamics shows that at HAT favorable distances, the ∠Fe–O–Cβ and ∠Fe–O–O_oh_ were less than 120° through most of the trajectory (Figure S45). Hence, the MD simulations suggest that the HAT reaction can proceed through the π-pathway based on the substrate conformational dynamics observed through KDE analysis. EDA analysis predicted that the TS stabilizing residues for O-HAT include E104, N253, N260, and K241, while for Cβ-HAT, the residues D291, N253, and W343 are involved in TS stabilization (Figure S46). D291 was consistently observed in TS stabilization of Cβ-HAT in both **Lys­(Me/Eth)** and **Lys­(Me/Eth–OH)** substrate oxidations, as it was observed in **Lys­(Me**_**3**_ **)** hydroxylation. TS stabilizing residues in **Lys­(Me/Eth–OH)** are involved in the SCS interactions with the active site residues. Out of these residues, N260 was found to be unique and contribute more energetically to the TS stabilization of O-HAT in **Lys­(Me/Eth–OH)**, which forms hydrogen bonding interactions with the C4 carboxylate of succinate. All TS stabilizing residues are involved in correlated motions with the DSBH fold and anticorrelated motions with the Zn­(II)-binding domain (Figure 8c). Interestingly, they also show anticorrelated motion with the disordered region of the KDM6B protein. Hence, the disordered region may play a crucial role in the subsequent oxidation of the **Lys­(Me/Eth–OH)** substrate by the KDM6B protein.

##### 3.4.3.2 Hydroxy Propan-2-yl Lysine (Lys­(iPr–OH))

The SNO analysis of both the IL–OH-TS1-O and IL–OH-TS1-Cβ revealed that O_oh_-H_oh_ HAT proceeded through the σ-pathway while Cβ HAT proceeded through the π-pathway (Figure S47). The spin density analysis also corroborated the SNO analysis with −0.77 on O_oh_ in IL–OH-IM1-O, while the spin density of the substrate was delocalized along Cβ-O_oh_ with 0.80 on Cβ and 0.14 on O_oh_ (Figure S48). Similar to MEL–OH-RC, the FMO analysis revealed that the energy split between σ*_z_2 and π*_xz_ was 0.371 eV (8.5 kcal/mol) in the IL–OH-RC (Figure S49). Hence, despite following the π-pathway, the Cβ HAT required a lower barrier of 17.9 kcal/mol. KDE analysis also revealed that the ∠Fe–O–O_oh_ was within 80°–150° when the O–O_oh_ was within the favorable HAT distance, whereas the ∠Fe–O–Cβ was narrower between 90° and 130° when the O–Cβ was within the favorable HAT distance. Hence, the KDE analysis revealed that the substrate disposition in MD dynamics of **Lys­(iPr–OH)** shows both σ- and π-pathways are feasible for O_oh_-H_oh_ HAT and Cβ HAT (Figure S50). Interestingly, based on EDA analysis, the residues N344 and S258 have been involved in high magnitude energy stabilizations of both O_oh_-H_oh_ and Cβ HAT TS states (Figure S51). Importantly, S258 forms hydrogen bonds with the O_oh_ atom of the Lys­(iPr–OH) substrate; hence, it contributes toward the oxidation reaction of the **Lys­(iPr–OH)** substrate. Interestingly, both residues are correlated with the DSBH fold but without any anticorrelated motion with the Zn­(II)-binding domain, as found consistently in other oxidation reactions. Further, they show correlated motions with the H3 peptide chains, which were not observed in other oxidation reactions discussed.

### 3.5 How Do Conformational Dynamics Orient Alkanal Substrates at Fe­(IV)=O Intermediate State of KDM6B?

Similar to hydroxylated alkyl substrates, we implemented MD simulations to ascertain the substrate dynamics of aldehyde variants of **Lys­(Me/Eth)** and **Lys­(iPr)** substrates **(Lys­(Me/Eth-AL)** and **Lys­(iPr-AL)** bound to the Fe­(IV)=O complexes of KDM6B (Figures S52, S53). The substrate dynamic analysis revealed that the aldehydic hydrogen was closer to the reactive Fe­(IV)=O species in most of the 1 μs MD trajectory of both systems. The histogram analysis showed that the aldehyde hydrogen and Cβ carbon were consistently closer to the Fe­(IV)=O species (Figure S54), followed by the methyl group, similar to **Lys­(Me/Eth–OH)** and conversely to the **Lys­(Me/Eth)** dynamics, where the methyl group was consistently closer to the Fe­(IV)=O species. Similarly, in **Lys­(iPr-AL)**, the histogram analysis revealed that the aldehydic hydrogen was consistently closer to the Fe­(IV)=O species throughout the trajectory (Figure S55). The hydrogen bonding analysis of the active site revealed that when the aldehyde group was closer to Fe­(IV)=O species in **Lys­(Me/Eth-AL)** system, the amino group of **Lys­(Me/Eth-AL)** substrate forms hydrogen bond with the hydroxo group of Y239, however when the methyl group was closer to Fe­(IV)=O species, the amino group forms hydrogen bond with N344. Conversely, in **Lys­(iPr-AL)**, the amino group forms a hydrogen bond with N344 consistently throughout the simulation. Additionally, we also evaluated the MM/GBSA binding free energies of the alkyl, alcohol, and aldehyde intermediates derived from the **Lys­(Me/Eth)** and **Lys­(iPr)** substrates at the ferryl stage (Table S2). The results indicate that alcohol and aldehyde intermediates exhibit stronger binding affinities than their parent substrates, supporting a processive mechanism. Nonetheless, more rigorous enhanced sampling approaches, such as QM/MM metadynamics or umbrella sampling, are necessary to reach a definitive conclusion.

We used PCA to analyze the dominant motions of the KDM6B enzyme in the presence of **Lys­(Me/Eth-AL)** and **Lys­(iPr-AL)** substrate. The analysis showed that the regions (i), (ii), and (iii) had the most flexibility in **Lys­(Me/Eth-AL)** and **Lys­(iPr-AL)** dynamics (Figure 12a,b). However, the magnitude of flexibility in **Lys­(Me/Eth-AL)** dynamics was limited in regions (i) and (ii) compared to **Lys­(Me/Eth)** and **Lys­(Me/Eth–OH)**, while in **Lys­(iPr-AL)** dynamics, the magnitude of the flexibility was higher compared to **Lys­(iPr)** and **Lys­(iPr–OH)**. In **Lys­(Me/Eth-AL)**, the region (iii), containing the disordered loop, showed a different direction of motion toward the H3 substrate loop compared to **Lys­(Me/Eth)** and **Lys­(Me/Eth–OH)** systems, where the motion was directed away from the H3 substrate loop. On the contrary, the direction of motion of (iii) was toward the H3 substrate loop in the **Lys­(iPr-AL)** system, although with a higher magnitude of motion than the **Lys­(Me/Eth-AL), Lys­(iPr), and Lys­(iPr–OH)** systems. Additionally, the DCCA analysis of **Lys­(Me/Eth-AL)** and **Lys­(iPr-AL)** dynamics revealed anticorrelated motions between regions (i), (ii), and (iii) with the DSBH fold, which contains the active site (Figure 12). In contrast, the residues that form β3, β4, α8, and the loop connecting them (100–149) showed correlated motion with the DSBH fold in both systems. However, the magnitude of correlation/anticorrelation was higher in **Lys­(iPr-AL)** compared to **Lys­(Me/Eth-AL)**.

**Figure 12:**
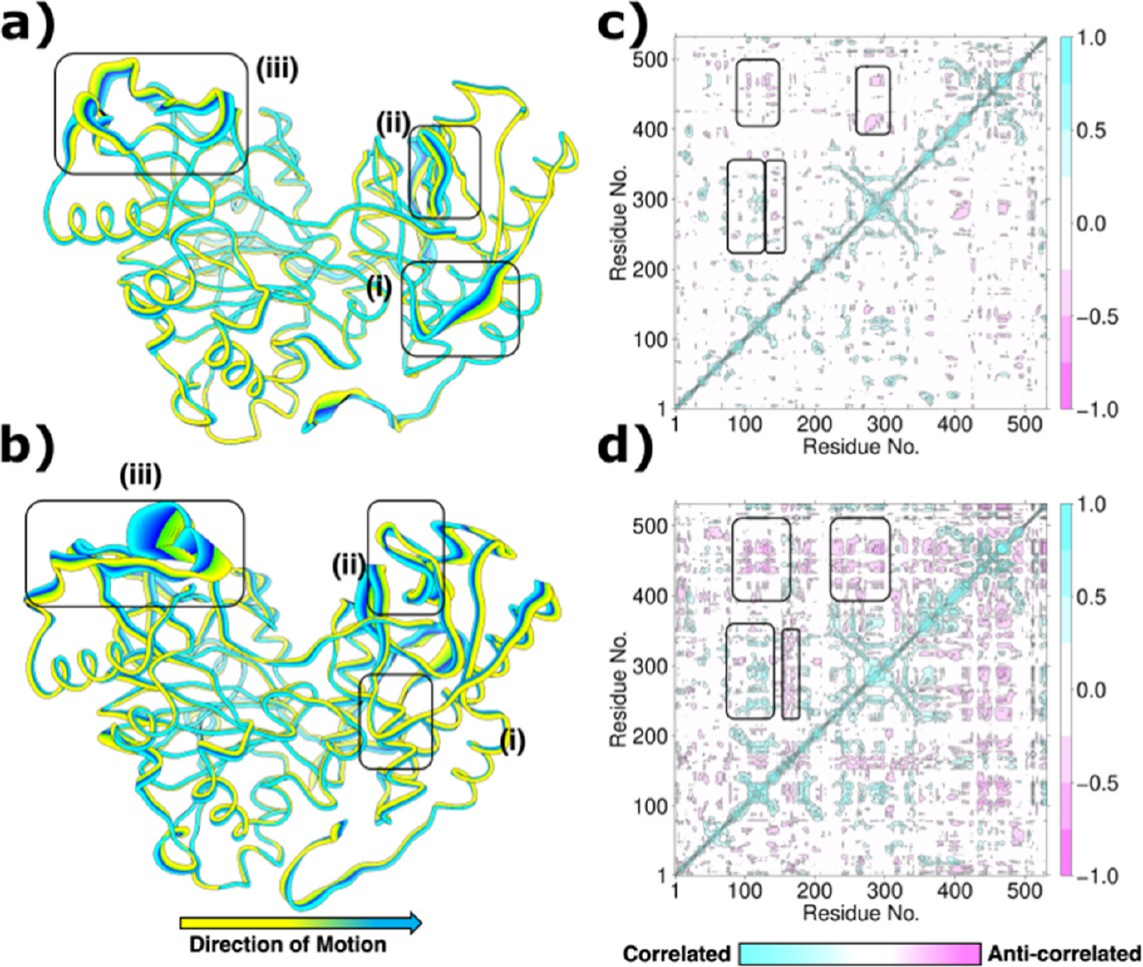
Conformational behavior of KDM6B–Fe­(IV)=O species with **Lys­(Me/Eth-AL)** and **Lys­(iPr-AL)**. PCA showing the dominant motions of the KDM6B enzyme with (a) **Lys­(Me/Eth-AL)** and (b) **Lys­(iPr-AL)** substrates. DCCA plot showing the correlated/anticorrelated motions involved in the KDM6B with (c) **Lys­(Me/Eth-AL)** and (d) **Lys­(iPr-AL)** substrates. Boxed areas show correlated and anticorrelated motions of the flexible regions.

Based on the conformational analysis of the aldehyde forms of the Lys substitutions, it was predicted that different regions of the KDM6B protein exhibit differential flexibilities and correlated motions with distinct H3 substrate modifications, suggesting that conformational dynamics influence the secondary oxidation of Lys substrates by the KDM6B enzyme.

### 3.6 Mechanism of Oxidation of Aldehyde-Substituted Substrates in KDM6B

KDM6B catalyzes the subsequent oxidation of aldehyde derivatives of the Lys27 residue of the H3 substrate. The following sections describe QM/MM reaction path calculations on the oxidation reaction catalyzed by KDM6B on the **Lys­(Me/Eth-AL)** and **Lys­(iPr-AL)** substrates. It also details the key intermediates and interactions that facilitate the conversion of the aldehydes to carboxylic acids.

#### 3.6.1 Mechanism of KDM6B Catalyzed Oxidation of Lys­(Me/Eth-AL)

To investigate the enzymatic influence of KDM6B on the catalytic conversion of aldehyde to carboxylic acid, we implemented QM/MM reaction path calculations on the most populated snapshot obtained from the MD trajectory of KDM6B–Fe­(IV)=O•Lys­(Me/Eth-AL). The obtained snapshot was optimized to obtain MEL-AL-RC, where the distance between ferryl oxygen (O) and the aldehydic hydrogen (H) was 3.08 Å and the ∠Fe–O–H was 103.4° (Figure 13). HAT from the aldehyde group to the Fe­(IV)=O center proceeded via a transition state (MEL-AL-TS1) with a calculated energy barrier of 17.0 kcal mol^–1^ (Figure 13). The barrier obtained for KDM6B-catalyzed oxidation of the aldehyde group to acid is much more favorable with **Lys­(Me/Eth-AL)** substrate compared to hTET2-catalyzed 5fC to 5-carboxylcytosine (5caC), which required a high barrier of 26.7 kcal mol^–1^, since the formyl group attached to cytosine has restricted rotation for HAT of formyl hydrogen.^56^ At this MEL-AL-TS1, the O–H bond length was 1.36 Å, the Fe–O–H angle was 126.7°, and the Cβ–H bond distance was 1.24 Å (Figure 13b). The resulting intermediate (MEL-AL-IM1) featured a shortened O–H distance of 0.98 Å, a Cβ-O distance of 3.43 Å, and a Fe–O–Cβ angle of 130.7°, with a carbon-centered radical at the aldehydic carbon (Figure 13). During the oxidation, S258 forms a hydrogen bond with the carbonyl oxygen of the aldehyde group of **Lys­(Me/Eth-AL)** substrate (Figure S56). The SNO analysis revealed that the HAT reaction proceeded through the σ-pathway with an α-electron transferred from σ_CH_ to the σ*z ^2^ orbital of Fe (Figure S57). The spin density analysis revealed that the β-electron occupancy on the substrate was delocalized along the C–O_s_ substrate with −0.66 on C and −0.24 on O_s_ at MEL-AL-IM1 (Figure S58). The FMO energy split between σ*_z_2 and π*_xz_ was 0.405 eV (9.3 kcal/mol) in MEL-AL-RC (Figure S59). KDE analysis distance­(O–H)-angle­(∠Fe–O–H) pair of the MD trajectory of **Lys­(Me/Eth-AL)** revealed that when the O–H distance was within a favorable HAT distance, the ∠Fe–O–H was within 90°–130° (Figure S60). Hence, the MD simulations show that the substrate disposition of **Lys­(Me/Eth-AL)** favors the σ-pathway for HAT from the aldehyde group. Subsequent hydroxyl rebound from the Fe­(III)–OH species to the radical center required an additional barrier of 12.6 kcal mol^–1^ (Figure 13) and proceeded through a second transition state (MEL-AL-TS2), where the Cβ-O distance was 2.29 Å and the Fe–O–Cβ angle had increased to 139.6° (Figure 13). The final product complex (MEL-AL-PC) corresponded to the carboxylic acid form of the substrate and was found to be thermodynamically favored by −50.6 kcal mol^–1^ relative to MEL-AL-RC (Figure 13). This stabilization was attributed to a hydrogen-bonding interaction between the carboxylic acid OH group and the nonbonded oxygen of residue E252 (Figure 13), underscoring the active site’s role in stabilizing the product state. EDA analysis of the reaction path of oxidation of **Lys­(Me/Eth-AL)** substrate revealed that residues E104, S258, and N260 were involved in HAT TS stabilizations (Figure S61). S258 forms hydrogen bonds with the carbonyl group of the **Lys­(Me/Eth-AL)** substrate, hence playing a crucial role in the consecutive oxidations of Lys as part of the H3 peptide chain.

**Figure 13:**
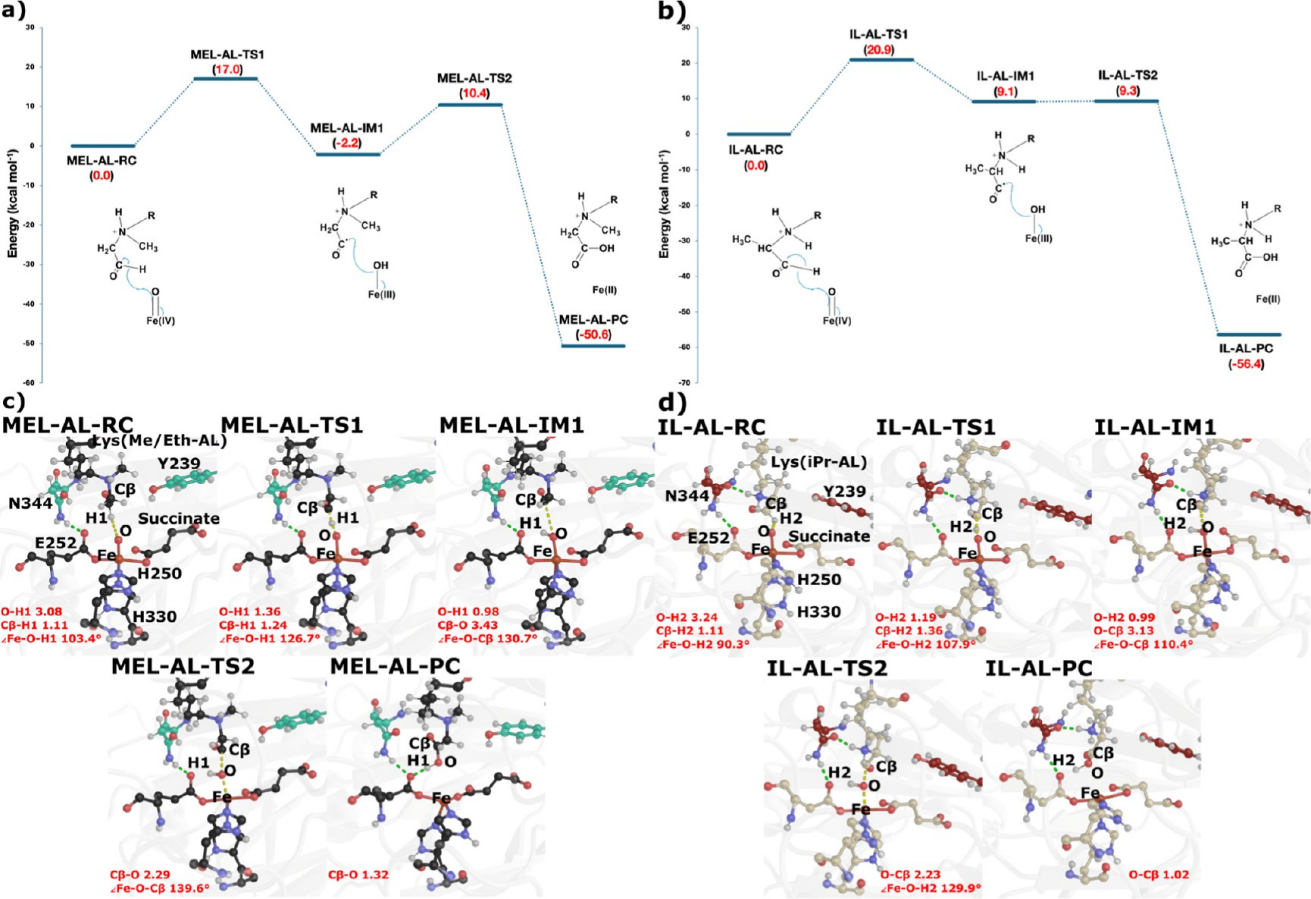
Reaction profile of oxidations catalyzed by KDM6B for (a) **Lys­(Me/Eth-AL)** and (b) **Lys­(iPr-AL)** substrates. QM/MM optimized structures for the stationary points obtained during oxidation from (c) MEL-AL-RC and (d) IL-AL-RC snapshots. Key distances are shown (Å).

#### 3.6.2 Mechanism of KDM6B-Catalyzed Oxidation of Lys­(iPr-AL)

The most populated conformation from the KDM6B–Fe­(IV)=O•Lys­(iPr-AL) MD trajectory was used for QM/MM studies to obtain IL-AL-RC. At the IL-AL-RC stage, the distance between the ferryl oxygen (O) and aldehydic hydrogen (H2) of **Lys­(iPr-AL)** substrate was 3.24 Å (O–H2), and ∠Fe–O–H2 was 90.3° (Figure 13). The reaction proceeds with a HAT from the aldehydic carbon through a transition state IL-AL-TS1 with a barrier of 20.9 kcal mol^–1^ to give Fe­(III)–OH and a carbonyl carbon-based radical (Cβ*) (IL-AL-IM1), 9.1 kcal mol^–1^ more energy than IL-AL-RC (Figure 13). At IL-AL-TS1, the O–H2 distance reduces to 1.19 Å and the Cβ-H2 distance increases to 1.36 Å with an increase in ∠Fe–O–H2 to 107.9° (Figure 13). Upon HAT, at IL-AL-IM1, the O–H2 distance reduces to 0.99 Å with an O–Cβ distance of 3.13 Å and ∠Fe–O–Cβ of 110.4° (Figure 13). Subsequently, the reaction proceeds with the rebound of the OH group to the Cβ radical to give the carboxylic acid derivatives of **Lys­(iPr)** substrate (IL-AL-PC) with −56.4 kcal mol^–1^ energy through the IL-AL-TS2 transition state, which was nearly isoenergetic with IL-AL-IM1, suggesting a near spontaneous rebound reaction (Figure 13). Throughout the reaction path, the hydrogen bonds of SCS residue N344 with the amino nitrogen of **Lys­(iPr-AL)** substrate and nonbonded oxygen of E252 were maintained, highlighting the importance of N344 in the consecutive oxidations catalyzed by KDM6B (Figure 13). Additionally, S258 forms hydrogen bonds with the carbonyl group, setting up the aldehyde for efficient oxidation (Figure S56). Overlaid TS states of HAT in **Lys­(Me/Eth-AL)** and **Lys­(iPr-AL)** showed differences in the SCS interactions, especially with respect to N344 (Figure S62). In contrast to the **Lys­(Me/Eth-AL)**, the **Lys­(iPr-AL)** HAT reaction proceeded through the π-pathway. Spin density analysis revealed that the substrate α-electron occupancy was delocalized among the C–O_s_ bonds with spin densities of 0.66 on C and 0.23 on the O_s_ atom at IL-AL-IM1 (Figure S63). The SNO analysis also revealed that the β-electron transfers from the σ_C–H_ bond to the π*_yz_ orbital of the Fe center (Figure S64). The FMO energy split between σ*_z_2 and π*_xz_ was 0.371 eV (8.5 kcal/mol) in IL-AL-RC (Figure S65). Thus, despite taking the π-pathway, the activation barrier for HAT was less (compared to **Lys­(Me/Eth-AL)** HAT) due to the lower energy split between acceptor orbitals. KDE analysis of the distribution of O–C and Fe–O–C pairs in the MD trajectory revealed that when O–C was within favorable HAT distances, the Fe–O–C was within 90°–110°, showing that the π-pathway was favorable for the HAT with **Lys­(iPr-AL)** (Figure S66). EDA analysis revealed that Q237, S258, and N344 are involved in stabilization of the transition states involved in oxidation of the aldehyde group in **Lys­(iPr-AL)** (Figure S67).

### 3.7 Comparison of Sequential Oxidation Mechanisms in hTET2 and KDM6B

hTET2 and KDM6B both operate through an iterative Fe­(IV)=O-mediated oxidation strategy in which a carbon-centered substituent is transformed stepwise through alcohol, aldehyde, and ultimately carboxylic acid derivatives. In hTET2, this is realized in the canonical 5mC → 5hmC → 5fC → 5caC pathway, where each stage proceeds through hydrogen abstraction chemistry followed either by hydroxyl rebound or by a second HAT/proton transfer, depending on the intermediate. Importantly, the second oxidation step in hTET2, namely 5hmC → 5fC, is not described by a single universally accepted HAT order. Rather, two distinct computational studies proposed different sequences: Lu et al.^56^ suggested that abstraction/proton transfer from the methylene moiety occurs first, followed by HAT from the hydroxyl group, whereas Torabifard et al.^57^ reported the opposite ordering, with hydroxyl HAT occurring first and the second hydrogen-transfer event following thereafter. Thus, in hTET2, the alcohol-to-aldehyde conversion appears to be highly sensitive to active-site geometry and second-sphere organization, such that different substrate orientations can support different stepwise oxidation sequences. A similar sequential oxidation manifold is observed in KDM6B for both **Lys­(Me/Eth)** and **Lys­(iPr)**, but here the substrate dependence is even more explicit. For **Lys­(Me/Eth)**, the initial β-carbon hydroxylation is relatively accessible, with HAT and rebound barriers of 19.6 and 15.5 kcal/mol, respectively. The second oxidation from alcohol to aldehyde can, in principle, proceed through two alternative HAT sequences, but the energetically preferred route involves O–H abstraction from the hydroxyl group followed by Cβ–H abstraction, with barriers of 20.9 and 15.0 kcal/mol, respectively. The final aldehyde-to-acid conversion then proceeds through HAT from the aldehydic carbon, followed by hydroxyl rebound, with barriers of 17.0 and 10.0 kcal/mol. In contrast, **Lys­(iPr)** exhibits a substantially more demanding first hydroxylation step at the β-carbon of the isopropyl substituent, with barriers of 26.6 kcal/mol for HAT and 27.4 kcal/mol for rebound, indicating that initial C–H activation is disfavored in **Lys­(iPr)**. Moreover, in the second oxidation step, **Lys­(iPr)** reverses the preferred order seen for **Lys­(Me/Eth)**, favoring C–H abstraction first followed by O–H abstraction, with corresponding barriers of 17.9 and 6.3 kcal/mol. The aldehyde-to-acid step again follows the same general mechanistic pattern as **Lys­(Me/Eth)**, with barriers of 20.9 kcal/mol for HAT and 9.3 kcal/mol for rebound. Taken together, the comparison shows that both hTET2 and KDM6B share a common mechanistic blueprint of sequential Fe­(IV)=O-oxidation, but both systems also exhibit nontrivial variability in the ordering of elementary hydrogen-transfer events during the alcohol-to-aldehyde step, likely due to differential SCS interactions and electrostatic environment. In hTET2, that variability arises from differences among computational models of the same substrate oxidation state,^56,57,111^ whereas in KDM6B it is manifested directly as substrate-controlled pathway selection between **Lys­(Me/Eth)** and **Lys­(iPr)**.

## 4 Conclusions

In this study, we employed a combination of classical MD simulations and hybrid QM/MM calculations to elucidate the mechanistic details of regioselective and sequential oxidations catalyzed by the nonheme Fe­(II)/2OG-dependent oxygenase KDM6B on synthetically modified lysine substrates. Our investigation focused on understanding how substrate binding, electronic structure, conformational dynamics, and SCS interactions contribute to HAT and subsequent oxidations in the catalytic cycle.

Our simulations on **Lys­(Me/Eth)** and **Lys­(iPr)** revealed distinct differences in substrate positioning and accessibility of alkyl groups to the reactive Fe­(IV)=O center. In the case of **Lys­(Me/Eth)**, the methyl group showed preferential access over the ethyl group, while in **Lys­(iPr)**, the isopropyl carbon (Cα) showed preferential access to the Fe­(IV)=O over the methyl groups (Cβ and Cβ′) attached to the isopropyl carbon. N344, E252, and Y239 determine the orientation of alkyl groups in the **Lys­(Me/Eth)** and **Lys­(iPr)** substrates.

KDE analyses of angle–distance distributions provided insight into the conformational space accessible to HAT targets. The σ-pathway was generally favored at higher ∠Fe–O–H angles, while lower angles allowed access to the π-pathway. These geometric preferences were reflected in the QM/MM-optimized TS structures and supported by SNO and spin density analyses. FMO analysis of the acceptor orbitals in the HAT reaction provides insight into the reasons for the preferred electron transfer pathway and its corresponding correlation with the activation barrier.

EDA identified SCS and LR residues contributing to TS stabilization and destabilization. Residues such as N344, K241, S258, N260, and D291 exhibited substrate-specific roles across different oxidation events. In **Lys­(Me/Eth)** and **Lys­(iPr)**, N344 and K241 were consistently involved in H-bonding with active site residues and substrate positioning, while N260 and S258 contributed to TS stabilization in the oxidation of hydroxy and aldehydic intermediates. Importantly, DCCA revealed that these residues are dynamically correlated with the DSBH fold and exhibit anticorrelated motions with the Zn­(II)-binding domain, underscoring their allosteric influence on catalysis. Additionally, MD simulations and QM/MM calculations on two SCS mutants, Y239A and N344A, indicated that these mutants can alter the relative positioning of the substrate in the active site and might result in altered product ratios compared to the WT enzyme.

Further, simulations of hydroxylated and aldehydic derivatives **(Lys­(Me/Eth–OH), Lys­(iPr–OH), Lys­(Me/Eth-AL)**, and **Lys­(iPr-AL))** highlighted the enzyme’s ability to modulate its active site and conformational dynamics to support subsequent oxidation steps. S258 forms a stabilizing hydrogen bond with the carbonyl group of the aldehydic groups of **Lys­(Me/Eth-AL)** and **Lys­(iPr-AL)**, thereby facilitating their further oxidation to the corresponding acids.

Our results suggest that KDM6B catalysis is governed by a complex interplay of substrate orientation, electron transfer mechanisms, and dynamic protein–substrate interactions. The regioselectivity and progression of sequential oxidations are modulated by both local chemical environments and long-range correlated motions involving the DSBH fold and Zn­(II)-binding domains.

Due to the reaction diversity of KDM6B, it can also be repurposed as a biocatalytic tool for designing and modifying peptide-based drugs.^71,113,114^ Therefore, the present computational study provides a mechanistic background that can be used to design side chain modifications into synthetic peptides that mimic H3 tail sequences. Ultimately, this integrative computational approach has the potential to broaden the catalytic repertoire of non-native, nonheme Fe­(II)/2OG-dependent enzymes and guide the enzyme engineering efforts to leverage KDM6B as a biocatalytic tool for the selective functionalization of peptide-based drugs.^115–117^

## Supplementary Material



## Notes

Additional images, optimized coordinates, and absolute energies obtained during QM/MM calculations (PDF)
