# Supplementary material for: Beyond Histone Demethylation: Mechanisms of N‑Alkyl Consecutive Oxidations by the Non-Heme Fe(II)/2-Oxoglutarate Oxygenase KDM6B
Source: JACS Au. 2026 Jun 6;6(7):4048–72. doi: 10.1021/jacsau.6c00575 (PMC13417232; doi:10.1021/jacsau.6c00575)
Supplement: Supplementary file 1 [file au6c00575_si_001.pdf]

Supplementary Information

# Beyond Histone Demethylation: Mechanisms of N-Alkyl Consecutive Oxidations by the Non-Heme Fe(II)/2-Oxoglutarate Oxygenase KDM6B

*Simahudeen Bathir Jaber Sathik Rifayee,*[a,$] *Sudheesh Devadas,*[a,$] *Midhun George Thomas,*[a] *Bhargav Varada,*[a] *Ethan Sommer,*[b] *Cassandra Talaba,*[c] *Christopher Schofield,*[d] *Christo Z. Christov.*[a]*

[a] Department of Chemistry, Michigan Technological University, Houghton, MI 49931, USA.

[b] Department of Biomedical Engineering, Michigan Technological University, Houghton, MI 49931, USA.

[c] Department of Biochemistry and Molecular Biology, Michigan Technological University, Houghton, MI 49931, USA.

[d] Chemistry Research Laboratory, Department of Chemistry, and the Ineos Oxford Institute for Antimicrobial Research, University of Oxford, Oxford, OX1 3TA, United Kingdom.

*Corresponding Author: Christo Z. Christov email: christov@mtu.edu*

**Table of Figures and Tables.**

**Scheme S1**. Consensus Mechanism of Fe(II)/2OG-dependent Oxygenases. Note variations on the consensus mechanism can occur, and the rates of individual steps can vary substantially. X is commonly an Asp/Glu residue, but in at least one case can be a water molecule.[1–5] .................. S8

**Figure S1**. Population histograms from K-means clustering analysis of molecular dynamics simulations for (a) Lys(Me/Eth), (b) Lys(Me/Eth-OH), and (c) Lys(Me/Eth-AL), based on all protein backbone atoms. The cluster selected for snapshot extraction is indicated by slanted lines. ........................................ S10
**Figure S2**. Population histograms from K-means clustering analysis of molecular dynamics simulations for (a) Lys(iPr), (b) Lys(iPr-OH), and (c) Lys(iPr -AL), based on all protein backbone atoms. The cluster selected for snapshot extraction is indicated by slanted lines. .................... S11
**Figure S3**. Conformational Dynamics of KDM6B-Fe(IV)=O•Lys(Me/Eth) system. a) The root mean square deviation (RMSD) of the dynamics suggests that the system is equilibrated; b) the root mean square fluctuation (RMSF) analysis of the system identifies flexible regions; c) the Radius of gyration (ROG) analysis shows the stability of the overall protein fold; and d) solvent accessible surface area (SAS) analysis implies that the system is equilibrated......................... S12
**Figure S4.** Substrate flexibility in WT-KDM6B-Fe(IV)=O•Lys(Me/Eth); a) Histogram plot of distance between the ferryl oxygen and different carbons of the substrate; b) Evolution of distances over time, c) Color-coded distances depicted in (a) and (b). .................................................. S13
**Figure S5.** Conformational behavior of KDM6B-Fe(IV)=O species with the Lys(Me3) substrate. a) PCA showing the dominant motions of the enzyme. Boxed regions showed dominant motions; b) DCCA plot showing the correlated/anticorrelated motions involved in the system. Boxed regions show correlated and anticorrelated motions of the flexible regions in (a). ................... S14
**Figure S6.** Conformational Dynamics of the WT-KDM6B-Fe(IV)=O•Lys(iPr) system. a) RMSD analysis of the dynamics suggests that the system is equilibrated; b) RMSF analysis of the system identifies flexible regions; c) ROG analysis shows the stability of the overall protein fold, and d) SAS implies that the system is equilibrated.......................................................................... S15
**Figure S7**. Substrate flexibility in KDM6B-Fe(IV)=O•Lys(iPr). a) Histogram plot of distance between the ferryl oxygen and different carbons of the substrate; b) Evolution of distances over time; c) The color-coded distances depicted in (a) and (b)...................................................... S16
**Figure S8.** Representative snapshot obtained from the Lys(iPr) MD simulation depicting the substrate orientation. ........................................................................................................ S17
**Figure S9.** Conformational Dynamics of the Y239A-KDM6B-Fe(IV)=O•Lys(iPr) system. a) RMSD analysis of the dynamics suggests that the system is equilibrated; b) RMSF analysis of the system identifies flexible regions; c) ROG analysis shows the stability of the overall protein fold, and d) SAS implies that the system is equilibrated................................................................. S18
**Figure S10.** Substrate flexibility in Y239A-KDM6B-Fe(IV)=O•Lys(Me/Eth); a) Histogram plot of distance between the ferryl oxygen and different carbons of the substrate; b) Evolution of distances over time, c) Color-coded distances depicted in (a) and (b). .................................... S19
**Figure S11.** Time-dependent fluctuations of (1) the hydrogen bond between the lysine amino H and Q236 carboxylate (red) and (2) the ferryl O–Cα distance of the lysine ethyl group in Y239A-KDM6B–Fe(IV)=O•Lys(Me/Eth): (a) histograms, (b) distance evolution over time, and (c) corresponding color-coded representations. ..................................................................... S20

**Figure S12.** Conformational Dynamics of the N344A-KDM6B-Fe(IV)=O•Lys(iPr) system. a) RMSD analysis of the dynamics suggests that the system is equilibrated; b) RMSF analysis of the system identifies flexible regions; c) ROG analysis shows the stability of the overall protein fold, and d) SAS implies that the system is equilibrated.................................................................. S21
**Figure S13.** Substrate flexibility in N344A-KDM6B-Fe(IV)=O•Lys(Me/Eth); a) Histogram plot of distance between the ferryl oxygen and different carbons of the substrate; b) Evolution of distances over time, c) Color-coded distances depicted in (a) and (b). .................................... S22
**Figure S14.** Time-dependent fluctuations of (1) the hydrogen bond between the lysine amino H and Q236 carboxylate (red) and (2) the ferryl O–Cα distance of the lysine ethyl group in N344A-KDM6B–Fe(IV)=O•Lys(Me/Eth): (a) histograms, (b) distance evolution over time, and (c) corresponding color-coded representations. ............................................................................ S23
**Figure S15.** Reaction Profile of the Hydroxylation of Methyl and Ethyl Groups from N344A-MEL1-RC snapshot, where methyl is closer to ferryl oxygen. The HAT profiles are shown as follows: MEL1-eth in black; WT-MEL1-eth in orange; WT-MEL1-meth in blue; MEL1-meth in green........................................................................................................................................ S24
**Figure S16.** Reaction Profile of the Hydroxylation of Methyl and Ethyl Groups from N344A-MEL2-RC snapshot, where the ethyl group is closer to ferryl oxygen. The HAT profiles are shown as follows: MEL2-meth in blue; MEL-eth-Cα in green; MEL-eth-Cβ in orange..................... S25
**Figure S17.** Reaction Profile of the Hydroxylation of Methyl and Ethyl Groups from Y239A-MEL1-RC snapshot, where methyl is closer to ferryl oxygen. The HAT profiles are shown as follows: MEL1-eth is shown in orange; MEL1-meth is shown in blue.................................. S26
**Figure S18.** Reaction Profile of the Hydroxylation of Methyl and Ethyl Groups from Y239A-MEL2-RC snapshot, where the ethyl group is closer to ferryl oxygen. The HAT profiles are shown as follows: MEL2-meth in blue; MEL2-eth-Cα in green; MEL2-eth-Cβ in orange.................. S27
**Figure S19.** Spin Natural Orbital (SNO) analysis of HAT TSs in a) MEL1-TS1-meth, and b) MEL1-TS1-eth-Cβ.......................................................................................................... S28
**Figure S20.** Spin density plots for the stationary points obtained during QM/MM simulations of the MEL1-RC snapshot.......................................................................................................... S29
**Figure S21.** Spin density plots for the stationary points obtained during QM/MM simulations of the MEL2-RC snapshot.......................................................................................................... S30
**Figure S22.** Spin Natural Orbital (SNO) analysis of HAT TSs in a) MEL2-TS1-eth-Cα, and b) MEL2-TS1-eth-Cβ.......................................................................................................... S31
**Figure S23.** Frontier Molecular Orbitals (FMO) for the HAT mechanism and their energies in the MEL1-RC. ...................................................................................................................... S32
**Figure S24.** FMO for the HAT mechanism and their energies in the MEL2-RC...................... S33
**Figure S25.** KDE plot of the O-X distance (in Å) versus the ∠Fe-O-X (in °) for the MD simulations of KDM6B-Fe(IV)=O•Lys(Me/Eth) system. X denotes carbons of the substrate..................... S34
**Figure S26**. EDA analysis of HAT and rebound reactions of methyl (a and b) and ethyl (c and d) groups in MEL1-RC snapshot. ........................................................................................ S35
**Figure S27.** EDA analysis of HAT and rebound reactions of ethyl Cα (a and b) and ethyl Cβ (c and d) groups in the MEL2-RC snapshot. ...................................................................... S36
**Figure S28.** Spin Natural Orbital (SNO) analysis of HAT TSs in a) IL1-TS1-Cα, and b) IL1-TS1-Cβ............................................................................................................................. S37
**Figure S29.** Spin density plots for the stationary points obtained during QM/MM simulations of the IL1-RC snapshot. ........................................................................................................ S38
**Figure S30.** FMO for the HAT mechanism and their energies in the IL1-RC. ......................... S39

**Figure S31.** KDE plot of the O-X distance (in Å) versus the ∠Fe-O-X (in °) for the MD simulations of KDM6B-Fe(IV)=O•Lys(iPr) system. X denotes carbons of the substrate. .......................... S40
**Figure S32.** EDA analysis of HAT and rebound reactions of Cα (a and b) and Cβ (c and d) of the iPr group in the IL1-RC snapshot. .......................... S41
**Figure S33.** Conformational Dynamics of KDM6B-Fe(IV)=O•Lys(Me/Eth-OH) system. a) RMSD analysis of the dynamics suggests that the system is equilibrated, b) RMSF analysis of the system identifies flexible regions, c) ROG analysis shows the stability of the overall protein fold, and d) SAS analysis implies that the system is equilibrated. .......................... S42
**Figure S34.** Conformational Dynamics of KDM6B-Fe(IV)=O•Lys(iPr-OH) system. a) RMSD analysis of the dynamics suggests that the system is equilibrated, b) RMSF analysis of the system identifies flexible regions, c) ROG analysis shows the stability of the overall protein fold, and d) SAS analysis implies that the system is equilibrated. .......................... S43
**Figure S35.** Substrate flexibility in KDM6B-Fe(IV)=O•Lys(Me/Eth-OH). a) Histogram plot of distance between the ferryl oxygen and different carbons and oxygen of the substrate, b) Evolution of distances over time, c) the color-coded distances depicted in (a). .......................... S44
**Figure S36.** Substrate flexibility in KDM6B-Fe(IV)=O•Lys(iPr-OH). a) Histogram plot of distance between the ferryl oxygen and different carbons and oxygen of the substrate, b) Evolution of distances over time, c) the color-coded distances depicted in (a). .......................... S45
**Figure S37.** a) Reaction Profile of the oxidation of **Lys(Me/Eth-OH)** through water-mediated HAT. Relative energies are presented at the QM(B3)/MM level. The energy profile in orange shows the initiation of substrate oxidation by Cβ-HAT, and the energy profile in blue shows the initiation of substrate oxidation by O-HAT. .......................... S46
**Figure S38.** QM/MM optimized stationary points obtained during PES simulations from MEL1-OH-RC$_{wat}$ during hydroxylation of a) methyl, and b) ethyl Cβ carbons through water-mediated HAT. Distances are given in Å and represented by yellow dashed lines. .......................... S47
**Figure S39.** Mechanism of aldehyde formation through the gem-diol pathway. .......................... S48
**Figure S40.** a) Reaction Profile of the oxidation of **Lys(Me/Eth-OH)** through gem-diol pathway. Relative energies are presented at the QM(B3)/MM level. Reaction profile bifurcation in blue and orange shows different dehydration possibilities. .......................... S49
**Figure S41.** a) Reaction Profile of the oxidation of **Lys(IL-OH)** through water-mediated HAT. Relative energies are presented at the QM(B3)/MM level. The energy profile in orange shows the initiation of substrate oxidation by Cβ-HAT, and the energy profile in blue shows the initiation of substrate oxidation by O-HAT. .......................... S50
**Figure S42.** Spin Natural Orbital (SNO) analysis of HAT TSs in a) MEL-OH-TS1-O, and b) MEL-OH-TS1-Cβ. .......................... S51
**Figure S43.** Spin density plots for the stationary points obtained during QM/MM simulations of the MEL-OH-RC snapshot. .......................... S52
**Figure S44.** FMO for the HAT mechanism and their energies in the MEL-OH-RC. .......................... S53
**Figure S45.** KDE plot of the O-X distance (in Å) versus the ∠Fe-O-X (in °) for the MD simulations of the KDM6B-Fe(IV)=O•Lys(Me/Eth-OH) system. X denotes carbons and oxygen of the substrate. .......................... S54
**Figure S46.** EDA analysis of HAT reactions initiated from $O_{oh}$ (a and b) and Cβ (c and d) of the Eth-OH group in MEL-OH-RC snapshot. .......................... S55
**Figure S47.** Spin Natural Orbital (SNO) analysis of HAT TSs in a) IL-OH-TS1-O, and b) IL-OH-TS1-Cβ. .......................... S56

**Figure S48.** Spin density plots for the stationary points obtained during QM/MM simulations of the IL-OH-RC snapshot. ........ S57
**Figure S49.** FMO for the HAT mechanism and their energies in the IL-OH-RC. ........ S58
**Figure S50.** KDE plot of the O-X distance (in Å) versus the ∠Fe-O-X (in °) for the MD simulations of KDM6B-Fe(IV)=O•Lys(iPr-OH) system. X denotes carbons and oxygen of the substrate. . S59
**Figure S51.** EDA analysis of HAT reactions initiated from $O_{oh}$ (a and b) and Cβ (c and d) of the iPr-OH group of the IL-OH-RC snapshot. ........ S60
**Figure S52.** Conformational Dynamics of the KDM6B-Fe(IV)=O•Lys(Me/Eth-AL) system. a) RMSD analysis of the dynamics suggests that the system is equilibrated, b) RMSF analysis of the system identifies flexible regions, c) ROG analysis shows the stability of the overall protein fold, and d) SAS analysis implies that the system is equilibrated. ........ S61
**Figure S53.** Conformational Dynamics of the KDM6B-Fe(IV)=O•Lys(iPr-AL) system. a) RMSD analysis of the dynamics suggests that the system is equilibrated, b) RMSF analysis of the system identifies flexible regions, c) ROG analysis shows the stability of the overall protein fold, and d) SAS analysis implies that the system is equilibrated. ........ S62
**Figure S54.** Substrate flexibility in KDM6B-Fe(IV)=O•Lys(Me/Eth-AL). a) Histogram plot of the distance between the ferryl oxygen and different carbons and hydrogen of the substrate, b) Evolution of distances over time, c) ChemDraw image showing the color-coded distances depicted in (a). ........ S63
**Figure S55.** Substrate flexibility in KDM6B-Fe(IV)=O•Lys(iPr-AL). a) Histogram plot of the distance between the ferryl oxygen and different carbons and hydrogen of the substrate, b) Evolution of distances over time, c) the color-coded distances depicted in (a). ........ S64
**Figure S56.** Hydrogen bonding interactions around the active in oxidation of Lys(Me/Eth-AL) (left) and Lys(iPr-AL) (right) ........ S65
**Figure S57.** Spin Natural Orbital (SNO) analysis of HAT TSs in MEL-AL-TS1 ........ S66
**Figure S58.** Spin density plots for the stationary points obtained during QM/MM simulations of the MEL-AL-RC snapshot. ........ S67
**Figure S59.** FMO for the HAT mechanism and their energies in the MEL-AL-RC. ........ S68
**Figure S60.** KDE plot of the O-X distance (in Å) versus the ∠Fe-O-X (in °) for the MD simulations of the KDM6B-Fe(IV)=O•Lys(Me/Eth-AL) system. X denotes carbons and hydrogen of the substrate. ........ S69
**Figure S61.** EDA analysis of the HAT and rebound reactions of MEL-AL group in the MEL-AL-RC snapshot. ........ S70
**Figure S62.** Overlaid TSs of the HAT from Lys(Me/Eth-AL) and Lys(iPr-AL). ........ 71
**Figure S63.** Spin density plots for the stationary points obtained during QM/MM simulations of the IL-AL-RC snapshot ........ S72
**Figure S64.** Spin Natural Orbital (SNO) analysis of HAT TSs in IL-AL-TS1. ........ S73
**Figure S65**. FMO for the HAT mechanism and their energies in the IL-AL-RC. ........ S74
**Figure S66.** KDE plot of the O-X distance (in Å) versus the ∠Fe-O-X (in °) for the MD simulations of KDM6B-Fe(IV)=O•Lys(iPr-AL) system. X denotes carbons and hydrogen of the substrate. ........ S75
**Figure S67.** EDA analysis of the HAT and rebound reactions of iPr-AL group in the IL-AL-RC snapshot ........ S76
**Figure S68.** a) Reaction Profile of the conversion of IL-OH-IM1-$O_{wat}$ to Aldehyde product through water-mediated HAT. Relative energies are presented at the QM(B3)/MM level........ S77

**Figure S69.** a) Reaction Profile of the conversion of IL-OH-IM1$_{wat}$ to Aldehyde product through water-mediated HAT. Relative energies are presented at the QM(B3)/MM level. .................... S78

**Table S1**: Imaginary frequencies of transition states computed in this study. ........................... S79
**Table S2:** Calculated MM/GBSA values from the equilibrated regions from the MD simulations for the different substrates considered in the study. ................................................................... S81

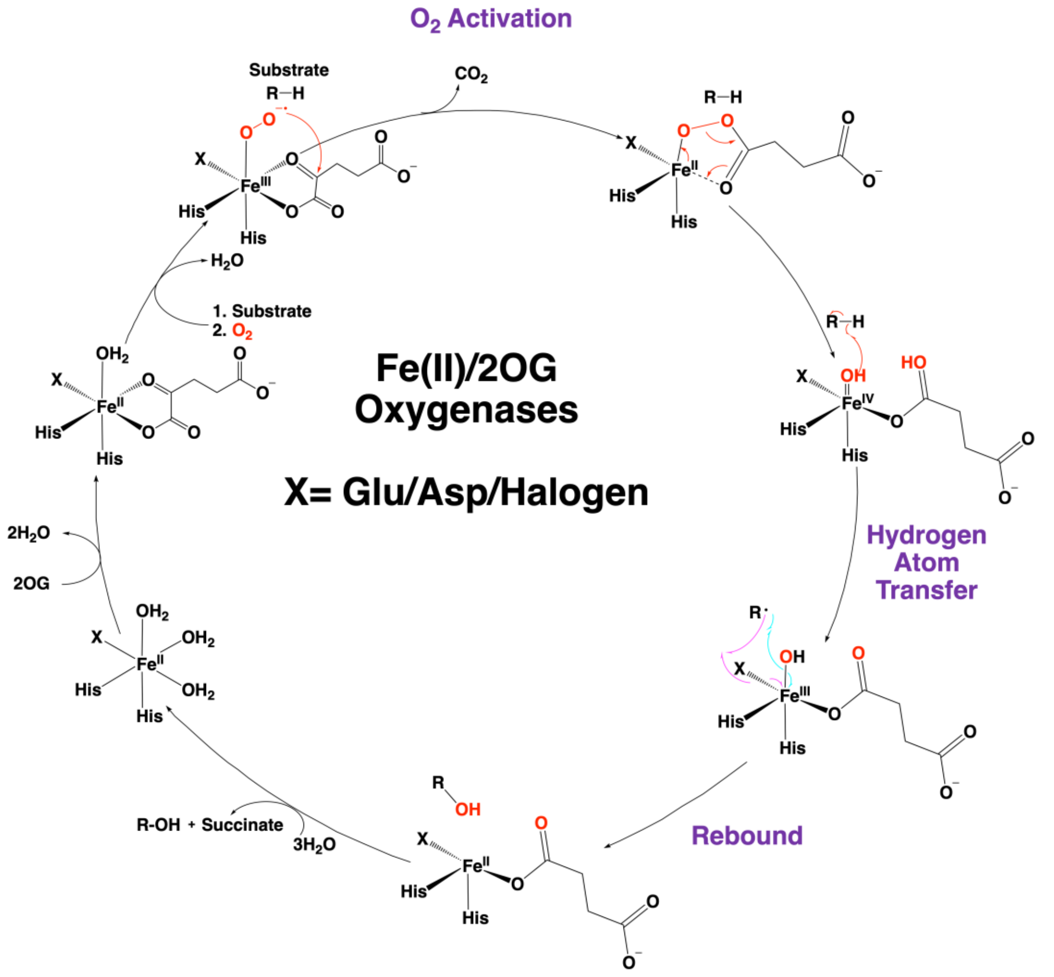


**Scheme S1**. Consensus Mechanism of Fe(II)/2OG-dependent Oxygenases. Note variations on the consensus mechanism can occur, and the rates of individual steps can vary substantially. X is commonly an Asp/Glu residue, but in at least one case can be a water molecule.[1–5]

## Clustering Analysis for QM/MM Snapshot Selection

MD trajectories were analyzed using different clustering approaches to identify representative structures for subsequent QM/MM calculations. Two types of clustering were performed. First, distance-based k-means clustering[6] was performed using geometric descriptors defined by distances between the ferryl oxygen and key substrate atoms. The choice of distances depended on the type of substrate:

**Lys(Me/Eth):** distances between i) ferryl oxygen and the methyl carbon; ii) the ferryl oxygen and Cα carbon of the ethyl group; iii) the ferryl oxygen and Cβ atom of the ethyl group of the substrate.

**Lys(Me/Eth–OH):** distances between i) the ferryl oxygen and the methyl carbon; (ii) ferryl oxygen and hydroxyl oxygen of the substrate; and (iii) ferryl oxygen and the Cβ atom of the ethyl group.

**Lys(Me/Eth–AL):** distances between (i) the ferryl oxygen and the methyl carbon; (ii) the ferryl oxygen and the Cβ atom of the ethyl group of the substrate; and (iii) the ferryl oxygen and the aldehydic hydrogen.

**Lys(iPr):** distances between the ferryl oxygen and the three isopropyl carbons.

**Lys(iPr–OH):** distances between (i) the ferryl oxygen and hydroxyl oxygen of the substrate; (ii) the ferryl oxygen and hydroxyl hydrogen of the substrate; and (iii) the ferryl oxygen and Cα atom of the substrate.

**Lys(iPr–AL):** distances between (i) the ferryl oxygen and the Cα atom of the substrate; (ii) the ferryl oxygen and the carbonyl oxygen of the aldehydic group of the substrate; (iii) the ferryl oxygen and the aldehydic hydrogen of the substrate

To verify that the selected QM/MM snapshots were representative of broader protein structure clustering, we also performed k-means clustering on all protein backbone atoms. The snapshots ultimately chosen for QM/MM calculations were confirmed to belong to the most populated protein conformational cluster, indicating that both the local reactive geometry and the overall protein conformation were adequately represented. The results of the protein-backbone clustering analysis are shown in (Figure S1-S2).

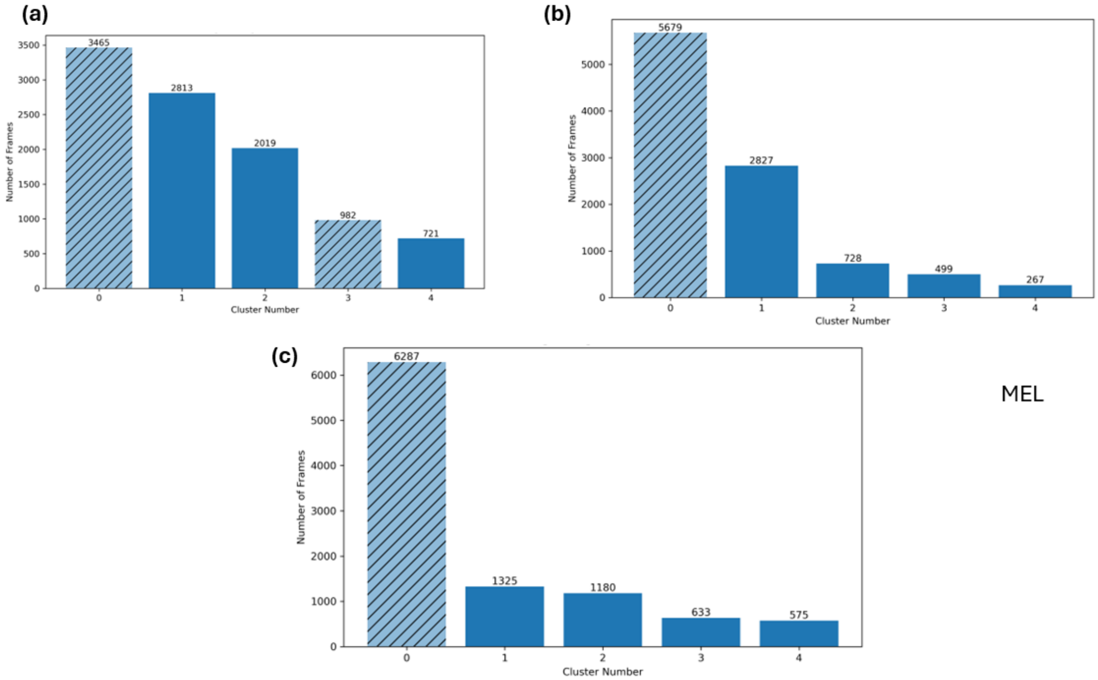


**Figure S1**. Population histograms from K-means clustering analysis of molecular dynamics simulations for (a) Lys(Me/Eth), (b) Lys(Me/Eth-OH), and (c) Lys(Me/Eth-AL), based on all protein backbone atoms. The cluster selected for snapshot extraction is indicated by slanted lines.

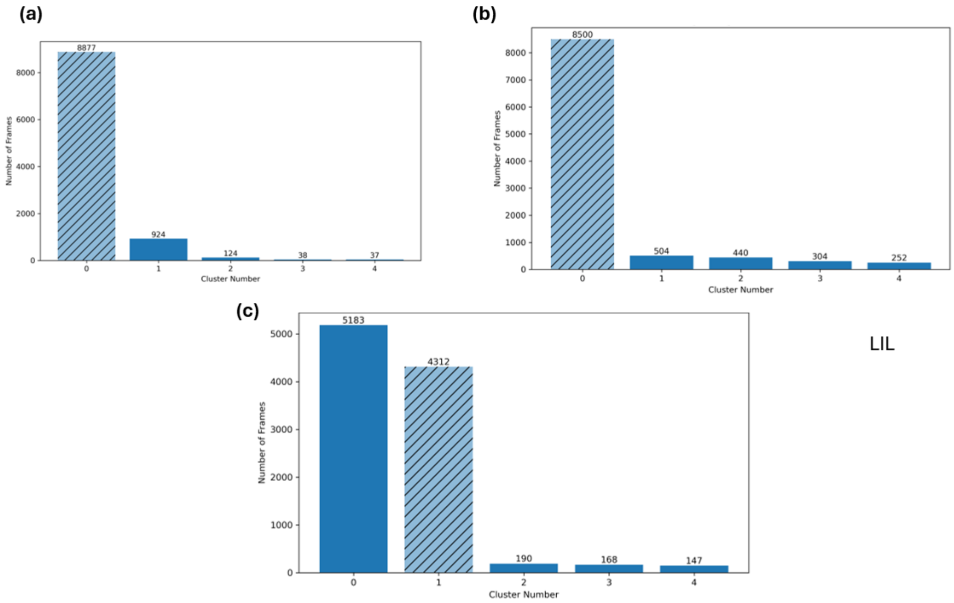


**Figure S2**. Population histograms from K-means clustering analysis of molecular dynamics simulations for (a) Lys(iPr), (b) Lys(iPr-OH), and (c) Lys(iPr -AL), based on all protein backbone atoms. The cluster selected for snapshot extraction is indicated by slanted lines.

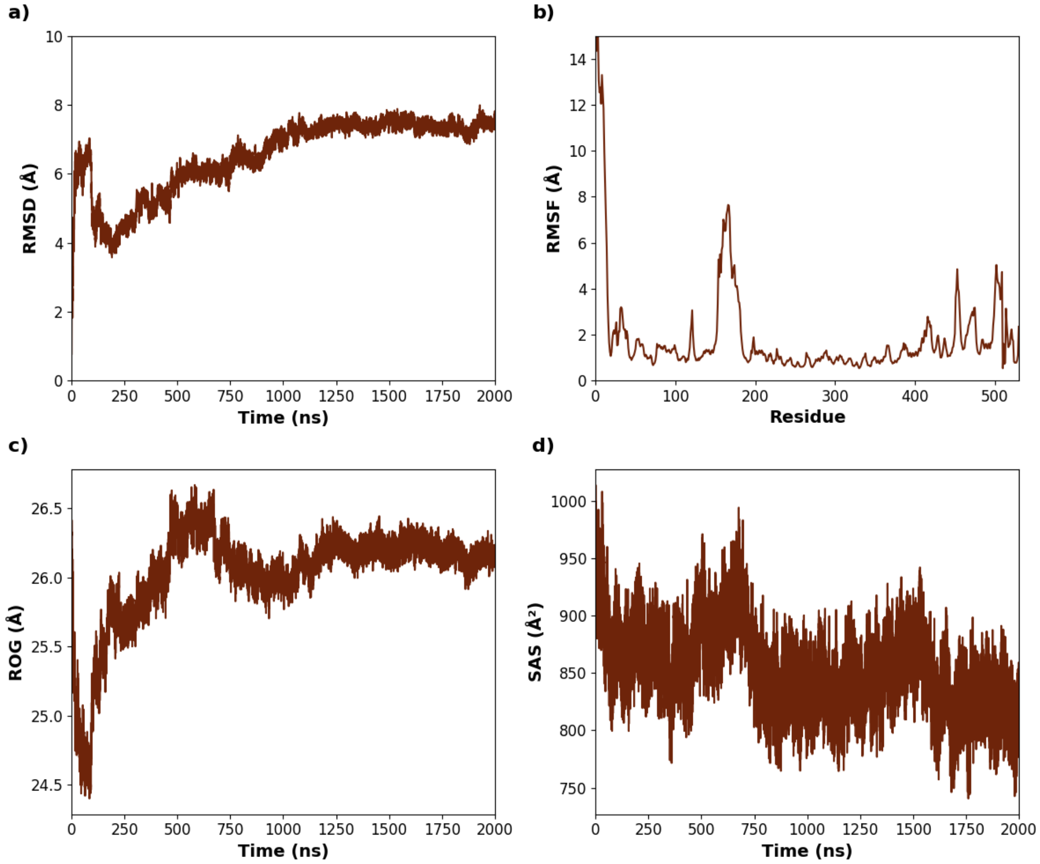


**Figure S3**. Conformational Dynamics of KDM6B-Fe(IV)=O•Lys(Me/Eth) system. a) The root mean square deviation (RMSD) of the dynamics suggests that the system is equilibrated; b) the root mean square fluctuation (RMSF) analysis of the system identifies flexible regions; c) the Radius of gyration (ROG) analysis shows the stability of the overall protein fold; and d) solvent accessible surface area (SAS) analysis implies that the system is equilibrated.

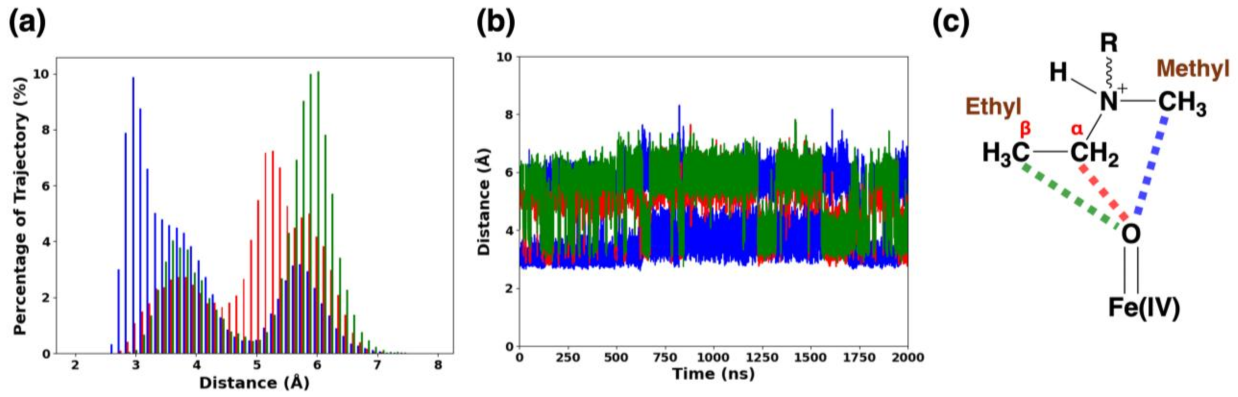


**Figure S4.** Substrate flexibility in WT-KDM6B-Fe(IV)=O•Lys(Me/Eth); a) Histogram plot of distance between the ferryl oxygen and different carbons of the substrate; b) Evolution of distances over time, c) Color-coded distances depicted in (a) and (b).

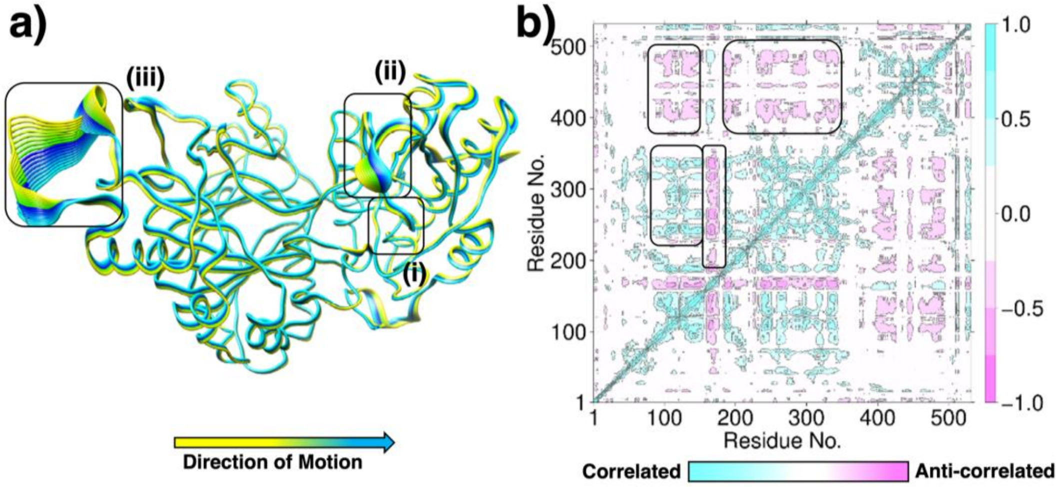


**Figure S5.** Conformational behavior of KDM6B-Fe(IV)=O species with the Lys(Me3) substrate. a) PCA showing the dominant motions of the enzyme. Boxed regions showed dominant motions; b) DCCA plot showing the correlated/anticorrelated motions involved in the system. Boxed regions show correlated and anticorrelated motions of the flexible regions in (a).

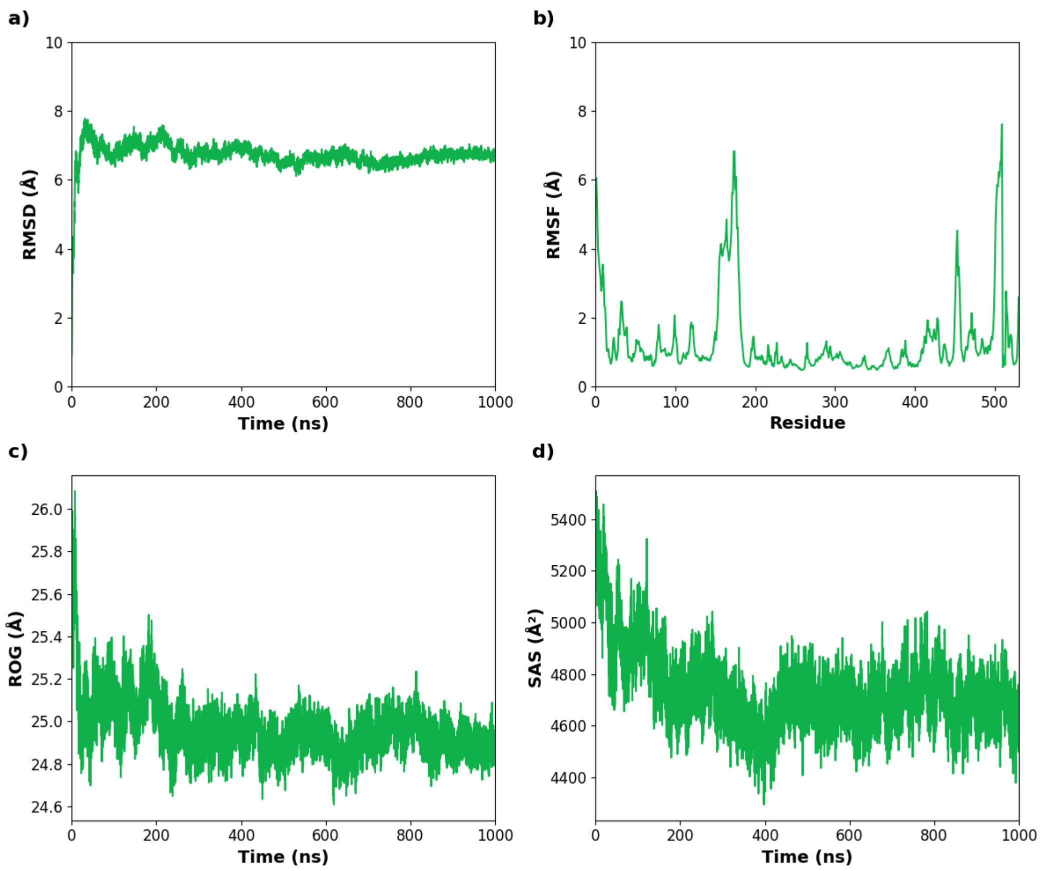


**Figure S6.** Conformational Dynamics of the WT-KDM6B-Fe(IV)=O•Lys(iPr) system. a) RMSD analysis of the dynamics suggests that the system is equilibrated; b) RMSF analysis of the system identifies flexible regions; c) ROG analysis shows the stability of the overall protein fold, and d) SAS implies that the system is equilibrated.

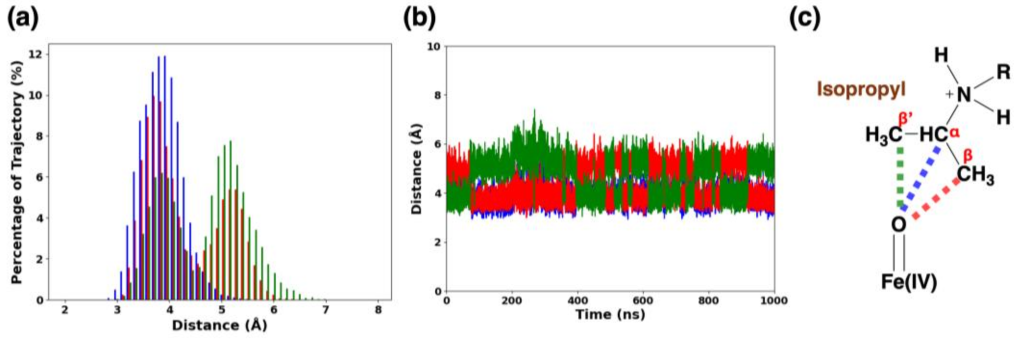


**Figure S7**. Substrate flexibility in KDM6B-Fe(IV)=O•Lys(iPr). a) Histogram plot of distance between the ferryl oxygen and different carbons of the substrate; b) Evolution of distances over time; c) The color-coded distances depicted in (a) and (b).

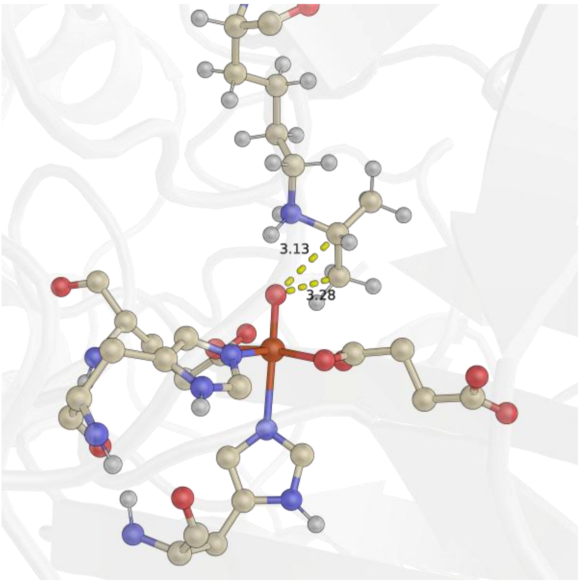


**Figure S8.** Representative snapshot obtained from the Lys(iPr) MD simulation depicting the substrate orientation.

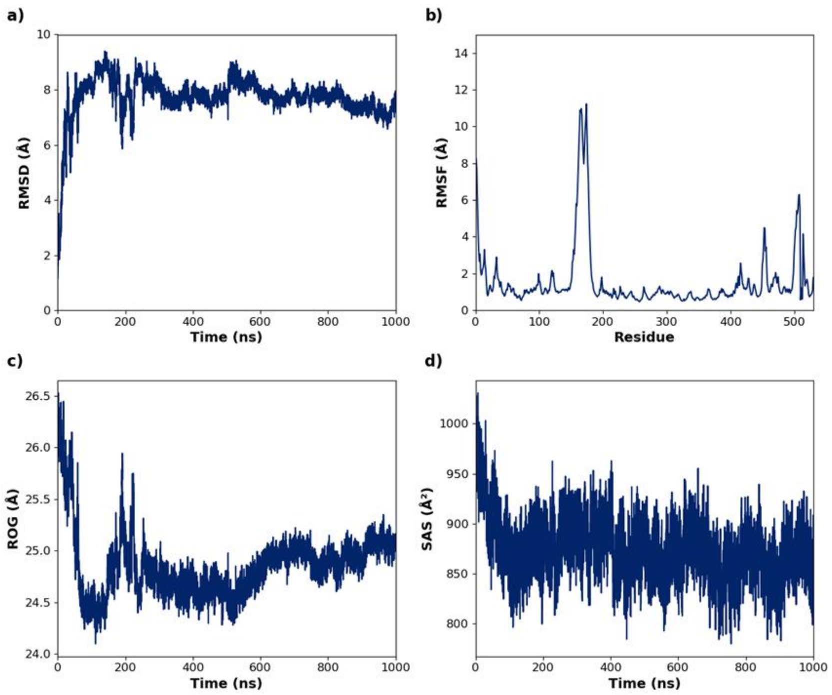


**Figure S9.** Conformational Dynamics of the Y239A-KDM6B-Fe(IV)=O•Lys(iPr) system. a) RMSD analysis of the dynamics suggests that the system is equilibrated; b) RMSF analysis of the system identifies flexible regions; c) ROG analysis shows the stability of the overall protein fold, and d) SAS implies that the system is equilibrated.

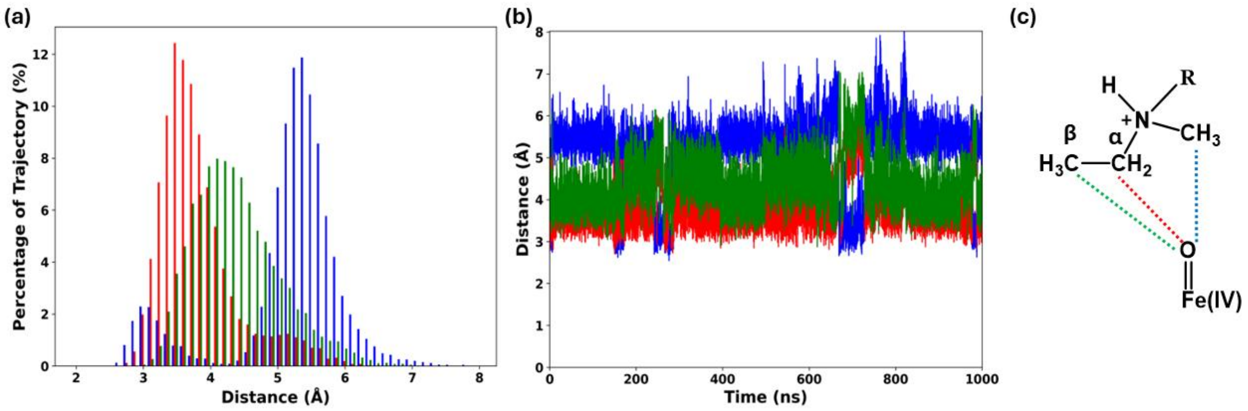


**Figure S10.** Substrate flexibility in Y239A-KDM6B-Fe(IV)=O•Lys(Me/Eth); a) Histogram plot of distance between the ferryl oxygen and different carbons of the substrate; b) Evolution of distances over time, c) Color-coded distances depicted in (a) and (b).

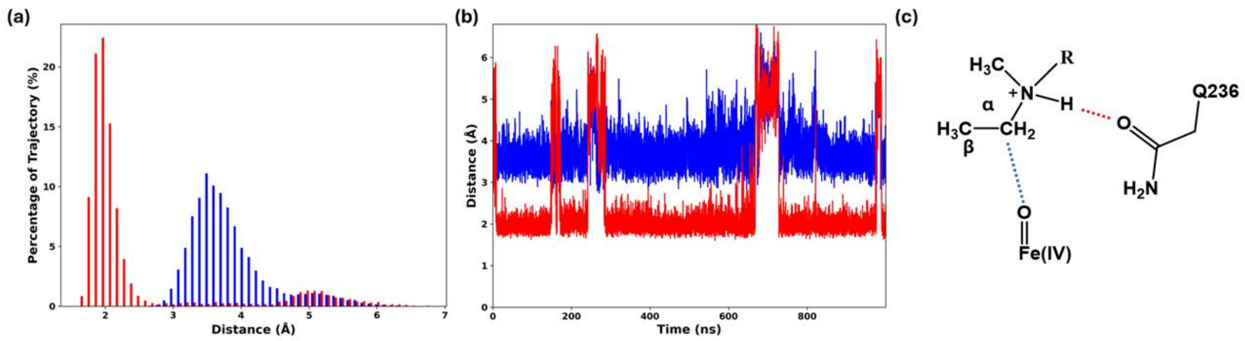


**Figure S11.** Time-dependent fluctuations of (1) the hydrogen bond between the lysine amino H and Q236 carboxylate (red) and (2) the ferryl O–Cα distance of the lysine ethyl group in Y239A-KDM6B–Fe(IV)=O•Lys(Me/Eth): (a) histograms, (b) distance evolution over time, and (c) corresponding color-coded representations.

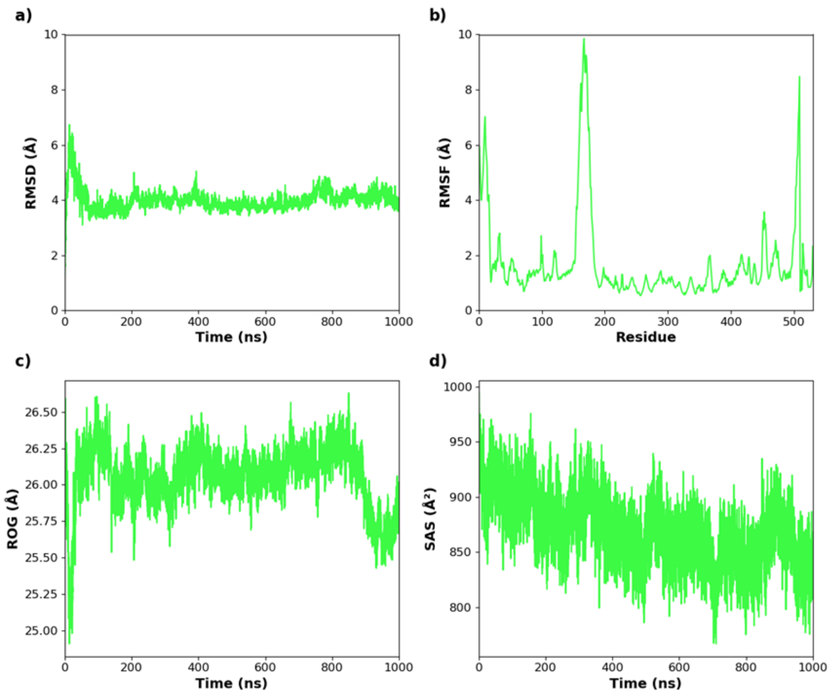


**Figure S12.** Conformational Dynamics of the N344A-KDM6B-Fe(IV)=O•Lys(iPr) system. a) RMSD analysis of the dynamics suggests that the system is equilibrated; b) RMSF analysis of the system identifies flexible regions; c) ROG analysis shows the stability of the overall protein fold, and d) SAS implies that the system is equilibrated.

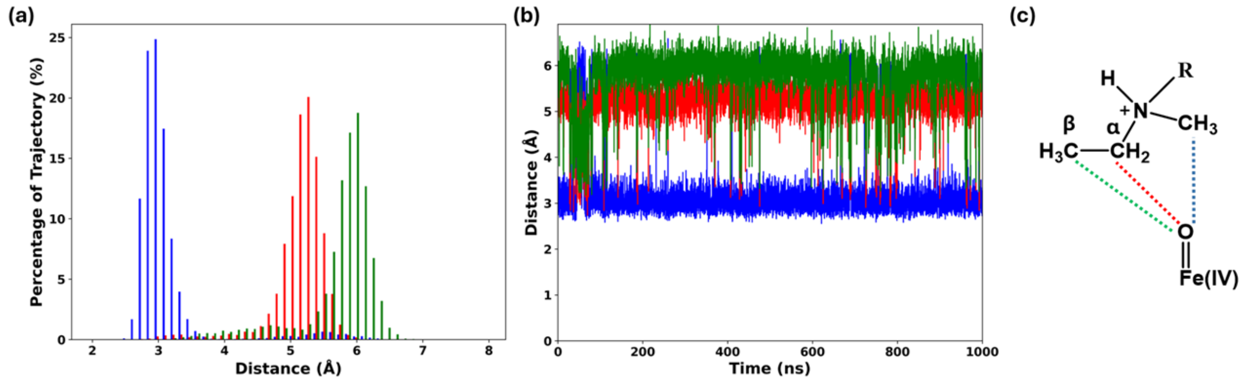


**Figure S13.** Substrate flexibility in N344A-KDM6B-Fe(IV)=O•Lys(Me/Eth); a) Histogram plot of distance between the ferryl oxygen and different carbons of the substrate; b) Evolution of distances over time, c) Color-coded distances depicted in (a) and (b).

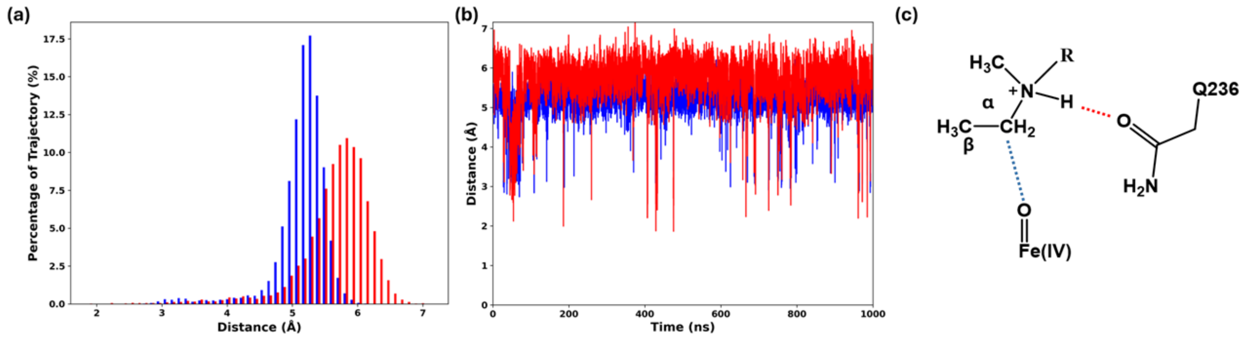


**Figure S14.** Time-dependent fluctuations of (1) the hydrogen bond between the lysine amino H and Q236 carboxylate (red) and (2) the ferryl O–Cα distance of the lysine ethyl group in N344A-KDM6B–Fe(IV)=O•Lys(Me/Eth): (a) histograms, (b) distance evolution over time, and (c) corresponding color-coded representations.

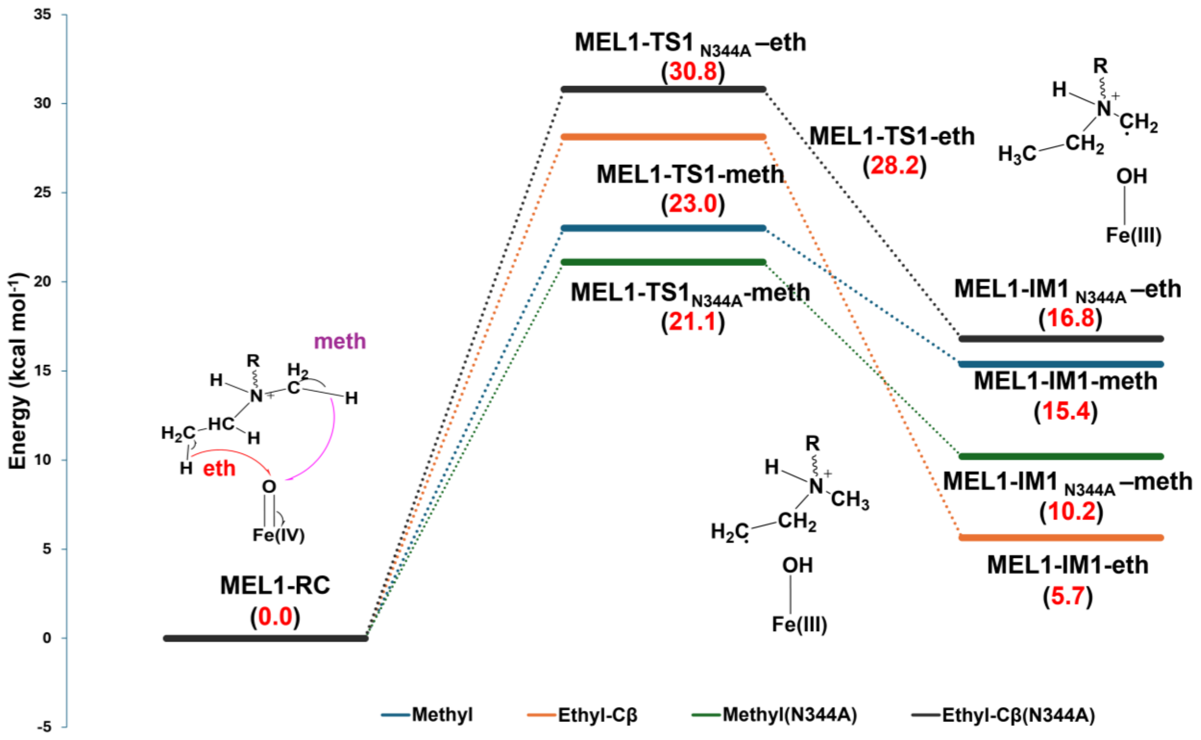


**Figure S15.** Reaction Profile of the Hydroxylation of Methyl and Ethyl Groups from N344A-MEL1-RC snapshot, where methyl is closer to ferryl oxygen. The HAT profiles are shown as follows: MEL1-eth in black; WT-MEL1-eth in orange; WT-MEL1-meth in blue; MEL1-meth in green.

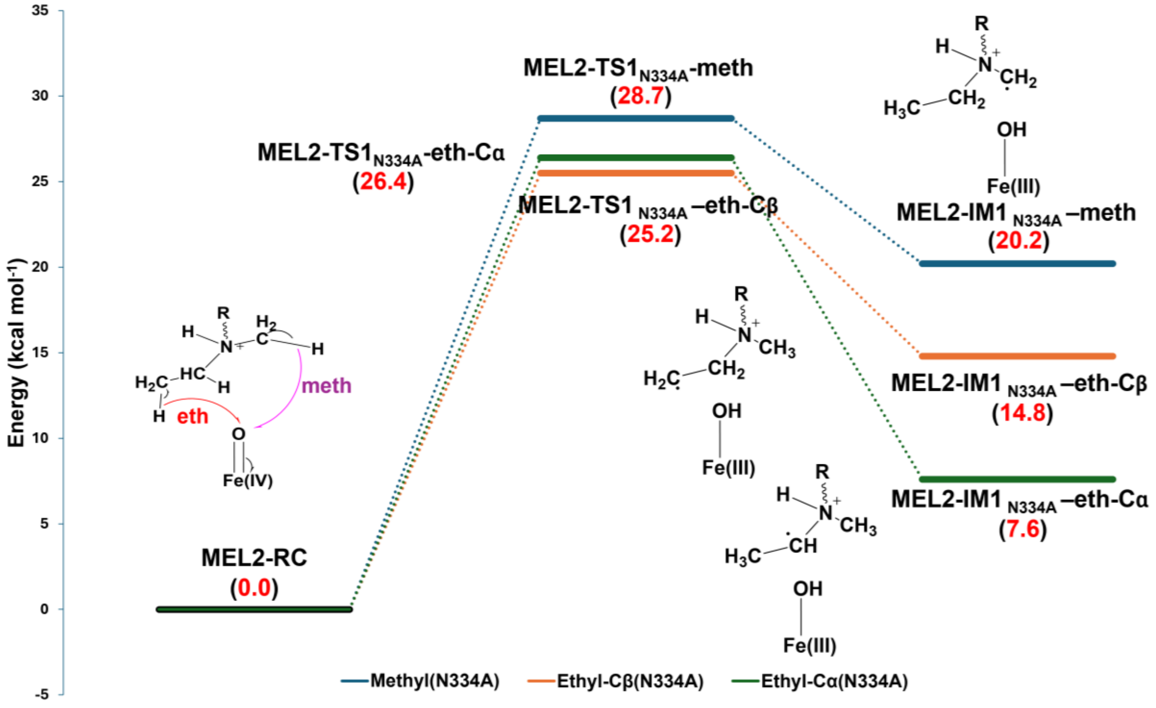


**Figure S16.** Reaction Profile of the Hydroxylation of Methyl and Ethyl Groups from N344A-MEL2-RC snapshot, where the ethyl group is closer to ferryl oxygen. The HAT profiles are shown as follows: MEL2-meth in blue; MEL-eth-Cα in green; MEL-eth-Cβ in orange.

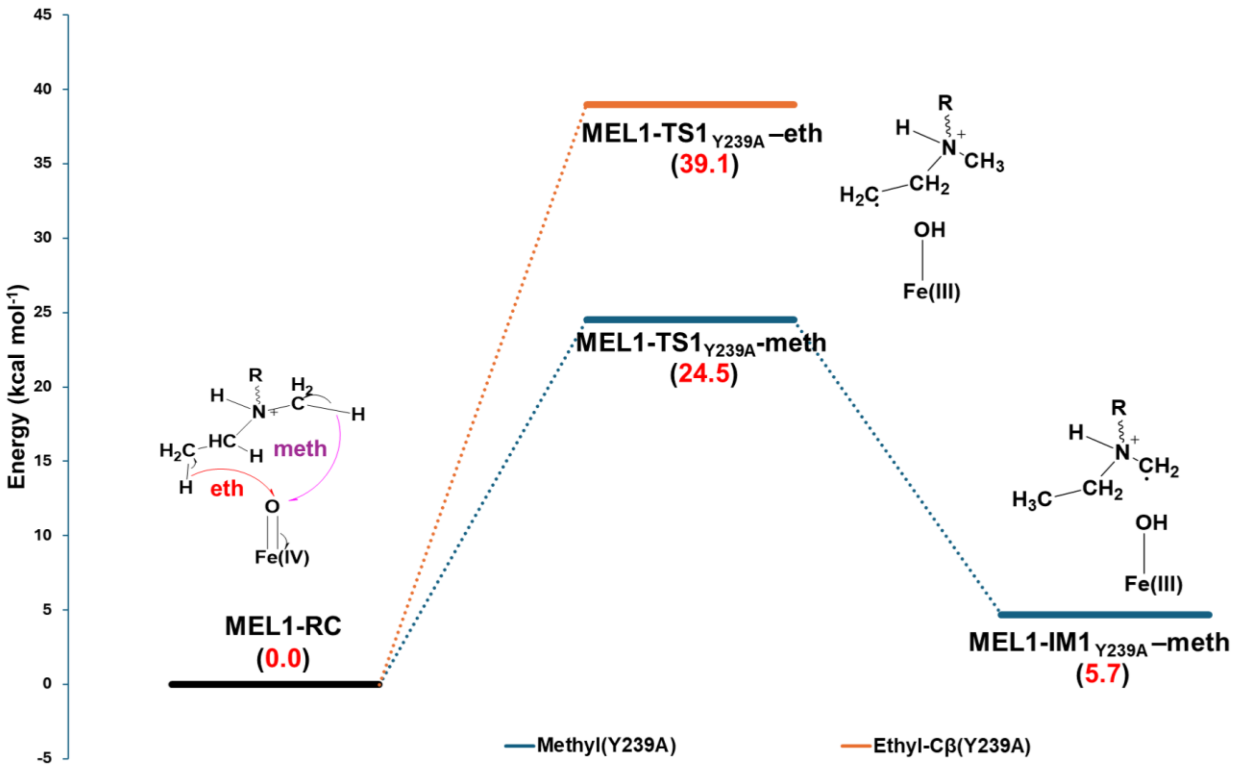


**Figure S17.** Reaction Profile of the Hydroxylation of Methyl and Ethyl Groups from Y239A-MEL1-RC snapshot, where methyl is closer to ferryl oxygen. The HAT profiles are shown as follows: MEL1-eth is shown in orange; MEL1-meth is shown in blue.

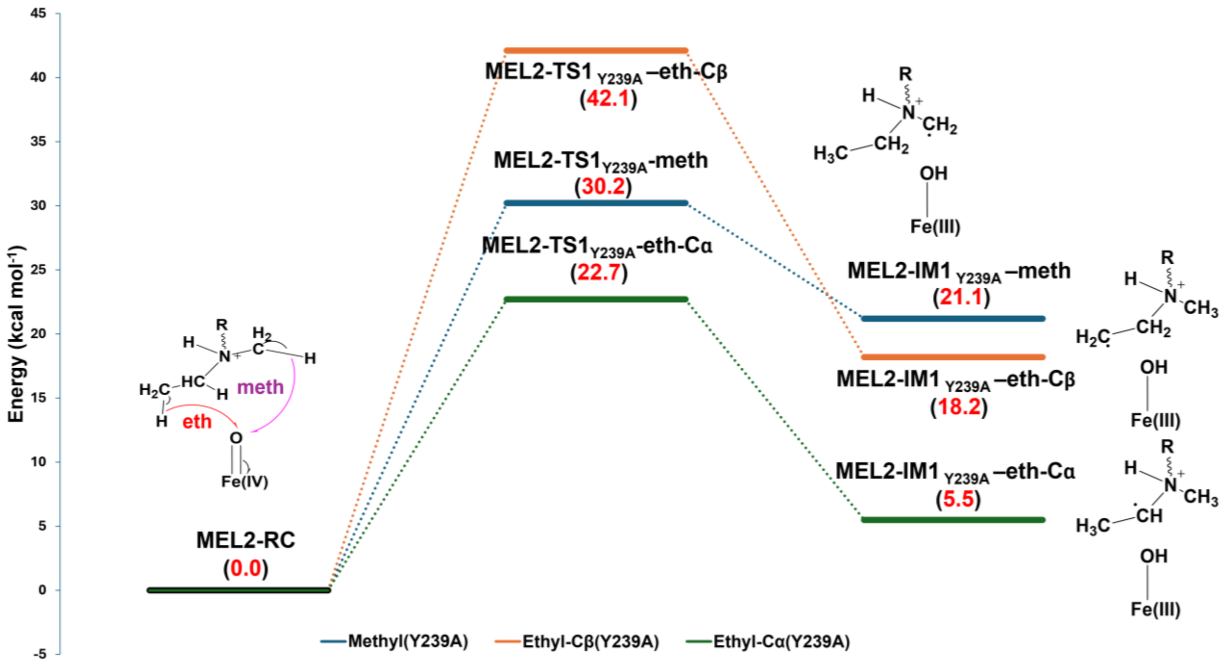


**Figure S18.** Reaction Profile of the Hydroxylation of Methyl and Ethyl Groups from Y239A-MEL2-RC snapshot, where the ethyl group is closer to ferryl oxygen. The HAT profiles are shown as follows: MEL2-meth in blue; MEL2-eth-Cα in green; MEL2-eth-Cβ in orange.

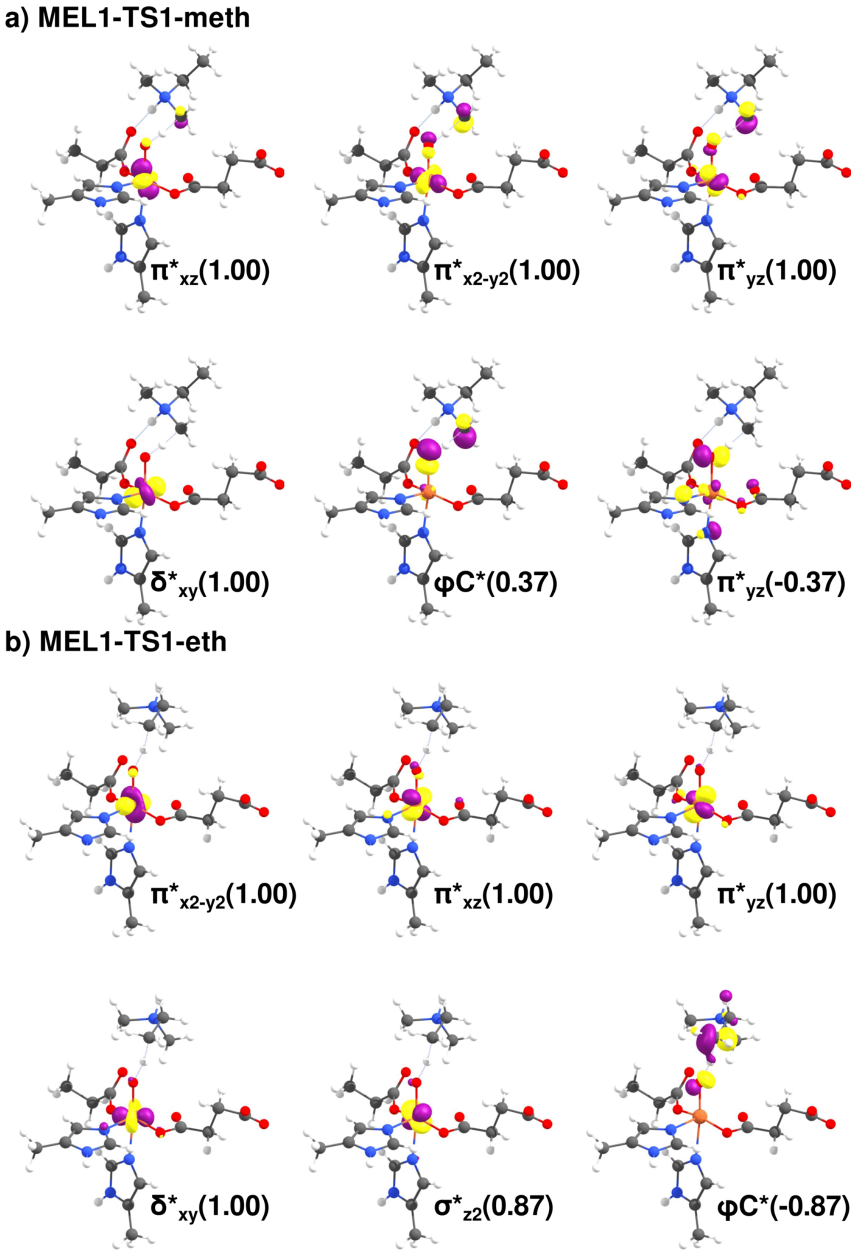


**Figure S19.** Spin Natural Orbital (SNO) analysis of HAT TSs in a) MEL1-TS1-meth, and b) MEL1-TS1-eth-Cβ.

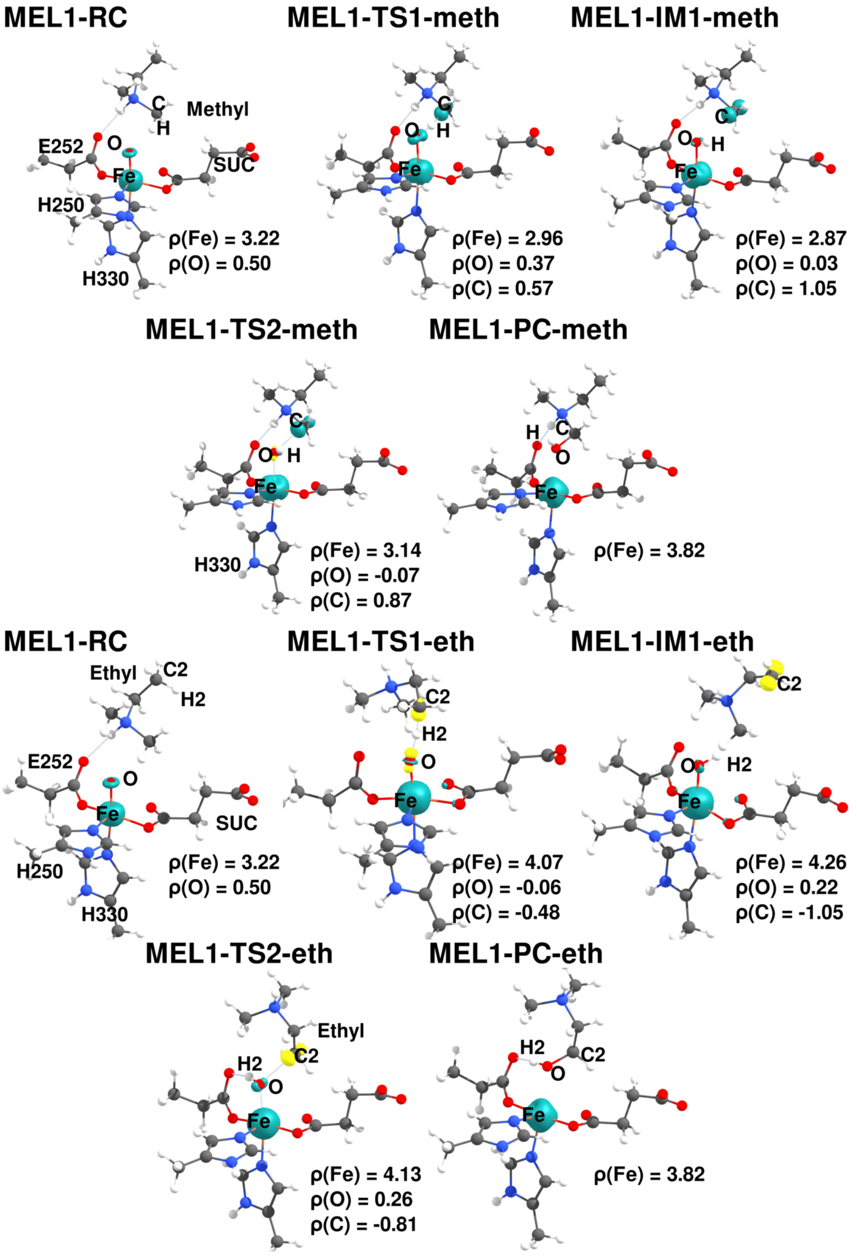


**Figure S20.** Spin density plots for the stationary points obtained during QM/MM simulations of the MEL1-RC snapshot.

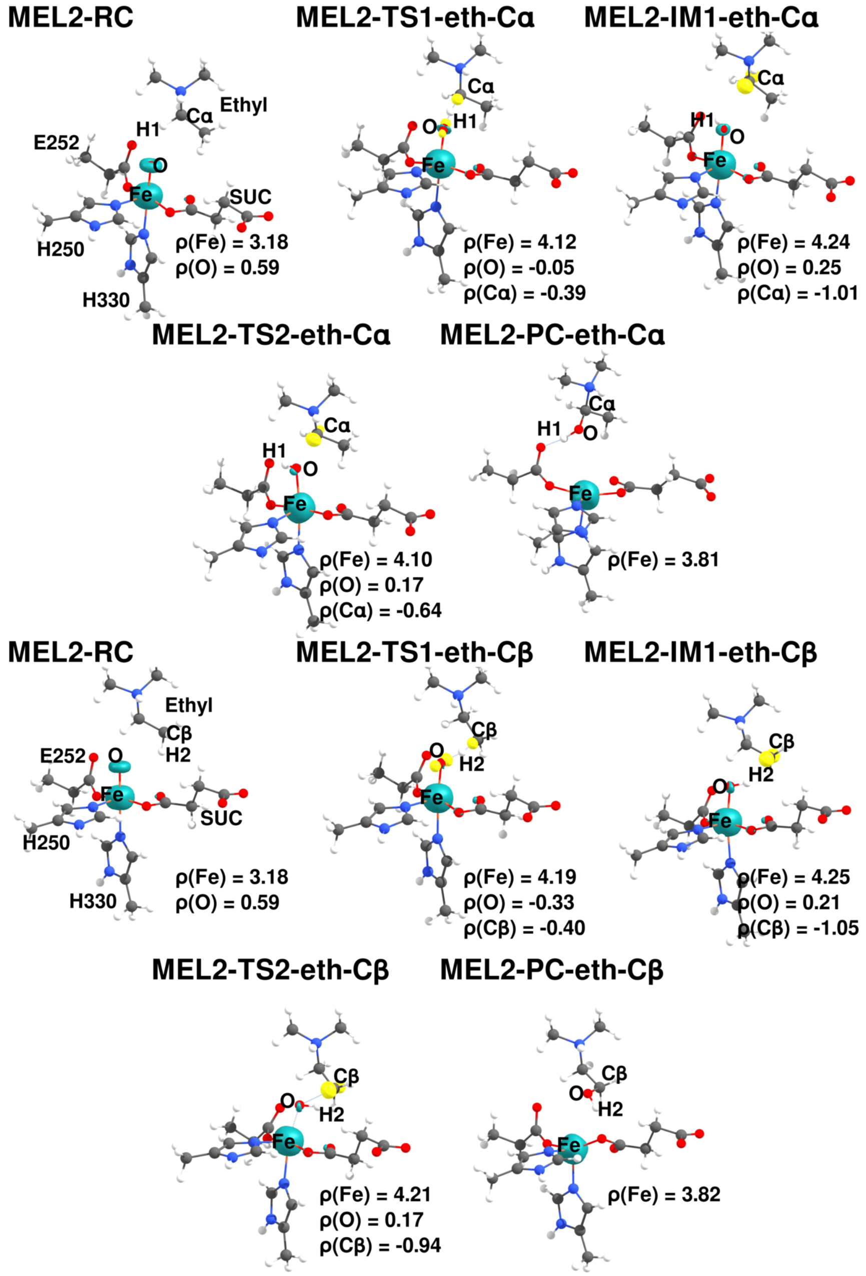


**Figure S21.** Spin density plots for the stationary points obtained during QM/MM simulations of the MEL2-RC snapshot.

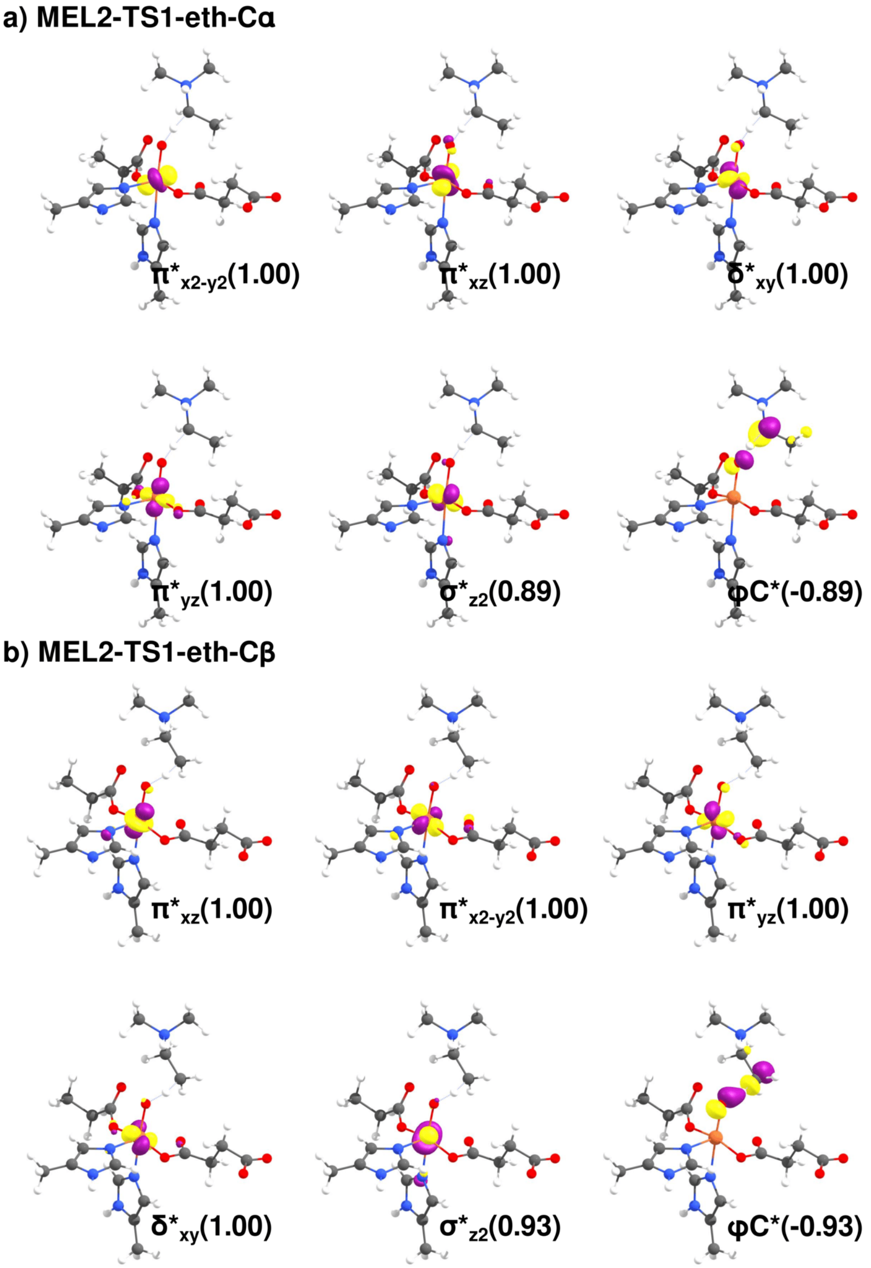


**Figure S22.** Spin Natural Orbital (SNO) analysis of HAT TSs in a) MEL2-TS1-eth-Cα, and b) MEL2-TS1-eth-Cβ

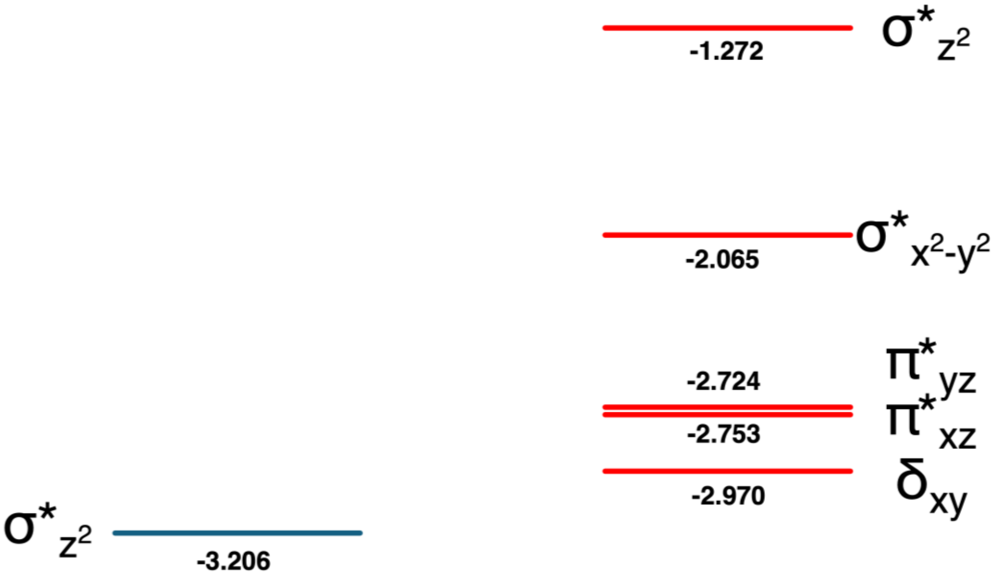


**Figure S23.** Frontier Molecular Orbitals (FMO) for the HAT mechanism and their energies in the MEL1-RC.

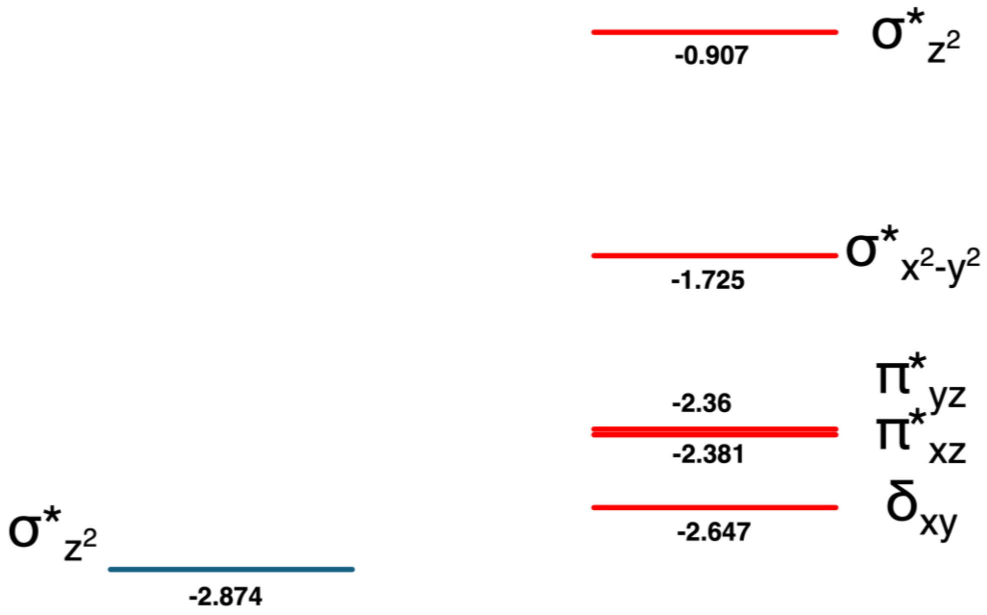


**Figure S24.** FMO for the HAT mechanism and their energies in the MEL2-RC.

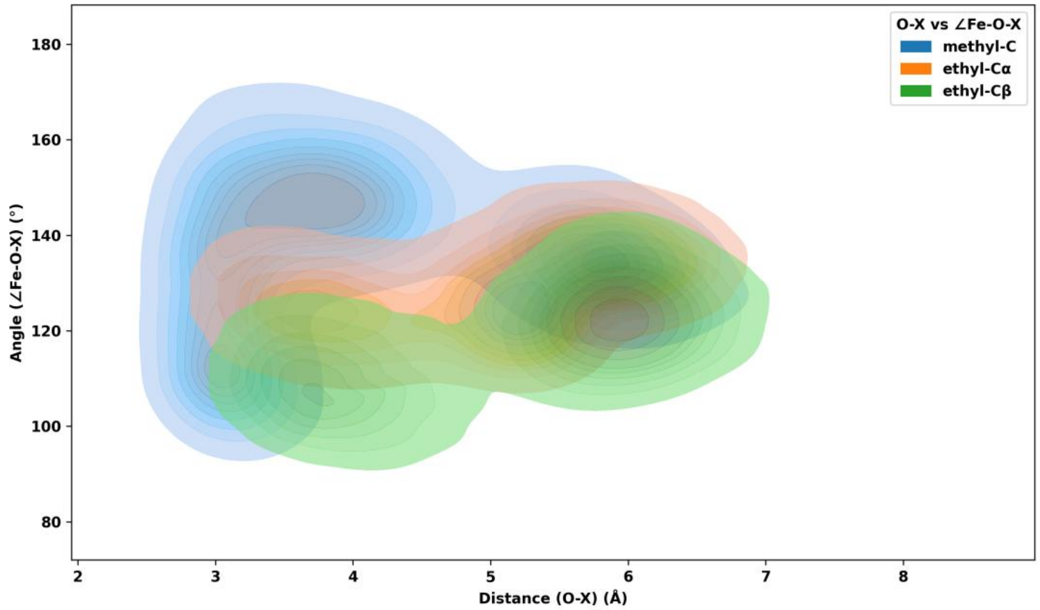


**Figure S25.** KDE plot of the O-X distance (in Å) versus the ∠Fe-O-X (in °) for the MD simulations of KDM6B-Fe(IV)=O•Lys(Me/Eth) system. X denotes carbons of the substrate.

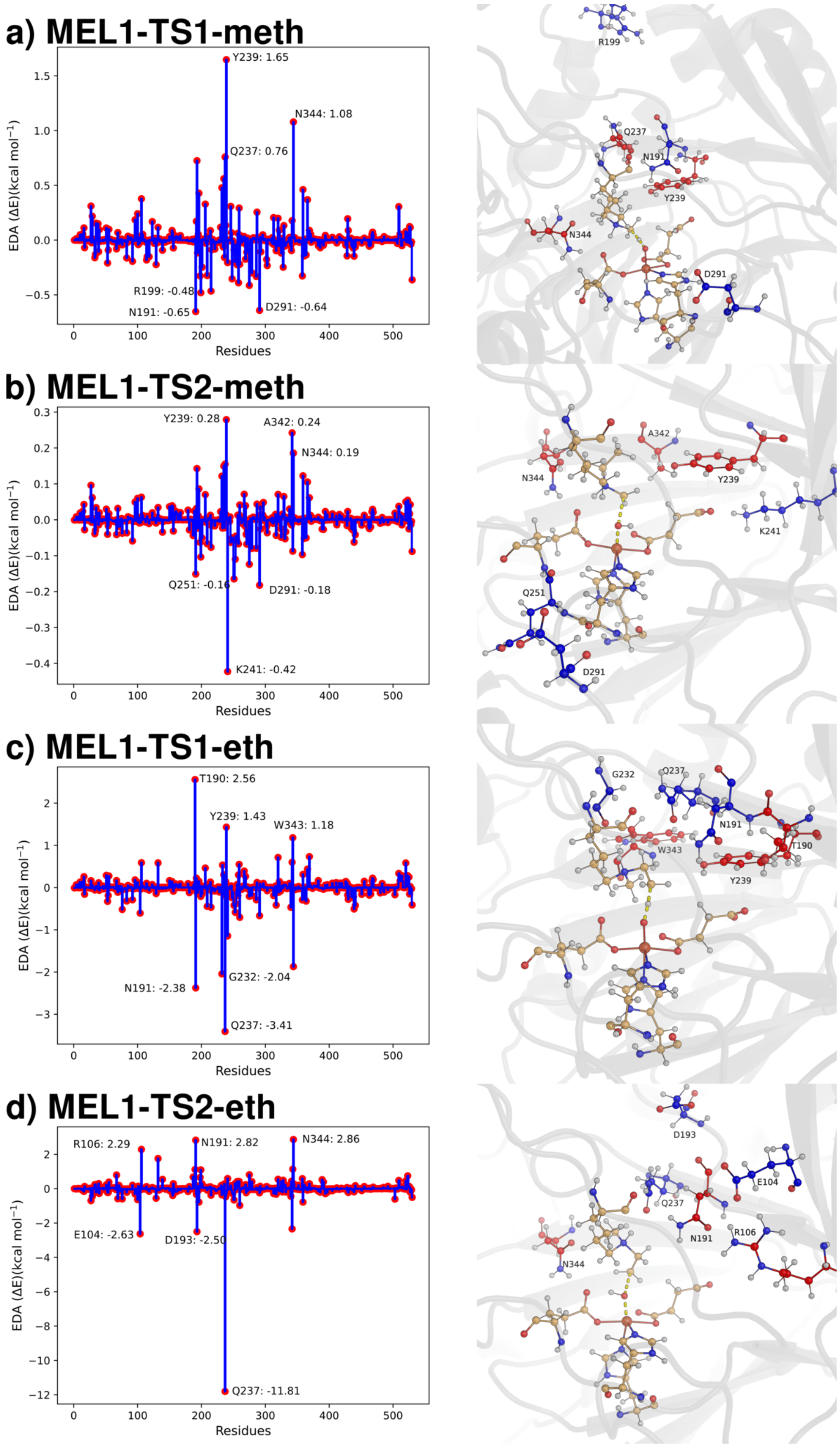


**Figure S26**. EDA analysis of HAT and rebound reactions of methyl (a and b) and ethyl (c and d) groups in MEL1-RC snapshot.

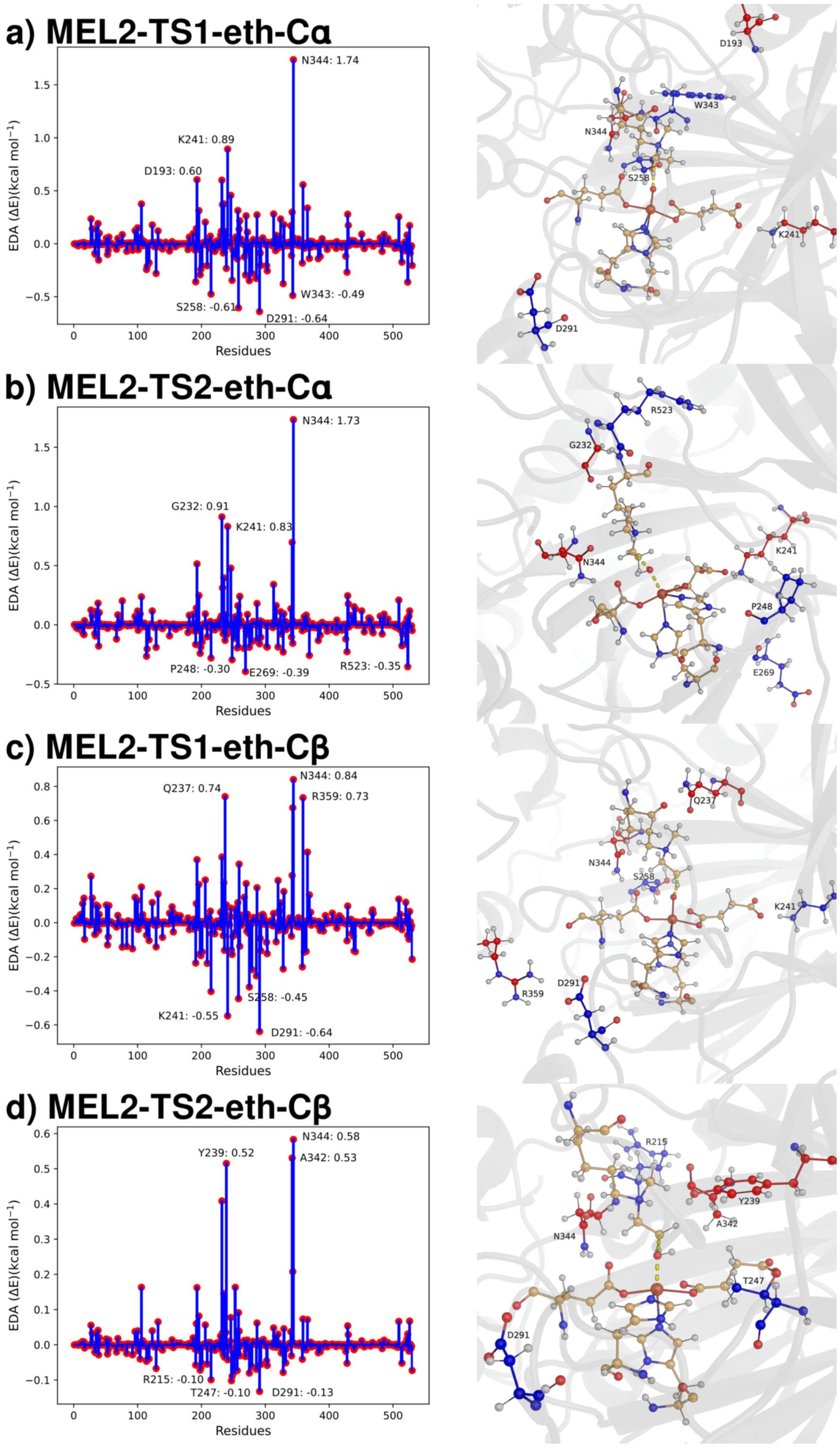


**Figure S27.** EDA analysis of HAT and rebound reactions of ethyl Cα (a and b) and ethyl Cβ (c and d) groups in the MEL2-RC snapshot.

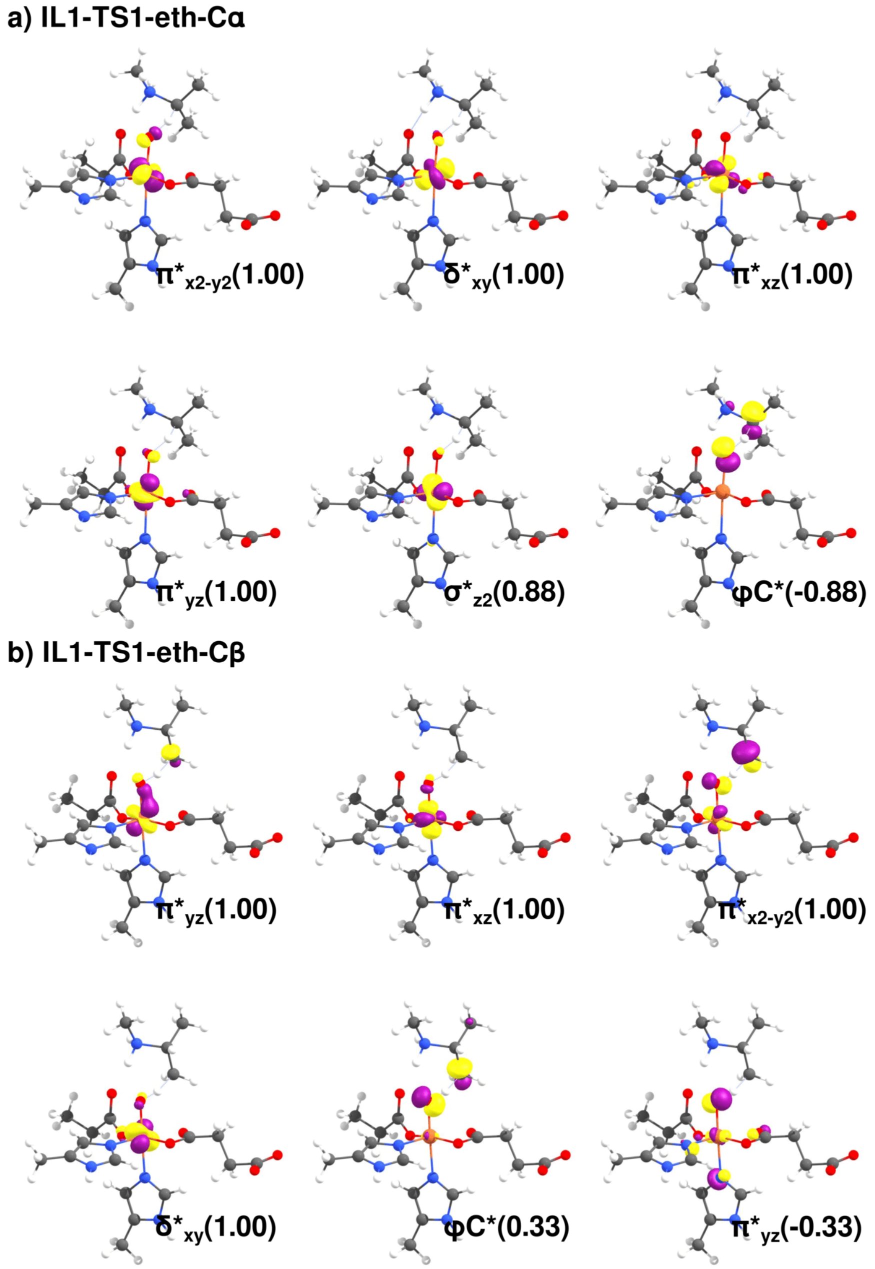


**Figure S28.** Spin Natural Orbital (SNO) analysis of HAT TSs in a) IL1-TS1-Cα, and b) IL1-TS1-Cβ.

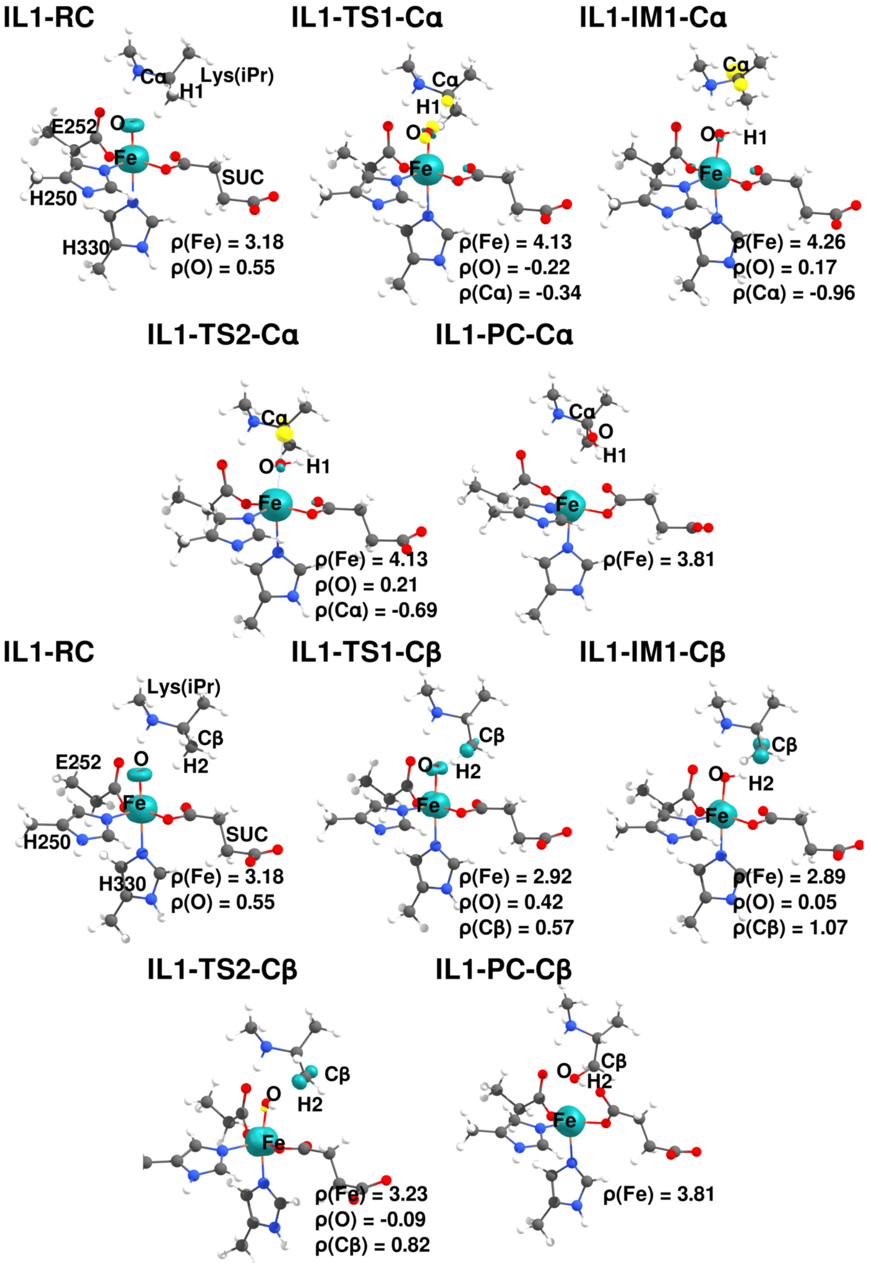


**Figure S29.** Spin density plots for the stationary points obtained during QM/MM simulations of the IL1-RC snapshot.

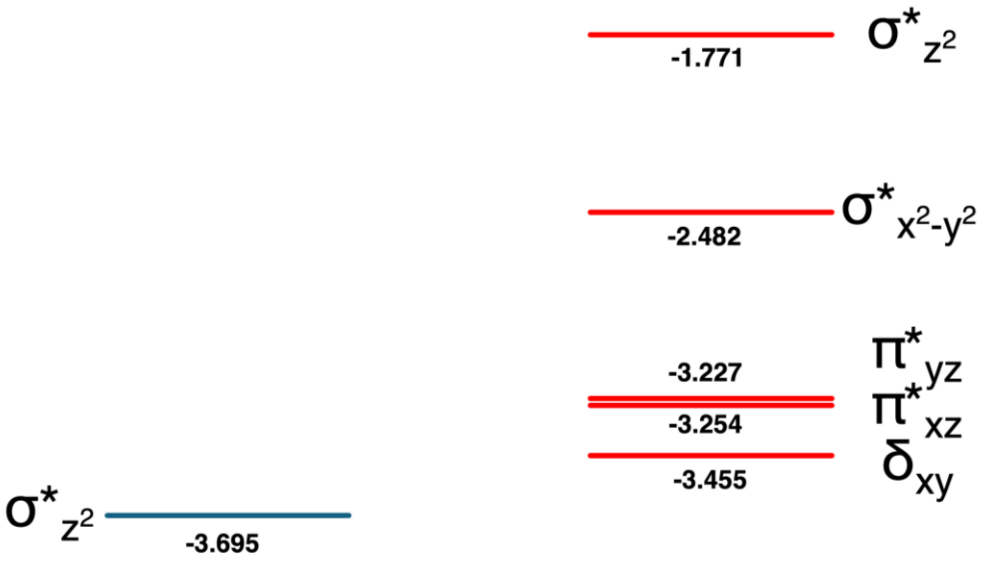


**Figure S30.** FMO for the HAT mechanism and their energies in the IL1-RC.

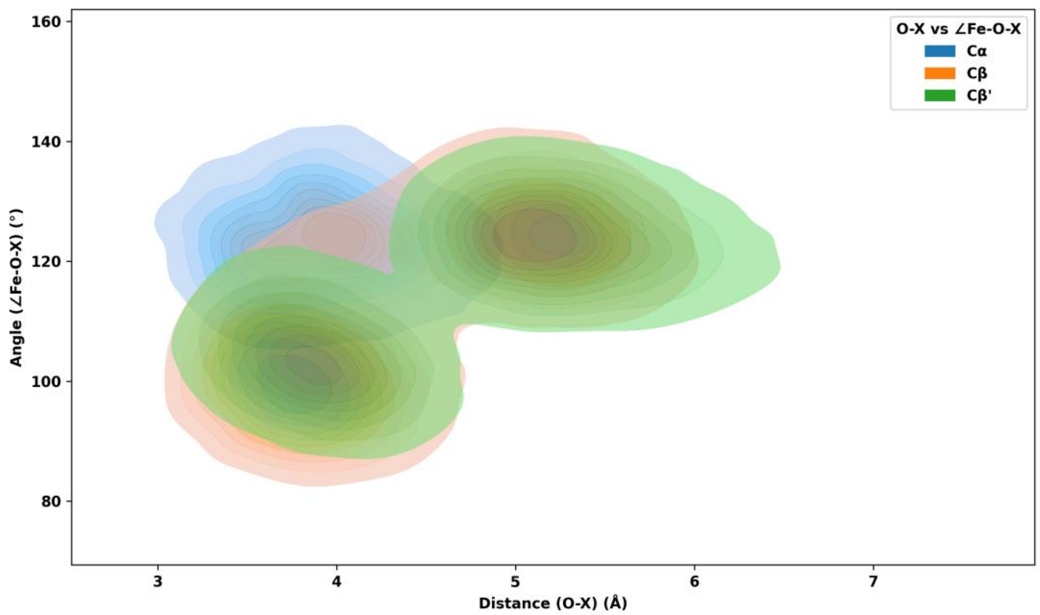


**Figure S31.** KDE plot of the O-X distance (in Å) versus the ∠Fe-O-X (in °) for the MD simulations of KDM6B-Fe(IV)=O•Lys(iPr) system. X denotes carbons of the substrate.

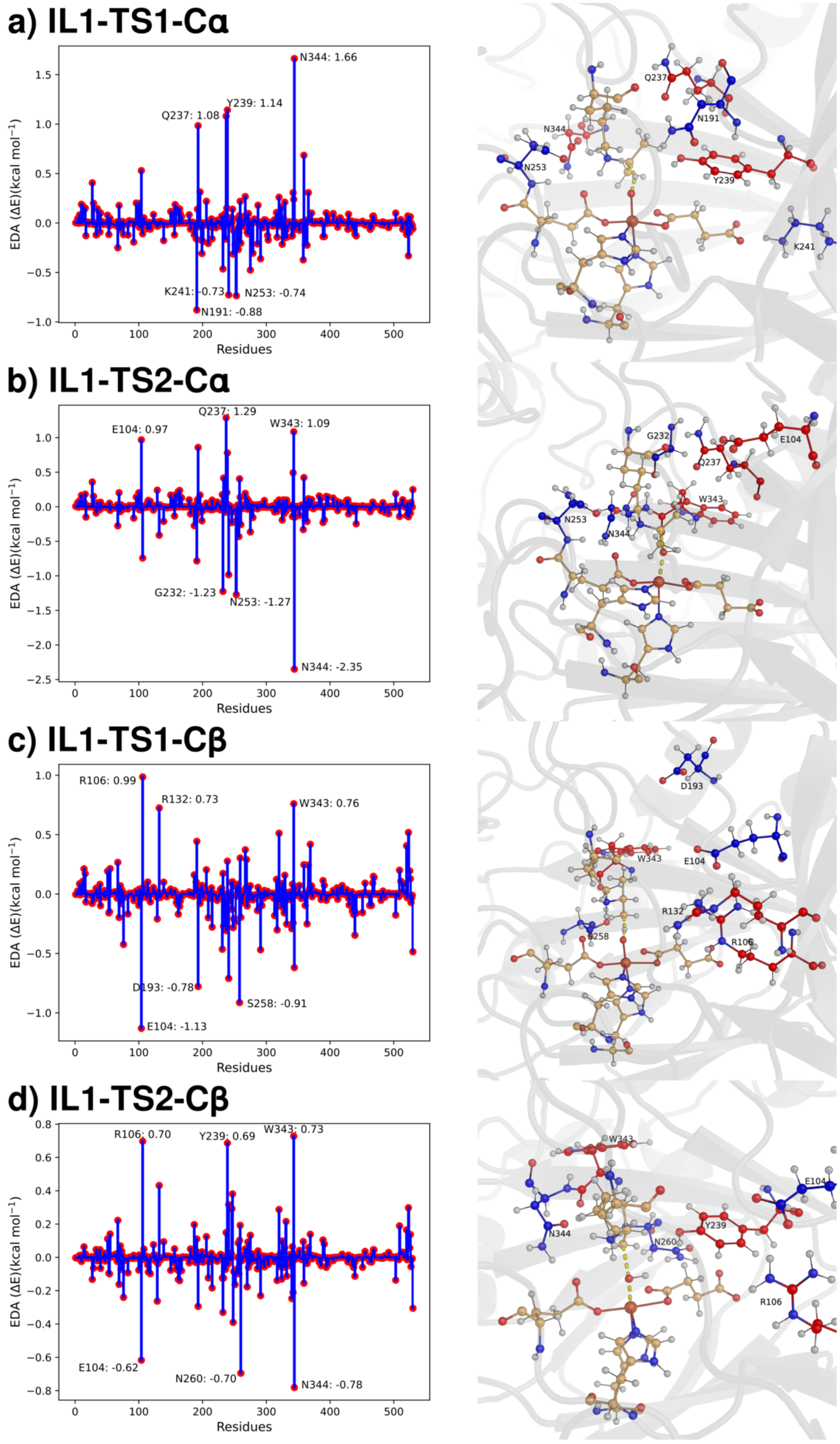


**Figure S32.** EDA analysis of HAT and rebound reactions of Cα (a and b) and Cβ (c and d) of the iPr group in the IL1-RC snapshot.

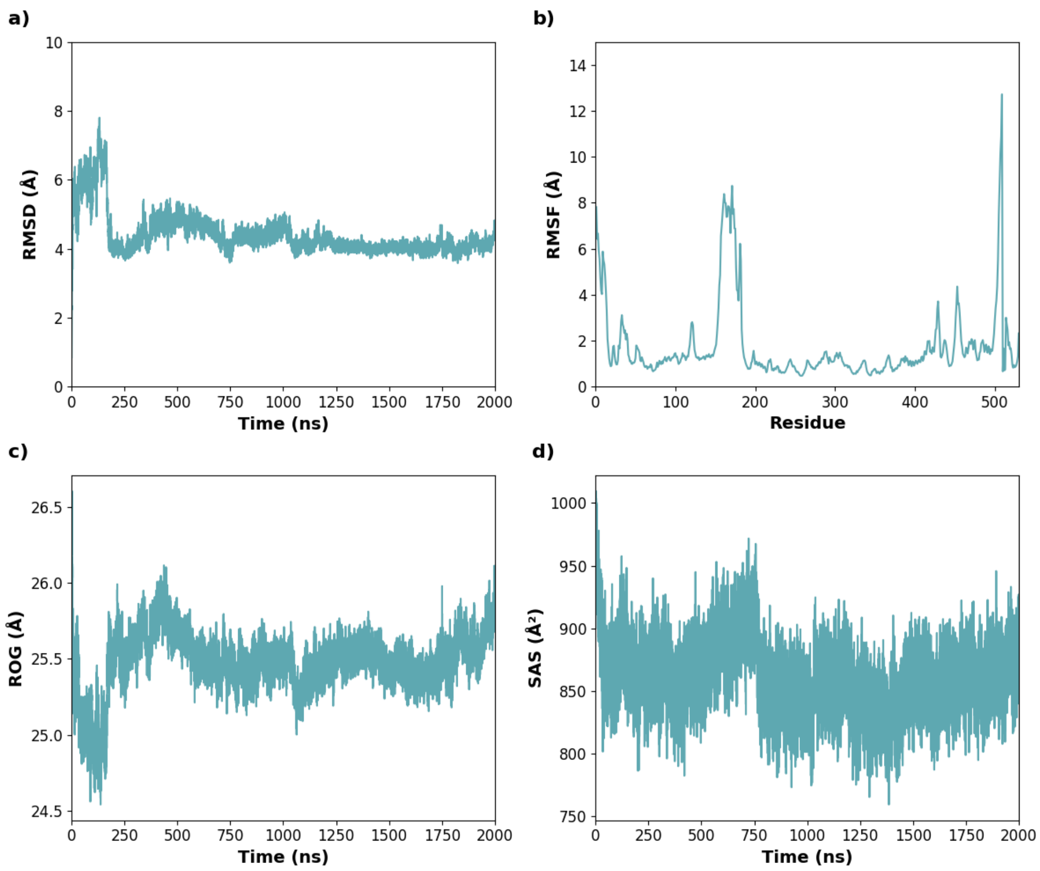


**Figure S33.** Conformational Dynamics of KDM6B-Fe(IV)=O•Lys(Me/Eth-OH) system. a) RMSD analysis of the dynamics suggests that the system is equilibrated, b) RMSF analysis of the system identifies flexible regions, c) ROG analysis shows the stability of the overall protein fold, and d) SAS analysis implies that the system is equilibrated.

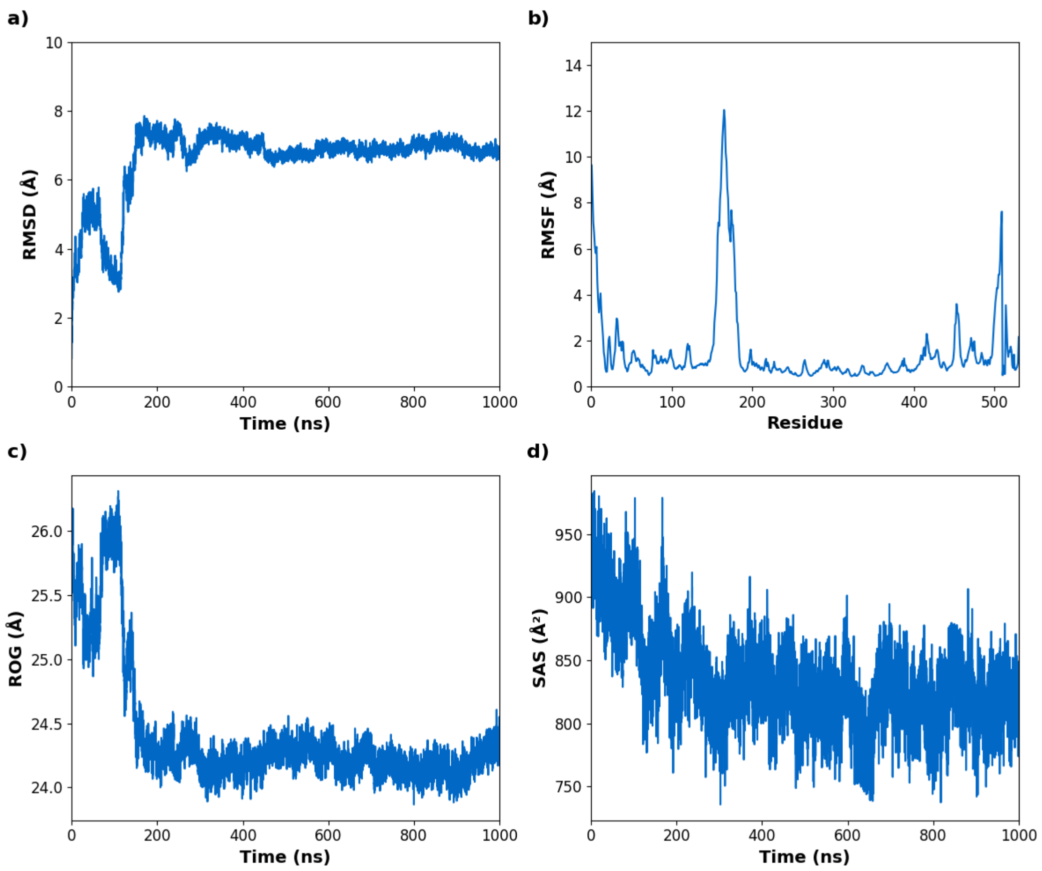


**Figure S34.** Conformational Dynamics of KDM6B-Fe(IV)=O•Lys(iPr-OH) system. a) RMSD analysis of the dynamics suggests that the system is equilibrated, b) RMSF analysis of the system identifies flexible regions, c) ROG analysis shows the stability of the overall protein fold, and d) SAS analysis implies that the system is equilibrated.

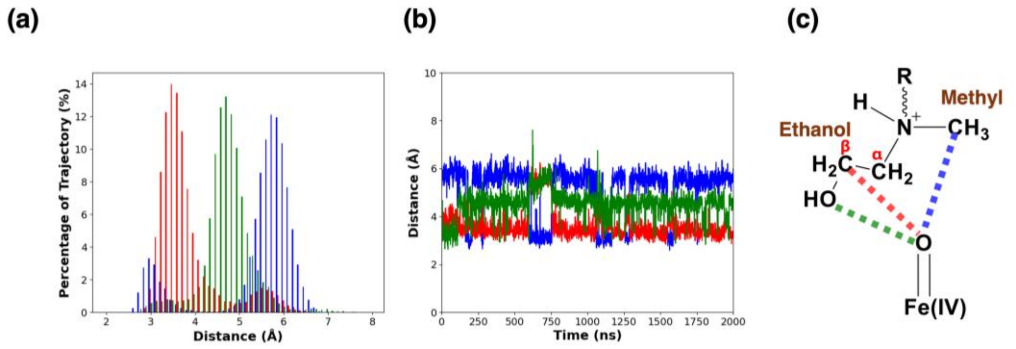


**Figure S35.** Substrate flexibility in KDM6B-Fe(IV)=O•Lys(Me/Eth-OH). a) Histogram plot of distance between the ferryl oxygen and different carbons and oxygen of the substrate, b) Evolution of distances over time, c) the color-coded distances depicted in (a).

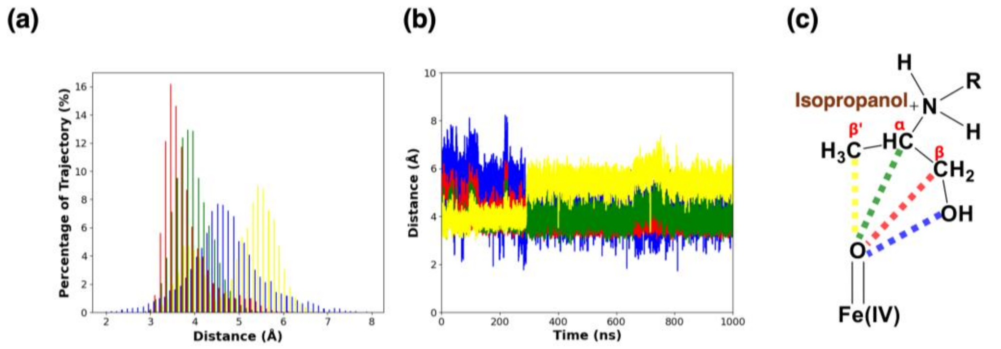


**Figure S36.** Substrate flexibility in KDM6B-Fe(IV)=O•Lys(iPr-OH). a) Histogram plot of distance between the ferryl oxygen and different carbons and oxygen of the substrate, b) Evolution of distances over time, c) the color-coded distances depicted in (a).

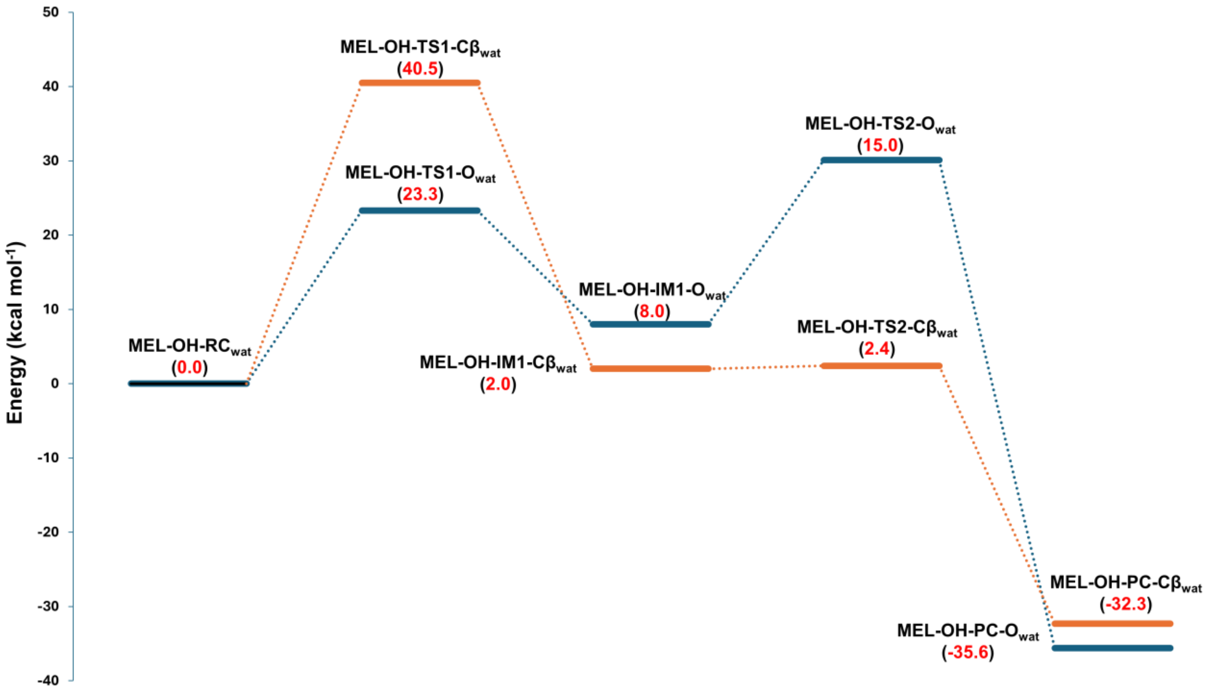


**Figure S37.** a) Reaction Profile of the oxidation of **Lys(Me/Eth-OH)** through water-mediated HAT. Relative energies are presented at the QM(B3)/MM level. The energy profile in orange shows the initiation of substrate oxidation by Cβ-HAT, and the energy profile in blue shows the initiation of substrate oxidation by O-HAT.

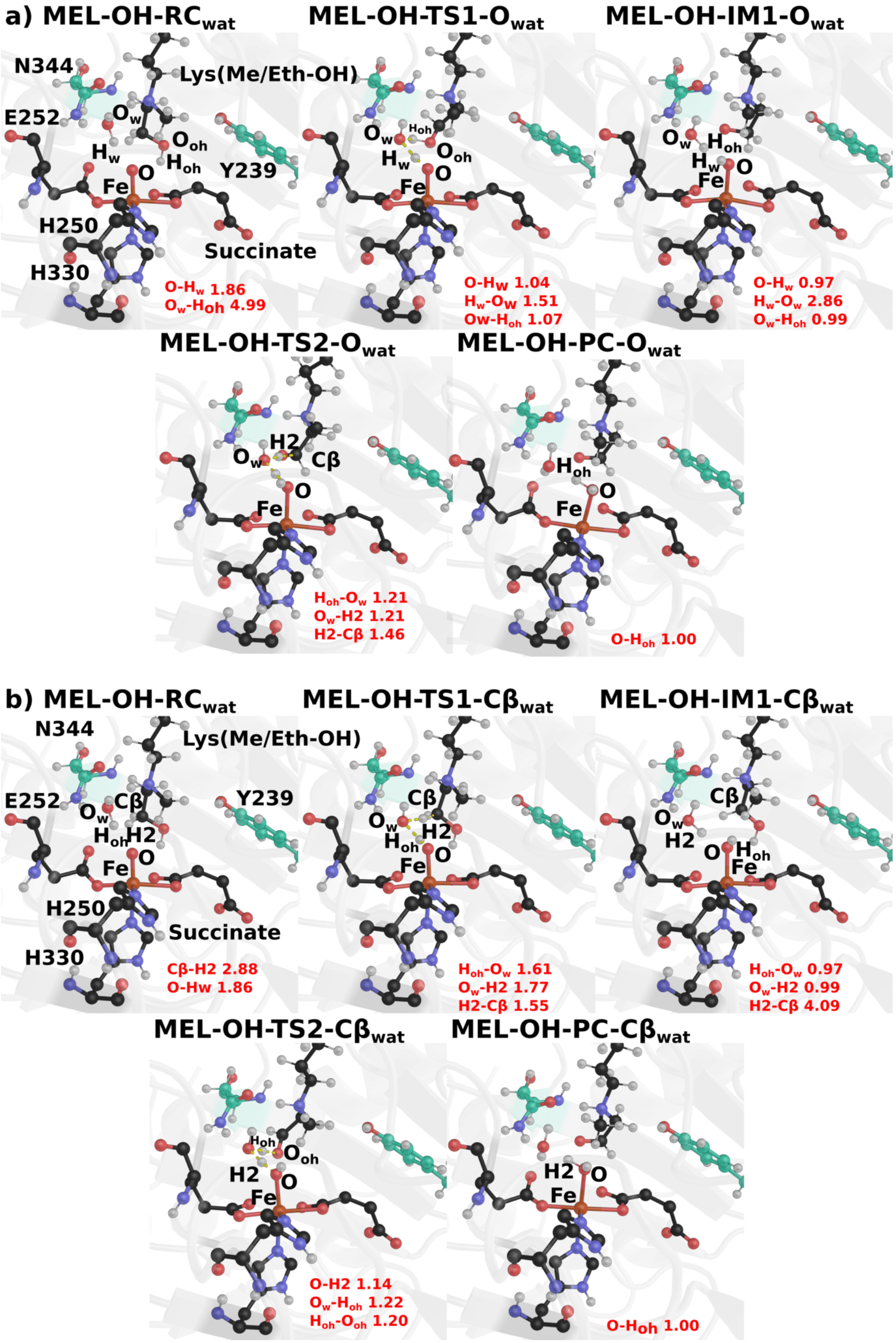


**Figure S38.** QM/MM optimized stationary points obtained during PES simulations from MEL1-OH-$RC_{wat}$ during hydroxylation of a) methyl, and b) ethyl Cβ carbons through water-mediated HAT. Distances are given in Å and represented by yellow dashed lines.

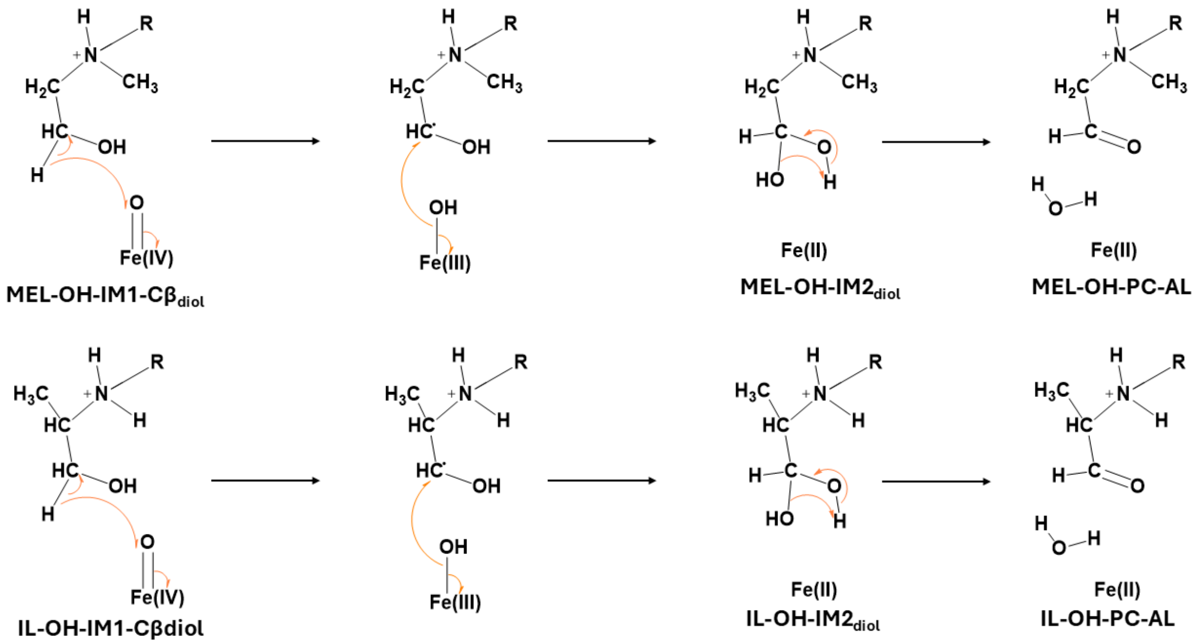


**Figure S39.** Mechanism of aldehyde formation through the gem-diol pathway.

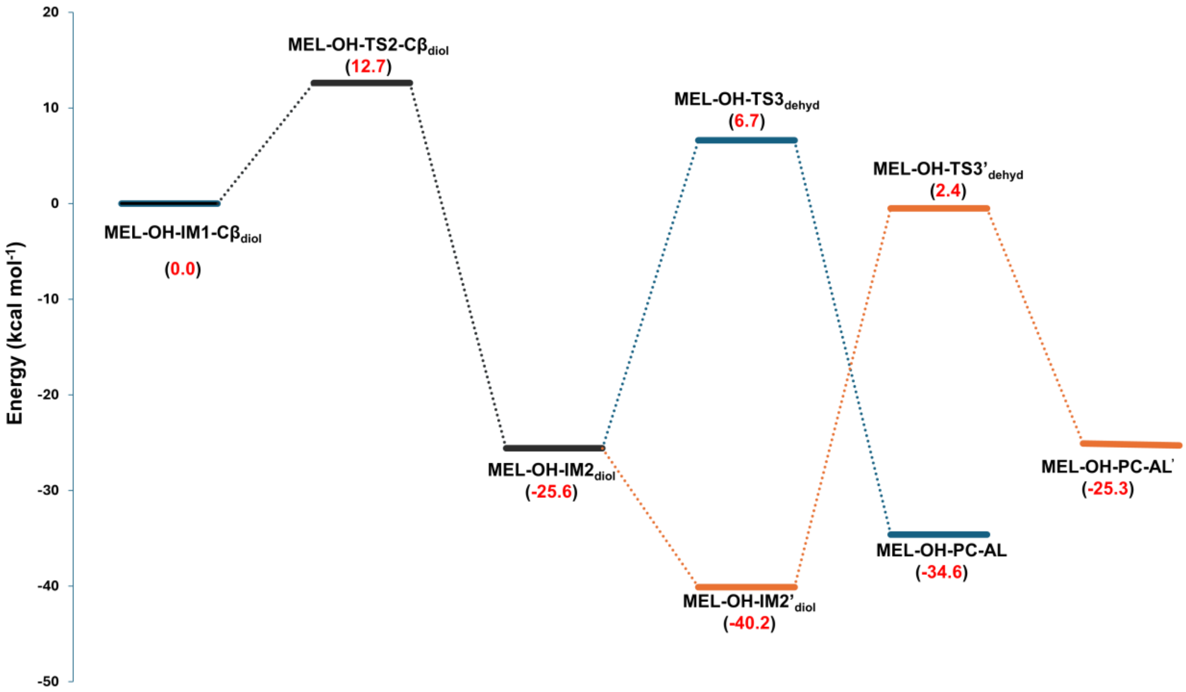


**Figure S40.** a) Reaction Profile of the oxidation of **Lys(Me/Eth-OH)** through gem-diol pathway. Relative energies are presented at the QM(B3)/MM level. Reaction profile bifurcation in blue and orange shows different dehydration possibilities.

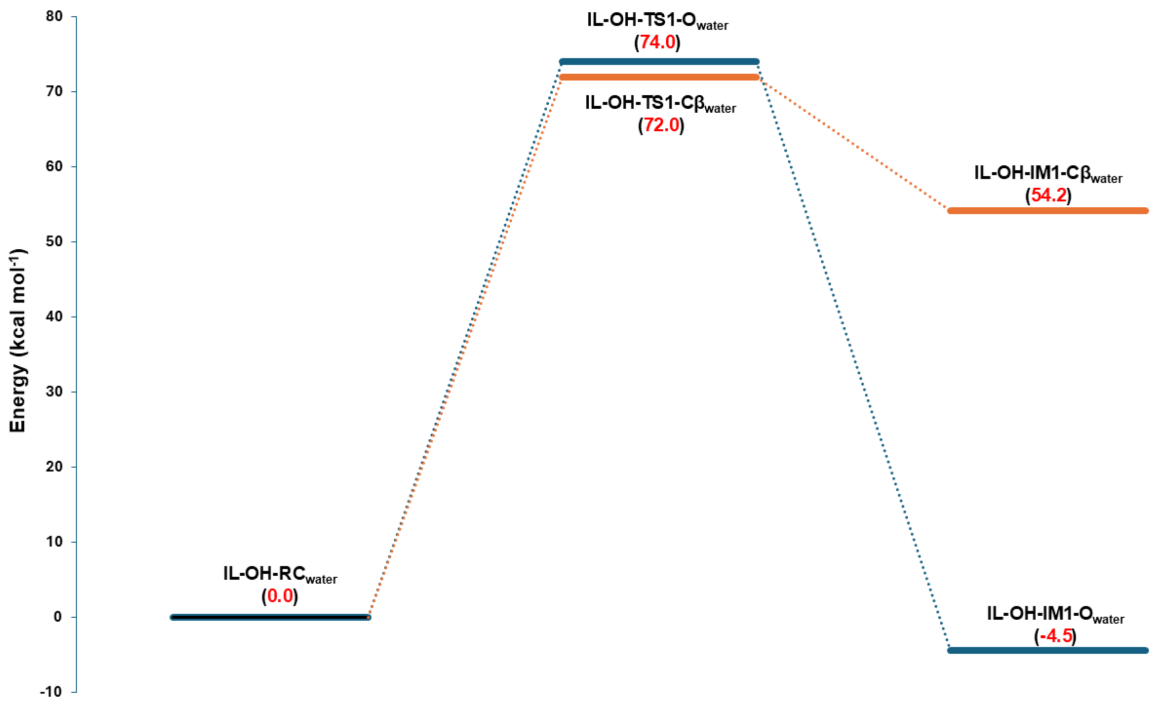


**Figure S41.** a) Reaction Profile of the oxidation of **Lys(IL-OH)** through water-mediated HAT. Relative energies are presented at the QM(B3)/MM level. The energy profile in orange shows the initiation of substrate oxidation by Cβ-HAT, and the energy profile in blue shows the initiation of substrate oxidation by O-HAT.

**a) MEL-OH-TS1-O**

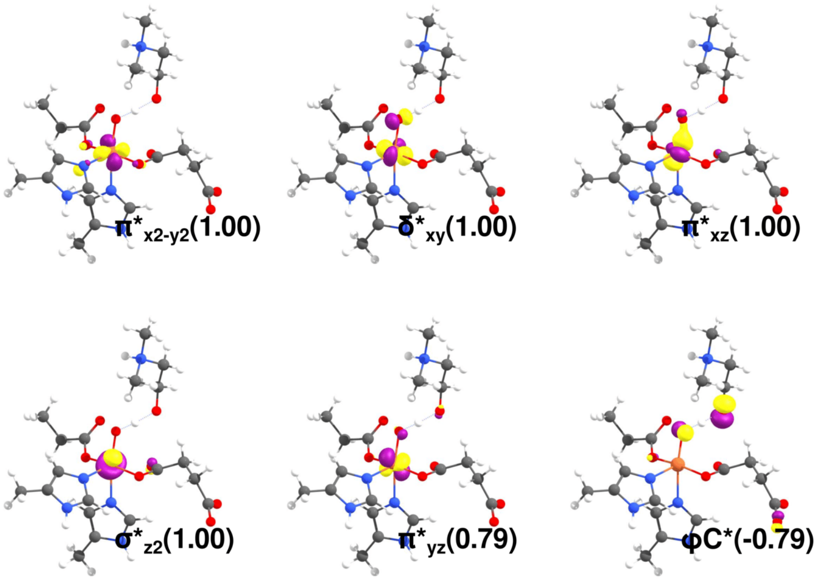


**b) MEL-OH-TS1-Cβ**

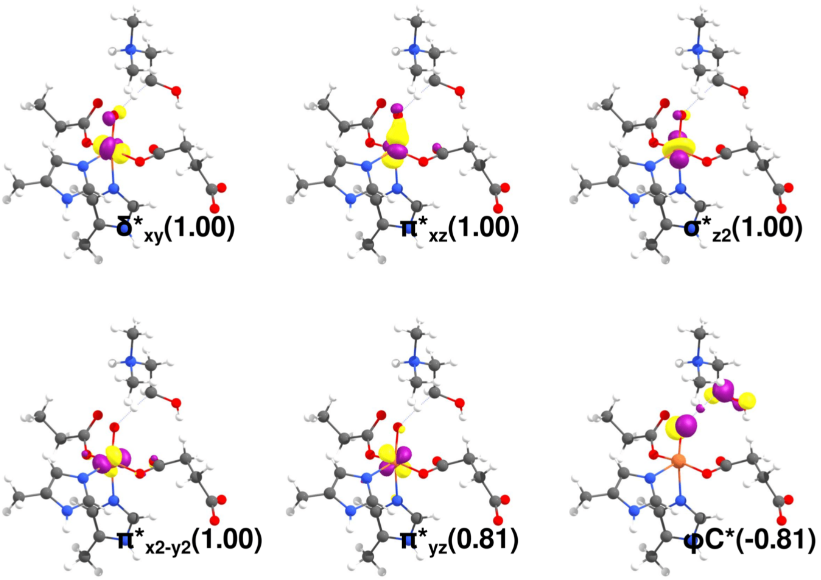


**Figure S42.** Spin Natural Orbital (SNO) analysis of HAT TSs in a) MEL-OH-TS1-O, and b) MEL-OH-TS1-Cβ.

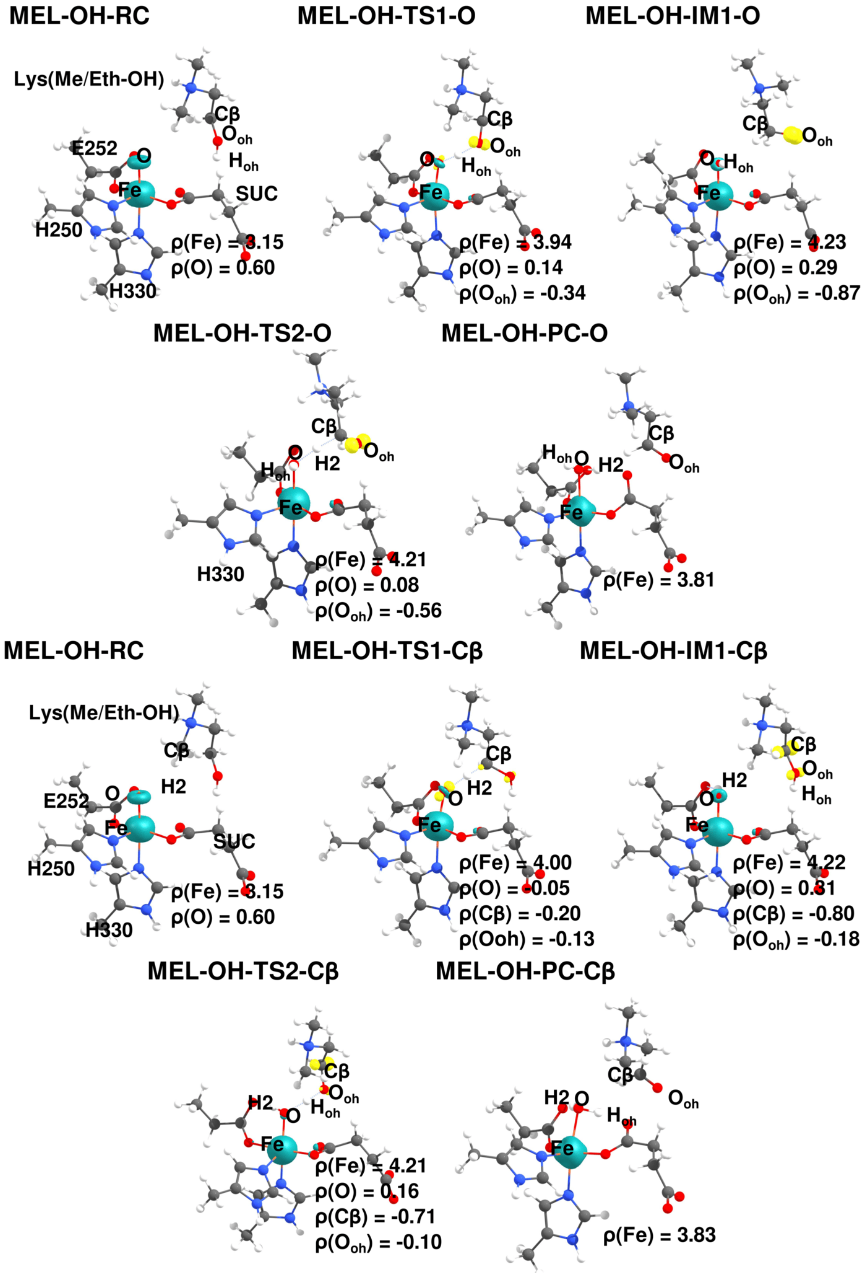


**Figure S43.** Spin density plots for the stationary points obtained during QM/MM simulations of the MEL-OH-RC snapshot.

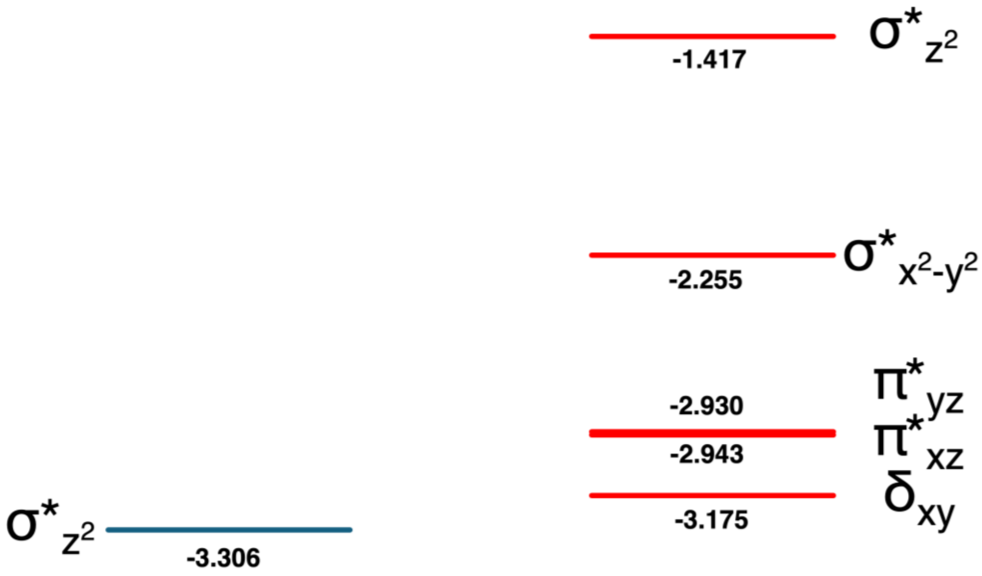


**Figure S44.** FMO for the HAT mechanism and their energies in the MEL-OH-RC.

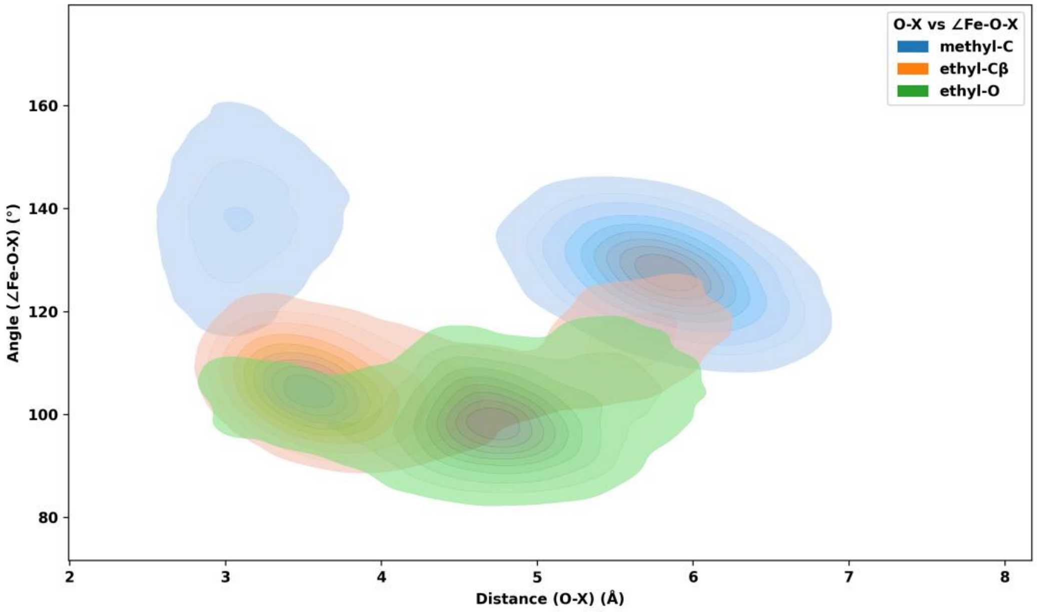


**Figure S45.** KDE plot of the O-X distance (in Å) versus the ∠Fe-O-X (in °) for the MD simulations of the KDM6B-Fe(IV)=O•Lys(Me/Eth-OH) system. X denotes carbons and oxygen of the substrate.

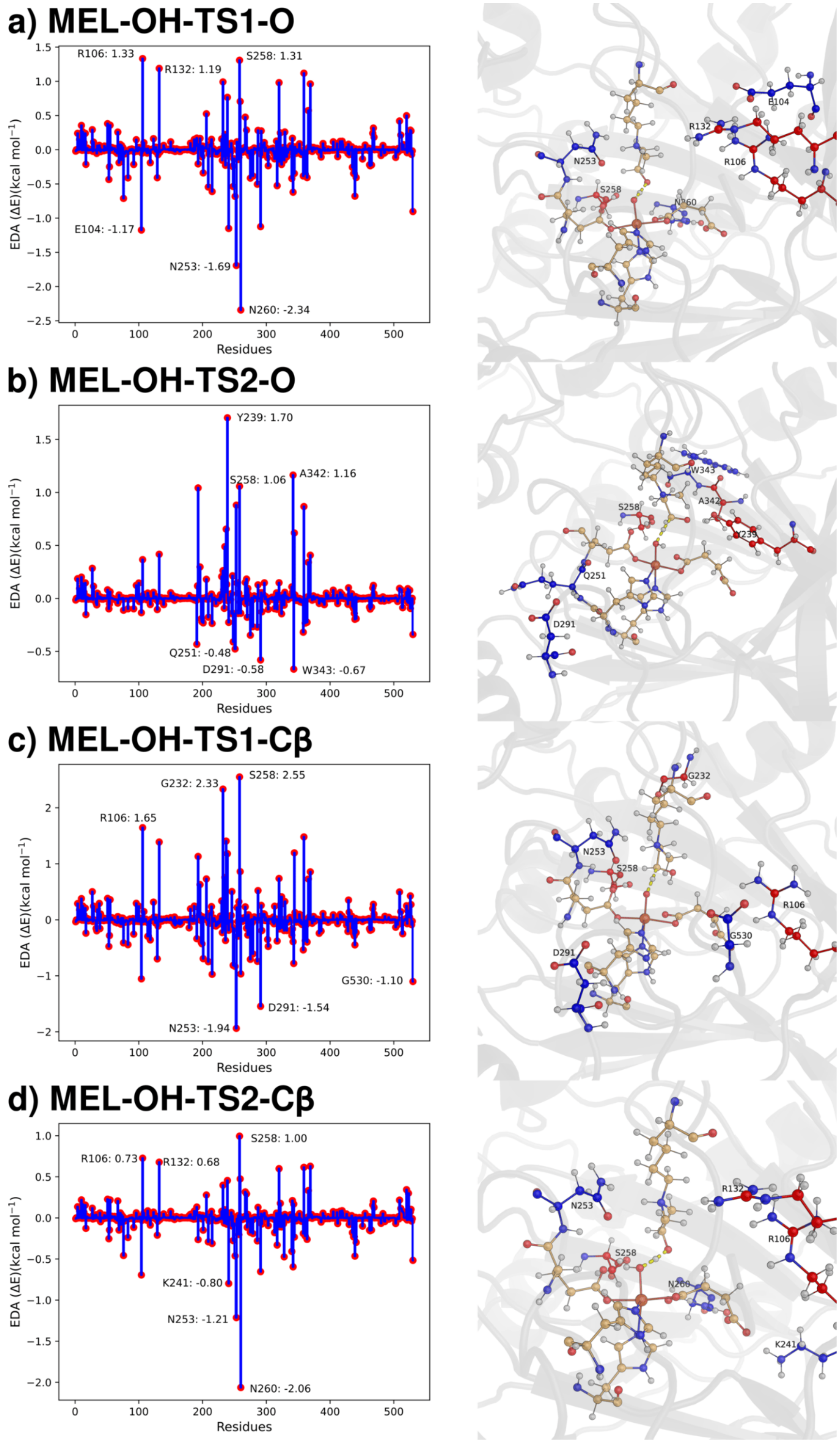


**Figure S46.** EDA analysis of HAT reactions initiated from $O_{oh}$ (a and b) and Cβ (c and d) of the Eth-OH group in MEL-OH-RC snapshot.

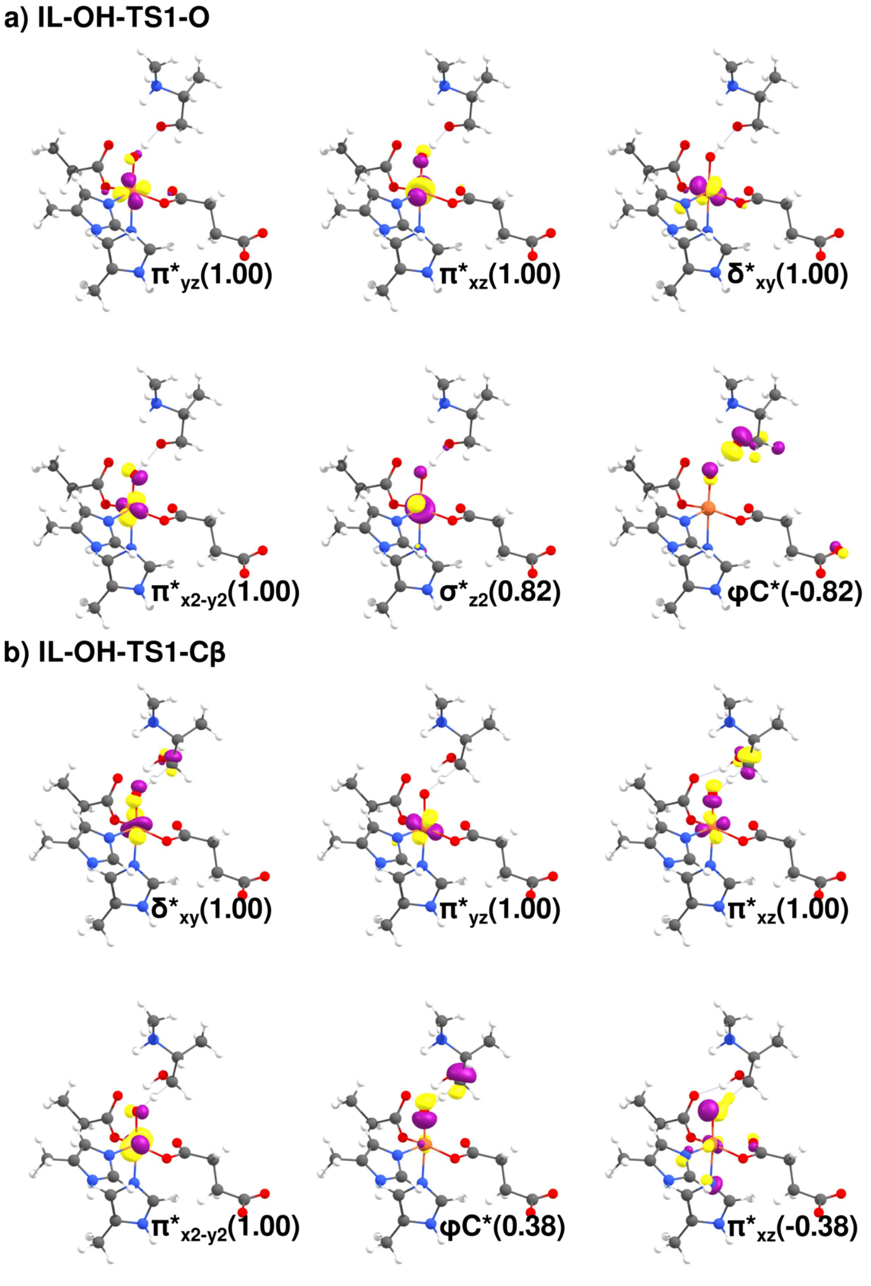


**Figure S47.** Spin Natural Orbital (SNO) analysis of HAT TSs in a) IL-OH-TS1-O, and b) IL-OH-TS1-Cβ.

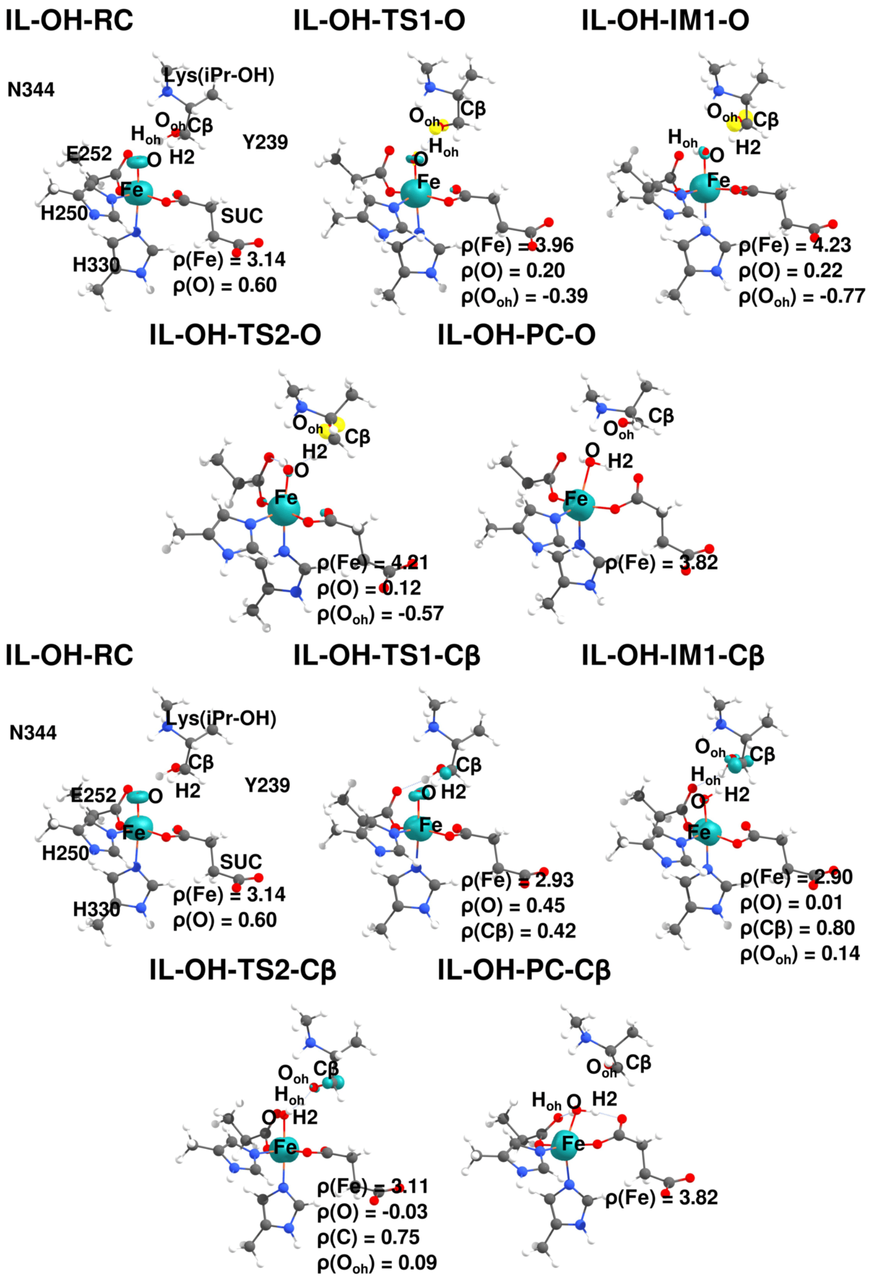


**Figure S48.** Spin density plots for the stationary points obtained during QM/MM simulations of the IL-OH-RC snapshot.

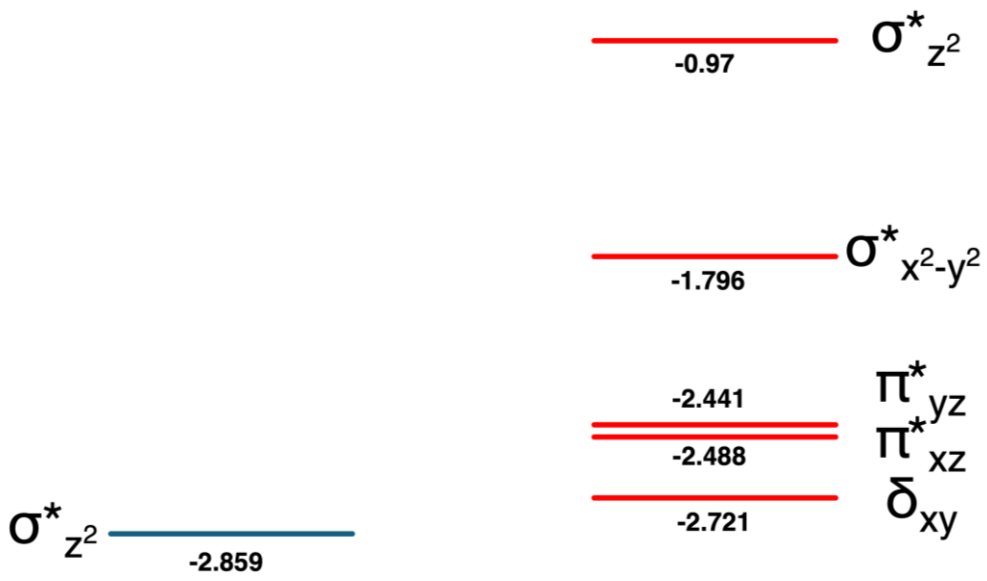


**Figure S49.** FMO for the HAT mechanism and their energies in the IL-OH-RC.

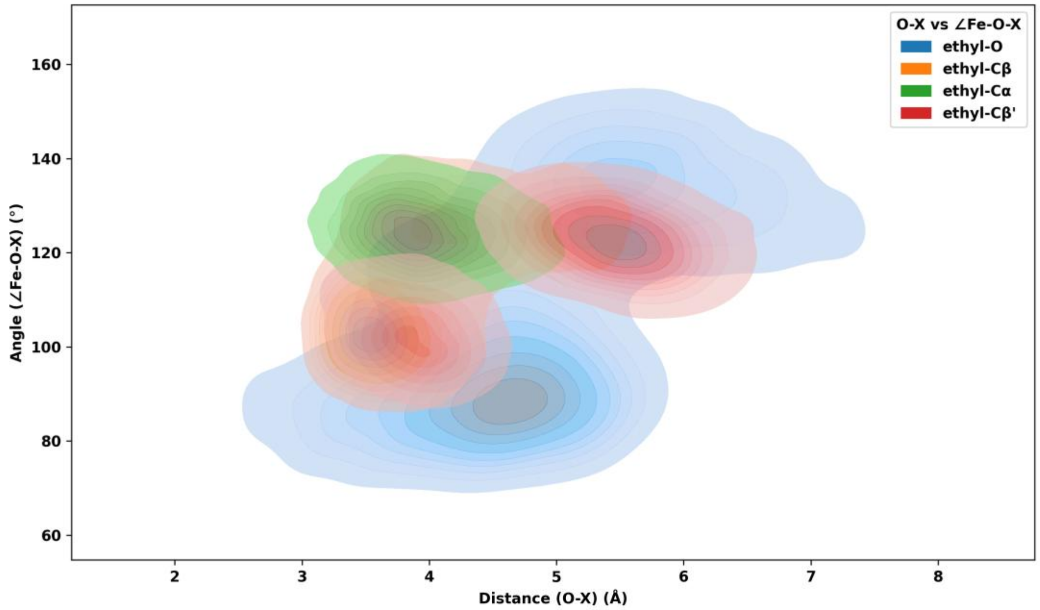


**Figure S50.** KDE plot of the O-X distance (in Å) versus the ∠Fe-O-X (in °) for the MD simulations of KDM6B-Fe(IV)=O•Lys(iPr-OH) system. X denotes carbons and oxygen of the substrate.

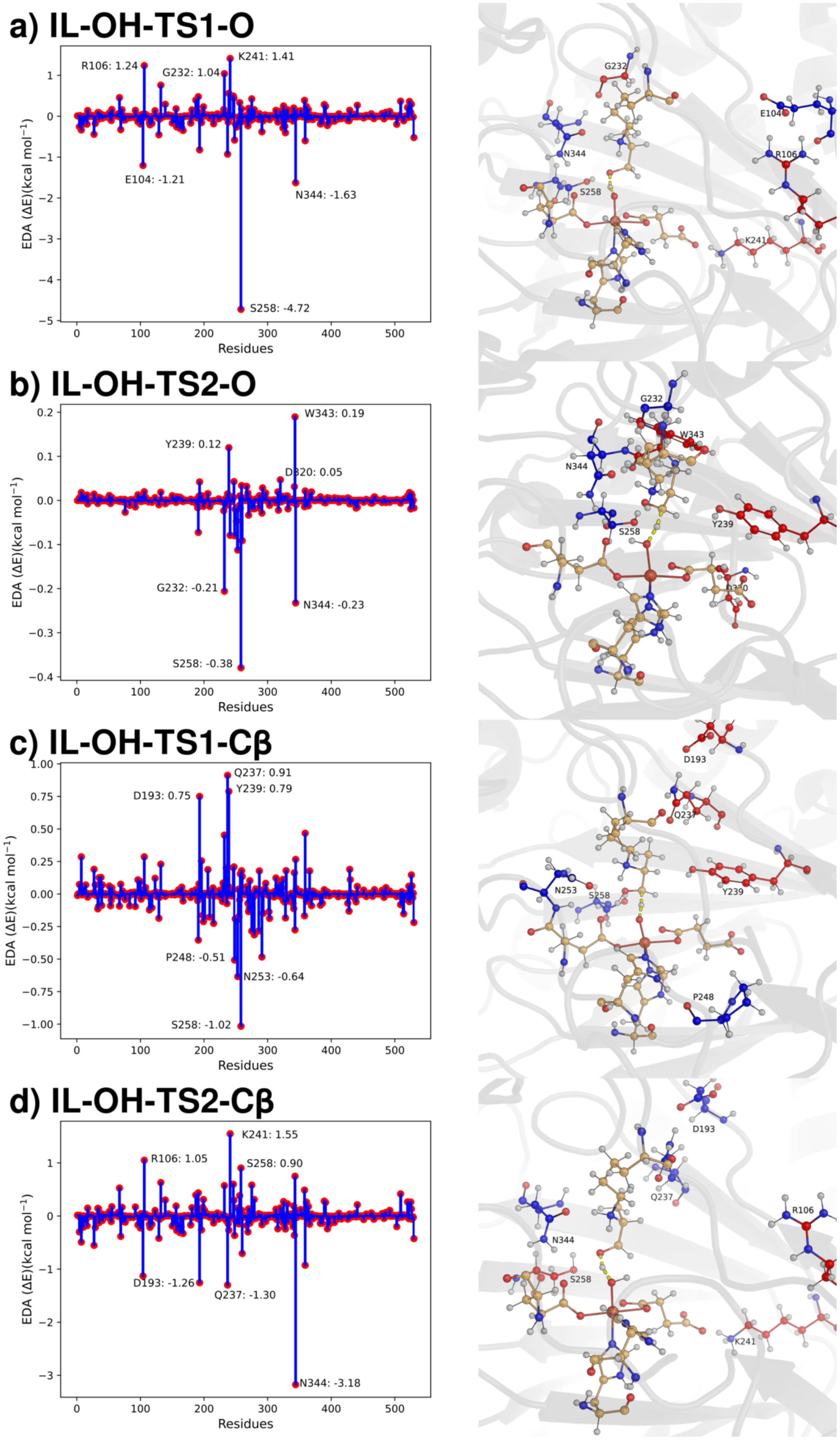


**Figure S51.** EDA analysis of HAT reactions initiated from $O_{oh}$ (a and b) and Cβ (c and d) of the iPr-OH group of the IL-OH-RC snapshot.

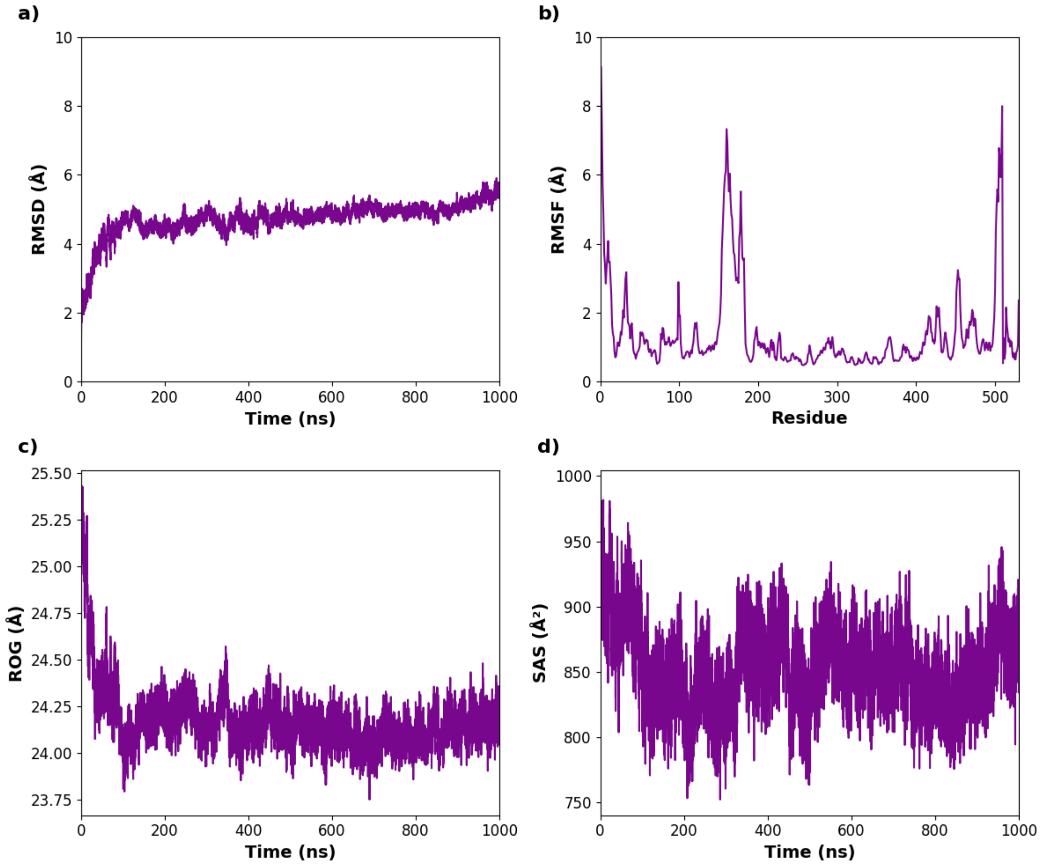


**Figure S52.** Conformational Dynamics of the KDM6B-Fe(IV)=O•Lys(Me/Eth-AL) system. a) RMSD analysis of the dynamics suggests that the system is equilibrated, b) RMSF analysis of the system identifies flexible regions, c) ROG analysis shows the stability of the overall protein fold, and d) SAS analysis implies that the system is equilibrated.

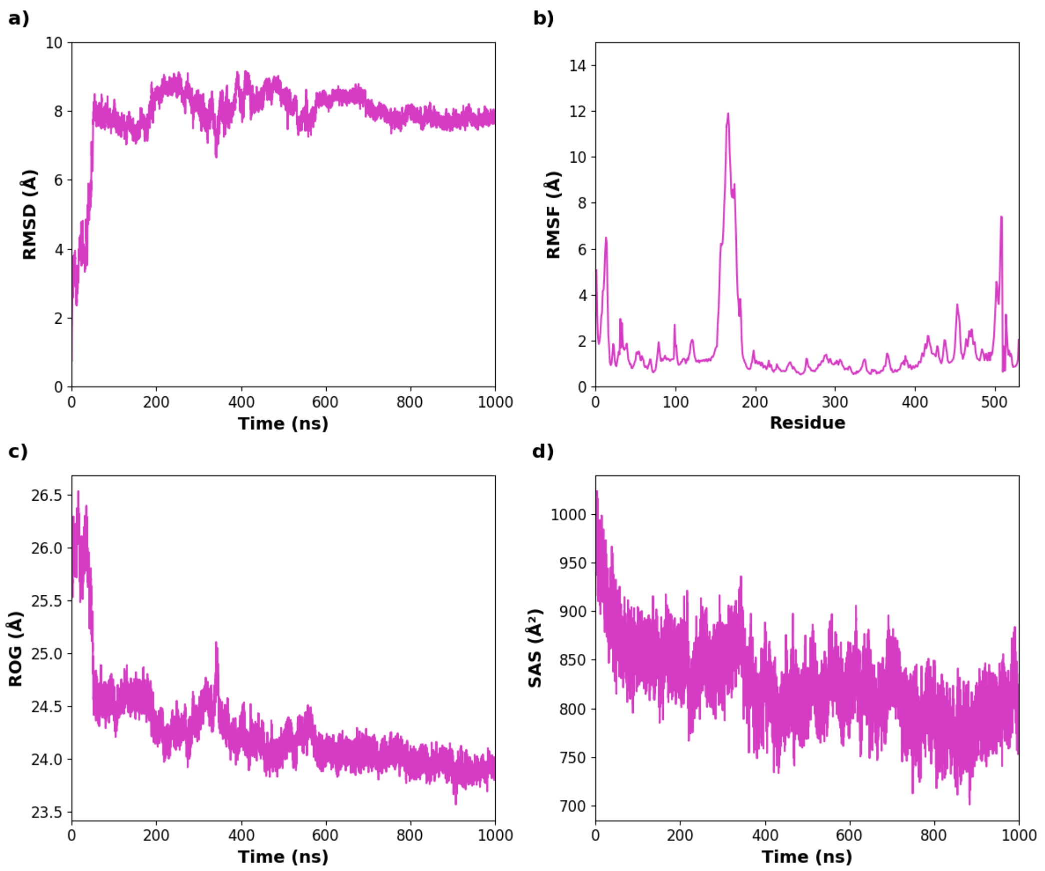


**Figure S53.** Conformational Dynamics of the KDM6B-Fe(IV)=O•Lys(iPr-AL) system. a) RMSD analysis of the dynamics suggests that the system is equilibrated, b) RMSF analysis of the system identifies flexible regions, c) ROG analysis shows the stability of the overall protein fold, and d) SAS analysis implies that the system is equilibrated.

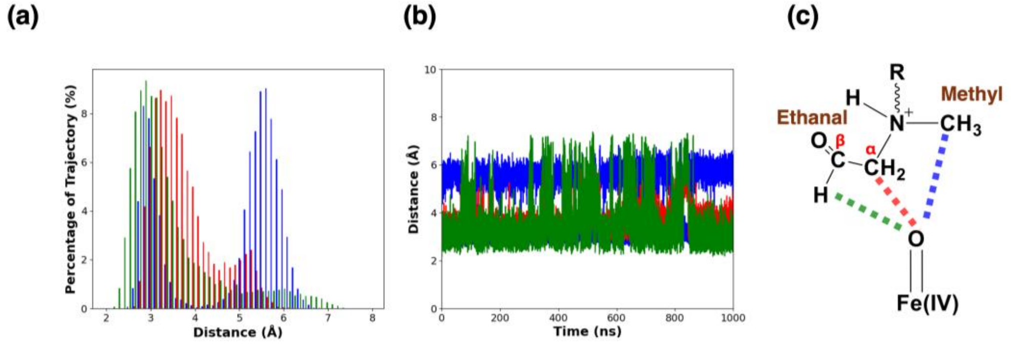


**Figure S54.** Substrate flexibility in KDM6B-Fe(IV)=O•Lys(Me/Eth-AL). a) Histogram plot of the distance between the ferryl oxygen and different carbons and hydrogen of the substrate, b) Evolution of distances over time, c) ChemDraw image showing the color-coded distances depicted in (a).

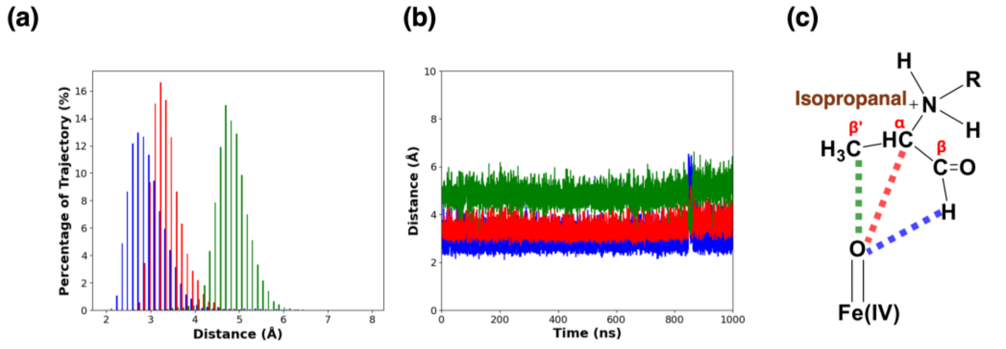


**Figure S55.** Substrate flexibility in KDM6B-Fe(IV)=O•Lys(iPr-AL). a) Histogram plot of the distance between the ferryl oxygen and different carbons and hydrogen of the substrate, b) Evolution of distances over time, c) the color-coded distances depicted in (a).

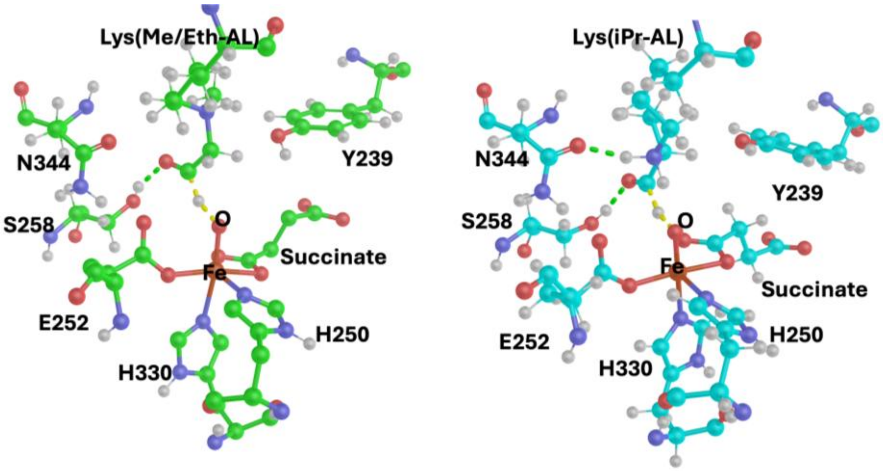


**Figure S56.** Hydrogen bonding interactions around the active in oxidation of Lys(Me/Eth-AL) (left) and Lys(iPr-AL) (right).

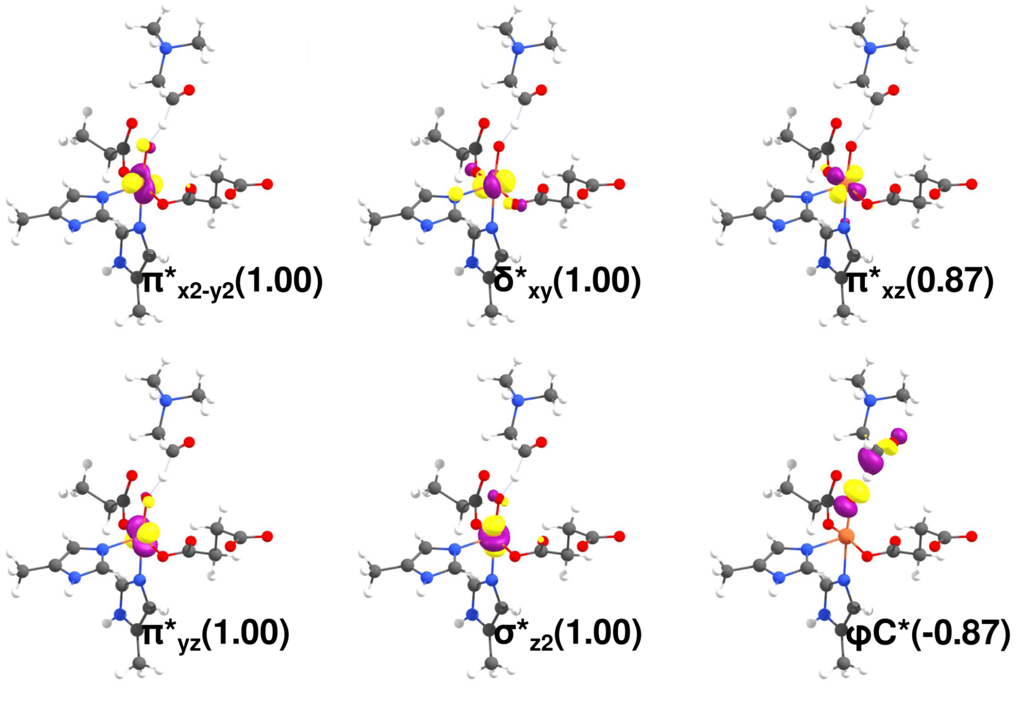


**Figure S57.** Spin Natural Orbital (SNO) analysis of HAT TSs in MEL-AL-TS1

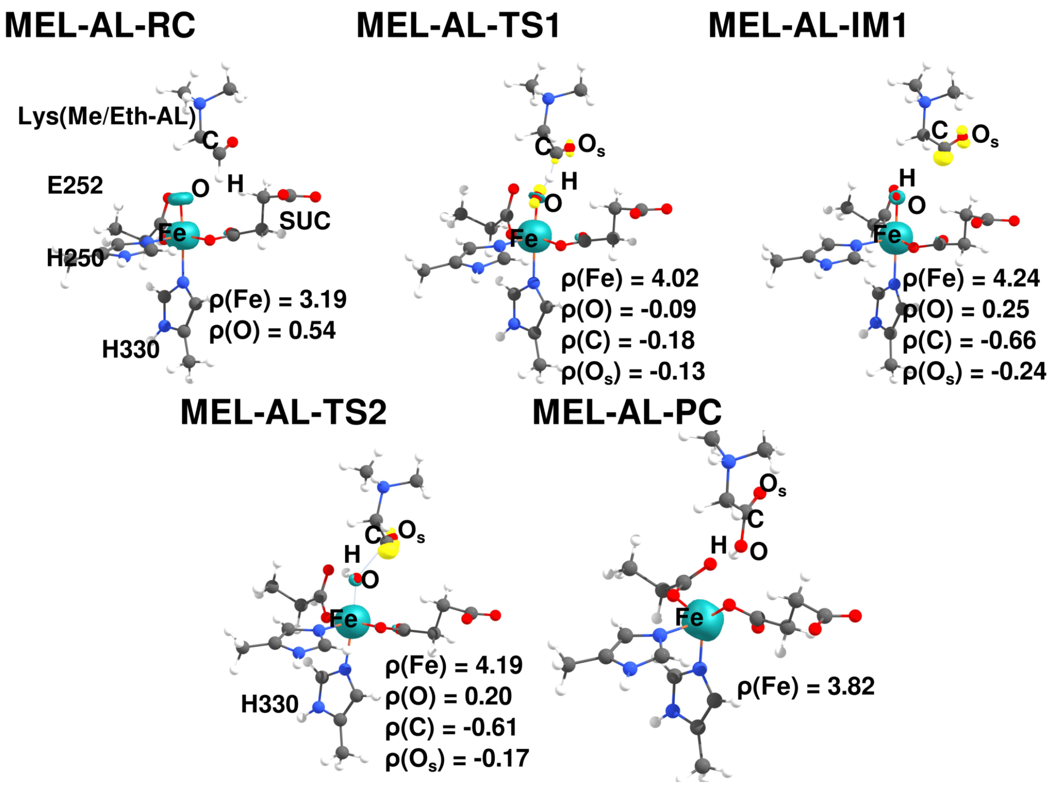


**Figure S58.** Spin density plots for the stationary points obtained during QM/MM simulations of the MEL-AL-RC snapshot.

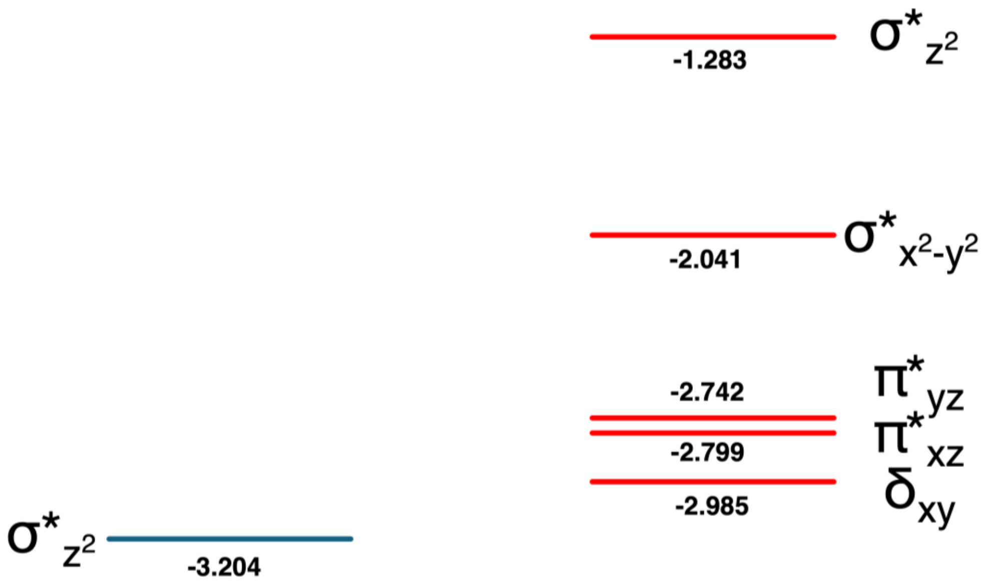


**Figure S59.** FMO for the HAT mechanism and their energies in the MEL-AL-RC.

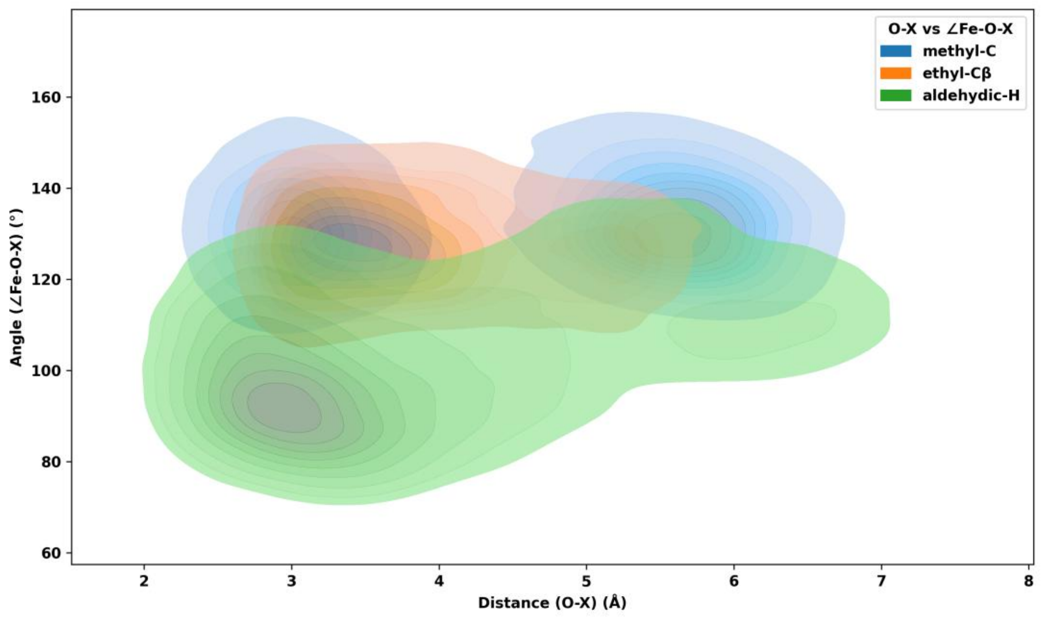


**Figure S60.** KDE plot of the O-X distance (in Å) versus the ∠Fe-O-X (in °) for the MD simulations of the KDM6B-Fe(IV)=O•Lys(Me/Eth-AL) system. X denotes carbons and hydrogen of the substrate.

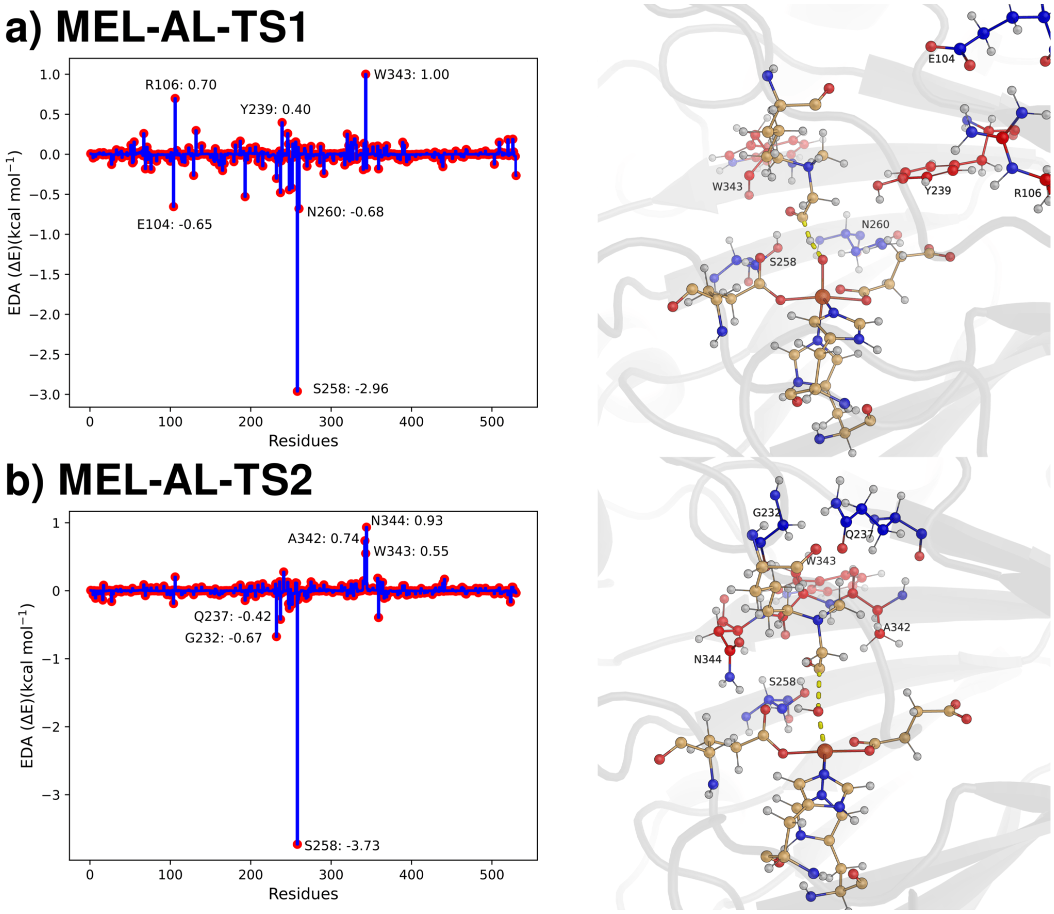


**Figure S61.** EDA analysis of the HAT and rebound reactions of MEL-AL group in the MEL-AL-RC snapshot.

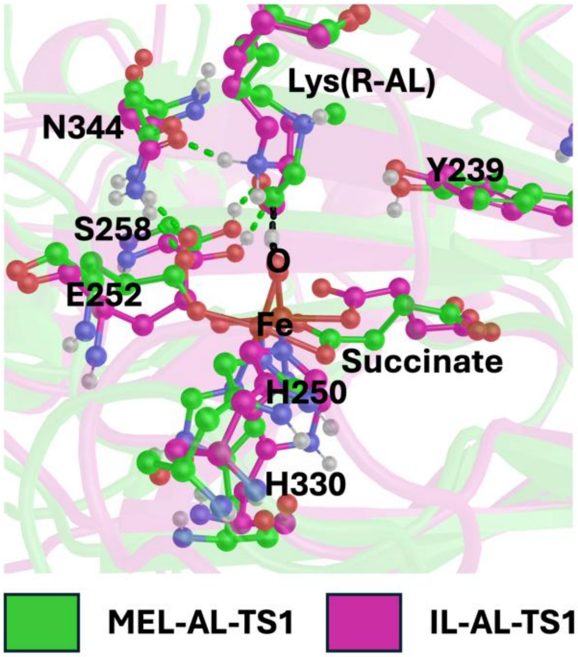


**Figure S62.** Overlaid TSs of the HAT from Lys(Me/Eth-AL) and Lys(iPr-AL).

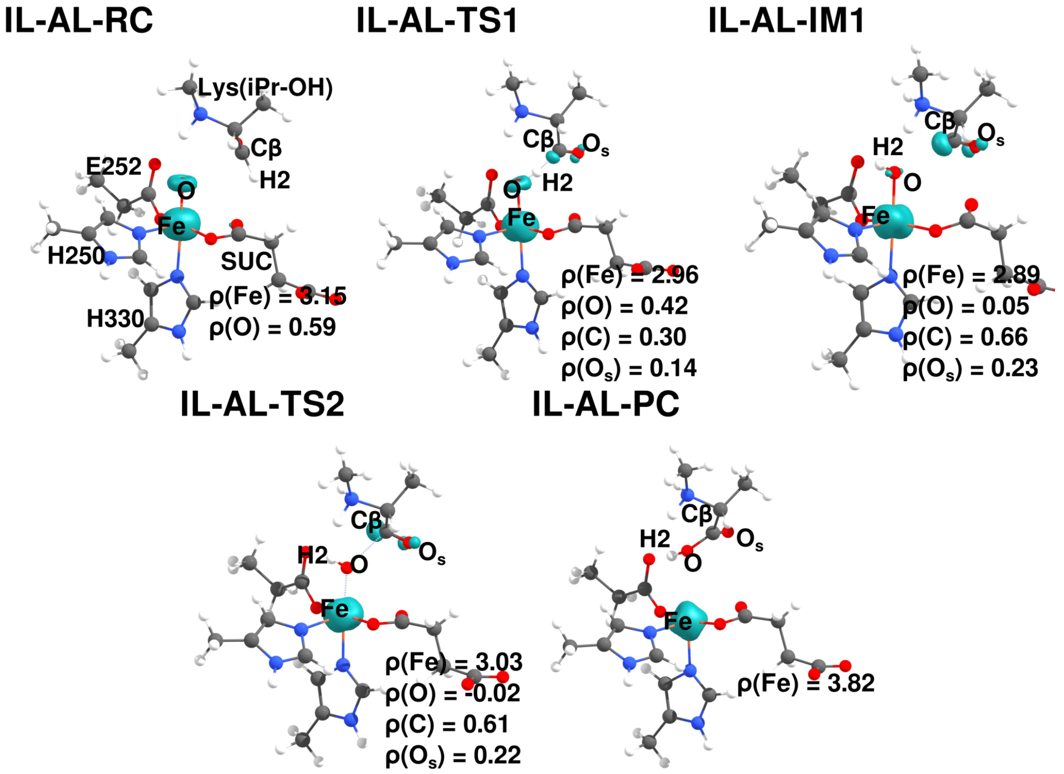


**Figure S63.** Spin density plots for the stationary points obtained during QM/MM simulations of the IL-AL-RC snapshot.

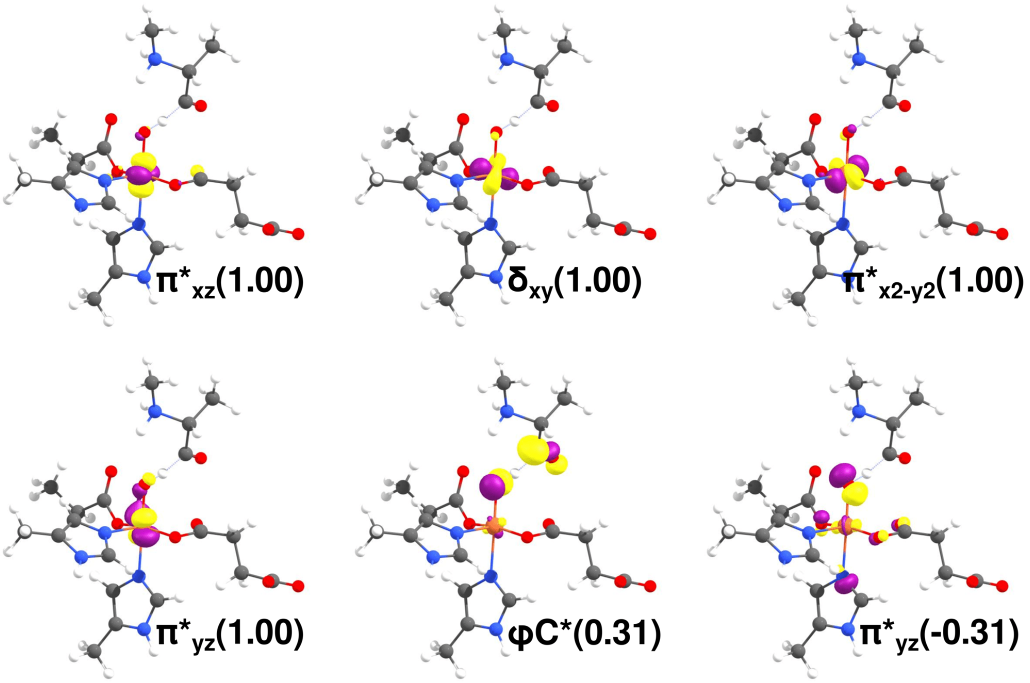


**Figure S64.** Spin Natural Orbital (SNO) analysis of HAT TSs in IL-AL-TS1.

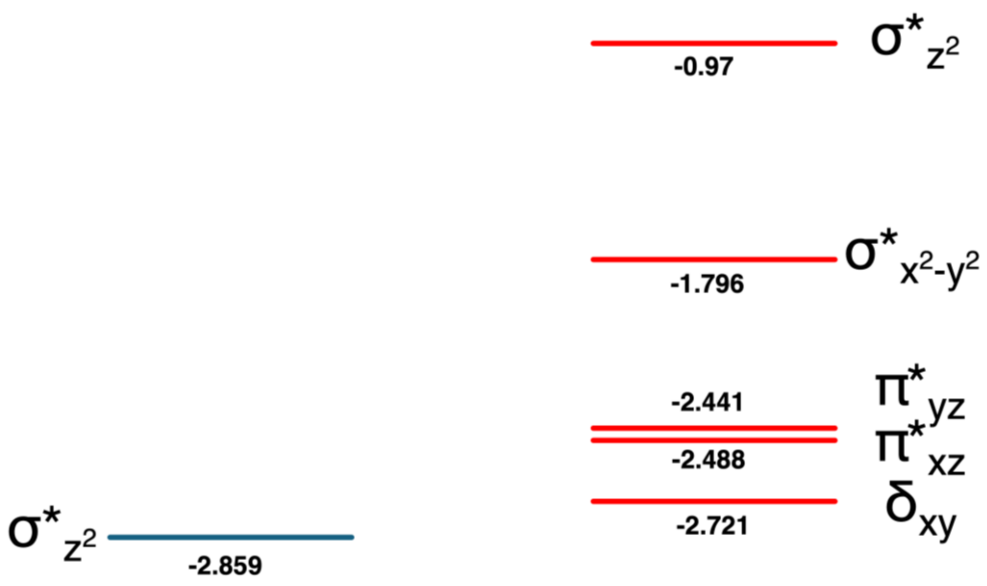


**Figure S65**. FMO for the HAT mechanism and their energies in the IL-AL-RC.

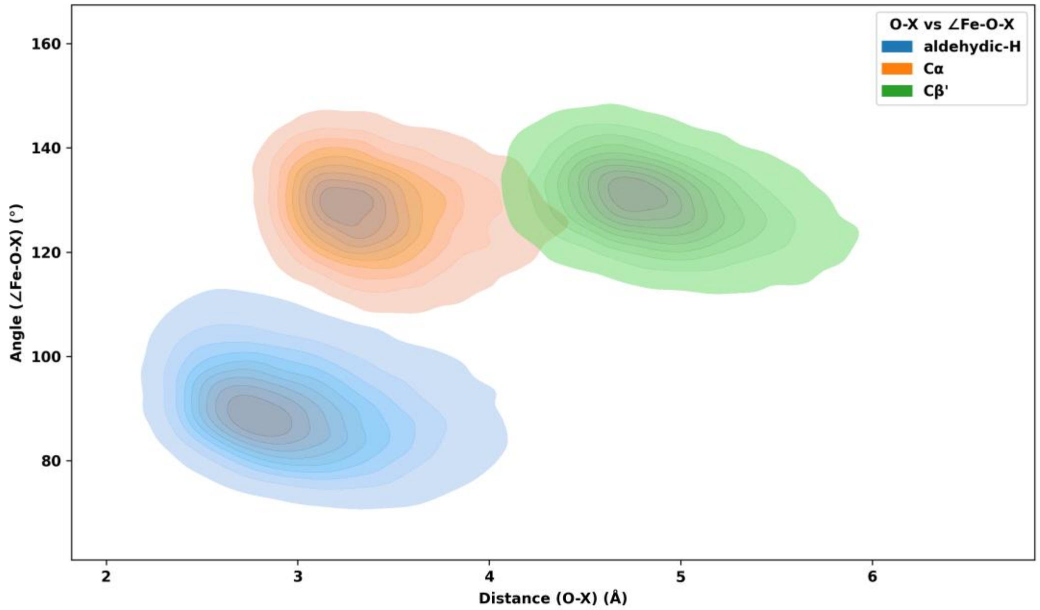


**Figure S66.** KDE plot of the O-X distance (in Å) versus the ∠Fe-O-X (in °) for the MD simulations of KDM6B-Fe(IV)=O•Lys(iPr-AL) system. X denotes carbons and hydrogen of the substrate.

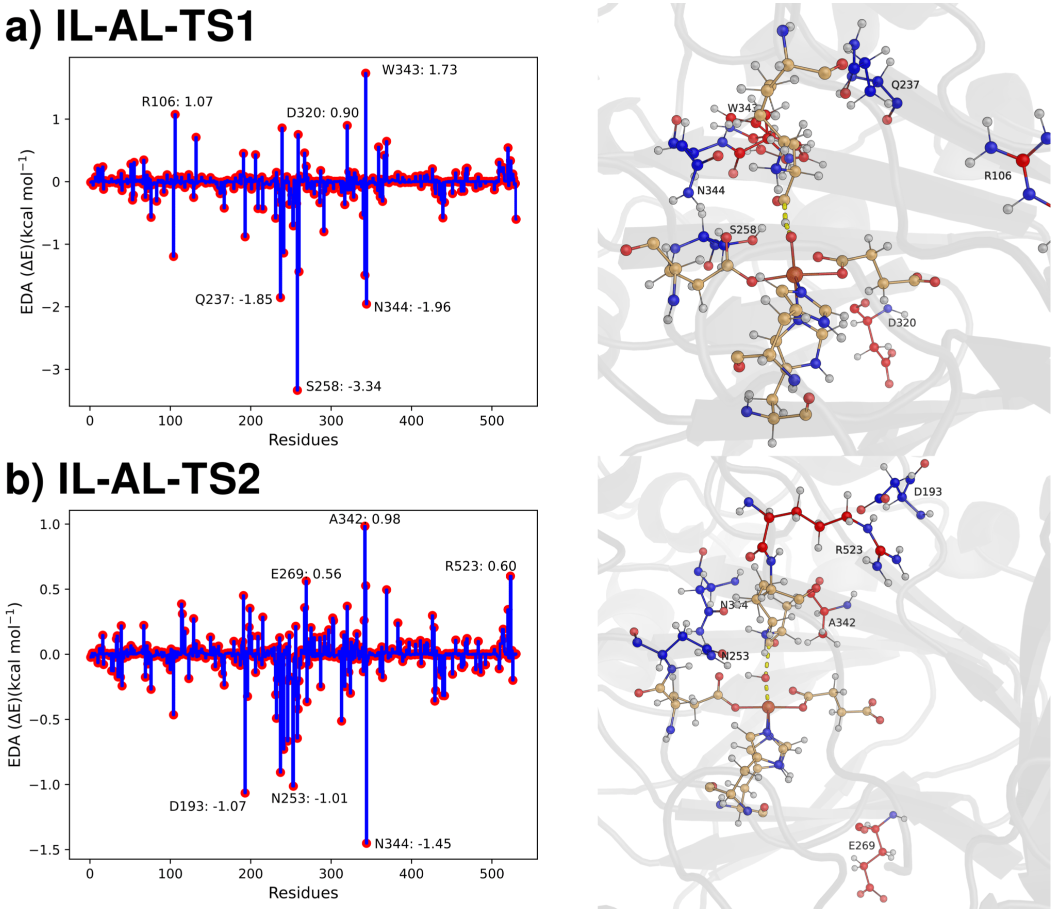


**Figure S67.** EDA analysis of the HAT and rebound reactions of iPr-AL group in the IL-AL-RC snapshot.

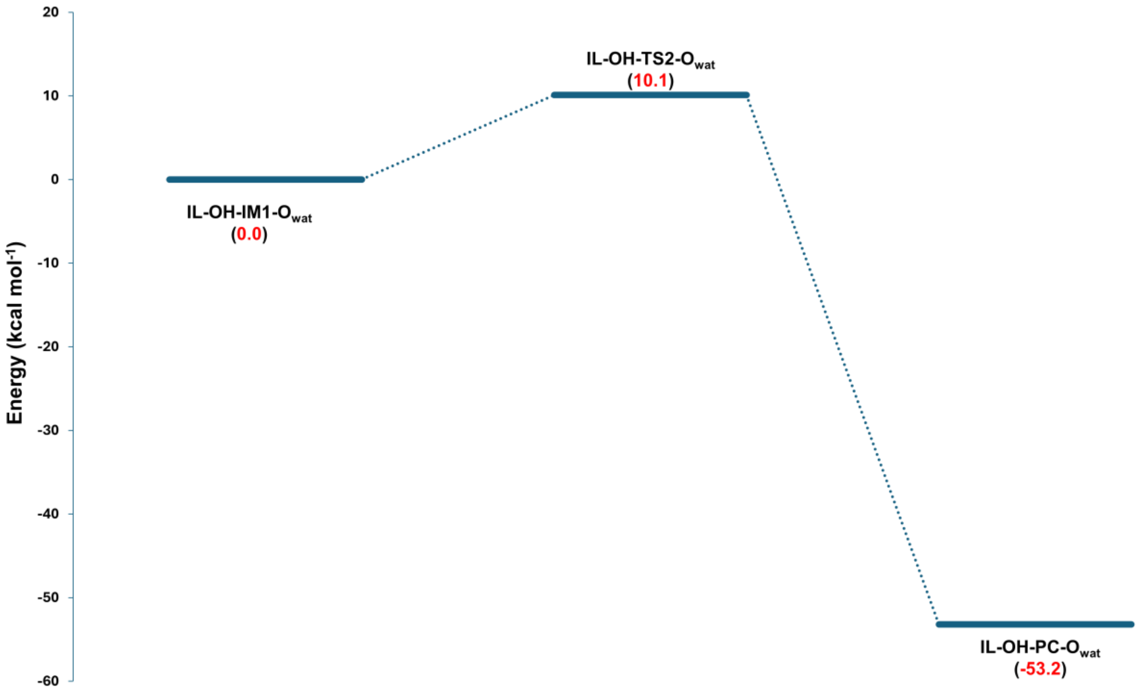


**Figure S68.** a) Reaction Profile of the conversion of IL-OH-IM1-O$_{wat}$ to Aldehyde product through water-mediated HAT. Relative energies are presented at the QM(B3)/MM level.

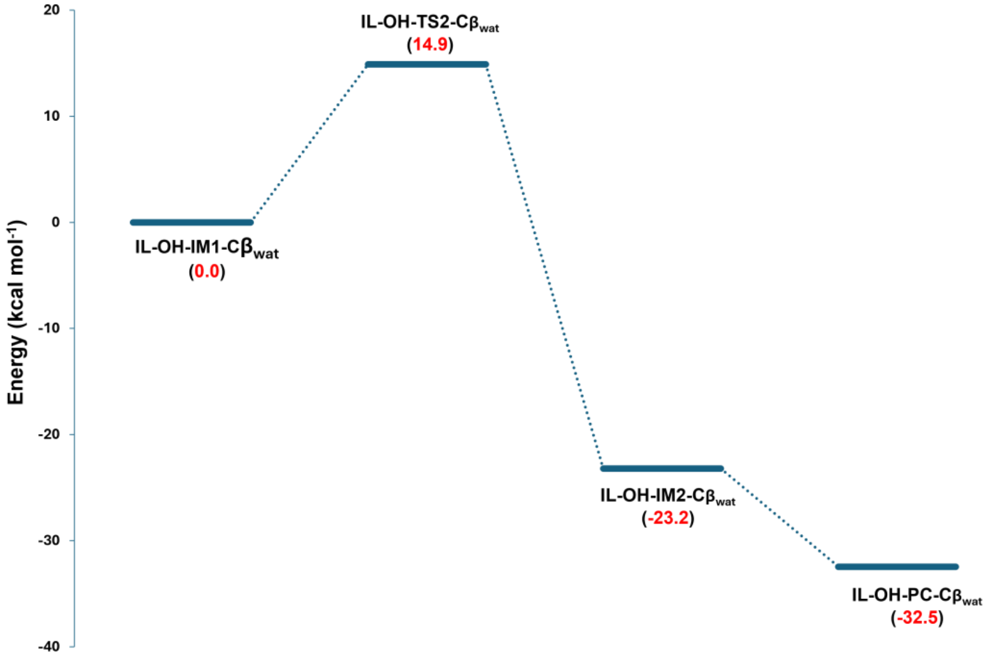


**Figure S69.** a) Reaction Profile of the conversion of IL-OH-IM1$_{wat}$ to Aldehyde product through water-mediated HAT. Relative energies are presented at the QM(B3)/MM level.

**Table S1**: Imaginary frequencies of transition states computed in this study.

| No. | TS | Imaginary frequency |
|---|---|---|
| 1 | MEL1-TS1-meth | -606.6324 |
| 2 | MEL1-TS2-meth | -149.5253 |
| 3 | MEL1-TS1-eth-Cbeta | -1458.7330 |
| 4 | MEL1-TS2-eth-Cbeta | -180.6653 |
| 5 | MEL2-TS1-eth-Cbeta | -114.1518 |
| 6 | MEL2-TS1-eth-Calpha | -908.6842 |
| 7 | MEL2-TS2-eth-Cbeta | -114.1518 |
| 8 | MEL2-TS2-eth-Calpha | -235.4463 |
| 9 | IL1-TS1-Calpha | -618.9546 |
| 10 | IL1-TS1-Cbeta | -1524.9032 |
| 11 | IL1-TS2-Calpha | -146.5801 |
| 12 | IL1-TS2-Cbeta | -205.6459 |
| 13 | MEL-OH-TS1-O | -759.4596 |
| 14 | MEL-OH-TS1-Cbeta | -266.9845 |
| 15 | MEL-OH-TS2-O | -903.6404 |
| 16 | MEL-OH-TS2-Cbeta | -1347.4830 |
| 17 | IL-OH-TS1-O | -143.2184 |

| No. | TS | Imaginary frequency |
|---|---|---|
| 18 | IL-OH-TS1-Cbeta | -1265.7219 |
| 19 | IL-OH-TS2-O | -158.8904 |
| 20 | IL-OH-TS2-Cbeta | -1387.2212 |
| 21 | MEL-AL-TS1 | -863.8298 |
| 22 | IL-AL-TS1 | -1570.2683 |
| 23 | IL-AL-TS2 | -110.7874 |
| 24 | MEL-OH-TS1-$O_{wat}$ | -123.2344 |
| 25 | MEL-OH-TS2-$O_{wat}$ | -488.534 |
| 26 | MEL-OH-TS2-$C\beta_{wat}$ | -306.1889 |
| 27 | MEL1-$TS1_{N344A}$-meth | -352.7859 |
| 28 | MEL1-$TS1_{N344A}$-eth | -1184.1177 |
| 29 | MEL2-$TS1_{N344A}$-meth | -708.3392 |
| 30 | MEL2-$TS1_{N344A}$-eth-Cα | -650.8593 |
| 31 | MEL2-$TS1_{N344A}$-eth-Cβ | -847.2622 |
| 32 | MEL1-$TS1_{Y239A}$-meth | -995.4136 |
| 33 | MEL1-$TS1_{Y239A}$-eth | -914.8043 |
| 34 | MEL2-$TS1_{Y239A}$-meth | -1301.8030 |
| 35 | MEL2-$TS1_{Y239A}$-eth-Cα | -1283.6437 |

**Table S2:** Calculated MM/GBSA values from the equilibrated regions from the MD simulations for the different substrates considered in the study.

| Substrate | Energy (kcal/mol) |
|---|---|
| WT | -98.2769 |
| Lys(Me/Eth) | -91.2039 |
| Lys(Me/Eth-OH) | -118.1133 |
| Lys(Me/Eth-AL) | -122.6597 |
| Lys(iPr) | -107.6939 |
| Lys(iPr-OH) | -110.9930 |
| Lys(iPr-AL) | -112.3404 |

## References


(1) Islam, Md. S.; Leissing, T. M.; Chowdhury, R.; Hopkinson, R. J.; Schofield, C. J. 2-Oxoglutarate-Dependent Oxygenases. *Annu. Rev. Biochem.* **2018**, *87* (1), 585–620. https://doi.org/10.1146/annurev-biochem-061516-044724.

(2) Hausinger, R. P. Fe(II)/α-Ketoglutarate-Dependent Hydroxylases and Related Enzymes. *Crit Rev Biochem Mol Biol* **2008**, *39* (1), 21–68. https://doi.org/10.1080/10409230490440541.

(3) Dinchuk, J. E.; Focht, R. J.; Kelley, J. A.; Henderson, N. L.; Zolotarjova, N. I.; Wynn, R.; Neff, N. T.; Link, J.; Huber, R. M.; Burn, T. C.; Rupar, M. J.; Cunningham, M. R.; Selling, B. H.; Ma, J.; Stern, A. A.; Hollis, G. F.; Stein, R. B.; Friedman, P. A. Absence of Post-Translational Aspartyl β-Hydroxylation of Epidermal Growth Factor Domains in Mice Leads to Developmental Defects and an Increased Incidence of Intestinal Neoplasia*. *J. Biol. Chem.* **2002**, *277* (15), 12970–12977. https://doi.org/10.1074/jbc.M110389200.

(4) Loenarz, C.; Schofield, C. J. Physiological and Biochemical Aspects of Hydroxylations and Demethylations Catalyzed by Human 2-Oxoglutarate Oxygenases. *Trends Biochem. Sci.* **2011**, *36* (1), 7–18. https://doi.org/10.1016/j.tibs.2010.07.002.

(5) Krishnan, A.; Waheed, S. O.; Varghese, A.; Cherilakkudy, F. H.; Schofield, C. J.; Karabencheva-Christova, T. G. Unusual Catalytic Strategy by Non-Heme Fe(II)/2-

Oxoglutarate-Dependent Aspartyl Hydroxylase AspH. *Chem. Sci.* **2024**, *15* (10), 3466–3484. https://doi.org/10.1039/D3SC05974J.

(6) Ikotun, A. M.; Ezugwu, A. E.; Abualigah, L.; Abuhaija, B.; Heming, J. K-Means Clustering Algorithms: A Comprehensive Review, Variants Analysis, and Advances in the Era of Big Data. *Inf. Sci.* **2023**, *622*, 178–210. https://doi.org/10.1016/j.ins.2022.11.139.

**Coordinates**

MEL1-RC

| | | | |
|---|---|---|---|
| 1 N | 56.6388394 | 48.2108091 | 44.3104712 |
| 2 C | 56.4147269 | 46.8480473 | 44.4001931 |
| 3 C | 55.5265163 | 46.0594226 | 43.4904287 |
| 4 N | 57.6427690 | 47.5524688 | 46.1368457 |
| 5 C | 57.0712756 | 46.4546326 | 45.5438954 |
| 6 C | 57.3724195 | 48.5989468 | 45.3694653 |
| 7 H | 55.6549814 | 46.3451376 | 42.4361628 |
| 8 H | 55.8093881 | 44.9963702 | 43.5508368 |
| 9 H | 57.1670278 | 45.4613301 | 45.9713556 |
| 10 H | 57.6966385 | 49.6179847 | 45.5595340 |
| 11 H | 56.2922072 | 48.8733751 | 43.6043216 |
| 12 C | 58.1782048 | 42.5489529 | 49.6489909 |
| 13 C | 58.2161745 | 43.9693377 | 50.2404142 |
| 14 C | 59.1100318 | 45.0084459 | 49.5372990 |
| 15 O | 60.3399359 | 44.9315151 | 49.6440324 |
| 16 O | 58.4383443 | 45.9205390 | 48.9195928 |
| 17 H | 59.1923912 | 42.1145797 | 49.6628837 |
| 18 H | 57.5530514 | 41.9257223 | 50.3092372 |
| 19 H | 57.2021015 | 44.3811878 | 50.3342753 |
| 20 H | 58.6057235 | 43.8687809 | 51.2675264 |
| 21 N | 54.7679811 | 47.6870099 | 49.6084257 |
| 22 C | 54.8059905 | 49.0427706 | 49.3215630 |
| 23 C | 53.6349292 | 49.9558134 | 49.5217563 |
| 24 N | 56.7805864 | 48.0736823 | 48.8184109 |
| 25 C | 56.0745939 | 49.2615288 | 48.8297129 |
| 26 C | 55.9600962 | 47.1432899 | 49.2901246 |
| 27 H | 53.0589820 | 49.6708477 | 50.4171418 |
| 28 H | 53.9790319 | 50.9878273 | 49.6882939 |
| 29 H | 56.5114880 | 50.1891536 | 48.4692093 |
| 30 H | 56.2009780 | 46.0915007 | 49.4001095 |
| 31 H | 53.9724350 | 47.1656468 | 49.9853218 |
| 32 Fe | 58.6790084 | 47.6286009 | 47.9547923 |
| 33 O | 60.0395110 | 47.1578827 | 47.1947827 |
| 34 C | 59.5310614 | 49.9438784 | 48.5120004 |
| 35 O | 58.8433343 | 49.6867455 | 47.4769216 |
| 36 C | 60.1389364 | 51.3098143 | 48.6848752 |
| 37 C | 61.5578996 | 51.3857600 | 48.0772102 |
| 38 C | 62.0953462 | 52.8402347 | 48.0867002 |
| 39 O | 62.4876508 | 53.3030957 | 46.9992525 |
| 40 O | 62.0746861 | 53.4402176 | 49.1975247 |
| 41 H | 60.1806461 | 51.5824106 | 49.7463501 |
| 42 H | 59.4837229 | 52.0301656 | 48.1723545 |
| 43 H | 62.2465456 | 50.7579306 | 48.6695367 |
| 44 H | 61.5573914 | 51.0178812 | 47.0407830 |
| 45 O | 59.7072079 | 48.9828852 | 49.3318505 |
| 46 C | 63.0794096 | 45.6657093 | 47.6339638 |
| 47 N | 62.9239558 | 46.1670372 | 49.0417843 |
| 48 H | 63.8333436 | 46.2890407 | 47.1353867 |
| 49 H | 62.1159972 | 45.8377505 | 47.1377851 |
| 50 H | 62.0813684 | 45.6786165 | 49.4114676 |
| 51 C | 64.0927764 | 45.8852883 | 49.9665471 |
| 52 H | 64.1765419 | 44.7869383 | 50.0373526 |

| | | | |
|---|---|---|---|
| 53 C | 65.3945509 | 46.5474835 | 49.5319939 |
| 54 H | 63.7701773 | 46.2540064 | 50.9527898 |
| 55 C | 62.5811654 | 47.6259031 | 49.0344649 |
| 56 H | 62.3846462 | 47.9492327 | 50.0648487 |
| 57 H | 63.4155129 | 48.1991298 | 48.6089791 |
| 58 H | 61.6782218 | 47.7627152 | 48.4338241 |
| 59 H | 65.3557930 | 47.6448909 | 49.5673619 |
| 60 H | 65.6980315 | 46.2405756 | 48.5203642 |
| 61 H | 66.2038725 | 46.2263696 | 50.2021454 |
| 62 H | 54.4713442 | 46.1891926 | 43.7309255 |
| 63 H | 57.7758480 | 42.4424497 | 48.6416015 |
| 64 H | 52.9162422 | 49.9523069 | 48.7022785 |
| 65 H | 63.3537164 | 44.6157453 | 47.5319903 |

MEL1-TS1-meth

| | | | |
|---|---|---|---|
| 1 N | 56.6915362 | 48.2197180 | 44.3642099 |
| 2 C | 56.4512052 | 46.8608447 | 44.4665869 |
| 3 C | 55.5753676 | 46.0723291 | 43.5437312 |
| 4 N | 57.6697318 | 47.5707853 | 46.2109413 |
| 5 C | 57.0893622 | 46.4731884 | 45.6233388 |
| 6 C | 57.4176860 | 48.6114237 | 45.4274666 |
| 7 H | 55.7185249 | 46.3618272 | 42.4920916 |
| 8 H | 55.8581890 | 45.0093188 | 43.6049802 |
| 9 H | 57.1663296 | 45.4836690 | 46.0639140 |
| 10 H | 57.7438965 | 49.6307941 | 45.6132066 |
| 11 H | 56.3534747 | 48.8777128 | 43.6493074 |
| 12 C | 58.0874348 | 42.5270756 | 49.7066041 |
| 13 C | 58.1035010 | 43.9534745 | 50.2846230 |
| 14 C | 59.0245865 | 44.9720113 | 49.5929684 |
| 15 O | 60.2563678 | 44.8049720 | 49.6793732 |
| 16 O | 58.4213378 | 45.9381925 | 49.0122253 |
| 17 H | 59.1026778 | 42.0981064 | 49.7511748 |
| 18 H | 57.4439640 | 41.9044154 | 50.3492322 |
| 19 H | 57.0883410 | 44.3688020 | 50.3429579 |
| 20 H | 58.4622666 | 43.8663312 | 51.3244198 |
| 21 N | 54.7155297 | 47.7014396 | 49.6384443 |
| 22 C | 54.7503505 | 49.0580030 | 49.3582567 |
| 23 C | 53.5742674 | 49.9672621 | 49.5420319 |
| 24 N | 56.7295962 | 48.0997848 | 48.8657650 |
| 25 C | 56.0211487 | 49.2861563 | 48.8775350 |
| 26 C | 55.9109735 | 47.1619815 | 49.3258621 |
| 27 H | 52.9856995 | 49.6816925 | 50.4287341 |
| 28 H | 53.9125820 | 51.0004410 | 49.7123161 |
| 29 H | 56.4598847 | 50.2152422 | 48.5239919 |
| 30 H | 56.1574318 | 46.1112095 | 49.4326894 |
| 31 H | 53.9202871 | 47.1737611 | 50.0074994 |
| 32 Fe | 58.5950367 | 47.7119361 | 48.0555651 |
| 33 O | 60.0894360 | 47.2464381 | 47.2366146 |
| 34 C | 59.4774345 | 50.0156064 | 48.5874400 |
| 35 O | 58.7352857 | 49.8080455 | 47.5852395 |
| 36 C | 60.1363231 | 51.3560098 | 48.7681608 |
| 37 C | 61.5426899 | 51.3968523 | 48.1291707 |
| 38 C | 62.1106617 | 52.8396525 | 48.1287199 |
| 39 O | 62.5022770 | 53.2921787 | 47.0366043 |

| | | | |
|---|---|---|---|
| 40 O | 62.1080336 | 53.4426623 | 49.2381349 |
| 41 H | 60.2116036 | 51.6139043 | 49.8308987 |
| 42 H | 59.4920961 | 52.1030788 | 48.2815647 |
| 43 H | 62.2293872 | 50.7527781 | 48.7068130 |
| 44 H | 61.5110902 | 51.0307189 | 47.0923055 |
| 45 O | 59.6703153 | 49.0238331 | 49.3769993 |
| 46 C | 62.8454886 | 45.4471475 | 47.4666175 |
| 47 N | 62.5608655 | 46.0204711 | 48.8205813 |
| 48 H | 63.5425998 | 46.1234455 | 46.9525262 |
| 49 H | 61.8947671 | 45.4590382 | 46.9182803 |
| 50 H | 61.7092909 | 45.5120080 | 49.1914646 |
| 51 C | 63.6715005 | 45.8683642 | 49.8552562 |
| 52 H | 63.7919616 | 44.7801211 | 50.0090606 |
| 53 C | 64.9701150 | 46.5615170 | 49.4705100 |
| 54 H | 63.2519408 | 46.2881029 | 50.7822729 |
| 55 C | 62.1402834 | 47.4232164 | 48.6794171 |
| 56 H | 61.7758391 | 47.8564466 | 49.6149562 |
| 57 H | 62.8465193 | 48.0368342 | 48.1074734 |
| 58 H | 61.0114742 | 47.4083250 | 47.8903919 |
| 59 H | 64.8796424 | 47.6563579 | 49.4323756 |
| 60 H | 65.3604778 | 46.2092784 | 48.5043468 |
| 61 H | 65.7424862 | 46.3170989 | 50.2119991 |
| 62 H | 54.5156114 | 46.1989500 | 43.7649804 |
| 63 H | 57.7143465 | 42.4222470 | 48.6878386 |
| 64 H | 52.8703092 | 49.9587112 | 48.7099040 |
| 65 H | 63.2491142 | 44.4354025 | 47.4275599 |

MEL1-IM1-meth

| | | | |
|---|---|---|---|
| 1 N | 56.6028901 | 48.2504084 | 44.4080527 |
| 2 C | 56.3832165 | 46.8840349 | 44.4635076 |
| 3 C | 55.5299137 | 46.1022969 | 43.5147378 |
| 4 N | 57.5349788 | 47.5586066 | 46.2677510 |
| 5 C | 56.9927087 | 46.4751528 | 45.6273288 |
| 6 C | 57.2933939 | 48.6150025 | 45.5078273 |
| 7 H | 55.6725236 | 46.4136648 | 42.4695196 |
| 8 H | 55.8306922 | 45.0430288 | 43.5563947 |
| 9 H | 57.0758366 | 45.4733366 | 46.0376818 |
| 10 H | 57.6199635 | 49.6280638 | 45.7252742 |
| 11 H | 56.2837195 | 48.9195861 | 43.6962629 |
| 12 C | 58.0905417 | 42.4594554 | 49.7082533 |
| 13 C | 58.1178820 | 43.8662056 | 50.3289086 |
| 14 C | 59.0025040 | 44.9222208 | 49.6411610 |
| 15 O | 60.2386723 | 44.8176256 | 49.7338515 |
| 16 O | 58.3351709 | 45.8512215 | 49.0663065 |
| 17 H | 59.1036665 | 42.0227909 | 49.7361848 |
| 18 H | 57.4479149 | 41.8220498 | 50.3376872 |
| 19 H | 57.1004787 | 44.2691861 | 50.4264606 |
| 20 H | 58.5065907 | 43.7488698 | 51.3546071 |
| 21 N | 54.6786564 | 47.6978916 | 49.7234523 |
| 22 C | 54.7348431 | 49.0495808 | 49.4159481 |
| 23 C | 53.5657940 | 49.9738761 | 49.5694378 |
| 24 N | 56.7118794 | 48.0571059 | 48.9834319 |
| 25 C | 56.0179344 | 49.2510488 | 48.9572862 |
| 26 C | 55.8734637 | 47.1394265 | 49.4475719 |

| | | | |
|---|---|---|---|
| 27 H | 52.9668747 | 49.7089095 | 50.4558273 |
| 28 H | 53.9127875 | 51.0062187 | 49.7275841 |
| 29 H | 56.4753379 | 50.1683904 | 48.5954498 |
| 30 H | 56.1072191 | 46.0874359 | 49.5746387 |
| 31 H | 53.8698124 | 47.1834521 | 50.0816585 |
| 32 Fe | 58.5348692 | 47.5929442 | 48.1609113 |
| 33 O | 60.0211859 | 46.9996245 | 47.3217952 |
| 34 C | 59.5180383 | 49.8771802 | 48.7427648 |
| 35 O | 58.8855979 | 49.5519128 | 47.6856886 |
| 36 C | 60.1236370 | 51.2582068 | 48.8350091 |
| 37 C | 61.5640207 | 51.3031773 | 48.2793923 |
| 38 C | 62.1009841 | 52.7550697 | 48.2198577 |
| 39 O | 62.4515039 | 53.1896797 | 47.1021318 |
| 40 O | 62.1243290 | 53.3928586 | 49.3072240 |
| 41 H | 60.1250772 | 51.6035731 | 49.8767091 |
| 42 H | 59.4890694 | 51.9404508 | 48.2489981 |
| 43 H | 62.2285032 | 50.7141454 | 48.9354942 |
| 44 H | 61.5927468 | 50.8677924 | 47.2696112 |
| 45 O | 59.6327386 | 48.9986691 | 49.6486949 |
| 46 C | 62.8421456 | 45.5467030 | 47.5267468 |
| 47 N | 62.5897456 | 46.0783116 | 48.9110626 |
| 48 H | 63.5893732 | 46.1978766 | 47.0516522 |
| 49 H | 61.8919186 | 45.6565605 | 46.9909457 |
| 50 H | 61.7072240 | 45.6032931 | 49.2492678 |
| 51 C | 63.7017039 | 45.8039235 | 49.9232494 |
| 52 H | 63.7769713 | 44.7052626 | 50.0203703 |
| 53 C | 65.0196228 | 46.4640159 | 49.5413661 |
| 54 H | 63.3179712 | 46.1983874 | 50.8760606 |
| 55 C | 62.2931293 | 47.5013488 | 48.8785957 |
| 56 H | 61.5125625 | 47.8804101 | 49.5350294 |
| 57 H | 62.8472161 | 48.1224598 | 48.1764183 |
| 58 H | 60.2956460 | 47.6767429 | 46.6654749 |
| 59 H | 64.9558487 | 47.5620091 | 49.5404341 |
| 60 H | 65.3793607 | 46.1332546 | 48.5558853 |
| 61 H | 65.8007153 | 46.1710930 | 50.2545025 |
| 62 H | 54.4681541 | 46.2057372 | 43.7384201 |
| 63 H | 57.7138990 | 42.3792667 | 48.6885587 |
| 64 H | 52.8661368 | 49.9613159 | 48.7337410 |
| 65 H | 63.1929665 | 44.5176111 | 47.4495354 |

MEL1-TS2-meth

| | | | |
|---|---|---|---|
| 1 N | 56.6637511 | 48.2357010 | 44.4238181 |
| 2 C | 56.4185168 | 46.8741992 | 44.4996522 |
| 3 C | 55.5599579 | 46.0955881 | 43.5526331 |
| 4 N | 57.5991949 | 47.5520127 | 46.2863614 |
| 5 C | 57.0272769 | 46.4699839 | 45.6655166 |
| 6 C | 57.3692820 | 48.6032537 | 45.5121828 |
| 7 H | 55.7082825 | 46.4049107 | 42.5074279 |
| 8 H | 55.8534678 | 45.0343928 | 43.5967299 |
| 9 H | 57.0896190 | 45.4740422 | 46.0934925 |
| 10 H | 57.7092582 | 49.6141004 | 45.7198048 |
| 11 H | 56.3433021 | 48.9013486 | 43.7090906 |
| 12 C | 58.0475531 | 42.4448468 | 49.7313863 |
| 13 C | 58.0673500 | 43.8588359 | 50.3355550 |

| | | | | | | | |
|---|---|---|---|---|---|---|---|
| 14 C | 58.9561726 | 44.8999911 | 49.6325544 | 41 H | 60.1338338 | 51.6498580 | 49.9200777 |
| 15 O | 60.1947975 | 44.7547033 | 49.7032013 | 42 H | 59.4669810 | 52.0535656 | 48.3210489 |
| 16 O | 58.3158687 | 45.8459723 | 49.0666324 | 43 H | 62.1844261 | 50.7270906 | 48.9036024 |
| 17 H | 59.0608399 | 42.0100502 | 49.7729554 | 44 H | 61.5084153 | 50.9263233 | 47.2593465 |
| 18 H | 57.3974359 | 41.8107842 | 50.3563436 | 45 O | 59.5557172 | 49.0724866 | 49.6355909 |
| 19 H | 57.0492441 | 44.2627803 | 50.4221515 | 46 C | 62.7701027 | 45.4232312 | 47.4532369 |
| 20 H | 58.4525845 | 43.7569924 | 51.3644046 | 47 N | 62.4642883 | 45.9988999 | 48.8006328 |
| 21 N | 54.6179784 | 47.7280284 | 49.7694517 | 48 H | 63.4903717 | 46.0920366 | 46.9601084 |
| 22 C | 54.6611906 | 49.0771169 | 49.4496397 | 49 H | 61.8303462 | 45.4560279 | 46.8890500 |
| 23 C | 53.4847466 | 49.9937998 | 49.5863485 | 50 H | 61.5873205 | 45.5091686 | 49.1543290 |
| 24 N | 56.6464492 | 48.1002487 | 49.0225775 | 51 C | 63.5560797 | 45.8183188 | 49.8543546 |
| 25 C | 55.9409230 | 49.2862853 | 48.9859426 | 52 H | 63.6663975 | 44.7287252 | 50.0069465 |
| 26 C | 55.8192390 | 47.1790749 | 49.4970054 | 53 C | 64.8636826 | 46.5042553 | 49.4838838 |
| 27 H | 52.8724405 | 49.7263033 | 50.4625154 | 54 H | 63.1292463 | 46.2432096 | 50.7753057 |
| 28 H | 53.8217199 | 51.0287813 | 49.7488541 | 55 C | 62.0963930 | 47.3971688 | 48.6721217 |
| 29 H | 56.3898335 | 50.2043205 | 48.6147626 | 56 H | 61.5329521 | 47.8378360 | 49.4914978 |
| 30 H | 56.0646221 | 46.1302086 | 49.6323166 | 57 H | 62.6749289 | 48.0065672 | 47.9793015 |
| 31 H | 53.8131904 | 47.2060851 | 50.1253618 | 58 H | 60.3532591 | 47.7814983 | 46.7574719 |
| 32 Fe | 58.4974799 | 47.6664472 | 48.2084017 | 59 H | 64.7606436 | 47.5967813 | 49.4051280 |
| 33 O | 60.1249319 | 47.1599048 | 47.4849299 | 60 H | 65.2822094 | 46.1226976 | 48.5407665 |
| 34 C | 59.4487871 | 49.9762215 | 48.7466275 | 61 H | 65.6189194 | 46.2929611 | 50.2517780 |
| 35 O | 58.8076600 | 49.6914747 | 47.6894306 | 62 H | 54.4972088 | 46.2053628 | 43.7684592 |
| 36 C | 60.0949497 | 51.3357134 | 48.8702203 | 63 H | 57.6821976 | 42.3628378 | 48.7077374 |
| 37 C | 61.5199008 | 51.3468586 | 48.2757588 | 64 H | 52.8017809 | 49.9733446 | 48.7371096 |
| 38 C | 62.0988324 | 52.7810905 | 48.2194509 | 65 H | 63.1754120 | 44.4123726 | 47.4090064 |
| 39 O | 62.4663785 | 53.2051037 | 47.1029015 | | | | |
| 40 O | 62.1360020 | 53.4188364 | 49.3064831 | | | | |

MEL1-PC-meth

| | | | | | | | |
|---|---|---|---|---|---|---|---|
| 1 N | 56.7304159 | 48.2756062 | 44.5136102 | 28 H | 53.6186909 | 51.0639122 | 49.7908451 |
| 2 C | 56.4756259 | 46.9175342 | 44.5928556 | 29 H | 56.2328742 | 50.2428899 | 48.8265172 |
| 3 C | 55.6181553 | 46.1445551 | 43.6390506 | 30 H | 55.8488372 | 46.1563264 | 49.7594548 |
| 4 N | 57.7439866 | 47.5743916 | 46.3419605 | 31 H | 53.5895127 | 47.2246112 | 50.1700738 |
| 5 C | 57.1260412 | 46.5048008 | 45.7355336 | 32 Fe | 58.4357347 | 47.8097508 | 48.4157423 |
| 6 C | 57.4832171 | 48.6279811 | 45.5788721 | 33 O | 60.5523538 | 47.2274221 | 47.7672881 |
| 7 H | 55.7816852 | 46.4599239 | 42.5972795 | 34 C | 59.4859133 | 50.1092424 | 48.8391763 |
| 8 H | 55.9105845 | 45.0830221 | 43.6808425 | 35 O | 58.7806542 | 49.8238181 | 47.8224898 |
| 9 H | 57.1619928 | 45.5054565 | 46.1639553 | 36 C | 60.1515655 | 51.4601744 | 48.9097168 |
| 10 H | 57.8142867 | 49.6417729 | 45.7920605 | 37 C | 61.5881471 | 51.4107820 | 48.3490018 |
| 11 H | 56.3856707 | 48.9443447 | 43.8128424 | 38 C | 62.1772661 | 52.8336888 | 48.2435127 |
| 12 C | 57.8790866 | 42.4264711 | 49.8068738 | 39 O | 62.5255585 | 53.2323453 | 47.1117596 |
| 13 C | 57.8939908 | 43.8601013 | 50.3657041 | 40 O | 62.2245057 | 53.4977914 | 49.3146085 |
| 14 C | 58.8709805 | 44.8256902 | 49.6836620 | 41 H | 60.1802327 | 51.8274697 | 49.9428623 |
| 15 O | 60.0914147 | 44.5214105 | 49.7260367 | 42 H | 59.5509602 | 52.1627873 | 48.3133883 |
| 16 O | 58.3774021 | 45.8552285 | 49.1404753 | 43 H | 62.2247146 | 50.8096161 | 49.0222816 |
| 17 H | 58.8811575 | 41.9789389 | 49.9143212 | 44 H | 61.5930310 | 50.9454043 | 47.3513396 |
| 18 H | 57.1811813 | 41.8190346 | 50.4054910 | 45 O | 59.6566107 | 49.2157291 | 49.7284020 |
| 19 H | 56.8898402 | 44.3052900 | 50.3639758 | 46 C | 62.5885426 | 45.3016287 | 47.4294194 |
| 20 H | 58.2046456 | 43.7883458 | 51.4222515 | 47 N | 62.2062430 | 45.8297789 | 48.7708308 |
| 21 N | 54.4044809 | 47.7558623 | 49.8566233 | 48 H | 63.2284977 | 46.0458093 | 46.9328891 |
| 22 C | 54.4644313 | 49.1072886 | 49.5586425 | 49 H | 61.6641179 | 45.2158151 | 46.8436165 |
| 23 C | 53.2863667 | 50.0276079 | 49.6291018 | 50 H | 61.3771052 | 45.2473789 | 49.1355608 |
| 24 N | 56.4682370 | 48.1327009 | 49.2065161 | 51 C | 63.2805764 | 45.8028937 | 49.8327892 |
| 25 C | 55.7665011 | 49.3187593 | 49.1620258 | 52 H | 63.4804507 | 44.7352999 | 50.0369676 |
| 26 C | 55.6197885 | 47.2102801 | 49.6250746 | 53 C | 64.5515529 | 46.5657766 | 49.4752249 |
| 27 H | 52.6322990 | 49.7781489 | 50.4798786 | 54 H | 62.8059509 | 46.2230245 | 50.7330568 |

| | | | |
|---|---|---|---|
| 55 C | 61.6247699 | 47.2198026 | 48.6496266 |
| 56 H | 61.2641878 | 47.5125037 | 49.6437471 |
| 57 H | 62.4232533 | 47.9036049 | 48.3275926 |
| 58 H | 60.8514362 | 47.4916328 | 46.8719609 |
| 59 H | 64.3941732 | 47.6468891 | 49.3425077 |
| 60 H | 65.0388131 | 46.1708703 | 48.5716271 |
| 61 H | 65.2799216 | 46.4434985 | 50.2877983 |
| 62 H | 54.5494315 | 46.2470603 | 43.8271709 |
| 63 H | 57.5670045 | 42.3360209 | 48.7664456 |
| 64 H | 52.6476936 | 49.9913439 | 48.7465777 |
| 65 H | 63.0915277 | 44.3354781 | 47.3890951 |

MEL1-RC

| | | | |
|---|---|---|---|
| 1 N | 56.6388394 | 48.2108091 | 44.3104712 |
| 2 C | 56.4147269 | 46.8480473 | 44.4001931 |
| 3 C | 55.5265163 | 46.0594226 | 43.4904287 |
| 4 N | 57.6427690 | 47.5524688 | 46.1368457 |
| 5 C | 57.0712756 | 46.4546326 | 45.5438954 |
| 6 C | 57.3724195 | 48.5989468 | 45.3694653 |
| 7 H | 55.6549814 | 46.3451376 | 42.4361628 |
| 8 H | 55.8093881 | 44.9963702 | 43.5508368 |
| 9 H | 57.1670278 | 45.4613301 | 45.9713556 |
| 10 H | 57.6966385 | 49.6179847 | 45.5595340 |
| 11 H | 56.2922072 | 48.8733751 | 43.6043216 |
| 12 C | 58.1782048 | 42.5489529 | 49.6489909 |
| 13 C | 58.2161745 | 43.9693377 | 50.2404142 |
| 14 C | 59.1100318 | 45.0084459 | 49.5372990 |
| 15 O | 60.3399359 | 44.9315151 | 49.6440324 |
| 16 O | 58.4383443 | 45.9205390 | 48.9195928 |
| 17 H | 59.1923912 | 42.1145797 | 49.6628837 |
| 18 H | 57.5530514 | 41.9257223 | 50.3092372 |
| 19 H | 57.2021015 | 44.3811878 | 50.3342753 |
| 20 H | 58.6057235 | 43.8687809 | 51.2675264 |
| 21 N | 54.7679811 | 47.6870099 | 49.6084257 |
| 22 C | 54.8059905 | 49.0427706 | 49.3215630 |
| 23 C | 53.6349292 | 49.9558134 | 49.5217563 |
| 24 N | 56.7805864 | 48.0736823 | 48.8184109 |
| 25 C | 56.0745939 | 49.2615288 | 48.8297129 |
| 26 C | 55.9600962 | 47.1432899 | 49.2901246 |
| 27 H | 53.0589820 | 49.6708477 | 50.4171418 |
| 28 H | 53.9790319 | 50.9878273 | 49.6882939 |
| 29 H | 56.5114880 | 50.1891536 | 48.4692093 |
| 30 H | 56.2009780 | 46.0915007 | 49.4001095 |
| 31 H | 53.9724350 | 47.1656468 | 49.9853218 |
| 32 Fe | 58.6790084 | 47.6286009 | 47.9547923 |
| 33 O | 60.0395110 | 47.1578827 | 47.1947827 |
| 34 C | 59.5310614 | 49.9438784 | 48.5120004 |
| 35 O | 58.8433343 | 49.6867455 | 47.4769216 |
| 36 C | 60.1389364 | 51.3098143 | 48.6848752 |
| 37 C | 61.5578996 | 51.3857600 | 48.0772102 |
| 38 C | 62.0953462 | 52.8402347 | 48.0867002 |
| 39 O | 62.4876508 | 53.3030957 | 46.9992525 |
| 40 O | 62.0746861 | 53.4402176 | 49.1975247 |
| 41 H | 60.1806461 | 51.5824106 | 49.7463501 |

42 H 59.4837229 52.0301656 48.1723545

43 H 62.2465456 50.7579306 48.6695367

44 H 61.5573914 51.0178812 47.0407830

45 O 59.7072079 48.9828852 49.3318505

46 C 63.0794096 45.6657093 47.6339638

47 N 62.9239558 46.1670372 49.0417843

48 H 63.8333436 46.2890407 47.1353867

49 H 62.1159972 45.8377505 47.1377851

50 H 62.0813684 45.6786165 49.4114676

51 C 64.0927764 45.8852883 49.9665471

52 H 64.1765419 44.7869383 50.0373526

53 C 65.3945509 46.5474835 49.5319939

54 H 63.7701773 46.2540064 50.9527898

55 C 62.5811654 47.6259031 49.0344649

56 H 62.3846462 47.9492327 50.0648487

57 H 63.4155129 48.1991298 48.6089791

58 H 61.6782218 47.7627152 48.4338241

59 H 65.3557930 47.6448909 49.5673619

60 H 65.6980315 46.2405756 48.5203642

61 H 66.2038725 46.2263696 50.2021454

62 H 54.4713442 46.1891926 43.7309255

63 H 57.7758480 42.4424497 48.6416015

64 H 52.9162422 49.9523069 48.7022785

65 H 63.3537164 44.6157453 47.5319903

MEL1-TS1-eth

1 N 56.7112118 48.1952714 44.4392218

2 C 56.4641552 46.8323582 44.4979654

3 C 55.5663959 46.0790865 43.5671098

4 N 57.8014263 47.4542257 46.1931658

5 C 57.1651578 46.3907211 45.5967913

6 C 57.5076176 48.5298973 45.4714352

7 H 55.7137123 46.3866979 42.5207710

8 H 55.8340614 45.0111604 43.6086512

9 H 57.2472641 45.3844763 45.9971874

10 H 57.8624137 49.5355553 45.6783325

11 H 56.3466100 48.8796133 43.7632816

12 C 57.8444970 42.4366775 49.7585100

13 C 57.8385751 43.8744064 50.3087774

14 C 58.8478138 44.8338271 49.6598953

15 O 60.0434229 44.6971553 49.9167019

16 O 58.3390090 45.7447367 48.8950898

17 H 58.8502806 41.9990707 49.8757322

18 H 57.1519653 41.8259688 50.3604994

19 H 56.8302674 44.3090320 50.2775660

20 H 58.1216573 43.8097846 51.3721043

21 N 54.7474268 47.6824628 49.6262834

22 C 54.8165896 49.0402056 49.3589477

23 C 53.6504929 49.9638870 49.5390120

24 N 56.7982168 48.0449731 48.9084233

25 C 56.1077861 49.2418576 48.9158903

26 C 55.9442303 47.1279359 49.3379125

27 H 53.0692290 49.6943366 50.4362315

28 H 54.0009702 50.9954269 49.6952869

| | | | |
|---|---|---|---|
| 29 H | 56.5696342 | 50.1710302 | 48.5893285 |
| 30 H | 56.1612886 | 46.0697972 | 49.4306561 |
| 31 H | 53.9356538 | 47.1675222 | 49.9749477 |
| 32 Fe | 58.8437860 | 47.4878849 | 48.0613094 |
| 33 O | 60.3808851 | 46.9225828 | 47.3165988 |
| 34 C | 59.6768154 | 49.8040710 | 48.6335410 |
| 35 O | 59.0522109 | 49.5426014 | 47.5597169 |
| 36 C | 60.2379508 | 51.1833656 | 48.8433059 |
| 37 C | 61.6305867 | 51.3374882 | 48.1960727 |
| 38 C | 62.1091943 | 52.8096758 | 48.2183823 |
| 39 O | 62.4707680 | 53.3009005 | 47.1301878 |
| 40 O | 62.0821760 | 53.3989343 | 49.3324390 |
| 41 H | 60.2978984 | 51.4223410 | 49.9108166 |
| 42 H | 59.5399198 | 51.8937507 | 48.3738089 |
| 43 H | 62.3628530 | 50.7205957 | 48.7460668 |
| 44 H | 61.6053730 | 50.9930573 | 47.1526826 |
| 45 O | 59.8192731 | 48.8441362 | 49.4631656 |
| 46 C | 62.5225595 | 44.2027098 | 48.2481528 |
| 47 N | 63.3770445 | 45.3315663 | 48.7808690 |
| 48 H | 61.7605234 | 44.6064572 | 47.5766411 |
| 49 H | 62.0121935 | 43.7844931 | 49.1207049 |
| 50 H | 64.2090718 | 44.8833390 | 49.1887596 |
| 51 C | 63.8637415 | 46.3176088 | 47.7356350 |
| 52 H | 64.7081227 | 45.8414434 | 47.2075966 |
| 53 C | 62.8143059 | 46.7489818 | 46.7411328 |
| 54 H | 64.2791172 | 47.1758932 | 48.2801225 |
| 55 C | 62.7053771 | 46.0679285 | 49.9065176 |
| 56 H | 63.4147394 | 46.8084543 | 50.2995200 |
| 57 H | 61.8035322 | 46.5567658 | 49.5236223 |
| 58 H | 62.4289763 | 45.3354715 | 50.6708508 |
| 59 H | 61.5861567 | 46.9204633 | 47.2064118 |
| 60 H | 62.6192473 | 46.0372026 | 45.9295239 |
| 61 H | 63.0254997 | 47.7461894 | 46.3524351 |
| 62 H | 54.5059916 | 46.2093203 | 43.7830936 |
| 63 H | 57.5351184 | 42.3334199 | 48.7184668 |
| 64 H | 52.9312803 | 49.9565432 | 48.7200209 |
| 65 H | 63.1121283 | 43.4044966 | 47.7972298 |

MEL1-IM1-eth

| | | | |
|---|---|---|---|
| 1 N | 56.6293557 | 48.2632039 | 44.4060295 |
| 2 C | 56.4036483 | 46.8977511 | 44.4817581 |
| 3 C | 55.5473346 | 46.1121986 | 43.5387191 |
| 4 N | 57.6058071 | 47.5874880 | 46.2467118 |
| 5 C | 57.0378685 | 46.4966278 | 45.6343523 |
| 6 C | 57.3471787 | 48.6388464 | 45.4799378 |
| 7 H | 55.6985564 | 46.4169597 | 42.4925181 |
| 8 H | 55.8425810 | 45.0519513 | 43.5904591 |
| 9 H | 57.1217489 | 45.4998052 | 46.0574810 |
| 10 H | 57.6712726 | 49.6556368 | 45.6831286 |
| 11 H | 56.2977569 | 48.9272692 | 43.6942201 |
| 12 C | 57.9866010 | 42.4970983 | 49.7235920 |
| 13 C | 57.9778001 | 43.9252708 | 50.2936842 |
| 14 C | 58.9330696 | 44.9269562 | 49.6239201 |
| 15 O | 60.1524771 | 44.7556094 | 49.7254630 |

| | | | |
|---|---|---|---|
| 16 O | 58.3503654 | 45.9136851 | 49.0306673 |
| 17 H | 59.0042417 | 42.0771502 | 49.7954569 |
| 18 H | 57.3293285 | 41.8677658 | 50.3455194 |
| 19 H | 56.9616718 | 44.3417938 | 50.3119905 |
| 20 H | 58.2995033 | 43.8474072 | 51.3465955 |
| 21 N | 54.6210762 | 47.7353504 | 49.7421470 |
| 22 C | 54.6619325 | 49.0890101 | 49.4451657 |
| 23 C | 53.4791783 | 49.9982531 | 49.5780929 |
| 24 N | 56.6633851 | 48.1289662 | 49.0272052 |
| 25 C | 55.9485709 | 49.3102490 | 49.0044514 |
| 26 C | 55.8310401 | 47.1999894 | 49.4739802 |
| 27 H | 52.8645463 | 49.7272794 | 50.4517397 |
| 28 H | 53.8099496 | 51.0349778 | 49.7421409 |
| 29 H | 56.3947712 | 50.2381732 | 48.6551829 |
| 30 H | 56.0733976 | 46.1493103 | 49.5961962 |
| 31 H | 53.8165438 | 47.2049411 | 50.0853197 |
| 32 Fe | 58.6416486 | 47.6571556 | 48.0940342 |
| 33 O | 60.2269272 | 47.1143687 | 47.1707792 |
| 34 C | 59.5431296 | 49.9634765 | 48.6305854 |
| 35 O | 58.7247014 | 49.7579265 | 47.6882011 |
| 36 C | 60.1675407 | 51.3176075 | 48.8082824 |
| 37 C | 61.6340208 | 51.3474961 | 48.3262131 |
| 38 C | 62.1479167 | 52.8059825 | 48.2643563 |
| 39 O | 62.5008724 | 53.2416441 | 47.1510480 |
| 40 O | 62.1335386 | 53.4497394 | 49.3486698 |
| 41 H | 60.1247282 | 51.6256617 | 49.8620604 |
| 42 H | 59.5694537 | 52.0311620 | 48.2231186 |
| 43 H | 62.2650859 | 50.7721171 | 49.0251127 |
| 44 H | 61.7160632 | 50.8967139 | 47.3269378 |
| 45 O | 59.8343543 | 48.9536971 | 49.3687738 |
| 46 C | 63.0571804 | 45.3741341 | 47.9193535 |
| 47 N | 63.2256112 | 45.7105959 | 49.3776807 |
| 48 H | 63.6350718 | 46.1079529 | 47.3414455 |
| 49 H | 61.9930281 | 45.4745871 | 47.6714608 |
| 50 H | 62.8886230 | 44.8863827 | 49.9082010 |
| 51 C | 64.6921131 | 45.8984286 | 49.8057460 |
| 52 H | 65.1971117 | 44.9402849 | 49.5892040 |
| 53 C | 65.3858080 | 47.0373864 | 49.1567039 |
| 54 H | 64.6406157 | 46.0183002 | 50.8975279 |
| 55 C | 62.4017151 | 46.8892788 | 49.7821564 |
| 56 H | 62.5410415 | 47.0729180 | 50.8557856 |
| 57 H | 62.7373377 | 47.7694727 | 49.2180536 |
| 58 H | 61.3523057 | 46.6521348 | 49.5862164 |
| 59 H | 61.0068860 | 47.4540851 | 47.6281390 |
| 60 H | 65.8376479 | 46.9291409 | 48.1690369 |
| 61 H | 65.4787784 | 48.0061128 | 49.6498702 |
| 62 H | 54.4835152 | 46.2210316 | 43.7496949 |
| 63 H | 57.6427834 | 42.3953349 | 48.6942715 |
| 64 H | 52.7995802 | 49.9752451 | 48.7262219 |
| 65 H | 63.3902568 | 44.3633830 | 47.6837491 |

MEL1-TS2-eth

| | | | |
|---|---|---|---|
| 1 N | 56.7428235 | 48.2667739 | 44.5031541 |
| 2 C | 56.4946503 | 46.9069249 | 44.5976834 |

| | | | | | | | |
|---|---|---|---|---|---|---|---|
| 3 C | 55.6350059 | 46.1243519 | 43.6543126 | 30 H | 55.9359901 | 46.1919710 | 49.6547241 |
| 4 N | 57.7545249 | 47.5894253 | 46.3351017 | 31 H | 53.6838503 | 47.2633153 | 50.1189324 |
| 5 C | 57.1453740 | 46.5070392 | 45.7435427 | 32 Fe | 58.5804438 | 47.7651773 | 48.3376578 |
| 6 C | 57.4923872 | 48.6356290 | 45.5614615 | 33 O | 60.3004221 | 47.0049983 | 47.9830626 |
| 7 H | 55.7939130 | 46.4294331 | 42.6088577 | 34 C | 59.3825679 | 50.1231680 | 48.8161422 |
| 8 H | 55.9271201 | 45.0631610 | 43.7071928 | 35 O | 58.8481777 | 49.7762845 | 47.7164144 |
| 9 H | 57.1997865 | 45.5129738 | 46.1800645 | 36 C | 60.0199574 | 51.4825253 | 48.9301391 |
| 10 H | 57.8273352 | 49.6503639 | 45.7580915 | 37 C | 61.4973848 | 51.4418415 | 48.4780322 |
| 11 H | 56.3976488 | 48.9271525 | 43.7946177 | 38 C | 62.0673488 | 52.8707429 | 48.3352285 |
| 12 C | 58.0156600 | 42.4220057 | 49.7771894 | 39 O | 62.4286726 | 53.2391861 | 47.2001120 |
| 13 C | 57.9719404 | 43.8345132 | 50.3860267 | 40 O | 62.0925352 | 53.5627447 | 49.3891198 |
| 14 C | 58.7835152 | 44.9083019 | 49.6525946 | 41 H | 59.9576893 | 51.8621108 | 49.9576407 |
| 15 O | 60.0224165 | 44.7762973 | 49.5566394 | 42 H | 59.4612945 | 52.1683654 | 48.2755615 |
| 16 O | 58.1096289 | 45.8881026 | 49.1876536 | 43 H | 62.0984815 | 50.9061027 | 49.2330434 |
| 17 H | 59.0465202 | 42.0314909 | 49.8182696 | 44 H | 61.5831710 | 50.9160928 | 47.5165741 |
| 18 H | 57.3896550 | 41.7577926 | 50.3950397 | 45 O | 59.4112821 | 49.2586469 | 49.7500461 |
| 19 H | 56.9384815 | 44.1893276 | 50.4986926 | 46 C | 63.0108883 | 45.1521732 | 47.9662303 |
| 20 H | 58.3868371 | 43.7550246 | 51.4055792 | 47 N | 63.9886279 | 46.2507012 | 48.2997650 |
| 21 N | 54.4912774 | 47.7901550 | 49.7788343 | 48 H | 62.3694167 | 45.5213776 | 47.1567688 |
| 22 C | 54.5438445 | 49.1402366 | 49.4717218 | 49 H | 62.3917078 | 44.9746750 | 48.8531797 |
| 23 C | 53.3609993 | 50.0514762 | 49.5744032 | 50 H | 64.7154430 | 46.3187542 | 47.5674201 |
| 24 N | 56.5371520 | 48.1595800 | 49.0683164 | 51 C | 63.2903161 | 47.5978141 | 48.2888732 |
| 25 C | 55.8341778 | 49.3468243 | 49.0352616 | 52 H | 62.9271746 | 47.7438556 | 47.2529799 |
| 26 C | 55.6958641 | 47.2413665 | 49.5180992 | 53 C | 62.1611812 | 47.6962697 | 49.2452041 |
| 27 H | 52.7305596 | 49.7935908 | 50.4404060 | 54 H | 64.0702819 | 48.3650419 | 48.4609267 |
| 28 H | 53.6863772 | 51.0912144 | 49.7286573 | 55 C | 64.7363163 | 46.0096719 | 49.5776156 |
| 29 H | 56.2871570 | 50.2703742 | 48.6815877 | 56 H | 65.3821965 | 46.8763823 | 49.7700742 |

| | | | |
|---|---|---|---|
| 57 H | 64.0124345 | 45.8670593 | 50.3881334 |
| 58 H | 65.3428441 | 45.0963262 | 49.4725981 |
| 59 H | 60.3675473 | 46.1768161 | 48.5172784 |
| 60 H | 61.7101505 | 48.6724955 | 49.4049032 |
| 61 H | 61.9611277 | 46.9381509 | 50.0029638 |
| 62 H | 54.5677220 | 46.2308844 | 43.8482794 |
| 63 H | 57.6605556 | 42.3330188 | 48.7505203 |
| 64 H | 52.7007769 | 50.0082440 | 48.7082020 |
| 65 H | 63.4625923 | 44.2057174 | 47.6691578 |

MEL1-PC-eth

| | | | |
|---|---|---|---|
| 1 N | 56.8861244 | 48.2387375 | 44.4581463 |
| 2 C | 56.5806372 | 46.8934203 | 44.5818064 |
| 3 C | 55.6809665 | 46.1272012 | 43.6616048 |
| 4 N | 57.9445539 | 47.5384146 | 46.2640407 |
| 5 C | 57.2569152 | 46.4816658 | 45.7093357 |
| 6 C | 57.6962725 | 48.5835936 | 45.4832841 |
| 7 H | 55.8275113 | 46.4282364 | 42.6129138 |
| 8 H | 55.9557448 | 45.0609114 | 43.7082607 |
| 9 H | 57.2610517 | 45.4942241 | 46.1657667 |
| 10 H | 58.0723101 | 49.5881641 | 45.6622067 |
| 11 H | 56.5260935 | 48.9066777 | 43.7643039 |
| 12 C | 58.0463819 | 42.4367810 | 49.7585407 |
| 13 C | 58.0093125 | 43.8546583 | 50.3592279 |
| 14 C | 58.8301186 | 44.9197996 | 49.6276232 |
| 15 O | 60.0863529 | 44.8322568 | 49.6460568 |
| 16 O | 58.1894206 | 45.8418191 | 49.0411669 |
| 17 H | 59.0746370 | 42.0396323 | 49.8038102 |
| 18 H | 57.4170305 | 41.7817212 | 50.3823142 |
| 19 H | 56.9772168 | 44.2180909 | 50.4583379 |
| 20 H | 58.4155076 | 43.7747151 | 51.3816586 |
| 21 N | 54.4285534 | 47.7584447 | 49.7506485 |
| 22 C | 54.4832212 | 49.1172004 | 49.4851677 |
| 23 C | 53.3034598 | 50.0312198 | 49.5976831 |
| 24 N | 56.4720039 | 48.1469415 | 49.0435181 |
| 25 C | 55.7730341 | 49.3349682 | 49.0534239 |
| 26 C | 55.6329754 | 47.2162259 | 49.4646752 |
| 27 H | 52.6666247 | 49.7649101 | 50.4565019 |
| 28 H | 53.6325378 | 51.0675505 | 49.7658364 |
| 29 H | 56.2272994 | 50.2688103 | 48.7285147 |
| 30 H | 55.8690545 | 46.1608802 | 49.5666320 |
| 31 H | 53.6239007 | 47.2234922 | 50.0835786 |
| 32 Fe | 58.5058601 | 47.8461375 | 48.3853302 |
| 33 O | 60.7179718 | 47.0253036 | 48.3804558 |
| 34 C | 59.4178617 | 50.1867237 | 48.8286035 |
| 35 O | 58.9480023 | 49.7989410 | 47.7114480 |
| 36 C | 60.0513253 | 51.5521003 | 48.9144330 |
| 37 C | 61.5376261 | 51.4894239 | 48.4996401 |
| 38 C | 62.1122686 | 52.9105228 | 48.3381870 |
| 39 O | 62.4716872 | 53.2664305 | 47.1982458 |
| 40 O | 62.1338797 | 53.6178337 | 49.3826026 |
| 41 H | 59.9677227 | 51.9650932 | 49.9275072 |
| 42 H | 59.5141607 | 52.2185113 | 48.2228759 |
| 43 H | 62.1154351 | 50.9659870 | 49.2818764 |

44 H 61.6408957 50.9423239 47.5517424
45 O 59.4075267 49.3740906 49.8029510
46 C 62.9396666 45.0676297 47.9263474
47 N 63.8575486 46.2113914 48.2713072
48 H 62.2894024 45.3925192 47.1062050
49 H 62.3234141 44.8608600 48.8088570
50 H 64.6092507 46.2946897 47.5654656
51 C 63.1177037 47.5411335 48.2172528
52 H 62.8649725 47.7177576 47.1589020
53 C 61.8331702 47.6181006 49.0388096
54 H 63.8403223 48.3105375 48.5297419
55 C 64.5760272 46.0030019 49.5729484
56 H 65.2001481 46.8841691 49.7705750
57 H 63.8359087 45.8492482 50.3672432
58 H 65.2046297 45.1020028 49.4929842
59 H 60.5271384 46.1352255 48.8171505
60 H 61.6198756 48.6828697 49.1986776
61 H 61.9543593 47.1796287 50.0451066
62 H 54.6170656 46.2474419 43.8658696
63 H 57.6882582 42.3401325 48.7336146
64 H 52.6510280 50.0002913 48.7250760
65 H 63.4390300 44.1425205 47.6384652

MEL2-RC

1 N 54.5917650 48.2305162 46.7358712
2 C 54.3609974 46.8883296 46.9750481
3 C 53.1392719 46.1453132 46.5418707
4 N 56.2902926 47.5284224 47.9335110
5 C 55.4418149 46.4660827 47.7178733
6 C 55.7496416 48.5790961 47.3302179
7 H 52.8299214 46.4267756 45.5253740
8 H 53.3742691 45.0714730 46.4954726
9 H 55.6410938 45.4765817 48.1232700
10 H 56.1730533 49.5797158 47.3283453
11 H 53.9862412 48.8928821 46.2308539
12 C 57.6390462 42.4923224 50.9788065
13 C 57.8619558 43.9386722 51.4491982
14 C 58.4556484 44.8834586 50.3939229
15 O 59.5083744 44.5808693 49.8296877
16 O 57.7731548 45.9607068 50.2047995
17 H 58.5977687 42.0765693 50.6270871
18 H 57.3051359 41.8790230 51.8307459
19 H 56.9449483 44.3784319 51.8591282
20 H 58.5895361 43.9055516 52.2788852
21 N 55.1580414 48.0565962 52.4382753
22 C 55.0253612 49.3694698 52.0179263
23 C 54.0506131 50.3147923 52.6463899
24 N 56.5620452 48.2855726 50.7677087
25 C 55.9187534 49.4927955 50.9751315
26 C 56.0731939 47.4437248 51.6680760
27 H 53.9601389 50.1146769 53.7262725
28 H 54.3979831 51.3546819 52.5548338
29 H 56.1262500 50.3649537 50.3611608
30 H 56.3574124 46.4048538 51.7720645

| | | | |
|---|---|---|---|
| 31 H | 54.6296744 | 47.5792834 | 53.1715693 |
| 32 Fe | 57.9703048 | 47.6909902 | 49.2707887 |
| 33 O | 59.0697452 | 47.1996657 | 48.1821252 |
| 34 C | 58.9270852 | 50.0046080 | 49.4469801 |
| 35 O | 57.9752635 | 49.6689065 | 48.6683107 |
| 36 C | 59.4166228 | 51.4254889 | 49.4286279 |
| 37 C | 60.1103139 | 51.8230695 | 48.1107935 |
| 38 C | 60.3292657 | 53.3622655 | 48.1124205 |
| 39 O | 59.5831266 | 54.0386836 | 47.3616394 |
| 40 O | 61.1901071 | 53.7863234 | 48.9101601 |
| 41 H | 60.0939781 | 51.6117458 | 50.2680385 |
| 42 H | 58.5278188 | 52.0648605 | 49.5623027 |
| 43 H | 61.0889595 | 51.3186637 | 48.0396102 |
| 44 H | 59.4967615 | 51.5340315 | 47.2479610 |
| 45 O | 59.3969574 | 49.0967221 | 50.2002877 |
| 46 C | 62.4954408 | 44.4443408 | 46.8464330 |
| 47 N | 62.8697228 | 45.8190262 | 47.3339906 |
| 48 H | 61.3959744 | 44.4235351 | 46.7664696 |
| 49 H | 62.7810425 | 43.7373552 | 47.6436698 |
| 50 H | 62.4597535 | 46.5060357 | 46.6803555 |
| 51 C | 62.1865265 | 46.0562434 | 48.6699290 |
| 52 H | 62.5739430 | 45.2761727 | 49.3427562 |
| 53 C | 62.3563853 | 47.4561682 | 49.2283490 |
| 54 H | 61.1231919 | 45.8388103 | 48.5141851 |
| 55 C | 64.3429953 | 46.0707971 | 47.3908805 |
| 56 H | 64.7466902 | 46.0179917 | 46.3673186 |
| 57 H | 64.5224168 | 47.0773094 | 47.7838780 |
| 58 H | 64.8011917 | 45.3065017 | 48.0343095 |
| 59 H | 61.6486248 | 47.5742898 | 50.0592803 |
| 60 H | 62.0912990 | 48.2329680 | 48.4962642 |
| 61 H | 63.3684847 | 47.6629710 | 49.6070509 |
| 62 H | 52.2770553 | 46.3138150 | 47.1870503 |
| 63 H | 56.8894384 | 42.3788826 | 50.1956802 |
| 64 H | 53.0365343 | 50.2354276 | 52.2547107 |
| 65 H | 62.9264124 | 44.1162508 | 45.9005538 |

MEL2-TS1-eth-Calpha

| | | | |
|---|---|---|---|
| 1 N | 54.6981334 | 48.1591715 | 46.6331652 |
| 2 C | 54.4494772 | 46.8223418 | 46.8970074 |
| 3 C | 53.1930185 | 46.1024880 | 46.5285950 |
| 4 N | 56.4759445 | 47.4086229 | 47.6821928 |
| 5 C | 55.5757058 | 46.3700883 | 47.5477359 |
| 6 C | 55.9106322 | 48.4750860 | 47.1228474 |
| 7 H | 52.8625434 | 46.3645548 | 45.5130980 |
| 8 H | 53.4044612 | 45.0232865 | 46.5023591 |
| 9 H | 55.7815581 | 45.3787053 | 47.9451426 |
| 10 H | 56.3545983 | 49.4661418 | 47.0940786 |
| 11 H | 54.0709100 | 48.8374826 | 46.1775502 |
| 12 C | 57.6268173 | 42.4707799 | 50.9856459 |
| 13 C | 57.8600003 | 43.9211553 | 51.4410123 |
| 14 C | 58.5180002 | 44.8327436 | 50.3929213 |
| 15 O | 59.6789013 | 44.6122958 | 50.0493067 |
| 16 O | 57.7797637 | 45.7990460 | 49.9584499 |
| 17 H | 58.5853621 | 42.0406271 | 50.6504711 |

| | | | |
|---|---|---|---|
| 18 H | 57.2767356 | 41.8695324 | 51.8399909 |
| 19 H | 56.9324757 | 44.3798155 | 51.8055377 |
| 20 H | 58.5546656 | 43.8853795 | 52.2965357 |
| 21 N | 55.1848496 | 47.9494034 | 52.2796530 |
| 22 C | 55.0921325 | 49.2815436 | 51.9112191 |
| 23 C | 54.1504289 | 50.2355426 | 52.5797508 |
| 24 N | 56.5996107 | 48.2007922 | 50.6130400 |
| 25 C | 55.9893501 | 49.4148819 | 50.8715245 |
| 26 C | 56.0829784 | 47.3428580 | 51.4795218 |
| 27 H | 54.0840606 | 50.0216694 | 53.6592822 |
| 28 H | 54.5145863 | 51.2704054 | 52.4936121 |
| 29 H | 56.2174849 | 50.3074772 | 50.2939743 |
| 30 H | 56.3307427 | 46.2906830 | 51.5365461 |
| 31 H | 54.6562706 | 47.4679688 | 53.0102901 |
| 32 Fe | 58.1322624 | 47.5652916 | 49.0479671 |
| 33 O | 59.4985416 | 46.9774268 | 48.0140343 |
| 34 C | 58.9966079 | 49.9337220 | 49.2614321 |
| 35 O | 58.1313043 | 49.5719881 | 48.4019558 |
| 36 C | 59.4378521 | 51.3723115 | 49.3014790 |
| 37 C | 60.1925964 | 51.8263794 | 48.0388339 |
| 38 C | 60.3570109 | 53.3705988 | 48.0878060 |
| 39 O | 59.5820943 | 54.0573021 | 47.3840635 |
| 40 O | 61.2214941 | 53.7878002 | 48.8899213 |
| 41 H | 60.0524126 | 51.5581359 | 50.1890506 |
| 42 H | 58.5193467 | 51.9767778 | 49.3952851 |
| 43 H | 61.1886141 | 51.3552954 | 48.0078108 |
| 44 H | 59.6377836 | 51.5506317 | 47.1323201 |
| 45 O | 59.4294026 | 49.0440132 | 50.0609676 |
| 46 C | 62.2111465 | 44.5288079 | 46.8795788 |
| 47 N | 62.5995572 | 45.9213878 | 47.3206957 |
| 48 H | 61.1124173 | 44.5138980 | 46.7950189 |
| 49 H | 62.4944535 | 43.8499690 | 47.7026708 |
| 50 H | 62.2771445 | 46.5837700 | 46.5959763 |
| 51 C | 61.8540735 | 46.2808554 | 48.5864813 |
| 52 H | 61.7280234 | 45.3765275 | 49.1948608 |
| 53 C | 62.3265397 | 47.5043391 | 49.3175678 |
| 54 H | 60.6285463 | 46.5582460 | 48.2809525 |
| 55 C | 64.0856122 | 46.1055942 | 47.4517675 |
| 56 H | 64.5411686 | 45.9456647 | 46.4610765 |
| 57 H | 64.2959387 | 47.1322567 | 47.7665775 |
| 58 H | 64.4597500 | 45.3781722 | 48.1843151 |
| 59 H | 61.5625194 | 47.7569661 | 50.0626429 |
| 60 H | 62.4331980 | 48.3829729 | 48.6632555 |
| 61 H | 63.2813383 | 47.3473793 | 49.8506137 |
| 62 H | 52.3460229 | 46.2989820 | 47.1859045 |
| 63 H | 56.8822960 | 42.3604572 | 50.1972399 |
| 64 H | 53.1237065 | 50.1858102 | 52.2172115 |
| 65 H | 62.6621312 | 44.1799067 | 45.9506289 |

MEL2-IM1-eth-Calpha

| | | | |
|---|---|---|---|
| 1 N | 54.6367034 | 48.1774593 | 46.6399702 |
| 2 C | 54.3864698 | 46.8420009 | 46.9091100 |
| 3 C | 53.1376954 | 46.1174272 | 46.5252145 |
| 4 N | 56.3795160 | 47.4476933 | 47.7577067 |

| | | | |
|---|---|---|---|
| 5 C | 55.4913892 | 46.4029199 | 47.6025175 |
| 6 C | 55.8291057 | 48.5053709 | 47.1684838 |
| 7 H | 52.8087789 | 46.3858794 | 45.5112042 |
| 8 H | 53.3535402 | 45.0393216 | 46.4900224 |
| 9 H | 55.6889260 | 45.4167170 | 48.0166708 |
| 10 H | 56.2736352 | 49.4964946 | 47.1407495 |
| 11 H | 54.0209749 | 48.8488126 | 46.1589235 |
| 12 C | 57.6577405 | 42.4644517 | 51.0033203 |
| 13 C | 57.9043179 | 43.8941137 | 51.5161741 |
| 14 C | 58.4637547 | 44.8907921 | 50.4945953 |
| 15 O | 59.6435361 | 44.7867802 | 50.1156803 |
| 16 O | 57.6554153 | 45.8078428 | 50.1118052 |
| 17 H | 58.6104518 | 42.0361144 | 50.6486285 |
| 18 H | 57.3122595 | 41.8394050 | 51.8422545 |
| 19 H | 56.9940580 | 44.3181026 | 51.9582246 |
| 20 H | 58.6480765 | 43.8213805 | 52.3262896 |
| 21 N | 55.0921041 | 47.9982137 | 52.3898723 |
| 22 C | 54.9990468 | 49.3246037 | 52.0026051 |
| 23 C | 54.0441827 | 50.2815809 | 52.6451080 |
| 24 N | 56.4997850 | 48.2236900 | 50.7183201 |
| 25 C | 55.8945403 | 49.4434469 | 50.9608414 |
| 26 C | 55.9848501 | 47.3769693 | 51.5979189 |
| 27 H | 53.9557170 | 50.0778670 | 53.7246862 |
| 28 H | 54.4056500 | 51.3168770 | 52.5564078 |
| 29 H | 56.1241961 | 50.3274640 | 50.3708031 |
| 30 H | 56.2286896 | 46.3241504 | 51.6677392 |
| 31 H | 54.5602297 | 47.5225574 | 53.1213212 |
| 32 Fe | 57.9931734 | 47.6182957 | 49.1627116 |
| 33 O | 59.4299232 | 46.7970008 | 48.2562526 |
| 34 C | 58.9441168 | 49.9465558 | 49.3018451 |
| 35 O | 58.0836218 | 49.5677421 | 48.4365256 |
| 36 C | 59.4132492 | 51.3706329 | 49.3010173 |
| 37 C | 60.1604486 | 51.7955765 | 48.0215580 |
| 38 C | 60.3538457 | 53.3368473 | 48.0606901 |
| 39 O | 59.5938881 | 54.0209983 | 47.3307468 |
| 40 O | 61.2060981 | 53.7572813 | 48.8698036 |
| 41 H | 60.0412497 | 51.5686558 | 50.1760478 |
| 42 H | 58.5058160 | 51.9935969 | 49.3924378 |
| 43 H | 61.1477509 | 51.3049959 | 47.9899021 |
| 44 H | 59.5904475 | 51.5089085 | 47.1277490 |
| 45 O | 59.3297241 | 49.0712677 | 50.1417338 |
| 46 C | 62.4863942 | 44.5008580 | 46.8881695 |
| 47 N | 62.9597472 | 45.8429002 | 47.3977424 |
| 48 H | 61.3879901 | 44.5622295 | 46.8156112 |
| 49 H | 62.7323820 | 43.7669991 | 47.6736078 |
| 50 H | 62.5768719 | 46.5689014 | 46.7658325 |
| 51 C | 62.3867917 | 46.0921347 | 48.7304516 |
| 52 H | 62.0291567 | 45.1957718 | 49.2414144 |
| 53 C | 62.4471535 | 47.4404112 | 49.3363562 |
| 54 H | 59.7689830 | 46.1254961 | 48.8932815 |
| 55 C | 64.4586869 | 46.0227088 | 47.4067033 |
| 56 H | 64.8142486 | 46.0146827 | 46.3634294 |
| 57 H | 64.6926279 | 46.9954166 | 47.8557416 |
| 58 H | 64.9001863 | 45.2024260 | 47.9887427 |

59 H 61.5160414 47.6326910 49.8898307
60 H 62.5507776 48.2439486 48.5921969
61 H 63.2844988 47.5512323 50.0541163
62 H 52.2927880 46.3090988 47.1866225
63 H 56.9085724 42.3730839 50.2168941
64 H 53.0274375 50.2186113 52.2573824
65 H 62.8969562 44.1628927 45.9367046

MEL2-TS2-eth-Calpha
1 N 54.7734588 48.1680344 46.6698253
2 C 54.5023789 46.8352227 46.9315164
3 C 53.2403594 46.1292778 46.5543641
4 N 56.5589369 47.3735756 47.6838781
5 C 55.6296652 46.3583614 47.5627979
6 C 56.0037287 48.4534693 47.1382662
7 H 52.9165886 46.4047865 45.5401847
8 H 53.4457412 45.0491377 46.5158160
9 H 55.8166334 45.3590437 47.9497772
10 H 56.4756203 49.4318709 47.0942572
11 H 54.1505470 48.8571282 46.2235991
12 C 57.6125613 42.3852735 51.0362436
13 C 57.8861865 43.8247197 51.5030919
14 C 58.4708667 44.7428924 50.4310343
15 O 59.5881968 44.4701073 49.9407537
16 O 57.7758905 45.7520585 50.0914522
17 H 58.5586209 41.9265858 50.7036718
18 H 57.2372938 41.7901831 51.8834857
19 H 56.9881751 44.2921591 51.9253387
20 H 58.6283895 43.7728305 52.3168438
21 N 55.1393937 47.9377064 52.2732547
22 C 55.0793684 49.2740986 51.9161246
23 C 54.1452143 50.2367699 52.5824445
24 N 56.5818095 48.1716319 50.6266466
25 C 55.9951179 49.3968387 50.8908559
26 C 56.0332340 47.3188777 51.4788129
27 H 54.0798832 50.0266094 53.6628358
28 H 54.5149593 51.2692076 52.4922695
29 H 56.2539991 50.2947130 50.3335440
30 H 56.2574185 46.2613798 51.5327961
31 H 54.5983692 47.4619905 52.9979754
32 Fe 58.1999445 47.5545890 49.1170918
33 O 59.7179951 46.5350282 48.2792375
34 C 59.1332306 49.8837170 49.1940150
35 O 58.4205305 49.4254077 48.2378013
36 C 59.5171117 51.3342406 49.2093732
37 C 60.2631825 51.8251922 47.9560507
38 C 60.3931051 53.3698811 48.0409228
39 O 59.6239087 54.0456736 47.3129629
40 O 61.2105781 53.7990682 48.8809609
41 H 60.1049746 51.5633027 50.1051760
42 H 58.5688489 51.8975171 49.2863678
43 H 61.2733506 51.3826531 47.9268763
44 H 59.7203943 51.5398742 47.0450498
45 O 59.4448808 49.0664413 50.1196647

46 C 62.2101690 44.5549862 46.9102738
47 N 62.5389260 45.9665650 47.3450123
48 H 61.1169312 44.5037509 46.7891225
49 H 62.4990741 43.8897763 47.7428019
50 H 62.1505746 46.6093556 46.6309992
51 C 61.8539594 46.3016883 48.6260794
52 H 61.7028510 45.4051172 49.2319863
53 C 62.1769109 47.5890007 49.2837439
54 H 59.7205047 45.7093573 48.8398835
55 C 64.0235655 46.2356118 47.4215710
56 H 64.4552520 46.0946761 46.4163666
57 H 64.1820847 47.2745506 47.7262528
58 H 64.4581196 45.5318076 48.1435743
59 H 61.3945089 47.8027146 50.0201384
60 H 62.2052296 48.4391183 48.5856742
61 H 63.1449419 47.5581971 49.8211690
62 H 52.3889505 46.3199180 47.2076837
63 H 56.8729358 42.3045182 50.2396887
64 H 53.1176998 50.1896980 52.2217986
65 H 62.6849331 44.2136120 45.9904206

MEL2-PC-eth-Calpha

1 N 54.6103805 48.1809103 46.5504069
2 C 54.3438468 46.8559075 46.8472762
3 C 53.0836128 46.1356028 46.4945518
4 N 56.3707012 47.4385624 47.6444801
5 C 55.4579013 46.4117272 47.5233087
6 C 55.8236672 48.4926845 47.0484370
7 H 52.7384623 46.3962372 45.4839826
8 H 53.2965076 45.0566141 46.4614252
9 H 55.6459791 45.4252272 47.9421317
10 H 56.2869363 49.4751666 46.9931266
11 H 53.9939122 48.8564134 46.0773816
12 C 57.6598789 42.5219860 50.9391553
13 C 57.9049130 43.9742751 51.3891274
14 C 58.4980526 44.9068684 50.3308994
15 O 59.6707040 44.6825648 49.9357950
16 O 57.7775385 45.8547029 49.9104962
17 H 58.6111842 42.0823962 50.5955478
18 H 57.3207726 41.9318769 51.8051997
19 H 56.9887111 44.4291774 51.7853659
20 H 58.6276490 43.9357063 52.2215235
21 N 54.9834881 47.9994330 52.4533168
22 C 54.8832192 49.3374316 52.1120619
23 C 53.9104034 50.2748175 52.7517546
24 N 56.3933408 48.2979507 50.7987671
25 C 55.7828355 49.5025839 51.0819686
26 C 55.8852932 47.4132017 51.6412202
27 H 53.7890594 50.0486943 53.8232258
28 H 54.2749333 51.3106491 52.6921531
29 H 56.0119010 50.4107282 50.5282764
30 H 56.1399325 46.3590671 51.6764044
31 H 54.4528935 47.4946461 53.1658865
32 Fe 57.7714370 47.7912228 49.2666514

| | | | |
|---|---|---|---|
| 33 O | 60.7572528 | 46.1409478 | 48.1827012 |
| 34 C | 59.1690368 | 49.8763880 | 49.1482495 |
| 35 O | 58.3151878 | 49.4984455 | 48.2701159 |
| 36 C | 59.6332110 | 51.3062625 | 49.1442517 |
| 37 C | 60.2645245 | 51.8133181 | 47.8382384 |
| 38 C | 60.4191457 | 53.3520137 | 47.9495436 |
| 39 O | 59.6558043 | 54.0536423 | 47.2390258 |
| 40 O | 61.2468164 | 53.7560933 | 48.7942041 |
| 41 H | 60.3226567 | 51.4857180 | 49.9748669 |
| 42 H | 58.7252109 | 51.9060953 | 49.3419284 |
| 43 H | 61.2611827 | 51.3580527 | 47.7049111 |
| 44 H | 59.6384177 | 51.5564421 | 46.9740051 |
| 45 O | 59.5207681 | 49.0381638 | 50.0327081 |
| 46 C | 62.5531031 | 44.4325872 | 46.8225061 |
| 47 N | 62.9124212 | 45.8151742 | 47.2943365 |
| 48 H | 61.4641288 | 44.3992750 | 46.6973356 |
| 49 H | 62.8194579 | 43.7396485 | 47.6382628 |
| 50 H | 62.5961191 | 46.4953890 | 46.5840706 |
| 51 C | 62.0898767 | 46.1447635 | 48.5676601 |
| 52 H | 62.3016613 | 45.2939462 | 49.2406809 |
| 53 C | 62.4835475 | 47.4676276 | 49.2015165 |
| 54 H | 60.2380570 | 45.6121724 | 48.9041679 |
| 55 C | 64.3848174 | 46.0146806 | 47.4697963 |
| 56 H | 64.8589653 | 45.8808678 | 46.4854818 |
| 57 H | 64.5795490 | 47.0363211 | 47.8097435 |
| 58 H | 64.7592456 | 45.2738037 | 48.1895282 |
| 59 H | 61.7236193 | 47.6885825 | 49.9620777 |
| 60 H | 62.4555987 | 48.3018856 | 48.4853714 |
| 61 H | 63.4680533 | 47.4376628 | 49.6890472 |
| 62 H | 52.2496520 | 46.3335566 | 47.1678948 |
| 63 H | 56.9044214 | 42.4012861 | 50.1627646 |
| 64 H | 52.9094502 | 50.2162246 | 52.3242890 |
| 65 H | 62.9975694 | 44.0966609 | 45.8856655 |

MEL2-RC

| | | | |
|---|---|---|---|
| 1 N | 54.5917650 | 48.2305162 | 46.7358712 |
| 2 C | 54.3609974 | 46.8883296 | 46.9750481 |
| 3 C | 53.1392719 | 46.1453132 | 46.5418707 |
| 4 N | 56.2902926 | 47.5284224 | 47.9335110 |
| 5 C | 55.4418149 | 46.4660827 | 47.7178733 |
| 6 C | 55.7496416 | 48.5790961 | 47.3302179 |
| 7 H | 52.8299214 | 46.4267756 | 45.5253740 |
| 8 H | 53.3742691 | 45.0714730 | 46.4954726 |
| 9 H | 55.6410938 | 45.4765817 | 48.1232700 |
| 10 H | 56.1730533 | 49.5797158 | 47.3283453 |
| 11 H | 53.9862412 | 48.8928821 | 46.2308539 |
| 12 C | 57.6390462 | 42.4923224 | 50.9788065 |
| 13 C | 57.8619558 | 43.9386722 | 51.4491982 |
| 14 C | 58.4556484 | 44.8834586 | 50.3939229 |
| 15 O | 59.5083744 | 44.5808693 | 49.8296877 |
| 16 O | 57.7731548 | 45.9607068 | 50.2047995 |
| 17 H | 58.5977687 | 42.0765693 | 50.6270871 |
| 18 H | 57.3051359 | 41.8790230 | 51.8307459 |
| 19 H | 56.9449483 | 44.3784319 | 51.8591282 |

20 H 58.5895361 43.9055516 52.2788852
21 N 55.1580414 48.0565962 52.4382753
22 C 55.0253612 49.3694698 52.0179263
23 C 54.0506131 50.3147923 52.6463899
24 N 56.5620452 48.2855726 50.7677087
25 C 55.9187534 49.4927955 50.9751315
26 C 56.0731939 47.4437248 51.6680760
27 H 53.9601389 50.1146769 53.7262725
28 H 54.3979831 51.3546819 52.5548338
29 H 56.1262500 50.3649537 50.3611608
30 H 56.3574124 46.4048538 51.7720645
31 H 54.6296744 47.5792834 53.1715693
32 Fe 57.9703048 47.6909902 49.2707887
33 O 59.0697452 47.1996657 48.1821252
34 C 58.9270852 50.0046080 49.4469801
35 O 57.9752635 49.6689065 48.6683107
36 C 59.4166228 51.4254889 49.4286279
37 C 60.1103139 51.8230695 48.1107935
38 C 60.3292657 53.3622655 48.1124205
39 O 59.5831266 54.0386836 47.3616394
40 O 61.1901071 53.7863234 48.9101601
41 H 60.0939781 51.6117458 50.2680385
42 H 58.5278188 52.0648605 49.5623027
43 H 61.0889595 51.3186637 48.0396102
44 H 59.4967615 51.5340315 47.2479610
45 O 59.3969574 49.0967221 50.2002877
46 C 62.4954408 44.4443408 46.8464330
47 N 62.8697228 45.8190262 47.3339906
48 H 61.3959744 44.4235351 46.7664696
49 H 62.7810425 43.7373552 47.6436698
50 H 62.4597535 46.5060357 46.6803555
51 C 62.1865265 46.0562434 48.6699290
52 H 62.5739430 45.2761727 49.3427562
53 C 62.3563853 47.4561682 49.2283490
54 H 61.1231919 45.8388103 48.5141851
55 C 64.3429953 46.0707971 47.3908805
56 H 64.7466902 46.0179917 46.3673186
57 H 64.5224168 47.0773094 47.7838780
58 H 64.8011917 45.3065017 48.0343095
59 H 61.6486248 47.5742898 50.0592803
60 H 62.0912990 48.2329680 48.4962642
61 H 63.3684847 47.6629710 49.6070509
62 H 52.2770553 46.3138150 47.1870503
63 H 56.8894384 42.3788826 50.1956802
64 H 53.0365343 50.2354276 52.2547107
65 H 62.9264124 44.1162508 45.9005538

MEL2-TS1-eth-Cbeta

1 N 54.6557425 48.2028380 46.6605471
2 C 54.4237826 46.8647578 46.9329666
3 C 53.1867335 46.1229487 46.5415050
4 N 56.4055099 47.5040312 47.7853013
5 C 55.5344786 46.4440382 47.6299407
6 C 55.8405722 48.5514976 47.1919962

| | | | | | | | |
|---|---|---|---|---|---|---|---|
| 7 H | 52.8683791 | 46.3805386 | 45.5210189 | 34 C | 58.8726445 | 50.0779879 | 49.3728021 |
| 8 H | 53.4135925 | 45.0467142 | 46.5194552 | 35 O | 57.9333677 | 49.7412214 | 48.5835243 |
| 9 H | 55.7456677 | 45.4635077 | 48.0504492 | 36 C | 59.3621727 | 51.4934316 | 49.3778355 |
| 10 H | 56.2644471 | 49.5514825 | 47.1692970 | 37 C | 60.1478383 | 51.8714710 | 48.1042194 |
| 11 H | 54.0316247 | 48.8645217 | 46.1770463 | 38 C | 60.3475194 | 53.4114430 | 48.1006149 |
| 12 C | 57.5986430 | 42.5356587 | 51.0064054 | 39 O | 59.5828247 | 54.0773087 | 47.3593157 |
| 13 C | 57.7977696 | 43.9891720 | 51.4726904 | 40 O | 61.2050385 | 53.8497223 | 48.8941332 |
| 14 C | 58.4866484 | 44.9215858 | 50.4633164 | 41 H | 59.9796619 | 51.6885475 | 50.2614938 |
| 15 O | 59.6596008 | 44.7021748 | 50.1571435 | 42 H | 58.4685151 | 52.1366894 | 49.4334299 |
| 16 O | 57.7691481 | 45.9035695 | 50.0315985 | 43 H | 61.1306543 | 51.3717177 | 48.1107544 |
| 17 H | 58.5681055 | 42.1261445 | 50.6768814 | 44 H | 59.5940808 | 51.5645604 | 47.2067721 |
| 18 H | 57.2560834 | 41.9246460 | 51.8570297 | 45 O | 59.3486247 | 49.1591964 | 50.1214716 |
| 19 H | 56.8523078 | 44.4353206 | 51.8046947 | 46 C | 62.4351533 | 44.3219690 | 46.8699913 |
| 20 H | 58.4604908 | 43.9563229 | 52.3534265 | 47 N | 62.6945295 | 45.7347649 | 47.3253284 |
| 21 N | 55.1201689 | 48.0018868 | 52.4010221 | 48 H | 61.3379991 | 44.2192619 | 46.8392311 |
| 22 C | 55.0011851 | 49.3300682 | 52.0252002 | 49 H | 62.8130922 | 43.6507681 | 47.6588757 |
| 23 C | 54.0298825 | 50.2691383 | 52.6686778 | 50 H | 62.1237172 | 46.3510462 | 46.7193044 |
| 24 N | 56.5340398 | 48.2769828 | 50.7414181 | 51 C | 62.1777277 | 45.8980918 | 48.7440350 |
| 25 C | 55.8996487 | 49.4795092 | 50.9907881 | 52 H | 63.0036031 | 45.5739713 | 49.3987705 |
| 26 C | 56.0344038 | 47.4101204 | 51.6089944 | 53 C | 61.7009697 | 47.2868047 | 49.0637387 |
| 27 H | 53.9303493 | 50.0509466 | 53.7443885 | 54 H | 61.3504330 | 45.1927579 | 48.8948095 |
| 28 H | 54.3850666 | 51.3079159 | 52.5969482 | 55 C | 64.1203172 | 46.1840209 | 47.2330665 |
| 29 H | 56.1133496 | 50.3712063 | 50.4071373 | 56 H | 64.4458625 | 46.1906284 | 46.1801771 |
| 30 H | 56.3068947 | 46.3644440 | 51.6749418 | 57 H | 64.1948602 | 47.2043489 | 47.6286584 |
| 31 H | 54.5911198 | 47.5079649 | 53.1227297 | 58 H | 64.7358663 | 45.4877998 | 47.8189046 |
| 32 Fe | 58.0224087 | 47.7138803 | 49.1717738 | 59 H | 61.4503846 | 47.3877119 | 50.1251373 |
| 33 O | 59.4123999 | 47.2172991 | 48.0361748 | 60 H | 60.6036122 | 47.4216867 | 48.5377338 |

61 H 62.3200546 48.1290836 48.7211874
62 H 52.3277567 46.3104616 47.1857603
63 H 56.8604698 42.4100722 50.2143224
64 H 53.0186696 50.2046576 52.2669841
65 H 62.8505469 44.0299898 45.9054896

MEL2-IM1-eth-Cbeta

1 N 54.6131117 48.1939321 46.6364285
2 C 54.3791465 46.8561560 46.9099851
3 C 53.1367400 46.1186965 46.5289437
4 N 56.3634279 47.4916662 47.7569685
5 C 55.4902295 46.4333461 47.6039473
6 C 55.7992497 48.5403890 47.1651646
7 H 52.8120136 46.3749641 45.5102677
8 H 53.3590325 45.0415292 46.5082186
9 H 55.7022890 45.4519226 48.0220873
10 H 56.2229727 49.5406853 47.1446106
11 H 53.9906145 48.8571097 46.1531896
12 C 57.6102177 42.5673469 50.9964410
13 C 57.8231681 44.0172635 51.4673512
14 C 58.5000455 44.9603022 50.4584291
15 O 59.6936166 44.8052618 50.2007487
16 O 57.7417564 45.8884097 49.9720825
17 H 58.5754268 42.1494017 50.6650374
18 H 57.2672462 41.9584915 51.8482714
19 H 56.8811083 44.4605240 51.8134899
20 H 58.4957371 43.9741850 52.3395964
21 N 55.1073331 47.9924477 52.3946232
22 C 54.9899842 49.3224367 52.0241641
23 C 54.0207675 50.2618644 52.6703299
24 N 56.5206070 48.2724346 50.7355450
25 C 55.8879798 49.4751437 50.9901152
26 C 56.0199400 47.4024287 51.5995232
27 H 53.9209675 50.0413196 53.7455280
28 H 54.3786309 51.2998110 52.6007810
29 H 56.1016684 50.3685430 50.4092324
30 H 56.2879078 46.3548957 51.6587469
31 H 54.5792584 47.4968967 53.1160702
32 Fe 57.9740374 47.6994130 49.1401346
33 O 59.4693539 47.2714259 48.0283406
34 C 58.8516731 50.0690047 49.3602735
35 O 57.8789957 49.7485431 48.6077710
36 C 59.3431186 51.4836644 49.3717843
37 C 60.1294742 51.8587206 48.0965141
38 C 60.3383055 53.3976424 48.0953613
39 O 59.5771402 54.0677241 47.3539195
40 O 61.1982229 53.8306504 48.8887668
41 H 59.9661552 51.6745141 50.2527463
42 H 58.4527132 52.1307648 49.4290570
43 H 61.1091645 51.3529078 48.1005518
44 H 59.5726172 51.5574430 47.1987596
45 O 59.3688805 49.1317887 50.0624589
46 C 62.4497788 44.3742920 46.8619554
47 N 62.8488599 45.7456235 47.3474202

48 H 61.3478489 44.3735088 46.8229760
49 H 62.7587784 43.6593484 47.6428510
50 H 62.3572067 46.4352145 46.7542963
51 C 62.3229139 45.9309733 48.7593242
52 H 62.9149593 45.2263009 49.3770022
53 C 62.3738233 47.3289761 49.2457128
54 H 61.2887175 45.5560515 48.7953103
55 C 64.3172023 46.0313913 47.2765889
56 H 64.6357927 46.0381680 46.2217561
57 H 64.5141445 47.0201734 47.7042223
58 H 64.8461623 45.2492123 47.8388630
59 H 61.6857435 47.6180152 50.0405019
60 H 60.2073124 47.7254428 48.4677342
61 H 63.1147263 48.0610270 48.9175078
62 H 52.2851414 46.3123038 47.1811425
63 H 56.8682264 42.4437003 50.2076270
64 H 53.0091034 50.2017009 52.2691026
65 H 62.8512760 44.0605290 45.8984099

MEL2-TS2-eth-Cbeta

1 N 54.6656210 48.1963427 46.6452708
2 C 54.4305037 46.8603105 46.9248140
3 C 53.1875809 46.1226452 46.5436995
4 N 56.4287720 47.4923315 47.7505237
5 C 55.5481057 46.4369147 47.6093000
6 C 55.8590912 48.5400236 47.1607912
7 H 52.8668913 46.3772355 45.5230126
8 H 53.4087103 45.0451379 46.5264522
9 H 55.7601012 45.4559109 48.0282929
10 H 56.2836062 49.5394479 47.1334216
11 H 54.0391166 48.8590883 46.1662495
12 C 57.5728706 42.5650851 51.0181962
13 C 57.7697567 44.0263598 51.4634696
14 C 58.4910270 44.9241907 50.4469229
15 O 59.6717789 44.6905869 50.1914358
16 O 57.7957048 45.8872516 49.9350457
17 H 58.5448850 42.1496546 50.7049663
18 H 57.2199858 41.9668958 51.8734241
19 H 56.8191479 44.4825944 51.7652104
20 H 58.4127402 44.0061874 52.3589015
21 N 55.1125172 47.9834575 52.3696286
22 C 54.9995629 49.3169539 52.0105655
23 C 54.0330131 50.2553599 52.6621780
24 N 56.5304621 48.2752540 50.7160761
25 C 55.8998539 49.4763404 50.9796032
26 C 56.0260080 47.3990058 51.5708675
27 H 53.9353785 50.0320440 53.7370359
28 H 54.3919416 51.2930847 52.5940903
29 H 56.1153232 50.3746307 50.4069969
30 H 56.2927190 46.3510450 51.6237129
31 H 54.5834109 47.4841156 53.0879233
32 Fe 58.0398449 47.7228404 49.1666902
33 O 59.6337642 47.3749638 48.1214999
34 C 58.8311694 50.1287644 49.3863106

35 O 57.9140090 49.7652931 48.5842325

36 C 59.3001729 51.5491185 49.3857705

37 C 60.1284681 51.8957358 48.1289885

38 C 60.3336585 53.4343085 48.1063506

39 O 59.5651629 54.0911054 47.3610679

40 O 61.1933828 53.8793374 48.8924948

41 H 59.8905482 51.7669336 50.2828655

42 H 58.4031264 52.1887289 49.3914159

43 H 61.1088919 51.3930395 48.1758228

44 H 59.5998680 51.5757514 47.2204324

45 O 59.3336715 49.2190933 50.1309897

46 C 62.4160407 44.3080454 46.8667668

47 N 62.6912095 45.7095505 47.3419661

48 H 61.3183972 44.2236224 46.8167781

49 H 62.7704407 43.6224035 47.6544572

50 H 62.1374820 46.3442943 46.7403775

51 C 62.1440830 45.8615328 48.7501985

52 H 62.9067740 45.3910749 49.4031454

53 C 61.8127368 47.2552593 49.1222236

54 H 61.2440173 45.2406106 48.8440118

55 C 64.1255273 46.1334135 47.2787212

56 H 64.4654426 46.1517225 46.2303488

57 H 64.2144138 47.1441262 47.6942023

58 H 64.7189603 45.4160450 47.8622413

59 H 61.2807366 47.3886987 50.0620167

60 H 60.1874071 48.1487208 48.2980122

61 H 62.4114394 48.1034925 48.7685614

62 H 52.3303457 46.3157898 47.1886104

63 H 56.8409582 42.4327958 50.2214083

64 H 53.0205161 50.1976371 52.2626969

65 H 62.8412876 44.0178628 45.9060242

MEL2-PC-eth-Cbeta

1 N 54.6860570 48.2100916 46.6208820

2 C 54.4139626 46.8880106 46.9221359

3 C 53.1698857 46.1580225 46.5303627

4 N 56.4206630 47.4799632 47.7673742

5 C 55.5111699 46.4520830 47.6320186

6 C 55.8883757 48.5265769 47.1463723

7 H 52.8478055 46.4291251 45.5148917

8 H 53.3937296 45.0813495 46.4902625

9 H 55.6854239 45.4690024 48.0650112

10 H 56.3535312 49.5074087 47.0808742

11 H 54.0769151 48.8794310 46.1289756

12 C 57.6101432 42.4653793 50.9978794

13 C 57.8443105 43.9233802 51.4289195

14 C 58.4665537 44.8163735 50.3483780

15 O 59.4823462 44.4129433 49.7579227

16 O 57.8852376 45.9299783 50.1279130

17 H 58.5685879 42.0320077 50.6681372

18 H 57.2555742 41.8765070 51.8584936

19 H 56.9282772 44.3933692 51.8078477

20 H 58.5555673 43.9073923 52.2735751

21 N 54.9483485 48.0163829 52.5188629

| | | | |
|---|---|---|---|
| 22 C | 54.8387239 | 49.3517948 | 52.1741427 |
| 23 C | 53.8512970 | 50.2849987 | 52.7949999 |
| 24 N | 56.3699354 | 48.3247196 | 50.8766477 |
| 25 C | 55.7467270 | 49.5230128 | 51.1530865 |
| 26 C | 55.8645373 | 47.4373067 | 51.7170872 |
| 27 H | 53.7112931 | 50.0609985 | 53.8644319 |
| 28 H | 54.2137658 | 51.3215180 | 52.7395989 |
| 29 H | 55.9717815 | 50.4370211 | 50.6074713 |
| 30 H | 56.1407916 | 46.3889233 | 51.7643801 |
| 31 H | 54.4143537 | 47.5045907 | 53.2233871 |
| 32 Fe | 57.8102603 | 47.7869638 | 49.4095894 |
| 33 O | 60.4010458 | 47.5911613 | 47.7011903 |
| 34 C | 59.0694492 | 50.0224145 | 49.3688564 |
| 35 O | 58.5371612 | 49.4409202 | 48.3370966 |
| 36 C | 59.4001998 | 51.4832570 | 49.2887342 |
| 37 C | 60.2298362 | 51.9025337 | 48.0643522 |
| 38 C | 60.3618855 | 53.4477950 | 48.0863831 |
| 39 O | 59.5657411 | 54.0940136 | 47.3601364 |
| 40 O | 61.1993603 | 53.9116742 | 48.8872381 |
| 41 H | 59.8997620 | 51.8023347 | 50.2110265 |
| 42 H | 58.4313765 | 52.0151402 | 49.2437416 |
| 43 H | 61.2353768 | 51.4525205 | 48.1217362 |
| 44 H | 59.7402099 | 51.5745362 | 47.1367778 |
| 45 O | 59.2432950 | 49.3229967 | 50.3986543 |
| 46 C | 62.4665165 | 44.3644399 | 46.8525160 |
| 47 N | 62.6799515 | 45.8022163 | 47.2441975 |
| 48 H | 61.3750841 | 44.2244727 | 46.7858396 |
| 49 H | 62.8359552 | 43.7344710 | 47.6795030 |
| 50 H | 62.1873150 | 46.3708388 | 46.5354180 |
| 51 C | 61.9985113 | 46.0823739 | 48.5717840 |
| 52 H | 62.7392051 | 45.8891815 | 49.3597094 |
| 53 C | 61.4366233 | 47.4865437 | 48.6653284 |
| 54 H | 61.1755256 | 45.3663032 | 48.6923125 |
| 55 C | 64.1120983 | 46.2387149 | 47.2722039 |
| 56 H | 64.5286882 | 46.1933435 | 46.2528382 |
| 57 H | 64.1581712 | 47.2774196 | 47.6218292 |
| 58 H | 64.6615740 | 45.5677743 | 47.9463435 |
| 59 H | 61.0423107 | 47.6167966 | 49.6862870 |
| 60 H | 59.8677585 | 48.4125663 | 47.8167182 |
| 61 H | 62.2114348 | 48.2614607 | 48.5132434 |
| 62 H | 52.3141451 | 46.3379308 | 47.1810610 |
| 63 H | 56.8697613 | 42.3460622 | 50.2068894 |
| 64 H | 52.8596866 | 50.2201806 | 52.3471444 |
| 65 H | 62.9081564 | 44.0508248 | 45.9066476 |

MEL-OH-RC

| | | | |
|---|---|---|---|
| 1 N | 57.6745108 | 49.7740361 | 49.5260611 |
| 2 C | 57.1905986 | 48.6275792 | 50.1297017 |
| 3 C | 55.8748219 | 48.5479801 | 50.8366016 |
| 4 N | 59.2574200 | 48.2791528 | 49.3359686 |
| 5 C | 58.1944321 | 47.6956610 | 49.9867906 |
| 6 C | 58.9117333 | 49.5308676 | 49.0701868 |
| 7 H | 55.0750364 | 49.0573971 | 50.2787774 |
| 8 H | 55.5705398 | 47.4929648 | 50.9193350 |

| | | | |
|---|---|---|---|
| 9 H | 58.2189747 | 46.6602052 | 50.3161031 |
| 10 H | 59.5280525 | 50.2545524 | 48.5475792 |
| 11 H | 57.2194153 | 50.6946935 | 49.4639621 |
| 12 C | 61.5358095 | 43.9620988 | 52.6581465 |
| 13 C | 62.3269289 | 45.2589528 | 52.4014853 |
| 14 C | 62.2895954 | 45.6638726 | 50.9223076 |
| 15 O | 62.8817961 | 44.9804754 | 50.0752461 |
| 16 O | 61.5752969 | 46.6960447 | 50.6661694 |
| 17 H | 61.9764835 | 43.1546246 | 52.0514657 |
| 18 H | 61.6221222 | 43.6499076 | 53.7104403 |
| 19 H | 61.9605642 | 46.0902493 | 53.0218378 |
| 20 H | 63.3801814 | 45.0711000 | 52.6687804 |
| 21 N | 62.9684985 | 51.0583121 | 50.4242529 |
| 22 C | 62.4043371 | 50.5567726 | 51.5886233 |
| 23 C | 62.5507900 | 51.2142713 | 52.9280303 |
| 24 N | 61.9273171 | 49.2216939 | 49.8389825 |
| 25 C | 61.7523953 | 49.4162423 | 51.1935278 |
| 26 C | 62.6682496 | 50.2302193 | 49.3988180 |
| 27 H | 63.0027638 | 50.5316210 | 53.6650808 |
| 28 H | 63.2331755 | 52.0708339 | 52.8397574 |
| 29 H | 61.1756728 | 48.7208810 | 51.7938636 |
| 30 H | 63.0110043 | 50.3884903 | 48.3771882 |
| 31 H | 63.5423638 | 51.8956957 | 50.3415343 |
| 32 Fe | 61.1324897 | 47.5060733 | 48.9009018 |
| 33 O | 60.5290024 | 46.1364855 | 48.2847421 |
| 34 C | 62.0699164 | 48.2215427 | 46.6609806 |
| 35 O | 60.9202334 | 48.6287935 | 46.9783115 |
| 36 C | 62.6353440 | 48.3623012 | 45.2715677 |
| 37 C | 62.1439488 | 49.5394757 | 44.4288552 |
| 38 C | 63.0458081 | 50.7969796 | 44.4770747 |
| 39 O | 62.5395044 | 51.8753439 | 44.1028182 |
| 40 O | 64.2468552 | 50.6346959 | 44.8240102 |
| 41 H | 62.4024361 | 47.3932158 | 44.7942439 |
| 42 H | 63.7299122 | 48.4068605 | 45.3386323 |
| 43 H | 62.1456745 | 49.2385483 | 43.3695356 |
| 44 H | 61.1041860 | 49.8105206 | 44.6607749 |
| 45 O | 62.7323213 | 47.5910528 | 47.5653836 |
| 46 C | 61.0952637 | 42.0127360 | 44.3525245 |
| 47 N | 61.7754886 | 42.8625820 | 45.3890373 |
| 48 H | 61.7809976 | 41.1859610 | 44.1133566 |
| 49 H | 60.9383804 | 42.6293954 | 43.4545034 |
| 50 H | 61.8036844 | 42.3069442 | 46.2656809 |
| 51 C | 63.2186161 | 43.0731614 | 44.9982500 |
| 52 H | 63.2235440 | 43.5164216 | 43.9912503 |
| 53 C | 63.9953652 | 43.9748116 | 45.9438330 |
| 54 H | 63.6475472 | 42.0622977 | 44.9497942 |
| 55 C | 61.0399599 | 44.1425907 | 45.6615443 |
| 56 H | 60.0027141 | 43.8934814 | 45.9223752 |
| 57 H | 61.5115674 | 44.6787509 | 46.4902149 |
| 58 H | 61.0672816 | 44.7571796 | 44.7530225 |
| 59 H | 63.7074216 | 43.7560680 | 46.9943006 |
| 60 H | 64.3145642 | 45.9007191 | 46.1276241 |
| 61 H | 65.0666386 | 43.7158548 | 45.8645415 |
| 62 O | 63.7605177 | 45.3054963 | 45.5763812 |

63 H 55.8935161 49.0062679 51.8253837

64 H 60.4728703 44.0554421 52.4356283

65 H 61.6209639 51.5693627 53.3723283

66 H 60.1441908 41.6055038 44.6955836

MEL-OH-TS1-Cbeta

1 N 57.9701099 49.6381102 49.3982819

2 C 57.4625411 48.5088925 50.0171386

3 C 56.1324023 48.4555928 50.7009369

4 N 59.4889549 48.0700583 49.1394564

5 C 58.4266008 47.5395701 49.8437380

6 C 59.1763267 49.3397464 48.8903025

7 H 55.3590593 48.9830408 50.1219995

8 H 55.8082575 47.4055572 50.7701000

9 H 58.4143870 46.5078128 50.1869275

10 H 59.8007209 50.0377438 48.3417576

11 H 57.5373192 50.5726020 49.3565739

12 C 61.5757359 43.8332947 52.6654560

13 C 62.3598504 45.1197791 52.3568173

14 C 62.3919559 45.4181490 50.8491464

15 O 62.9287987 44.6077908 50.0872371

16 O 61.8069079 46.5127251 50.5004889

17 H 62.0153318 43.0071061 52.0849436

18 H 61.6576999 43.5561926 53.7277034

19 H 61.9676271 45.9888553 52.9054138

20 H 63.4039781 44.9653634 52.6785452

21 N 63.0369784 51.0701166 50.4758461

22 C 62.4981365 50.5009892 51.6200812

23 C 62.5850157 51.1537558 52.9669801

24 N 62.1802195 49.1535682 49.8398378

25 C 61.9616881 49.3096181 51.1930228

26 C 62.8356311 50.2320483 49.4340435

27 H 63.0084543 50.4666680 53.7164692

28 H 63.2725540 52.0094030 52.9106246

29 H 61.4400195 48.5514419 51.7689519

30 H 63.1934101 50.4315430 48.4250212

31 H 63.5373627 51.9549917 50.4205699

32 Fe 61.4781534 47.3818701 48.7310744

33 O 61.3033881 45.8641914 47.8455351

34 C 62.3697326 48.3250970 46.5409865

35 O 61.1823132 48.5970516 46.8829607

36 C 62.8259030 48.4216861 45.1108753

37 C 62.3002215 49.6090215 44.3074525

38 C 63.1599458 50.8862959 44.4351293

39 O 62.6260604 51.9678318 44.1149567

40 O 64.3588567 50.7363185 44.7945203

41 H 62.4979184 47.4616887 44.6711508

42 H 63.9212475 48.4217616 45.0834250

43 H 62.3237655 49.3501009 43.2375112

44 H 61.2490268 49.8334894 44.5392183

45 O 63.1363731 47.8506068 47.4490175

46 C 60.9366748 41.8663525 44.8286486

47 N 61.2452239 43.0672889 45.6826047

48 H 61.7166109 41.1077358 44.9908889

49 H 60.9613550 42.2043680 43.7825734

50 H 60.9880858 42.8545408 46.6657997

51 C 62.7261234 43.3172277 45.7065687

52 H 63.0430708 43.4748761 44.6657059

53 C 63.1885986 44.5177864 46.5347114

54 H 63.1601207 42.3848858 46.0942108

55 C 60.4704400 44.2705502 45.2288725

56 H 59.4053362 44.0014021 45.1422245

57 H 60.5889607 45.0768935 45.9617492

58 H 60.8651588 44.5795624 44.2488717

59 H 62.2800416 45.1051440 47.0854659

60 H 64.4986374 45.9418472 46.2185763

61 H 63.7592360 44.1908712 47.4182638

62 O 63.8912639 45.3571757 45.7005729

63 H 56.1125795 48.9069789 51.6928667

64 H 60.5146362 43.9317551 52.4364577

65 H 61.6426617 51.5121266 53.3812341

66 H 59.9543810 41.4509802 45.0536641

MEL-OH-IM1-Cbeta

1 N 57.9167395 49.7561775 49.5007706

2 C 57.4031401 48.6064893 50.0741983

3 C 56.0613510 48.5285862 50.7318714

4 N 59.4775028 48.2282961 49.2930129

5 C 58.3927143 47.6592431 49.9269635

6 C 59.1523842 49.4936547 49.0508352

7 H 55.2896220 49.0563051 50.1514691

8 H 55.7472780 47.4744025 50.7844540

9 H 58.3855952 46.6171887 50.2372044

10 H 59.7888002 50.2185972 48.5531937

11 H 57.4687606 50.6820021 49.4468224

12 C 61.5174381 43.7995256 52.6233721

13 C 62.3547716 45.0707887 52.4027609

14 C 62.4000050 45.4796676 50.9282992

15 O 62.7427550 44.6304777 50.0767127

16 O 62.0355345 46.6723163 50.6575925

17 H 61.9438786 42.9900785 52.0113628

18 H 61.5675607 43.4704548 53.6726665

19 H 62.0015193 45.9216126 53.0040779

20 H 63.3901178 44.8513357 52.7177228

21 N 63.0811906 51.1916503 50.5758285

22 C 62.5266674 50.6198950 51.7113284

23 C 62.5893929 51.2540420 53.0658896

24 N 62.1865677 49.3003556 49.9183135

25 C 61.9689526 49.4461544 51.2712420

26 C 62.8645374 50.3686257 49.5239209

27 H 63.0020864 50.5590065 53.8138996

28 H 63.2730734 52.1115308 53.0310586

29 H 61.4364179 48.6927571 51.8425070

30 H 63.2265500 50.5712763 48.5162698

31 H 63.5933580 52.0701488 50.5302561

32 Fe 61.4395639 47.5197318 48.8647441

33 O 61.1463862 45.7934359 48.2298023

34 C 62.3192946 48.2436596 46.6103617

| | | | |
|---|---|---|---|
| 35 O | 61.1356889 | 48.5289477 | 46.9733045 |
| 36 C | 62.7484375 | 48.3296147 | 45.1706795 |
| 37 C | 62.2238435 | 49.5234647 | 44.3707226 |
| 38 C | 63.1061519 | 50.7915050 | 44.4489507 |
| 39 O | 62.5828279 | 51.8703826 | 44.1011767 |
| 40 O | 64.3083751 | 50.6363695 | 44.7941024 |
| 41 H | 62.3994693 | 47.3776499 | 44.7304729 |
| 42 H | 63.8436012 | 48.3176476 | 45.1230391 |
| 43 H | 62.2099708 | 49.2533638 | 43.3034929 |
| 44 H | 61.1838264 | 49.7681442 | 44.6306592 |
| 45 O | 63.0985701 | 47.7868429 | 47.5133444 |
| 46 C | 61.0579067 | 41.9406983 | 44.2758246 |
| 47 N | 61.7475943 | 42.8721322 | 45.2204416 |
| 48 H | 61.7283221 | 41.0823933 | 44.1180570 |
| 49 H | 60.9030873 | 42.4665296 | 43.3199222 |
| 50 H | 61.7377199 | 42.4272540 | 46.1578894 |
| 51 C | 63.2401948 | 42.9896888 | 44.8289935 |
| 52 H | 63.2333691 | 43.3618485 | 43.7923385 |
| 53 C | 64.0452460 | 43.8728190 | 45.6809127 |
| 54 H | 63.5927355 | 41.9473066 | 44.8438747 |
| 55 C | 61.0921998 | 44.2133681 | 45.3038198 |
| 56 H | 60.0142543 | 44.0690168 | 45.4570866 |
| 57 H | 61.4992157 | 44.7804307 | 46.1496737 |
| 58 H | 61.2702681 | 44.7419513 | 44.3560288 |
| 59 H | 61.8437123 | 45.2766904 | 48.7041741 |
| 60 H | 64.6195604 | 45.7087375 | 45.9578929 |
| 61 H | 64.4778748 | 43.5608835 | 46.6355592 |
| 62 O | 64.0751520 | 45.1699350 | 45.3256407 |
| 63 H | 56.0353479 | 48.9701874 | 51.7280538 |
| 64 H | 60.4641328 | 43.9326978 | 52.3766328 |
| 65 H | 61.6318467 | 51.5957236 | 53.4588605 |
| 66 H | 60.1041710 | 41.5827906 | 44.6635840 |

MEL-OH-TS2-Cbeta

| | | | |
|---|---|---|---|
| 1 N | 58.0100989 | 49.7160044 | 49.4435567 |
| 2 C | 57.5171988 | 48.5831286 | 50.0666994 |
| 3 C | 56.1665839 | 48.5027960 | 50.7071665 |
| 4 N | 59.5837216 | 48.1917406 | 49.2627765 |
| 5 C | 58.5165250 | 47.6419250 | 49.9439013 |
| 6 C | 59.2396271 | 49.4449914 | 48.9789773 |
| 7 H | 55.4042143 | 49.0216112 | 50.1064537 |
| 8 H | 55.8589308 | 47.4469030 | 50.7626586 |
| 9 H | 58.5250849 | 46.6168112 | 50.3060869 |
| 10 H | 59.8584979 | 50.1580502 | 48.4431438 |
| 11 H | 57.5549185 | 50.6384092 | 49.3783839 |
| 12 C | 61.5127694 | 43.8587955 | 52.7635544 |
| 13 C | 62.3097881 | 45.1625444 | 52.5578996 |
| 14 C | 62.3596537 | 45.5500332 | 51.0809830 |
| 15 O | 62.8820694 | 44.7382423 | 50.2832498 |
| 16 O | 61.8047119 | 46.6453868 | 50.7342732 |
| 17 H | 61.9758724 | 43.0640001 | 52.1596462 |
| 18 H | 61.5530949 | 43.5319300 | 53.8140693 |
| 19 H | 61.9123850 | 46.0000424 | 53.1499087 |
| 20 H | 63.3467336 | 44.9773606 | 52.8857114 |

| | | | |
|---|---|---|---|
| 21 N | 63.1973519 | 51.1406129 | 50.6407376 |
| 22 C | 62.5660940 | 50.6092149 | 51.7567288 |
| 23 C | 62.5969686 | 51.2646376 | 53.1018529 |
| 24 N | 62.2939790 | 49.2601637 | 49.9777166 |
| 25 C | 61.9999416 | 49.4411594 | 51.3132676 |
| 26 C | 63.0234538 | 50.3022359 | 49.5969730 |
| 27 H | 63.0104528 | 50.5877227 | 53.8660804 |
| 28 H | 63.2697331 | 52.1301598 | 53.0616923 |
| 29 H | 61.4133504 | 48.7136598 | 51.8649963 |
| 30 H | 63.4521707 | 50.4590103 | 48.6079789 |
| 31 H | 63.7343949 | 52.0048206 | 50.6034287 |
| 32 Fe | 61.5986759 | 47.5692744 | 48.8599088 |
| 33 O | 61.8289392 | 45.7675308 | 48.1398229 |
| 34 C | 62.3473350 | 48.3024939 | 46.5624529 |
| 35 O | 61.1174337 | 48.3391751 | 46.9003269 |
| 36 C | 62.7824132 | 48.3758129 | 45.1277310 |
| 37 C | 62.2627600 | 49.5716967 | 44.3239542 |
| 38 C | 63.1348924 | 50.8432967 | 44.4383699 |
| 39 O | 62.6056283 | 51.9254564 | 44.1082825 |
| 40 O | 64.3329905 | 50.6878324 | 44.7926807 |
| 41 H | 62.4286370 | 47.4248974 | 44.6930145 |
| 42 H | 63.8776852 | 48.3683219 | 45.0946550 |
| 43 H | 62.2796282 | 49.3070264 | 43.2553026 |
| 44 H | 61.2129903 | 49.8058225 | 44.5551807 |
| 45 O | 63.1785637 | 48.1019855 | 47.5073415 |
| 46 C | 60.9749670 | 41.8772867 | 44.2820260 |
| 47 N | 61.5244003 | 42.8335636 | 45.2925793 |
| 48 H | 61.7500271 | 41.1204757 | 44.0844691 |
| 49 H | 60.7576731 | 42.4349303 | 43.3572153 |
| 50 H | 61.6087499 | 42.3121239 | 46.1858048 |
| 51 C | 62.9702985 | 43.2701163 | 44.9482526 |
| 52 H | 62.8807303 | 43.8652024 | 44.0246194 |
| 53 C | 63.5819096 | 44.0397606 | 46.0575869 |
| 54 H | 63.5040806 | 42.3313724 | 44.7442020 |
| 55 C | 60.6365677 | 44.0244616 | 45.4949893 |
| 56 H | 59.5959034 | 43.6859791 | 45.6058705 |
| 57 H | 60.9544164 | 44.5791153 | 46.3817823 |
| 58 H | 60.7146827 | 44.6659081 | 44.6074596 |
| 59 H | 62.3036291 | 45.2855342 | 48.8789659 |
| 60 H | 62.7036528 | 45.5662775 | 47.1080182 |
| 61 H | 64.1446721 | 43.5149022 | 46.8435310 |
| 62 O | 63.2561215 | 45.3036878 | 46.2034943 |
| 63 H | 56.1093974 | 48.9508947 | 51.6991365 |
| 64 H | 60.4615104 | 43.9664701 | 52.4964816 |
| 65 H | 61.6309018 | 51.6001629 | 53.4789536 |
| 66 H | 60.0712736 | 41.3922481 | 44.6510016 |

MEL-OH-PC-Cbeta

| | | | |
|---|---|---|---|
| 1 N | 57.8906090 | 49.8001110 | 49.5023882 |
| 2 C | 57.3531635 | 48.6402921 | 50.0284114 |
| 3 C | 56.0248700 | 48.5630959 | 50.7131723 |
| 4 N | 59.4046950 | 48.2355925 | 49.1841706 |
| 5 C | 58.3099660 | 47.6729386 | 49.8093332 |
| 6 C | 59.1127848 | 49.5211008 | 49.0204258 |

| | | | |
|---|---|---|---|
| 7 H | 55.2424519 | 49.0967569 | 50.1522420 |
| 8 H | 55.7091579 | 47.5091623 | 50.7623424 |
| 9 H | 58.2720674 | 46.6189703 | 50.0769972 |
| 10 H | 59.7686487 | 50.2636376 | 48.5746619 |
| 11 H | 57.4663550 | 50.7381332 | 49.4924116 |
| 12 C | 61.4802877 | 43.7255161 | 52.6932774 |
| 13 C | 62.3685983 | 44.9457854 | 52.3989792 |
| 14 C | 62.3470678 | 45.3204008 | 50.9157365 |
| 15 O | 62.5917003 | 44.4160301 | 50.0662521 |
| 16 O | 62.0133571 | 46.5043564 | 50.6237692 |
| 17 H | 61.8680087 | 42.8690165 | 52.1208396 |
| 18 H | 61.5257127 | 43.4467004 | 53.7571609 |
| 19 H | 62.0845403 | 45.8263242 | 52.9935779 |
| 20 H | 63.4046073 | 44.6791302 | 52.6716297 |
| 21 N | 63.0332156 | 51.1927860 | 50.5760794 |
| 22 C | 62.5360893 | 50.6003149 | 51.7264182 |
| 23 C | 62.5956684 | 51.2464496 | 53.0750007 |
| 24 N | 62.2037946 | 49.2619406 | 49.9452894 |
| 25 C | 62.0195616 | 49.4005219 | 51.3035815 |
| 26 C | 62.8222954 | 50.3572663 | 49.5309084 |
| 27 H | 63.0049572 | 50.5567075 | 53.8294724 |
| 28 H | 63.2810218 | 52.1025735 | 53.0358910 |
| 29 H | 61.5359530 | 48.6246899 | 51.8901900 |
| 30 H | 63.1351997 | 50.5722593 | 48.5091691 |
| 31 H | 63.5118714 | 52.0891974 | 50.5185037 |
| 32 Fe | 61.4114903 | 47.5483395 | 48.9256999 |
| 33 O | 61.2763257 | 45.5027147 | 48.0765977 |
| 34 C | 62.4247138 | 47.6458564 | 46.1348525 |
| 35 O | 61.6725664 | 48.2445003 | 46.9787968 |
| 36 C | 62.7642643 | 48.2675822 | 44.8003144 |
| 37 C | 62.1114074 | 49.5842317 | 44.3917995 |
| 38 C | 63.0597526 | 50.8105485 | 44.4158680 |
| 39 O | 62.5529752 | 51.9178056 | 44.1392898 |
| 40 O | 64.2767661 | 50.6073248 | 44.6695346 |
| 41 H | 62.6016609 | 47.4665971 | 44.0647259 |
| 42 H | 63.8561394 | 48.4258293 | 44.8103561 |
| 43 H | 61.7431427 | 49.5190545 | 43.3561447 |
| 44 H | 61.2250698 | 49.8072235 | 45.0026216 |
| 45 O | 62.9086260 | 46.4988975 | 46.3691429 |
| 46 C | 61.0425980 | 41.7803801 | 44.2207241 |
| 47 N | 61.6858678 | 42.8375174 | 45.0674713 |
| 48 H | 61.6942285 | 40.8945558 | 44.2423751 |
| 49 H | 60.9640961 | 42.1646955 | 43.1902152 |
| 50 H | 61.4497924 | 42.6234285 | 46.0616608 |
| 51 C | 63.1702978 | 42.8386900 | 44.9368265 |
| 52 H | 63.4489205 | 43.2188284 | 43.9431046 |
| 53 C | 63.8427002 | 43.6641744 | 46.0277477 |
| 54 H | 63.5021799 | 41.7906104 | 45.0241046 |
| 55 C | 61.1327885 | 44.1986993 | 44.7748826 |
| 56 H | 60.0503012 | 44.1837705 | 44.9529043 |
| 57 H | 61.6051920 | 44.9323977 | 45.4360675 |
| 58 H | 61.3495529 | 44.4505400 | 43.7261282 |
| 59 H | 61.7959153 | 45.0081241 | 48.7736431 |
| 60 H | 61.9711833 | 45.7749973 | 47.3948985 |

61 H 63.2707897 43.7581029 46.9834918
62 O 64.9345509 44.1322731 45.8837777
63 H 56.0142480 48.9951934 51.7137952
64 H 60.4308696 43.8841497 52.4450352
65 H 61.6370706 51.5912380 53.4626584
66 H 60.0531794 41.5190505 44.5959905

MEL-OH-RC

1 N 57.6745108 49.7740361 49.5260611
2 C 57.1905986 48.6275792 50.1297017
3 C 55.8748219 48.5479801 50.8366016
4 N 59.2574200 48.2791528 49.3359686
5 C 58.1944321 47.6956610 49.9867906
6 C 58.9117333 49.5308676 49.0701868
7 H 55.0750364 49.0573971 50.2787774
8 H 55.5705398 47.4929648 50.9193350
9 H 58.2189747 46.6602052 50.3161031
10 H 59.5280525 50.2545524 48.5475792
11 H 57.2194153 50.6946935 49.4639621
12 C 61.5358095 43.9620988 52.6581465
13 C 62.3269289 45.2589528 52.4014853
14 C 62.2895954 45.6638726 50.9223076
15 O 62.8817961 44.9804754 50.0752461
16 O 61.5752969 46.6960447 50.6661694
17 H 61.9764835 43.1546246 52.0514657
18 H 61.6221222 43.6499076 53.7104403
19 H 61.9605642 46.0902493 53.0218378
20 H 63.3801814 45.0711000 52.6687804
21 N 62.9684985 51.0583121 50.4242529
22 C 62.4043371 50.5567726 51.5886233
23 C 62.5507900 51.2142713 52.9280303
24 N 61.9273171 49.2216939 49.8389825
25 C 61.7523953 49.4162423 51.1935278
26 C 62.6682496 50.2302193 49.3988180
27 H 63.0027638 50.5316210 53.6650808
28 H 63.2331755 52.0708339 52.8397574
29 H 61.1756728 48.7208810 51.7938636
30 H 63.0110043 50.3884903 48.3771882
31 H 63.5423638 51.8956957 50.3415343
32 Fe 61.1324897 47.5060733 48.9009018
33 O 60.5290024 46.1364855 48.2847421
34 C 62.0699164 48.2215427 46.6609806
35 O 60.9202334 48.6287935 46.9783115
36 C 62.6353440 48.3623012 45.2715677
37 C 62.1439488 49.5394757 44.4288552
38 C 63.0458081 50.7969796 44.4770747
39 O 62.5395044 51.8753439 44.1028182
40 O 64.2468552 50.6346959 44.8240102
41 H 62.4024361 47.3932158 44.7942439
42 H 63.7299122 48.4068605 45.3386323
43 H 62.1456745 49.2385483 43.3695356
44 H 61.1041860 49.8105206 44.6607749
45 O 62.7323213 47.5910528 47.5653836
46 C 61.0952637 42.0127360 44.3525245

| | | | |
|---|---|---|---|
| 47 N | 61.7754886 | 42.8625820 | 45.3890373 |
| 48 H | 61.7809976 | 41.1859610 | 44.1133566 |
| 49 H | 60.9383804 | 42.6293954 | 43.4545034 |
| 50 H | 61.8036844 | 42.3069442 | 46.2656809 |
| 51 C | 63.2186161 | 43.0731614 | 44.9982500 |
| 52 H | 63.2235440 | 43.5164216 | 43.9912503 |
| 53 C | 63.9953652 | 43.9748116 | 45.9438330 |
| 54 H | 63.6475472 | 42.0622977 | 44.9497942 |
| 55 C | 61.0399599 | 44.1425907 | 45.6615443 |
| 56 H | 60.0027141 | 43.8934814 | 45.9223752 |
| 57 H | 61.5115674 | 44.6787509 | 46.4902149 |
| 58 H | 61.0672816 | 44.7571796 | 44.7530225 |
| 59 H | 63.7074216 | 43.7560680 | 46.9943006 |
| 60 H | 64.3145642 | 45.9007191 | 46.1276241 |
| 61 H | 65.0666386 | 43.7158548 | 45.8645415 |
| 62 O | 63.7605177 | 45.3054963 | 45.5763812 |
| 63 H | 55.8935161 | 49.0062679 | 51.8253837 |
| 64 H | 60.4728703 | 44.0554421 | 52.4356283 |
| 65 H | 61.6209639 | 51.5693627 | 53.3723283 |
| 66 H | 60.1441908 | 41.6055038 | 44.6955836 |

MEL-OH-TS1-O

| | | | |
|---|---|---|---|
| 1 N | 57.9506043 | 49.7003658 | 49.4363344 |
| 2 C | 57.4629944 | 48.5666179 | 50.0623705 |
| 3 C | 56.1253222 | 48.4908839 | 50.7295644 |
| 4 N | 59.5163659 | 48.1749268 | 49.2309447 |
| 5 C | 58.4572834 | 47.6228466 | 49.9218691 |
| 6 C | 59.1740962 | 49.4299552 | 48.9558329 |
| 7 H | 55.3501380 | 49.0077195 | 50.1438088 |
| 8 H | 55.8162110 | 47.4360337 | 50.7948531 |
| 9 H | 58.4701361 | 46.5962361 | 50.2799071 |
| 10 H | 59.7904079 | 50.1363859 | 48.4091826 |
| 11 H | 57.4969517 | 50.6234807 | 49.3769315 |
| 12 C | 61.5624206 | 43.8805790 | 52.6726691 |
| 13 C | 62.3334558 | 45.1891607 | 52.4279433 |
| 14 C | 62.3802718 | 45.5500485 | 50.9351500 |
| 15 O | 62.9476701 | 44.8006493 | 50.1464539 |
| 16 O | 61.7611748 | 46.6483298 | 50.6268426 |
| 17 H | 62.0183832 | 43.0838402 | 52.0643686 |
| 18 H | 61.6385963 | 43.5629458 | 53.7240698 |
| 19 H | 61.9264688 | 46.0302866 | 53.0083906 |
| 20 H | 63.3768278 | 45.0314627 | 52.7491103 |
| 21 N | 63.1193285 | 51.1254009 | 50.5854760 |
| 22 C | 62.5208846 | 50.5843519 | 51.7143605 |
| 23 C | 62.5860540 | 51.2363580 | 53.0607915 |
| 24 N | 62.1921138 | 49.2546026 | 49.9305029 |
| 25 C | 61.9393354 | 49.4209027 | 51.2759014 |
| 26 C | 62.9113237 | 50.2980038 | 49.5388807 |
| 27 H | 63.0068472 | 50.5547905 | 53.8168404 |
| 28 H | 63.2652002 | 52.0972075 | 53.0099442 |
| 29 H | 61.3647435 | 48.6907123 | 51.8370512 |
| 30 H | 63.3118605 | 50.4694520 | 48.5412703 |
| 31 H | 63.6599551 | 51.9871002 | 50.5419622 |
| 32 Fe | 61.5204273 | 47.5188488 | 48.8701662 |

| | | | |
|---|---|---|---|
| 33 O | 61.3621926 | 45.9918697 | 47.9882373 |
| 34 C | 62.3798848 | 48.3933521 | 46.6463445 |
| 35 O | 61.1903358 | 48.6867578 | 46.9554080 |
| 36 C | 62.8626331 | 48.4031670 | 45.2218717 |
| 37 C | 62.3523551 | 49.5364837 | 44.3401617 |
| 38 C | 63.1780381 | 50.8397626 | 44.4557702 |
| 39 O | 62.6201820 | 51.8981886 | 44.0941625 |
| 40 O | 64.3682137 | 50.7316824 | 44.8456325 |
| 41 H | 62.5276270 | 47.4159199 | 44.8502688 |
| 42 H | 63.9575784 | 48.3825631 | 45.2159369 |
| 43 H | 62.4444644 | 49.2352627 | 43.2849666 |
| 44 H | 61.2860125 | 49.7466802 | 44.5072167 |
| 45 O | 63.1346206 | 47.9596154 | 47.5838152 |
| 46 C | 60.9912730 | 41.7781706 | 44.4711444 |
| 47 N | 61.4384879 | 42.8338084 | 45.4405811 |
| 48 H | 61.7829790 | 41.0154955 | 44.4039428 |
| 49 H | 60.8577439 | 42.2626010 | 43.4915963 |
| 50 H | 61.3986941 | 42.4145258 | 46.3886898 |
| 51 C | 62.8878417 | 43.1933659 | 45.2253704 |
| 52 H | 62.9521130 | 43.7071535 | 44.2542532 |
| 53 C | 63.4631338 | 44.0612425 | 46.3454077 |
| 54 H | 63.4263867 | 42.2361865 | 45.1686743 |
| 55 C | 60.5346896 | 44.0373965 | 45.3796317 |
| 56 H | 59.4864446 | 43.7019464 | 45.3790609 |
| 57 H | 60.7298423 | 44.6958445 | 46.2273639 |
| 58 H | 60.7525375 | 44.5699688 | 44.4440992 |
| 59 H | 63.3449038 | 43.5395832 | 47.3188201 |
| 60 H | 62.2182091 | 45.7140733 | 47.3121723 |
| 61 H | 64.5646863 | 44.0924943 | 46.1796607 |
| 62 O | 62.9727275 | 45.3424288 | 46.3768080 |
| 63 H | 56.0923004 | 48.9421516 | 51.7211965 |
| 64 H | 60.5021799 | 43.9739464 | 52.4376384 |
| 65 H | 61.6302680 | 51.5813238 | 53.4551755 |
| 66 H | 60.0527334 | 41.3213227 | 44.7849923 |

MEL-OH-IM1-O

| | | | |
|---|---|---|---|
| 1 N | 57.8927829 | 49.7219429 | 49.4504856 |
| 2 C | 57.4083194 | 48.5862407 | 50.0757218 |
| 3 C | 56.0757572 | 48.5077922 | 50.7516890 |
| 4 N | 59.4660746 | 48.2055013 | 49.2526530 |
| 5 C | 58.4069929 | 47.6477761 | 49.9387405 |
| 6 C | 59.1191226 | 49.4582911 | 48.9756088 |
| 7 H | 55.2957415 | 49.0251540 | 50.1731289 |
| 8 H | 55.7673068 | 47.4527261 | 50.8173590 |
| 9 H | 58.4246408 | 46.6207375 | 50.2953772 |
| 10 H | 59.7365378 | 50.1677912 | 48.4342978 |
| 11 H | 57.4379655 | 50.6439530 | 49.3884628 |
| 12 C | 61.5569091 | 43.8570798 | 52.6610637 |
| 13 C | 62.3444476 | 45.1537676 | 52.4113666 |
| 14 C | 62.3856147 | 45.5235332 | 50.9199012 |
| 15 O | 62.8813324 | 44.7299453 | 50.1164707 |
| 16 O | 61.8565475 | 46.6629686 | 50.6265070 |
| 17 H | 62.0033331 | 43.0553930 | 52.0521603 |
| 18 H | 61.6285949 | 43.5390044 | 53.7128478 |

| | | | |
|---|---|---|---|
| 19 H | 61.9549804 | 45.9999167 | 52.9967908 |
| 20 H | 63.3871087 | 44.9791468 | 52.7282020 |
| 21 N | 63.0948673 | 51.1913488 | 50.6124959 |
| 22 C | 62.5330104 | 50.6209819 | 51.7452284 |
| 23 C | 62.5931029 | 51.2567229 | 53.0987069 |
| 24 N | 62.1854834 | 49.3115646 | 49.9479781 |
| 25 C | 61.9664970 | 49.4527575 | 51.3010959 |
| 26 C | 62.8737229 | 50.3744092 | 49.5579167 |
| 27 H | 63.0016499 | 50.5626108 | 53.8498210 |
| 28 H | 63.2789190 | 52.1124136 | 53.0644536 |
| 29 H | 61.4242040 | 48.7009606 | 51.8659403 |
| 30 H | 63.2405795 | 50.5750382 | 48.5520185 |
| 31 H | 63.6165752 | 52.0642218 | 50.5702085 |
| 32 Fe | 61.4535300 | 47.5473140 | 48.8814428 |
| 33 O | 61.2082826 | 45.9139905 | 47.9633317 |
| 34 C | 62.3520325 | 48.3719257 | 46.6490174 |
| 35 O | 61.1872625 | 48.7339249 | 46.9842239 |
| 36 C | 62.8210589 | 48.4030016 | 45.2186497 |
| 37 C | 62.3136060 | 49.5601799 | 44.3606147 |
| 38 C | 63.1601349 | 50.8477849 | 44.4620300 |
| 39 O | 62.6183287 | 51.9166778 | 44.1084362 |
| 40 O | 64.3570378 | 50.7204833 | 44.8329220 |
| 41 H | 62.4801541 | 47.4317297 | 44.8136814 |
| 42 H | 63.9170771 | 48.3807901 | 45.2049877 |
| 43 H | 62.3652664 | 49.2648244 | 43.3006500 |
| 44 H | 61.2563596 | 49.7872037 | 44.5599172 |
| 45 O | 63.0874891 | 47.8590378 | 47.5624172 |
| 46 C | 60.9880538 | 41.7593076 | 44.4013277 |
| 47 N | 61.5346124 | 42.7830419 | 45.3542272 |
| 48 H | 61.7268075 | 40.9477734 | 44.3189753 |
| 49 H | 60.8584532 | 42.2471291 | 43.4225146 |
| 50 H | 61.4706435 | 42.3810620 | 46.3101551 |
| 51 C | 63.0035395 | 43.0040525 | 45.1033396 |
| 52 H | 63.1006047 | 43.4901812 | 44.1207522 |
| 53 C | 63.6491615 | 43.8462249 | 46.2019879 |
| 54 H | 63.4545510 | 42.0016615 | 45.0641631 |
| 55 C | 60.7366028 | 44.0612679 | 45.3020589 |
| 56 H | 59.6673813 | 43.8031352 | 45.2920789 |
| 57 H | 60.9469044 | 44.6859958 | 46.1782463 |
| 58 H | 61.0004893 | 44.5887328 | 44.3746656 |
| 59 H | 63.4998903 | 43.3946183 | 47.2076882 |
| 60 H | 62.1016639 | 45.7425980 | 47.6126709 |
| 61 H | 64.7604789 | 43.8267135 | 46.0807005 |
| 62 O | 63.3049310 | 45.1578715 | 46.2000129 |
| 63 H | 56.0547657 | 48.9580852 | 51.7440915 |
| 64 H | 60.4976863 | 43.9603899 | 52.4256039 |
| 65 H | 61.6340050 | 51.6007963 | 53.4857621 |
| 66 H | 60.0330358 | 41.3604642 | 44.7432804 |

MEL-OH-TS2-O

| | | | |
|---|---|---|---|
| 1 N | 58.0643689 | 49.8034423 | 49.4640997 |
| 2 C | 57.5201807 | 48.6384376 | 49.9737028 |
| 3 C | 56.1824992 | 48.5571543 | 50.6399444 |
| 4 N | 59.5615589 | 48.2348089 | 49.1118917 |

| | | | | | | | |
|---|---|---|---|---|---|---|---|
| 5 C | 58.4691910 | 47.6665295 | 49.7374465 | 32 Fe | 61.5281612 | 47.4763315 | 48.6098450 |
| 6 C | 59.2799676 | 49.5261064 | 48.9669672 | 33 O | 60.8059177 | 45.9315908 | 47.5736968 |
| 7 H | 55.4092226 | 49.0942537 | 50.0696968 | 34 C | 62.4501206 | 48.2786022 | 46.4106772 |
| 8 H | 55.8680902 | 47.5028110 | 50.6803299 | 35 O | 61.3093761 | 48.6954719 | 46.8007153 |
| 9 H | 58.4257987 | 46.6082104 | 49.9847650 | 36 C | 62.9030612 | 48.4096882 | 44.9921887 |
| 10 H | 59.9379981 | 50.2605665 | 48.5126334 | 37 C | 62.3810048 | 49.6214008 | 44.2292948 |
| 11 H | 57.6432555 | 50.7439808 | 49.4749139 | 38 C | 63.1925456 | 50.9130225 | 44.4595750 |
| 12 C | 61.5596224 | 43.7854235 | 52.4830462 | 39 O | 62.6556820 | 51.9897082 | 44.1238482 |
| 13 C | 62.3858670 | 45.0044243 | 52.0488543 | 40 O | 64.3606596 | 50.7811975 | 44.9154856 |
| 14 C | 62.3715152 | 45.2197019 | 50.5274368 | 41 H | 62.5741022 | 47.4579895 | 44.5379337 |
| 15 O | 62.6622238 | 44.2952257 | 49.7634012 | 42 H | 63.9985234 | 48.3821931 | 44.9678811 |
| 16 O | 62.0267594 | 46.4075025 | 50.1549840 | 43 H | 62.4478063 | 49.4142565 | 43.1498122 |
| 17 H | 61.9309793 | 42.8998856 | 51.9441310 | 44 H | 61.3167527 | 49.8121473 | 44.4325985 |
| 18 H | 61.6819996 | 43.5776115 | 53.5566600 | 45 O | 63.1326565 | 47.6384434 | 47.2826603 |
| 19 H | 62.0670580 | 45.9286459 | 52.5526193 | 46 C | 60.8133750 | 41.2178880 | 45.4865436 |
| 20 H | 63.4359189 | 44.8218921 | 52.3375190 | 47 N | 61.0317092 | 42.7039865 | 45.4407426 |
| 21 N | 63.1302409 | 51.0862668 | 50.4193045 | 48 H | 61.2668016 | 40.8772398 | 46.4317727 |
| 22 C | 62.5660600 | 50.5064106 | 51.5464046 | 49 H | 61.3566416 | 40.7569156 | 44.6506588 |
| 23 C | 62.6354306 | 51.1408027 | 52.9033592 | 50 H | 60.3869535 | 43.1053385 | 46.1465139 |
| 24 N | 62.2601017 | 49.1943599 | 49.7389944 | 51 C | 62.4032176 | 43.0672245 | 45.9727676 |
| 25 C | 62.0251365 | 49.3271346 | 51.0916424 | 52 H | 63.1336329 | 42.6503586 | 45.2604775 |
| 26 C | 62.9362611 | 50.2702092 | 49.3596853 | 53 C | 62.6441223 | 44.5762472 | 46.1431654 |
| 27 H | 63.0533154 | 50.4445572 | 53.6474559 | 54 H | 62.4875148 | 42.5422171 | 46.9360317 |
| 28 H | 63.3221903 | 51.9983094 | 52.8649068 | 55 C | 60.7259334 | 43.3005482 | 44.1019091 |
| 29 H | 61.4833073 | 48.5685536 | 51.6498524 | 56 H | 59.6412747 | 43.2410501 | 43.9390268 |
| 30 H | 63.3059365 | 50.4851039 | 48.3568100 | 57 H | 61.0838587 | 44.3360304 | 44.0635563 |
| 31 H | 63.6505736 | 51.9612180 | 50.3868074 | 58 H | 61.2479078 | 42.7274838 | 43.3242920 |

59 H 61.6892563 45.1831528 46.8446694
60 H 60.2048414 46.3053072 46.8853126
61 H 63.3752120 44.7548343 46.9690864
62 O 62.7780154 45.2860578 45.0733938
63 H 56.1510587 48.9833475 51.6426597
64 H 60.4941779 43.9174660 52.2947379
65 H 61.6910296 51.4951771 53.3163877
66 H 59.7585233 40.9435464 45.4961178

MEL-OH-PC-O

1 N 57.6672626 49.8588886 49.5780524
2 C 57.1102624 48.6978100 50.0746467
3 C 55.8098073 48.6317734 50.8092938
4 N 59.1360613 48.2751215 49.1867565
5 C 58.0405118 47.7167853 49.8091880
6 C 58.8780618 49.5708407 49.0726485
7 H 55.0061483 49.1676555 50.2827193
8 H 55.4878579 47.5805236 50.8722772
9 H 57.9921265 46.6574569 50.0519335
10 H 59.5490029 50.3170430 48.6557596
11 H 57.2742711 50.8087817 49.5989943
12 C 61.4333545 43.6845021 52.4531333
13 C 62.3232950 44.8708567 52.0530444
14 C 62.2320584 45.1838017 50.5475108
15 O 62.6447226 44.3561680 49.7179628
16 O 61.6660976 46.2963971 50.2781929
17 H 61.7766802 42.7957033 51.8982089
18 H 61.5363386 43.4450222 53.5228728
19 H 62.0748735 45.7776037 52.6241488
20 H 63.3651481 44.5975150 52.2900269
21 N 62.9386334 51.0342862 50.4020895
22 C 62.4565588 50.4628748 51.5697204
23 C 62.5796469 51.1243589 52.9105087
24 N 62.0384200 49.1224618 49.8007595
25 C 61.8988720 49.2721424 51.1639598
26 C 62.6743341 50.1988847 49.3669594
27 H 63.0023122 50.4357776 53.6589409
28 H 63.2803191 51.9692263 52.8388500
29 H 61.4071180 48.5124890 51.7664524
30 H 62.9626744 50.4006283 48.3354027
31 H 63.4469156 51.9129905 50.3280060
32 Fe 61.0596280 47.4471425 48.7873078
33 O 60.0065857 45.7195817 47.7659383
34 C 61.8451800 47.4795407 45.9096120
35 O 61.4209885 48.1565608 46.9235153
36 C 62.7318112 48.2135379 44.9357663
37 C 62.1021009 49.5005576 44.3678268
38 C 63.0381718 50.7331327 44.3223792
39 O 62.5169828 51.8093941 43.9581020
40 O 64.2452514 50.5692783 44.6357120
41 H 63.0535738 47.5212365 44.1482091
42 H 63.6307631 48.5230682 45.4926159
43 H 61.7448887 49.3480228 43.3391615
44 H 61.2133973 49.7922727 44.9457726

45 O 61.5277747 46.2746267 45.7402238

46 C 60.9430143 41.1604065 45.0594736

47 N 61.3005876 42.6071589 45.2367291

48 H 61.5328799 40.6249314 45.8188164

49 H 61.2534936 40.8501656 44.0535661

50 H 61.0314757 42.8356630 46.2177251

51 C 62.7844595 42.7949386 45.1454236

52 H 63.0797197 42.8671307 44.0896960

53 C 63.3149751 43.9909262 45.9262550

54 H 63.2421652 41.8989360 45.5951501

55 C 60.5700151 43.5450396 44.3184336

56 H 59.4950564 43.5013846 44.5542905

57 H 60.9475457 44.5643865 44.4817540

58 H 60.7533281 43.2377864 43.2764885

59 H 60.4799122 45.8831619 46.8849175

60 H 59.0444660 45.8233455 47.6184986

61 H 62.7865803 44.2449797 46.8756320

62 O 64.3156921 44.5553119 45.5679134

63 H 55.8623910 49.0649142 51.8081387

64 H 60.3744045 43.8539077 52.2582135

65 H 61.6509820 51.5019886 53.3383456

66 H 59.8857330 40.9437471 45.2120584

MEL-AL-RC

1 N 67.7412067 37.0601145 52.8126821

2 C 67.5902047 37.9753666 51.7900126

3 C 68.5393746 38.1179923 50.6463898

4 N 65.9394930 38.1970757 53.2943245

5 C 66.4512288 38.6781910 52.1109415

6 C 66.7472926 37.2238927 53.7013331

7 H 68.8454699 37.1319873 50.2683385

8 H 68.0384100 38.6283627 49.8115138

9 H 65.9729086 39.4925789 51.5802188

10 H 66.6251138 36.6547049 54.6169898

11 H 68.4845804 36.3559598 52.9074355

12 C 63.2884444 43.1638947 51.1807148

13 C 63.3602332 42.8459659 52.6827913

14 C 63.0199944 41.3890420 53.0476226

15 O 61.8984937 40.9401080 52.8260896

16 O 64.0061724 40.7548311 53.5951568

17 H 62.2707914 42.9688177 50.8078012

18 H 63.4974585 44.2286880 51.0065259

19 H 64.3374095 43.1150871 53.1080718

20 H 62.6177402 43.4839600 53.1926918

21 N 67.4815529 41.6512112 55.4199109

22 C 67.7960396 40.7287409 56.4010581

23 C 69.0223787 40.8147947 57.2474141

24 N 65.8958082 40.1368001 55.3482919

25 C 66.7875716 39.7918792 56.3432844

26 C 66.3509042 41.2607838 54.8102030

27 H 69.2166354 41.8513979 57.5661659

28 H 68.8873530 40.2263614 58.1663559

29 H 66.6638933 38.8908342 56.9378239

30 H 65.8881612 41.7871513 53.9848849

| | | | |
|---|---|---|---|
| 31 H | 68.0235752 | 42.4705177 | 55.1277842 |
| 32 Fe | 64.3643361 | 39.0166960 | 54.4174829 |
| 33 O | 63.2167464 | 38.1810882 | 53.6250478 |
| 34 C | 63.9022077 | 37.7423687 | 56.5258047 |
| 35 O | 64.8568778 | 37.4323981 | 55.7524987 |
| 36 C | 63.5344939 | 36.7946857 | 57.6299847 |
| 37 C | 62.6700703 | 35.6246128 | 57.1109252 |
| 38 C | 62.6079220 | 34.5467062 | 58.2124197 |
| 39 O | 63.2935581 | 33.5120836 | 58.0423674 |
| 40 O | 61.9192169 | 34.8408725 | 59.2227612 |
| 41 H | 63.0057175 | 37.3033532 | 58.4411087 |
| 42 H | 64.4746112 | 36.3864852 | 58.0297203 |
| 43 H | 61.6531835 | 35.9867412 | 56.8869487 |
| 44 H | 63.1055844 | 35.1971571 | 56.1978797 |
| 45 O | 63.2726190 | 38.8180553 | 56.2693071 |
| 46 C | 58.0955437 | 38.4176992 | 51.4002743 |
| 47 N | 58.7741517 | 37.4534610 | 52.3513823 |
| 48 H | 58.2392533 | 39.4424476 | 51.7666601 |
| 49 H | 57.0211334 | 38.2018847 | 51.3906648 |
| 50 H | 59.2046616 | 36.7067890 | 51.7781292 |
| 51 C | 59.9574614 | 38.0476985 | 53.0670152 |
| 52 H | 60.6844421 | 38.4151321 | 52.3177138 |
| 53 C | 59.6690349 | 39.1652644 | 54.0403776 |
| 54 H | 60.4657267 | 37.2347409 | 53.6122854 |
| 55 C | 57.8101064 | 36.7670671 | 53.2802017 |
| 56 H | 57.2892836 | 37.5329172 | 53.8652202 |
| 57 H | 57.1010462 | 36.1854755 | 52.6681290 |
| 58 H | 58.3871577 | 36.0942941 | 53.9348156 |
| 59 H | 60.5945561 | 39.5163219 | 54.5465176 |
| 60 O | 58.5797338 | 39.6412895 | 54.2584995 |
| 61 H | 69.4481746 | 38.6581433 | 50.9116944 |
| 62 H | 63.9989472 | 42.6092350 | 50.5678481 |
| 63 H | 69.9314265 | 40.4876559 | 56.7427564 |
| 64 H | 58.5005558 | 38.3382898 | 50.3914500 |

MEL-AL-TS1

| | | | |
|---|---|---|---|
| 1 N | 67.6333908 | 37.0699958 | 52.8170584 |
| 2 C | 67.5034678 | 38.0043304 | 51.8057615 |
| 3 C | 68.4685248 | 38.1472693 | 50.6746311 |
| 4 N | 65.7961401 | 38.1762403 | 53.2615333 |
| 5 C | 66.3480717 | 38.6896396 | 52.1051588 |
| 6 C | 66.6098714 | 37.2039813 | 53.6740747 |
| 7 H | 68.7683526 | 37.1593177 | 50.2953926 |
| 8 H | 67.9801425 | 38.6643816 | 49.8365965 |
| 9 H | 65.8841655 | 39.5162983 | 51.5793775 |
| 10 H | 66.4695985 | 36.6221418 | 54.5791883 |
| 11 H | 68.3866881 | 36.3772822 | 52.9247700 |
| 12 C | 63.3287475 | 43.2287254 | 51.1235850 |
| 13 C | 63.4261789 | 42.9160060 | 52.6257217 |
| 14 C | 63.0856995 | 41.4584689 | 52.9826447 |
| 15 O | 61.9593894 | 41.0254911 | 52.7345834 |
| 16 O | 64.0519086 | 40.8032675 | 53.5304064 |
| 17 H | 62.3046124 | 43.0323890 | 50.7696228 |
| 18 H | 63.5407183 | 44.2911200 | 50.9370097 |

| | | | |
|---|---|---|---|
| 19 H | 64.4121221 | 43.1760136 | 53.0361956 |
| 20 H | 62.6942822 | 43.5554416 | 53.1486649 |
| 21 N | 67.5279601 | 41.6849113 | 55.4383626 |
| 22 C | 67.8317270 | 40.7685601 | 56.4288175 |
| 23 C | 69.0656232 | 40.8324631 | 57.2657846 |
| 24 N | 65.9114331 | 40.1995569 | 55.3960146 |
| 25 C | 66.8031169 | 39.8532711 | 56.3891605 |
| 26 C | 66.3849711 | 41.3063091 | 54.8416315 |
| 27 H | 69.2772593 | 41.8630540 | 57.5931853 |
| 28 H | 68.9274614 | 40.2384048 | 58.1805350 |
| 29 H | 66.6681840 | 38.9655709 | 57.0021101 |
| 30 H | 65.9247903 | 41.8307769 | 54.0130941 |
| 31 H | 68.0809595 | 42.4901592 | 55.1302345 |
| 32 Fe | 64.2943532 | 39.0507239 | 54.4812897 |
| 33 O | 62.8123070 | 38.3358057 | 53.8109579 |
| 34 C | 63.9333086 | 37.7460037 | 56.6018288 |
| 35 O | 64.7779556 | 37.3966878 | 55.7187412 |
| 36 C | 63.5832524 | 36.7655573 | 57.6829296 |
| 37 C | 62.6799692 | 35.6311122 | 57.1516545 |
| 38 C | 62.6063371 | 34.5311720 | 58.2282457 |
| 39 O | 63.2855621 | 33.4952578 | 58.0389943 |
| 40 O | 61.9174120 | 34.8067003 | 59.2436016 |
| 41 H | 63.0937485 | 37.2567868 | 58.5289362 |
| 42 H | 64.5270763 | 36.3230467 | 58.0373461 |
| 43 H | 61.6700811 | 36.0259741 | 56.9523652 |
| 44 H | 63.0918763 | 35.2179943 | 56.2214824 |
| 45 O | 63.3932729 | 38.8874394 | 56.4722025 |
| 46 C | 58.1014454 | 38.4076112 | 51.4168905 |
| 47 N | 58.7074321 | 37.3537501 | 52.3130849 |
| 48 H | 58.3346251 | 39.3994550 | 51.8310696 |
| 49 H | 57.0123654 | 38.2760005 | 51.4107715 |
| 50 H | 58.8525591 | 36.5116893 | 51.7380651 |
| 51 C | 60.0962239 | 37.6858425 | 52.7762179 |
| 52 H | 60.7411117 | 37.8876127 | 51.8968324 |
| 53 C | 60.3254023 | 38.8285336 | 53.7248743 |
| 54 H | 60.5225297 | 36.7866013 | 53.2526336 |
| 55 C | 57.8020545 | 36.9464954 | 53.4420905 |
| 56 H | 57.5503537 | 37.8408884 | 54.0238299 |
| 57 H | 56.9049534 | 36.4861663 | 53.0046472 |
| 58 H | 58.3412458 | 36.2137565 | 54.0633202 |
| 59 H | 61.5467241 | 38.8082168 | 53.9520236 |
| 60 O | 59.5241495 | 39.5971732 | 54.1728201 |
| 61 H | 69.3842662 | 38.6753441 | 50.9403790 |
| 62 H | 64.0302349 | 42.6626086 | 50.5108091 |
| 63 H | 69.9662098 | 40.4965234 | 56.7518241 |
| 64 H | 58.4924595 | 38.3406895 | 50.4016583 |

MEL-AL-IM1

| | | | |
|---|---|---|---|
| 1 N | 67.7452965 | 37.0676992 | 52.8055446 |
| 2 C | 67.5931971 | 37.9888975 | 51.7862219 |
| 3 C | 68.5449371 | 38.1355726 | 50.6457871 |
| 4 N | 65.9256815 | 38.1851646 | 53.2804906 |
| 5 C | 66.4470198 | 38.6791272 | 52.1038859 |
| 6 C | 66.7445346 | 37.2167315 | 53.6858496 |

| | | | | | | | |
|---|---|---|---|---|---|---|---|
| 7 H | 68.8511556 | 37.1499780 | 50.2669912 | 34 C | 63.9231425 | 37.6949928 | 56.5168668 |
| 8 H | 68.0444543 | 38.6458172 | 49.8106316 | 35 O | 64.7992807 | 37.3511284 | 55.6619458 |
| 9 H | 65.9657781 | 39.4928347 | 51.5743360 | 36 C | 63.5637379 | 36.7311157 | 57.6066737 |
| 10 H | 66.6206720 | 36.6399068 | 54.5967546 | 37 C | 62.6573701 | 35.5863876 | 57.1047835 |
| 11 H | 68.4926106 | 36.3676905 | 52.9002187 | 38 C | 62.6017007 | 34.5100291 | 58.2059349 |
| 12 C | 63.2734662 | 43.1962170 | 51.1338728 | 39 O | 63.2961786 | 33.4802439 | 58.0368218 |
| 13 C | 63.3630641 | 42.9263311 | 52.6444967 | 40 O | 61.9147114 | 34.7988428 | 59.2193972 |
| 14 C | 63.1008594 | 41.4723987 | 53.0555714 | 41 H | 63.0793756 | 37.2385570 | 58.4464763 |
| 15 O | 61.9907914 | 40.9577124 | 52.8418335 | 42 H | 64.5083661 | 36.2929340 | 57.9648346 |
| 16 O | 64.0886846 | 40.8813687 | 53.6155770 | 43 H | 61.6448064 | 35.9755856 | 56.9089418 |
| 17 H | 62.2557517 | 42.9756126 | 50.7757477 | 44 H | 63.0609257 | 35.1552812 | 56.1786543 |
| 18 H | 63.4669427 | 44.2584217 | 50.9261848 | 45 O | 63.3771488 | 38.8321813 | 56.3602821 |
| 19 H | 64.3322493 | 43.2416104 | 53.0549983 | 46 C | 57.9540320 | 38.3234657 | 51.3585351 |
| 20 H | 62.5987959 | 43.5485114 | 53.1407854 | 47 N | 58.6545302 | 37.3786232 | 52.3067407 |
| 21 N | 67.5879767 | 41.7165544 | 55.4738620 | 48 H | 58.0368438 | 39.3470912 | 51.7482224 |
| 22 C | 67.8951913 | 40.7943064 | 56.4565265 | 49 H | 56.8942482 | 38.0485628 | 51.3141636 |
| 23 C | 69.1332168 | 40.8519198 | 57.2845627 | 50 H | 59.0990327 | 36.6312570 | 51.7422198 |
| 24 N | 65.9766201 | 40.2260189 | 55.4216141 | 51 C | 59.8430912 | 38.0186124 | 53.0031969 |
| 25 C | 66.8699216 | 39.8758366 | 56.4111749 | 52 H | 60.5375145 | 38.4005696 | 52.2341700 |
| 26 C | 66.4454253 | 41.3385715 | 54.8758434 | 53 C | 59.5395174 | 39.1194331 | 53.9693216 |
| 27 H | 69.3535246 | 41.8810805 | 57.6098682 | 54 H | 60.3960252 | 37.2314829 | 53.5420938 |
| 28 H | 68.9974263 | 40.2579506 | 58.1993453 | 55 C | 57.7175882 | 36.7007814 | 53.2654942 |
| 29 H | 66.7379060 | 38.9839059 | 57.0194417 | 56 H | 57.1846396 | 37.4698369 | 53.8372109 |
| 30 H | 65.9826379 | 41.8710445 | 54.0539182 | 57 H | 57.0115320 | 36.0923778 | 52.6764601 |
| 31 H | 68.1389652 | 42.5227667 | 55.1662185 | 58 H | 58.3141094 | 36.0571367 | 53.9323157 |
| 32 Fe | 64.3896391 | 39.0414491 | 54.4580460 | 59 H | 62.3092601 | 39.2223643 | 53.4378030 |
| 33 O | 62.8688060 | 38.4187555 | 53.5289161 | 60 O | 58.5070589 | 39.5989017 | 54.2935143 |

| | | | |
|---|---|---|---|
| 61 H | 69.4538581 | 38.6749998 | 50.9121477 |
| 62 H | 63.9854127 | 42.6310850 | 50.5323539 |
| 63 H | 70.0246860 | 40.5095654 | 56.7590693 |
| 64 H | 58.3863322 | 38.2830798 | 50.3587588 |

MEL-AL-TS2

| | | | |
|---|---|---|---|
| 1 N | 67.6328260 | 37.1150934 | 52.8304100 |
| 2 C | 67.5113040 | 38.0327021 | 51.8033510 |
| 3 C | 68.4794015 | 38.1584514 | 50.6725595 |
| 4 N | 65.7858369 | 38.2281315 | 53.2388232 |
| 5 C | 66.3533886 | 38.7227044 | 52.0830074 |
| 6 C | 66.5965941 | 37.2627939 | 53.6728148 |
| 7 H | 68.7784415 | 37.1659240 | 50.3046476 |
| 8 H | 67.9929033 | 38.6668562 | 49.8279893 |
| 9 H | 65.8942417 | 39.5359290 | 51.5332621 |
| 10 H | 66.4390599 | 36.6799000 | 54.5745489 |
| 11 H | 68.3817858 | 36.4191350 | 52.9503031 |
| 12 C | 63.3215436 | 43.2934760 | 51.0555647 |
| 13 C | 63.4070845 | 43.0099875 | 52.5638470 |
| 14 C | 63.1174501 | 41.5553337 | 52.9260873 |
| 15 O | 62.0418076 | 41.0382144 | 52.5695504 |
| 16 O | 64.0334180 | 40.9423154 | 53.5693487 |
| 17 H | 62.2984969 | 43.0972340 | 50.6989321 |
| 18 H | 63.5429921 | 44.3503187 | 50.8499275 |
| 19 H | 64.3793216 | 43.2972608 | 52.9872204 |
| 20 H | 62.6518167 | 43.6353878 | 53.0700660 |
| 21 N | 67.5420601 | 41.6793379 | 55.4121736 |
| 22 C | 67.8216981 | 40.7785834 | 56.4220983 |
| 23 C | 69.0531937 | 40.8448101 | 57.2621469 |
| 24 N | 65.8950295 | 40.2232660 | 55.3845003 |
| 25 C | 66.7735781 | 39.8832265 | 56.3930475 |
| 26 C | 66.3967523 | 41.3095505 | 54.8147409 |
| 27 H | 69.2643090 | 41.8773229 | 57.5843586 |
| 28 H | 68.9146176 | 40.2550069 | 58.1795788 |
| 29 H | 66.6143092 | 39.0203917 | 57.0365483 |
| 30 H | 65.9549923 | 41.8335044 | 53.9765679 |
| 31 H | 68.1056146 | 42.4749450 | 55.0989086 |
| 32 Fe | 64.2161932 | 39.0847257 | 54.4369356 |
| 33 O | 62.5788735 | 38.6120602 | 53.5375976 |
| 34 C | 63.7704636 | 37.6479045 | 56.4298507 |
| 35 O | 64.4412705 | 37.2341091 | 55.4319024 |
| 36 C | 63.4809852 | 36.6980618 | 57.5512192 |
| 37 C | 62.5736059 | 35.5312639 | 57.1130559 |
| 38 C | 62.5740496 | 34.4789352 | 58.2357282 |
| 39 O | 63.2919774 | 33.4649465 | 58.0703407 |
| 40 O | 61.9047818 | 34.7710124 | 59.2594205 |
| 41 H | 63.0411417 | 37.2165739 | 58.4086896 |
| 42 H | 64.4526824 | 36.2822626 | 57.8672667 |
| 43 H | 61.5491378 | 35.9037955 | 56.9496090 |
| 44 H | 62.9485288 | 35.0869731 | 56.1813039 |
| 45 O | 63.3738026 | 38.8561394 | 56.3991093 |
| 46 C | 58.1415503 | 38.3920760 | 51.4531684 |
| 47 N | 58.7336314 | 37.3067747 | 52.3202839 |
| 48 H | 58.4087315 | 39.3705730 | 51.8780756 |

| | | | |
|---|---|---|---|
| 49 H | 57.0486896 | 38.2928471 | 51.4637678 |
| 50 H | 58.9082273 | 36.4926703 | 51.7130413 |
| 51 C | 60.1255945 | 37.6114209 | 52.8438011 |
| 52 H | 60.7601167 | 37.8837003 | 51.9810299 |
| 53 C | 60.3167908 | 38.6796598 | 53.8850672 |
| 54 H | 60.5269729 | 36.6675158 | 53.2404690 |
| 55 C | 57.8001146 | 36.8394887 | 53.3991421 |
| 56 H | 57.4918345 | 37.7066968 | 53.9960193 |
| 57 H | 56.9353490 | 36.3637913 | 52.9126619 |
| 58 H | 58.3367709 | 36.1070290 | 54.0228454 |
| 59 H | 62.2890286 | 39.4708162 | 53.1311457 |
| 60 O | 59.6348686 | 39.6249447 | 54.1253534 |
| 61 H | 69.3940630 | 38.6897275 | 50.9356318 |
| 62 H | 64.0210297 | 42.7077330 | 50.4591660 |
| 63 H | 69.9550137 | 40.5079861 | 56.7509352 |
| 64 H | 58.5088759 | 38.3291660 | 50.4288729 |

MEL-AL-PC

| | | | |
|---|---|---|---|
| 1 N | 67.6239546 | 36.9984345 | 52.7728185 |
| 2 C | 67.5408168 | 37.9531367 | 51.7788689 |
| 3 C | 68.5174291 | 38.0962403 | 50.6574834 |
| 4 N | 65.7902280 | 38.1379394 | 53.1891592 |
| 5 C | 66.3915446 | 38.6555293 | 52.0623414 |
| 6 C | 66.5687087 | 37.1417922 | 53.5983495 |
| 7 H | 68.8207184 | 37.1078046 | 50.2825620 |
| 8 H | 68.0312573 | 38.6077210 | 49.8144988 |
| 9 H | 65.9578067 | 39.4978390 | 51.5356501 |
| 10 H | 66.3917672 | 36.5237629 | 54.4744030 |
| 11 H | 68.3645963 | 36.2943802 | 52.8900100 |
| 12 C | 63.4449889 | 43.2391859 | 51.1298445 |
| 13 C | 63.5882860 | 42.9804115 | 52.6408071 |
| 14 C | 63.1188181 | 41.6159683 | 53.1415387 |
| 15 O | 61.9973020 | 41.5776319 | 53.6857383 |
| 16 O | 63.8822655 | 40.5974776 | 52.9926473 |
| 17 H | 62.4019142 | 43.0768350 | 50.8135711 |
| 18 H | 63.6713384 | 44.2957254 | 50.9231570 |
| 19 H | 64.6239386 | 43.1419204 | 52.9764015 |
| 20 H | 62.9692299 | 43.7201294 | 53.1662208 |
| 21 N | 67.6218536 | 41.7388182 | 55.1706139 |
| 22 C | 67.8045765 | 40.9286160 | 56.2757285 |
| 23 C | 69.0115536 | 40.9644186 | 57.1556360 |
| 24 N | 65.8696361 | 40.4163447 | 55.2355100 |
| 25 C | 66.6874246 | 40.1218703 | 56.3056703 |
| 26 C | 66.4677538 | 41.3899443 | 54.5707204 |
| 27 H | 69.2512045 | 41.9931520 | 57.4718531 |
| 28 H | 68.8164494 | 40.3963781 | 58.0769819 |
| 29 H | 66.4304883 | 39.3503359 | 57.0289897 |
| 30 H | 66.1125930 | 41.8352245 | 53.6466836 |
| 31 H | 68.2336939 | 42.4809782 | 54.8208124 |
| 32 Fe | 64.3514186 | 39.1579356 | 54.3975892 |
| 33 O | 61.1156704 | 39.3151940 | 54.2902308 |
| 34 C | 63.4672200 | 37.5503664 | 56.1058022 |
| 35 O | 63.5971279 | 37.1529724 | 54.9058729 |
| 36 C | 63.3157523 | 36.5719643 | 57.2367891 |

| | | | |
|---|---|---|---|
| 37 C | 62.4163679 | 35.3625875 | 56.9681864 |
| 38 C | 62.5269227 | 34.4140762 | 58.1745549 |
| 39 O | 63.2806855 | 33.4186312 | 58.0557953 |
| 40 O | 61.9046908 | 34.7682675 | 59.2085653 |
| 41 H | 62.9971342 | 37.1025333 | 58.1422009 |
| 42 H | 64.3417472 | 36.2106524 | 57.4458618 |
| 43 H | 61.3712964 | 35.6943468 | 56.8567516 |
| 44 H | 62.7303185 | 34.8427278 | 56.0518227 |
| 45 O | 63.5807468 | 38.7970252 | 56.3400133 |
| 46 C | 58.1788332 | 38.4120180 | 51.2928196 |
| 47 N | 58.7675265 | 37.3823646 | 52.2343533 |
| 48 H | 58.3625535 | 39.4112185 | 51.7073235 |
| 49 H | 57.0945422 | 38.2555249 | 51.2438960 |
| 50 H | 59.0911405 | 36.5870450 | 51.6600532 |
| 51 C | 60.0340224 | 37.8385426 | 52.9023727 |
| 52 H | 60.7812346 | 38.0791327 | 52.1227565 |
| 53 C | 59.9236312 | 39.0694831 | 53.7885004 |
| 54 H | 60.4459076 | 36.9970110 | 53.4798968 |
| 55 C | 57.7610216 | 36.8354934 | 53.2053158 |
| 56 H | 57.3436727 | 37.6738960 | 53.7736708 |
| 57 H | 56.9834711 | 36.3077378 | 52.6302278 |
| 58 H | 58.2810728 | 36.1252663 | 53.8675965 |
| 59 H | 61.3761889 | 40.3036052 | 54.2459998 |
| 60 O | 58.9189625 | 39.7278575 | 53.9522590 |
| 61 H | 69.4315605 | 38.6277206 | 50.9219823 |
| 62 H | 64.1126124 | 42.6550194 | 50.4965236 |
| 63 H | 69.9214376 | 40.5845683 | 56.6909941 |
| 64 H | 58.6063407 | 38.3305552 | 50.2934857 |

MEL1-RCY239A

| | | | |
|---|---|---|---|
| N | 55.6945508 | 48.4980043 | 45.7753339 |
| C | 56.3974796 | 47.4668803 | 45.1827925 |
| C | 56.8656083 | 47.4687107 | 43.7688823 |
| N | 55.8934192 | 46.9463191 | 47.2996049 |
| C | 56.5223120 | 46.5067736 | 46.1593479 |
| C | 55.4063012 | 48.1505452 | 47.0405343 |
| H | 57.2615158 | 48.4522204 | 43.4819013 |
| H | 57.6827547 | 46.7423285 | 43.6659765 |
| H | 57.0118629 | 45.5384848 | 46.1005383 |
| H | 54.8768765 | 48.7785375 | 47.7497071 |
| H | 55.3871589 | 49.3775445 | 45.3403651 |
| C | 56.7858682 | 41.2477571 | 47.3653869 |
| C | 55.4990939 | 41.8096664 | 47.9914168 |
| C | 55.7847598 | 42.9655882 | 48.9598112 |
| O | 56.4008715 | 42.7595338 | 49.9970176 |
| O | 55.3264376 | 44.1188699 | 48.5675706 |
| H | 57.4763378 | 40.9810707 | 48.1806874 |
| H | 56.5770715 | 40.3195351 | 46.8102022 |
| H | 54.7666311 | 42.1228720 | 47.2324393 |
| H | 55.0488095 | 41.0005290 | 48.5896118 |
| N | 51.5475447 | 46.9086532 | 48.6398026 |
| C | 51.6355418 | 45.8765152 | 47.7193965 |
| C | 50.4888603 | 45.4850772 | 46.8388078 |

| | | | |
|---|---|---|---|
| N | 53.6043141 | 46.1684455 | 48.7725336 |
| C | 52.9311708 | 45.4300547 | 47.8203428 |
| C | 52.7439651 | 47.0554714 | 49.2493281 |
| H | 50.3780004 | 44.3913453 | 46.7888446 |
| H | 49.5487614 | 45.8766332 | 47.2562622 |
| H | 53.4291414 | 44.6370426 | 47.2720359 |
| H | 52.9417563 | 47.8005868 | 50.0151979 |
| H | 50.7161538 | 47.4526789 | 48.8558386 |
| Fe | 55.6856675 | 45.9479895 | 49.1071122 |
| O | 57.2930650 | 45.7652903 | 49.2524255 |
| C | 55.4741077 | 47.4586788 | 51.1511685 |
| O | 55.5485487 | 47.9048197 | 49.9726951 |
| C | 55.3656615 | 48.4278003 | 52.3064666 |
| C | 53.9895732 | 49.1262933 | 52.3471382 |
| C | 53.9780550 | 50.3177372 | 53.3406040 |
| O | 53.9235389 | 50.0410301 | 54.5703172 |
| O | 54.0313629 | 51.4557329 | 52.8312103 |
| H | 56.1385333 | 49.1993774 | 52.1507877 |
| H | 55.5516168 | 47.9139501 | 53.2604916 |
| H | 53.7501021 | 49.5205149 | 51.3487305 |
| H | 53.2140125 | 48.4007934 | 52.6411668 |
| O | 55.4576975 | 46.1916462 | 51.3032639 |
| C | 62.3742017 | 45.9685797 | 51.4041006 |
| N | 61.1097477 | 45.4026880 | 52.0053279 |
| H | 62.7268923 | 46.7573005 | 52.0840761 |
| H | 62.1371557 | 46.4405814 | 50.4363123 |
| H | 61.0884379 | 44.3856341 | 51.8120870 |
| C | 61.1473567 | 45.5427277 | 53.5127382 |
| H | 61.3104958 | 46.6174235 | 53.7021119 |
| C | 59.9108051 | 45.0279576 | 54.2220232 |
| H | 62.0474436 | 44.9949765 | 53.8411707 |
| C | 59.8768895 | 45.9974737 | 51.3984855 |
| H | 59.0115085 | 45.3550666 | 51.6248071 |
| H | 59.7185369 | 47.0001570 | 51.8280999 |
| H | 60.0312785 | 46.0649458 | 50.3151436 |
| H | 59.0082654 | 45.5861576 | 53.9426713 |
| H | 60.0651175 | 45.1691137 | 55.3017832 |
| H | 59.7253472 | 43.9618839 | 54.0210093 |
| H | 56.0933886 | 47.2252734 | 43.0391686 |
| H | 57.2744835 | 41.9305654 | 46.6703365 |
| H | 50.5655990 | 45.8303722 | 45.8078133 |
| H | 63.1363139 | 45.1992826 | 51.2798667 |

MEL1-TS1Y239A-meth

| | | | |
|---|---|---|---|
| N | 55.8896995 | 48.5288775 | 45.8333191 |
| C | 56.5215757 | 47.4702572 | 45.2057471 |
| C | 56.9071291 | 47.4486833 | 43.7671726 |
| N | 56.2404687 | 47.0243447 | 47.3836589 |
| C | 56.7345354 | 46.5390670 | 46.1943743 |
| C | 55.7377602 | 48.2264192 | 47.1334457 |
| H | 57.3029738 | 48.4248334 | 43.4560960 |
| H | 57.7101371 | 46.7119590 | 43.6305930 |
| H | 57.2048919 | 45.5626793 | 46.1163522 |

| | | | |
|---|---|---|---|
| H | 55.2812078 | 48.8732434 | 47.8752267 |
| H | 55.5319308 | 49.3904033 | 45.3988176 |
| C | 56.7877604 | 41.2749760 | 47.3588473 |
| C | 55.5750302 | 41.9095519 | 48.0602141 |
| C | 55.9813263 | 43.0472934 | 49.0052195 |
| O | 56.4707038 | 42.7922132 | 50.0997973 |
| O | 55.7892888 | 44.2443808 | 48.5357877 |
| H | 57.5077060 | 40.9609094 | 48.1311404 |
| H | 56.4936266 | 40.3640532 | 46.8139432 |
| H | 54.8263792 | 42.2675981 | 47.3368308 |
| H | 55.1124798 | 41.1283099 | 48.6838306 |
| N | 51.7603923 | 46.9219035 | 48.7161104 |
| C | 51.8625771 | 45.9010287 | 47.7856099 |
| C | 50.6924179 | 45.4795140 | 46.9453111 |
| N | 53.8381780 | 46.2188285 | 48.8387456 |
| C | 53.1720509 | 45.4810783 | 47.8793314 |
| C | 52.9587139 | 47.0809754 | 49.3224272 |
| H | 50.6064544 | 44.3827726 | 46.9035233 |
| H | 49.7585948 | 45.8466801 | 47.4014136 |
| H | 53.6833057 | 44.7061123 | 47.3153908 |
| H | 53.1396297 | 47.8240635 | 50.0941248 |
| H | 50.9191805 | 47.4458820 | 48.9431757 |
| Fe | 56.0861293 | 46.0524091 | 49.2263575 |
| O | 57.8789028 | 45.9111508 | 49.5010924 |
| C | 55.6736640 | 47.5558802 | 51.2441997 |
| O | 55.8014653 | 48.0231387 | 50.0793535 |
| C | 55.4448332 | 48.4957830 | 52.4023407 |
| C | 54.0391052 | 49.1318702 | 52.3878454 |
| C | 53.9680670 | 50.3277622 | 53.3709954 |
| O | 53.9374465 | 50.0583271 | 54.6036845 |
| O | 53.9652705 | 51.4613602 | 52.8512433 |
| H | 56.1765163 | 49.3141584 | 52.2909320 |
| H | 55.6085248 | 47.9783132 | 53.3585713 |
| H | 53.8158584 | 49.5125472 | 51.3810883 |
| H | 53.2816796 | 48.3808370 | 52.6631510 |
| O | 55.7202682 | 46.2830709 | 51.3804223 |
| C | 61.8570838 | 45.6080710 | 50.6914018 |
| N | 60.6906995 | 45.4826548 | 51.6397335 |
| H | 62.1649570 | 46.6637994 | 50.7148811 |
| H | 61.4815649 | 45.4234798 | 49.6719349 |
| H | 60.3443440 | 44.5031985 | 51.6061303 |
| C | 61.0969543 | 45.7278595 | 53.0963318 |
| H | 61.3682757 | 46.7952397 | 53.1798461 |
| C | 60.0360983 | 45.3165509 | 54.1002324 |
| H | 62.0083290 | 45.1278189 | 53.2448453 |
| C | 59.6234561 | 46.4332991 | 51.2262791 |
| H | 58.9625307 | 46.7016181 | 52.0591161 |
| H | 60.0832490 | 47.2958630 | 50.7228754 |
| H | 58.7307191 | 45.9951493 | 50.3322172 |
| H | 59.1233081 | 45.9207794 | 54.0216491 |
| H | 60.4444166 | 45.4693475 | 55.1111295 |
| H | 59.7614324 | 44.2565662 | 53.9911882 |
| H | 56.1061081 | 47.2125946 | 43.0666766 |
| H | 57.2742072 | 41.9340964 | 46.6398223 |

H 50.6979815 45.8187300 45.9094691
H 62.7190777 44.9693976 50.8840930

MEL1-TS1-ethY239A

N 55.8205241 48.5357694 45.7824775
C 56.4386991 47.4615621 45.1688860
C 56.8552125 47.4317552 43.7383619
N 56.1169366 47.0336910 47.3490330
C 56.6128973 46.5286065 46.1635518
C 55.6410909 48.2454610 47.0801966
H 57.2782578 48.3999937 43.4364332
H 57.6471000 46.6797789 43.6192782
H 57.0674992 45.5446478 46.0924529
H 55.1862857 48.9076272 47.8093029
H 55.4939296 49.4067342 45.3435687
C 56.8290491 41.3341045 47.2322122
C 55.6793611 42.0008247 47.9989871
C 56.1548697 43.1194283 48.9389695
O 57.0257240 42.8875129 49.7787767
O 55.5523934 44.2565473 48.7714536
H 57.5842011 41.0107093 47.9657228
H 56.4871242 40.4304590 46.7036439
H 54.8952145 42.3868447 47.3304506
H 55.2199954 41.2308513 48.6430916
N 51.5393707 46.9155439 48.6182001
C 51.6890586 45.8988421 47.6895242
C 50.5543230 45.4525745 46.8179660
N 53.6396167 46.2962498 48.7511405
C 53.0117682 45.5303320 47.7908882
C 52.7254215 47.1212282 49.2325813
H 50.4957380 44.3547966 46.7647347
H 49.6007202 45.7958424 47.2483165
H 53.5534293 44.7694926 47.2358301
H 52.8682699 47.8623074 50.0149596
H 50.6784874 47.4107474 48.8338540
Fe 55.8156254 46.1470093 49.2298049
O 57.5131155 46.0731080 49.7611581
C 55.3575436 47.7895065 51.1407668
O 55.6345490 48.2234574 49.9919944
C 55.2057977 48.7327962 52.3027557
C 53.7591107 49.2327577 52.4863601
C 53.7829647 50.4348350 53.4586879
O 53.7250156 50.1925464 54.6963571
O 53.9175821 51.5516106 52.9194813
H 55.8442016 49.6077290 52.1073254
H 55.5425510 48.2393984 53.2261309
H 53.3664916 49.5914462 51.5215900
H 53.1155802 48.4258604 52.8661747
O 55.1844046 46.5289477 51.2814486
C 62.4318460 45.9282881 51.2585538
N 61.0134696 45.4739591 51.4282037
H 62.6972315 46.4908864 52.1668269
H 62.4233171 46.6224542 50.4034294

| | | | |
|---|---|---|---|
| H | 60.9564774 | 44.8024408 | 52.2115558 |
| C | 60.1717351 | 46.6866429 | 51.7612225 |
| H | 60.3457947 | 47.3997731 | 50.9347084 |
| C | 58.7281488 | 46.3471027 | 51.9682117 |
| H | 60.6218010 | 47.1254748 | 52.6716292 |
| C | 60.5099062 | 44.7254025 | 50.2359629 |
| H | 59.4315327 | 44.5565610 | 50.3159962 |
| H | 60.7630510 | 45.2995664 | 49.3314517 |
| H | 61.0174234 | 43.7516930 | 50.2240362 |
| H | 58.1250438 | 46.1856983 | 50.8653040 |
| H | 58.1647386 | 47.1695354 | 52.4200592 |
| H | 58.5650203 | 45.4193261 | 52.5343754 |
| H | 56.0627928 | 47.2164815 | 43.0215668 |
| H | 57.2833721 | 41.9914494 | 46.4908921 |
| H | 50.5943779 | 45.8075483 | 45.7881814 |
| H | 63.1502449 | 45.1243623 | 51.0983244 |

MEL1-IM1Y239A-meth

| | | | |
|---|---|---|---|
| N | 55.6214264 | 48.4711625 | 45.7832271 |
| C | 56.3607841 | 47.4634916 | 45.1953651 |
| C | 56.8632257 | 47.4891387 | 43.7914197 |
| N | 55.7930606 | 46.8952487 | 47.2939143 |
| C | 56.4621432 | 46.4851660 | 46.1608279 |
| C | 55.2976030 | 48.0943140 | 47.0339310 |
| H | 57.2476408 | 48.4811198 | 43.5166711 |
| H | 57.6987842 | 46.7818474 | 43.6981299 |
| H | 56.9550030 | 45.5177738 | 46.0988708 |
| H | 54.7426891 | 48.7083510 | 47.7370199 |
| H | 55.3262501 | 49.3591261 | 45.3564482 |
| C | 56.7848499 | 41.2081706 | 47.3596210 |
| C | 55.5029328 | 41.7606299 | 48.0014047 |
| C | 55.7923064 | 42.8741679 | 49.0186229 |
| O | 56.5061002 | 42.6413562 | 49.9884831 |
| O | 55.2333066 | 44.0166951 | 48.7596795 |
| H | 57.4767860 | 40.9289345 | 48.1693050 |
| H | 56.5756901 | 40.2906602 | 46.7867281 |
| H | 54.7728522 | 42.1093164 | 47.2553761 |
| H | 55.0403067 | 40.9323843 | 48.5643363 |
| N | 51.4180543 | 46.9086105 | 48.5851343 |
| C | 51.5191324 | 45.8481094 | 47.6998607 |
| C | 50.4019512 | 45.4571074 | 46.7849103 |
| N | 53.4622586 | 46.1424272 | 48.8014827 |
| C | 52.8042562 | 45.3862714 | 47.8560299 |
| C | 52.6024496 | 47.0530460 | 49.2241616 |
| H | 50.3028857 | 44.3632009 | 46.7202169 |
| H | 49.4479686 | 45.8353027 | 47.1806740 |
| H | 53.3035229 | 44.5657828 | 47.3494133 |
| H | 52.7902289 | 47.8143481 | 49.9778668 |
| H | 50.5889203 | 47.4705041 | 48.7573079 |
| Fe | 55.6602291 | 45.8829460 | 49.1759988 |
| O | 57.5166126 | 45.6565665 | 49.1745213 |
| C | 55.4474853 | 47.4410076 | 51.1817693 |

| | | | |
|---|---|---|---|
| O | 55.4848344 | 47.8826362 | 50.0012147 |
| C | 55.3270188 | 48.4044180 | 52.3379684 |
| C | 53.9586972 | 49.1189929 | 52.3609704 |
| C | 53.9476859 | 50.3097986 | 53.3530063 |
| O | 53.8650612 | 50.0351452 | 54.5825245 |
| O | 54.0308443 | 51.4483089 | 52.8473762 |
| H | 56.1125970 | 49.1655543 | 52.1957756 |
| H | 55.4968566 | 47.8857451 | 53.2917061 |
| H | 53.7375409 | 49.5124635 | 51.3584719 |
| H | 53.1707216 | 48.4029667 | 52.6452419 |
| O | 55.4824065 | 46.1690585 | 51.3380137 |
| C | 62.1543151 | 45.8023842 | 51.0674662 |
| N | 60.9557183 | 45.3981148 | 51.8818602 |
| H | 62.4521092 | 46.8029087 | 51.4088282 |
| H | 61.8511171 | 45.8372834 | 50.0087319 |
| H | 60.8340394 | 44.3702077 | 51.7875866 |
| C | 61.1794566 | 45.6470725 | 53.3799971 |
| H | 61.3589340 | 46.7317009 | 53.4833743 |
| C | 60.0240040 | 45.1626250 | 54.2337625 |
| H | 62.1079950 | 45.1070986 | 53.6336051 |
| C | 59.7544114 | 46.0463741 | 51.3791631 |
| H | 58.8067223 | 45.5260257 | 51.5356930 |
| H | 59.8836862 | 47.0480643 | 50.9587644 |
| H | 58.0586920 | 46.3639090 | 48.7826497 |
| H | 59.0916236 | 45.6924277 | 54.0002253 |
| H | 60.2694167 | 45.3623734 | 55.2881480 |
| H | 59.8484885 | 44.0824857 | 54.1083984 |
| H | 56.1039249 | 47.2390228 | 43.0504935 |
| H | 57.2720098 | 41.9034264 | 46.6759825 |
| H | 50.5098110 | 45.8166808 | 45.7616115 |
| H | 62.9891754 | 45.1070536 | 51.1545903 |

MEL2-RCY239A

| | | | |
|---|---|---|---|
| N | 62.8372821 | 50.3525123 | 47.1562414 |
| C | 62.6622783 | 49.1810410 | 46.4447833 |
| C | 63.1792358 | 48.9736650 | 45.0632052 |
| N | 61.7007414 | 49.0135956 | 48.4584572 |
| C | 61.9580993 | 48.3473387 | 47.2830878 |
| C | 62.2467490 | 50.2149867 | 48.3555557 |
| H | 64.1653886 | 49.4457019 | 44.9535074 |
| H | 63.3270741 | 47.8995201 | 44.8880817 |
| H | 61.6257101 | 47.3282387 | 47.1040692 |
| H | 62.2346104 | 50.9615246 | 49.1397401 |
| H | 63.3063827 | 51.2065867 | 46.8244360 |
| C | 58.1372840 | 44.4526569 | 47.6156885 |
| C | 57.6431712 | 45.6928736 | 48.3758548 |
| C | 58.6441239 | 46.1540932 | 49.4450050 |
| O | 58.9944153 | 45.3646479 | 50.3353258 |
| O | 59.0657722 | 47.3566692 | 49.3003793 |
| H | 58.3140183 | 43.6468472 | 48.3454482 |
| H | 57.3694051 | 44.0839570 | 46.9187571 |
| H | 57.4153542 | 46.5287478 | 47.6986656 |
| H | 56.7081953 | 45.4168776 | 48.8929474 |
| N | 58.4873026 | 52.0699442 | 49.1967226 |

| | | | |
|---|---|---|---|
| C | 57.9499657 | 51.2851490 | 48.1880716 |
| C | 56.9953907 | 51.8337920 | 47.1700925 |
| N | 59.3159484 | 50.0631243 | 49.5043129 |
| C | 58.4844167 | 50.0354072 | 48.4010993 |
| C | 59.2972661 | 51.3067225 | 49.9624332 |
| H | 56.2024741 | 51.1048039 | 46.9457716 |
| H | 56.4917290 | 52.7209663 | 47.5844196 |
| H | 58.3327897 | 49.1218578 | 47.8352325 |
| H | 59.8415857 | 51.6847057 | 50.8248923 |
| H | 58.2607292 | 53.0437230 | 49.3844866 |
| Fe | 60.5622209 | 48.4074894 | 50.0720731 |
| O | 61.5653829 | 47.1651212 | 50.3470266 |
| C | 60.8499230 | 49.5549579 | 52.3073714 |
| O | 61.6042703 | 49.9018215 | 51.3456100 |
| C | 60.9096638 | 50.2280667 | 53.6540655 |
| C | 61.1052963 | 51.7454407 | 53.6013033 |
| C | 61.2799786 | 52.4052171 | 54.9995618 |
| O | 61.0601099 | 51.6935387 | 56.0068143 |
| O | 61.6218041 | 53.6087497 | 54.9731117 |
| H | 61.7539296 | 49.7850964 | 54.2109527 |
| H | 60.0103101 | 49.9561674 | 54.2193757 |
| H | 61.9793711 | 52.0171055 | 52.9891030 |
| H | 60.2357309 | 52.2374575 | 53.1286597 |
| O | 60.0196737 | 48.6114974 | 52.0832495 |
| C | 63.5087980 | 44.1929778 | 52.1525474 |
| N | 63.4283207 | 44.9372219 | 53.4557571 |
| H | 64.3260612 | 44.6338008 | 51.5661892 |

| | | | |
|---|---|---|---|
| H | 62.5659492 | 44.3821815 | 51.6168467 |
| H | 64.3174907 | 44.8134982 | 53.9733629 |
| C | 63.2825646 | 46.4273915 | 53.1769566 |
| H | 62.2721419 | 46.5381700 | 52.7519463 |
| C | 63.4830647 | 47.2938305 | 54.4043826 |
| H | 64.0026207 | 46.6713027 | 52.3790918 |
| C | 62.3135427 | 44.4637236 | 54.3462585 |
| H | 62.3952768 | 43.3760928 | 54.5021162 |
| H | 62.4015721 | 44.9737480 | 55.3129210 |
| H | 61.3657722 | 44.7450195 | 53.8530979 |
| H | 62.7013144 | 47.1394171 | 55.1575289 |
| H | 63.4403871 | 48.3479291 | 54.0978896 |
| H | 64.4604417 | 47.1538291 | 54.8874144 |
| H | 62.5468310 | 49.3823441 | 44.2750981 |
| H | 59.0449584 | 44.6287444 | 47.0384644 |
| H | 57.4203698 | 52.1205944 | 46.2082171 |
| H | 63.6775315 | 43.1194355 | 52.2368275 |

MEL2-TS1Y239A-meth

| | | | |
|---|---|---|---|
| N | 63.0047472 | 50.2862008 | 47.1714043 |
| C | 62.7283622 | 49.1528940 | 46.4290702 |
| C | 63.1923978 | 48.9588256 | 45.0270546 |
| N | 61.8900260 | 48.9501014 | 48.4970060 |
| C | 62.0358637 | 48.3229530 | 47.2793582 |
| C | 62.4886923 | 50.1291309 | 48.4012935 |
| H | 64.1866157 | 49.4091864 | 44.9029287 |
| H | 63.3131848 | 47.8846198 | 44.8327122 |
| H | 61.6428837 | 47.3287905 | 47.0839825 |

| | | | |
|---|---|---|---|
| H | 62.5540710 | 50.8503788 | 49.2077933 |
| H | 63.4789569 | 51.1363965 | 46.8360117 |
| C | 58.1550030 | 44.5016849 | 47.5489841 |
| C | 57.6986793 | 45.7677427 | 48.2939784 |
| C | 58.6964976 | 46.2011598 | 49.3726850 |
| O | 58.9613846 | 45.4191348 | 50.3053386 |
| O | 59.2167175 | 47.3605734 | 49.2190446 |
| H | 58.3186738 | 43.6994682 | 48.2857314 |
| H | 57.3707363 | 44.1448480 | 46.8645592 |
| H | 57.5093371 | 46.6059260 | 47.6077679 |
| H | 56.7501782 | 45.5300127 | 48.8047415 |
| N | 58.4744975 | 52.1260966 | 49.0845004 |
| C | 57.9610864 | 51.3267145 | 48.0762049 |
| C | 56.9917920 | 51.8566392 | 47.0630757 |
| N | 59.3873922 | 50.1495722 | 49.3725353 |
| C | 58.5491958 | 50.0978765 | 48.2764778 |
| C | 59.3204827 | 51.3880485 | 49.8365688 |
| H | 56.2116379 | 51.1148473 | 46.8358153 |
| H | 56.4731204 | 52.7335148 | 47.4796410 |
| H | 58.4288556 | 49.1846886 | 47.7014517 |
| H | 59.8508895 | 51.7850531 | 50.6987712 |
| H | 58.2174788 | 53.0922057 | 49.2722439 |
| Fe | 60.6648864 | 48.4268663 | 50.0946552 |
| O | 61.7660673 | 47.0947429 | 50.6011179 |
| C | 60.7616771 | 49.7329357 | 52.2676520 |
| O | 61.6204782 | 49.9911866 | 51.3698627 |
| C | 60.7251009 | 50.4197475 | 53.6017447 |
| C | 61.0867487 | 51.9028903 | 53.6053892 |
| C | 61.2627835 | 52.4667337 | 55.0421155 |
| O | 61.0122667 | 51.6980842 | 55.9994826 |
| O | 61.6507648 | 53.6541711 | 55.1057791 |
| H | 61.4369435 | 49.8956694 | 54.2616405 |
| H | 59.7401105 | 50.2356060 | 54.0495896 |
| H | 62.0193674 | 52.0943760 | 53.0504802 |
| H | 60.3077604 | 52.5082394 | 53.1098312 |
| O | 59.8977882 | 48.8291429 | 51.9937413 |
| C | 64.3538723 | 44.5201779 | 52.3557417 |
| N | 63.2976667 | 45.3320101 | 53.0529449 |
| H | 65.2774162 | 44.6540281 | 52.9352369 |
| H | 64.4867621 | 44.9186406 | 51.3411743 |
| H | 63.1624263 | 44.8878163 | 53.9731200 |
| C | 63.7835574 | 46.7406489 | 53.3718732 |
| H | 63.8832789 | 47.2498004 | 52.3998190 |
| C | 62.8626793 | 47.4462231 | 54.3399672 |
| H | 64.7744223 | 46.6050320 | 53.8237537 |
| C | 61.9886419 | 45.3218380 | 52.3696990 |
| H | 61.8049879 | 44.3641084 | 51.8702476 |
| H | 61.1603040 | 45.6622127 | 53.0069700 |
| H | 61.8907930 | 46.2438798 | 51.3788020 |
| H | 61.8658560 | 47.6286559 | 53.9173570 |
| H | 63.3078023 | 48.4149960 | 54.6036604 |
| H | 62.7623199 | 46.8735101 | 55.2766466 |
| H | 62.5570376 | 49.3924105 | 44.2547926 |
| H | 59.0587355 | 44.6551381 | 46.9592495 |

H 57.4190319 52.1530676 46.1051281
H 64.1147581 43.4567936 52.3454802

MEL2-TS1Y239A-eth-Cα

N 63.0307535 50.2517854 47.2589978
C 62.7651611 49.1154280 46.5169654
C 63.2112800 48.9288583 45.1070371
N 61.9856743 48.8764084 48.6074456
C 62.1142938 48.2641197 47.3798651
C 62.5505456 50.0714079 48.5017995
H 64.2005357 49.3870336 44.9689803
H 63.3398561 47.8555159 44.9117571
H 61.7371722 47.2634866 47.1849207
H 62.6186623 50.7930271 49.3086279
H 63.4745673 51.1152003 46.9158707
C 58.1556562 44.5298042 47.6455539
C 57.7598103 45.7914387 48.4332792
C 58.7975603 46.1406825 49.5068471
O 58.9928689 45.3500721 50.4424703
O 59.4376673 47.2377839 49.3232620
H 58.3010637 43.7045077 48.3605754
H 57.3495021 44.2169307 46.9651407
H 57.6007992 46.6555693 47.7721108
H 56.8093455 45.5790010 48.9510249
N 58.5917449 52.0274147 49.2317111
C 58.0699378 51.2277003 48.2278552
C 57.0768573 51.7558025 47.2329471
N 59.4886099 50.0433932 49.5340331
C 58.6501576 49.9945799 48.4369427
C 59.4308736 51.2851456 49.9891817
H 56.2988902 51.0069062 47.0212514
H 56.5577331 52.6266816 47.6651636
H 58.5269058 49.0798724 47.8646706
H 59.9671233 51.6826150 50.8480549
H 58.3359834 52.9929962 49.4226859
Fe 60.8155062 48.3070001 50.2639426
O 61.9531609 47.0563853 50.8404616
C 60.8537881 49.5932772 52.4314710
O 61.6876028 49.8846531 51.5118064
C 60.8308388 50.3121969 53.7481388
C 61.1105368 51.8121603 53.6853150
C 61.2831066 52.4550461 55.0886494
O 61.0620629 51.7307323 56.0869517
O 61.6334000 53.6556506 55.0805185
H 61.5971265 49.8458034 54.3923074
H 59.8735108 50.0937478 54.2356729
H 62.0182427 52.0298418 53.1004254
H 60.2877317 52.3508850 53.1829869
O 60.0314730 48.6488348 52.1924881
C 63.5486670 44.1438042 51.9420665
N 63.4695601 45.0055577 53.1716083
H 64.3775839 44.5146245 51.3239099
H 62.6118950 44.3003628 51.3869628

| | | | |
|---|---|---|---|
| H | 64.3370106 | 44.8788516 | 53.7292295 |
| C | 63.4516051 | 46.4572205 | 52.7683872 |
| H | 62.5140272 | 46.6779237 | 51.8485400 |
| C | 63.2542058 | 47.4354884 | 53.8880119 |
| H | 64.3358659 | 46.6454219 | 52.1412288 |
| C | 62.3305949 | 44.6441420 | 54.0903461 |
| H | 62.3455582 | 43.5577898 | 54.2602601 |
| H | 62.4820407 | 45.1609679 | 55.0449418 |
| H | 61.3859933 | 44.9776922 | 53.6230260 |
| H | 62.2443794 | 47.3844048 | 54.3171559 |
| H | 63.4045041 | 48.4497497 | 53.4985352 |
| H | 63.9884226 | 47.2976853 | 54.7032521 |
| H | 62.5663802 | 49.3545078 | 44.3382758 |
| H | 59.0563044 | 44.6649625 | 47.0466947 |
| H | 57.4646711 | 52.0674792 | 46.2631403 |
| H | 63.7036530 | 43.0813468 | 52.1297650 |

MEL2-TS1Y239A-ethCβ

| | | | |
|---|---|---|---|
| N | 62.8346001 | 50.3798546 | 47.1580076 |
| C | 62.5929203 | 49.2188300 | 46.4511008 |
| C | 63.1267471 | 48.9731326 | 45.0811160 |
| N | 61.5820637 | 49.1346091 | 48.4462051 |
| C | 61.8132862 | 48.4432655 | 47.2801720 |
| C | 62.2139534 | 50.2925260 | 48.3463722 |
| H | 64.1278240 | 49.4151477 | 44.9814839 |
| H | 63.2460367 | 47.8925721 | 44.9224410 |
| H | 61.4073129 | 47.4516647 | 47.1000105 |
| H | 62.2491808 | 51.0372580 | 49.1313610 |
| H | 63.3531000 | 51.2045173 | 46.8271367 |
| C | 58.1069865 | 44.5464158 | 47.5828710 |
| C | 57.6405104 | 45.8136448 | 48.3177462 |
| C | 58.6276740 | 46.2441931 | 49.4142916 |
| O | 58.9173962 | 45.4165738 | 50.3014811 |
| O | 59.0997575 | 47.4216886 | 49.3079875 |
| H | 58.2634612 | 43.7522989 | 48.3293371 |
| H | 57.3350689 | 44.1803874 | 46.8888164 |
| H | 57.4601113 | 46.6509051 | 47.6277113 |
| H | 56.6827980 | 45.5782399 | 48.8130089 |
| N | 58.3869607 | 52.1623121 | 49.1438052 |
| C | 57.8753314 | 51.3468639 | 48.1452210 |
| C | 56.9362993 | 51.8622068 | 47.0972280 |
| N | 59.2374394 | 50.1770823 | 49.5053080 |
| C | 58.4232872 | 50.1100653 | 48.3912999 |
| C | 59.1968704 | 51.4301816 | 49.9372081 |
| H | 56.1643948 | 51.1144541 | 46.8623703 |
| H | 56.4047542 | 52.7425648 | 47.4892309 |
| H | 58.2975833 | 49.1807117 | 47.8455063 |
| H | 59.7319514 | 51.8310689 | 50.7948350 |
| H | 58.1531694 | 53.1403582 | 49.2983319 |
| Fe | 60.5032310 | 48.6475035 | 50.1257534 |
| O | 61.7441933 | 47.4719388 | 50.4813113 |
| C | 60.8139078 | 49.7641482 | 52.3397515 |
| O | 61.5257614 | 50.1930758 | 51.3808597 |
| C | 60.9167188 | 50.3423515 | 53.7234405 |

| | | | |
|---|---|---|---|
| C | 61.0746376 | 51.8632449 | 53.7524035 |
| C | 61.2930311 | 52.4320822 | 55.1791589 |
| O | 61.0567620 | 51.6702433 | 56.1449645 |
| O | 61.6815379 | 53.6206893 | 55.2254206 |
| H | 61.8016143 | 49.8826618 | 54.2015373 |
| H | 60.0524936 | 50.0145482 | 54.3131445 |
| H | 61.9126744 | 52.1913209 | 53.1182831 |
| H | 60.1717544 | 52.3556465 | 53.3460457 |
| O | 60.0055062 | 48.8041907 | 52.0908032 |
| C | 63.3796765 | 43.2501757 | 51.9785814 |
| N | 63.3836736 | 44.4169703 | 52.9243312 |
| H | 63.8676522 | 43.5614711 | 51.0449189 |
| H | 62.3229605 | 43.0154183 | 51.7773596 |
| H | 64.3113011 | 44.5299903 | 53.3661103 |
| C | 63.0835850 | 45.6477800 | 52.0606637 |
| H | 62.2145614 | 45.3330171 | 51.4628372 |
| C | 62.7760362 | 46.9551112 | 52.7169565 |
| H | 63.9417174 | 45.7485941 | 51.3811471 |
| C | 62.3930826 | 44.2558299 | 54.0437122 |
| H | 62.4785580 | 43.2406872 | 54.4627782 |
| H | 62.6283837 | 44.9972668 | 54.8170443 |
| H | 61.3842694 | 44.4438190 | 53.6338226 |
| H | 61.9830264 | 46.9795447 | 53.4696171 |
| H | 62.1399579 | 47.4512735 | 51.5711891 |
| H | 63.6068409 | 47.6383292 | 52.9256503 |
| H | 62.5203810 | 49.3913605 | 44.2777029 |
| H | 59.0195559 | 44.6966662 | 47.0060616 |
| H | 57.3832499 | 52.1466888 | 46.1446671 |
| H | 63.8830574 | 42.3507285 | 52.3330805 |

MEL2-IM1-methY239A

| | | | |
|---|---|---|---|
| N | 62.9226473 | 50.3700523 | 47.1252912 |
| C | 62.6901043 | 49.2099423 | 46.4114556 |
| C | 63.1840141 | 48.9916026 | 45.0234083 |
| N | 61.7655444 | 49.0658777 | 48.4482084 |
| C | 61.9739706 | 48.4000506 | 47.2617796 |
| C | 62.3534161 | 50.2460730 | 48.3356058 |
| H | 64.1767215 | 49.4467809 | 44.9043969 |
| H | 63.3126865 | 47.9151435 | 44.8473249 |
| H | 61.6074126 | 47.3921672 | 47.0855420 |
| H | 62.3838174 | 50.9900025 | 49.1217783 |
| H | 63.4088597 | 51.2114015 | 46.7871285 |
| C | 58.1728611 | 44.4557298 | 47.5635859 |
| C | 57.6759206 | 45.7064855 | 48.3059591 |
| C | 58.6675034 | 46.1882732 | 49.3663041 |
| O | 59.0837599 | 45.3678858 | 50.2222250 |
| O | 59.0332647 | 47.4029985 | 49.2944969 |
| H | 58.3498694 | 43.6577186 | 48.3014973 |
| H | 57.4044926 | 44.0793877 | 46.8717660 |
| H | 57.4465986 | 46.5361162 | 47.6218602 |
| H | 56.7401648 | 45.4367194 | 48.8258315 |
| N | 58.4022387 | 52.1879379 | 49.0650654 |
| C | 57.8777215 | 51.3803033 | 48.0686767 |
| C | 56.9343305 | 51.9019592 | 47.0302749 |
| N | 59.2606142 | 50.1962813 | 49.3985358 |

| | | | | | | | |
|---|---|---|---|---|---|---|---|
| C | 58.4308783 | 50.1422915 | 48.2981402 | H | 63.8696358 | 46.9019943 | 52.6809719 |
| C | 59.2238321 | 51.4441124 | 49.8391683 | C | 62.7386272 | 47.1232283 | 54.5579816 |
| H | 56.1589141 | 51.1578107 | 46.7959853 | H | 64.7000725 | 46.3115545 | 54.1765098 |
| H | 56.4086864 | 52.7827736 | 47.4267857 | C | 62.0440159 | 44.8927367 | 52.6546366 |
| H | 58.2918178 | 49.2208826 | 47.7407245 | H | 62.0682880 | 44.4739579 | 51.6481882 |
| H | 59.7572007 | 51.8394632 | 50.7017087 | H | 61.1739339 | 45.4145437 | 53.0653647 |
| H | 58.1686962 | 53.1641786 | 49.2301033 | H | 61.1110668 | 46.1954990 | 50.4712689 |
| Fe | 60.5581718 | 48.5165504 | 50.0812251 | H | 61.7921659 | 47.3443641 | 54.0474938 |
| O | 61.6238910 | 47.0162763 | 50.4386301 | H | 63.1811104 | 48.0739126 | 54.8876962 |
| C | 60.7683408 | 49.6740721 | 52.3068098 | H | 62.5302842 | 46.5332430 | 55.4666351 |
| O | 61.5771589 | 49.9368689 | 51.3544046 | H | 62.5525028 | 49.4112140 | 44.2403458 |
| C | 60.8782348 | 50.3191420 | 53.6555123 | H | 59.0788110 | 44.6284227 | 46.9826393 |
| C | 61.0592672 | 51.8399745 | 53.6327571 | H | 57.3873424 | 52.1820510 | 46.0792776 |
| C | 61.2449587 | 52.4436271 | 55.0551429 | H | 64.2224424 | 43.1851603 | 52.5119340 |
| O | 60.9749924 | 51.7070811 | 56.0314985 | MEL2-IM1Y239A-ethCβ | | | |
| O | 61.6457720 | 53.6281453 | 55.0781816 | N | 62.8396622 | 50.3422389 | 47.1297426 |
| H | 61.7535339 | 49.8766056 | 54.1612652 | C | 62.6353017 | 49.1722748 | 46.4248317 |
| H | 60.0043497 | 50.0348385 | 54.2544390 | C | 63.1561089 | 48.9504014 | 45.0468773 |
| H | 61.9231511 | 52.1379456 | 53.0182953 | N | 61.6430857 | 49.0514375 | 48.4273052 |
| H | 60.1757054 | 52.3326138 | 53.1880277 | C | 61.8945599 | 48.3677189 | 47.2611319 |
| O | 59.8513157 | 48.8233770 | 52.0538881 | C | 62.2280899 | 50.2343074 | 48.3212668 |
| C | 64.4313820 | 44.2445187 | 52.6608342 | H | 64.1512898 | 49.4039568 | 44.9418151 |
| N | 63.3128691 | 44.9760045 | 53.3515193 | H | 63.2847850 | 47.8736570 | 44.8731842 |
| H | 65.3089514 | 44.3495447 | 53.3159259 | H | 61.5333881 | 47.3575031 | 47.0892498 |
| H | 64.6237346 | 44.7520392 | 51.7056579 | H | 62.2312560 | 50.9860774 | 49.1006834 |
| H | 63.1911329 | 44.5341067 | 54.2754118 | H | 63.3372137 | 51.1797688 | 46.7992032 |
| C | 63.7334564 | 46.4219183 | 53.6644602 | C | 58.1429152 | 44.4455062 | 47.6019103 |

| | | | |
|---|---|---|---|
| C | 57.6326900 | 45.6805730 | 48.3577531 |
| C | 58.6245447 | 46.1785145 | 49.4205323 |
| O | 59.0273601 | 45.3713701 | 50.2893717 |
| O | 58.9750076 | 47.3945782 | 49.3100448 |
| H | 58.3314900 | 43.6444864 | 48.3340786 |
| H | 57.3794825 | 44.0634511 | 46.9070680 |
| H | 57.3872660 | 46.5088319 | 47.6770993 |
| H | 56.7024442 | 45.3910890 | 48.8769115 |
| N | 58.4198292 | 52.1380500 | 49.1281523 |
| C | 57.8899350 | 51.3385517 | 48.1279741 |
| C | 56.9440880 | 51.8701573 | 47.0951112 |
| N | 59.2693752 | 50.1454116 | 49.4495493 |
| C | 58.4367133 | 50.0963544 | 48.3488269 |
| C | 59.2409111 | 51.3916974 | 49.8991260 |
| H | 56.1613728 | 51.1326410 | 46.8638025 |
| H | 56.4274519 | 52.7548728 | 47.4965030 |
| H | 58.2934665 | 49.1749416 | 47.7937129 |
| H | 59.7892193 | 51.7786686 | 50.7549782 |
| H | 58.1902962 | 53.1138232 | 49.3006929 |
| Fe | 60.4897273 | 48.5692182 | 50.0139746 |
| O | 61.6087852 | 47.1767950 | 50.3331482 |
| C | 60.8169243 | 49.6563929 | 52.2596752 |
| O | 61.5548860 | 50.0751092 | 51.3156302 |
| C | 60.8824014 | 50.2636007 | 53.6388389 |
| C | 61.0324105 | 51.7877168 | 53.6487601 |
| C | 61.2703704 | 52.3899556 | 55.0617318 |
| O | 61.0817955 | 51.6418256 | 56.0492590 |
| O | 61.6268873 | 53.5900022 | 55.0733409 |
| H | 61.7606502 | 49.8307470 | 54.1494371 |
| H | 60.0082674 | 49.9362152 | 54.2142422 |
| H | 61.8631991 | 52.1089576 | 53.0017206 |
| H | 60.1247124 | 52.2720649 | 53.2445364 |
| O | 60.0068414 | 48.7025052 | 52.0014066 |
| C | 63.4568903 | 44.2214876 | 52.2056829 |
| N | 63.3768325 | 44.9333400 | 53.5283010 |
| H | 64.2554561 | 44.6951440 | 51.6191272 |
| H | 62.5034145 | 44.3984164 | 51.6848724 |
| H | 64.2845332 | 44.8376639 | 54.0196850 |
| C | 63.1585162 | 46.4302194 | 53.3076049 |
| H | 62.1121182 | 46.5336165 | 52.9845085 |
| C | 63.4471553 | 47.2403706 | 54.5128828 |
| H | 63.7875987 | 46.7149380 | 52.4377539 |
| C | 62.3090446 | 44.4068610 | 54.4444166 |
| H | 62.4837770 | 43.3394533 | 54.6576126 |
| H | 62.3505004 | 44.9875191 | 55.3758932 |
| H | 61.3414948 | 44.5667365 | 53.9357195 |
| H | 62.8751880 | 48.1506945 | 54.6944841 |
| H | 61.1697291 | 46.6579194 | 51.0215526 |
| H | 64.3721635 | 47.1223066 | 55.0814359 |
| H | 62.5346942 | 49.3717072 | 44.2566797 |
| H | 59.0475475 | 44.6288006 | 47.0221597 |
| H | 57.3881612 | 52.1515309 | 46.1402840 |
| H | 63.6405163 | 43.1485814 | 52.2624369 |

MEL2-IM1Y239A-ethCα

| | | | |
|---|---|---|---|
| N | 62.9227574 | 50.3160381 | 47.1747081 |
| C | 62.6872350 | 49.1680893 | 46.4412113 |
| C | 63.1820355 | 48.9672724 | 45.0503437 |
| N | 61.7624785 | 48.9897790 | 48.4761912 |
| C | 61.9678265 | 48.3459923 | 47.2772019 |
| C | 62.3547778 | 50.1707305 | 48.3843540 |
| H | 64.1722347 | 49.4288113 | 44.9335173 |
| H | 63.3169439 | 47.8929846 | 44.8647017 |
| H | 61.5955203 | 47.3433211 | 47.0835480 |
| H | 62.3871261 | 50.9028134 | 49.1825531 |
| H | 63.4073402 | 51.1627479 | 46.8471914 |
| C | 58.1286896 | 44.4644557 | 47.5899298 |
| C | 57.6365268 | 45.7072801 | 48.3461390 |
| C | 58.6173853 | 46.1473289 | 49.4397800 |
| O | 58.9737536 | 45.3194239 | 50.3000496 |
| O | 59.0191699 | 47.3580371 | 49.3687927 |
| H | 58.2960571 | 43.6587119 | 48.3216278 |
| H | 57.3648652 | 44.0997152 | 46.8865218 |
| H | 57.4322661 | 46.5520288 | 47.6722013 |
| H | 56.6871438 | 45.4413542 | 48.8423754 |
| N | 58.4587538 | 52.1536188 | 49.1089710 |
| C | 57.8987452 | 51.3456726 | 48.1316712 |
| C | 56.9514667 | 51.8767245 | 47.1001948 |
| N | 59.2649902 | 50.1428761 | 49.4658090 |
| C | 58.4187208 | 50.0958471 | 48.3769173 |
| C | 59.2695430 | 51.3985577 | 49.8847167 |
| H | 56.1693525 | 51.1378487 | 46.8710535 |
| H | 56.4339376 | 52.7607920 | 47.5018780 |
| H | 58.2460703 | 49.1689930 | 47.8382542 |
| H | 59.8279405 | 51.7904179 | 50.7326770 |
| H | 58.2504353 | 53.1367460 | 49.2651302 |
| Fe | 60.5559001 | 48.4150961 | 50.1134780 |
| O | 61.7020471 | 46.9589244 | 50.4463545 |
| C | 60.8191286 | 49.5580283 | 52.3512565 |
| O | 61.5468313 | 49.9031238 | 51.3639923 |
| C | 60.9290297 | 50.2309994 | 53.6903167 |
| C | 61.1061893 | 51.7510524 | 53.6369671 |
| C | 61.2920208 | 52.4046824 | 55.0359050 |
| O | 61.0703348 | 51.6912778 | 56.0419277 |
| O | 61.6424583 | 53.6058365 | 55.0143382 |
| H | 61.8040595 | 49.7966071 | 54.2054841 |
| H | 60.0576017 | 49.9511019 | 54.2938410 |
| H | 61.9680588 | 52.0353607 | 53.0133900 |
| H | 60.2241267 | 52.2326212 | 53.1773629 |
| O | 59.9849508 | 48.6122122 | 52.1506601 |
| C | 63.6288831 | 44.1929727 | 52.0951693 |
| N | 63.5530906 | 44.9243725 | 53.4103463 |
| H | 64.4872930 | 44.5962616 | 51.5421906 |
| H | 62.7117135 | 44.4323901 | 51.5357511 |
| H | 64.3831254 | 44.6771271 | 53.9853795 |
| C | 63.6471100 | 46.3826897 | 53.1900801 |
| H | 61.3558026 | 46.4187298 | 51.1779282 |

| | | | |
|---|---|---|---|
| C | 63.4881051 | 47.3135515 | 54.3247283 |
| H | 63.6429819 | 46.6931232 | 52.1401579 |
| C | 62.3358171 | 44.5493806 | 54.2299596 |
| H | 62.3331844 | 43.4584429 | 54.3825379 |
| H | 62.4111599 | 45.0514180 | 55.2016179 |
| H | 61.4422406 | 44.8987148 | 53.6814710 |
| H | 62.4207622 | 47.4871395 | 54.5640388 |
| H | 63.9136023 | 48.2909607 | 54.0590527 |
| H | 63.9847157 | 46.9772607 | 55.2488740 |
| H | 62.5474838 | 49.3891432 | 44.2709617 |
| H | 59.0408850 | 44.6375808 | 47.0189706 |
| H | 57.3928913 | 52.1572255 | 46.1438838 |
| H | 63.7430682 | 43.1127677 | 52.1856460 |

MEL1-RCN344A

| | | | |
|---|---|---|---|
| N | 64.9497535 | 45.7971648 | 55.2481171 |
| C | 65.2616915 | 45.6163776 | 53.9141703 |
| C | 66.6469295 | 45.7228675 | 53.3685038 |
| N | 63.0504235 | 45.4636556 | 54.2262390 |
| C | 64.0555003 | 45.3961917 | 53.2909387 |
| C | 63.6189596 | 45.7055306 | 55.3980182 |
| H | 67.3662940 | 45.2790090 | 54.0693782 |
| H | 66.7283992 | 45.1526465 | 52.4312042 |
| H | 63.8539642 | 45.2076359 | 52.2399386 |
| H | 63.0945553 | 45.7924727 | 56.3446858 |
| H | 65.5957614 | 45.9908706 | 56.0249860 |
| C | 61.1660818 | 45.8118190 | 48.7109360 |
| C | 60.4527626 | 46.4119091 | 49.9253121 |
| C | 60.2564979 | 45.3997975 | 51.0698298 |
| O | 59.8024968 | 44.2841593 | 50.8477286 |
| O | 60.6162350 | 45.8765036 | 52.2230098 |
| H | 60.5766604 | 44.9495850 | 48.3615367 |
| H | 61.2398163 | 46.5329967 | 47.8854935 |
| H | 60.9649951 | 47.3058267 | 50.3101325 |
| H | 59.4426567 | 46.7258452 | 49.6070189 |
| N | 60.5471576 | 48.9857931 | 55.7302371 |
| C | 61.1406636 | 49.3726026 | 54.5392635 |
| C | 61.4745694 | 50.8039271 | 54.2480988 |
| N | 60.8163196 | 47.1439451 | 54.5796925 |
| C | 61.3000763 | 48.2016064 | 53.8361823 |
| C | 60.3672533 | 47.6486080 | 55.7181789 |
| H | 61.1203816 | 51.1014945 | 53.2492010 |
| H | 60.9476377 | 51.4501385 | 54.9652333 |
| H | 61.7349260 | 48.0485700 | 52.8534277 |
| H | 59.9101303 | 47.0996761 | 56.5369932 |
| H | 60.2500531 | 49.5994392 | 56.4848585 |
| Fe | 61.0227448 | 45.1329632 | 53.9604683 |
| O | 61.3196211 | 43.6086646 | 53.4837056 |
| C | 59.7376500 | 44.3958853 | 56.0085461 |
| O | 60.9778482 | 44.6517313 | 56.0938365 |
| C | 58.9763945 | 43.9585886 | 57.2262253 |
| C | 58.6596801 | 45.1770151 | 58.1155184 |
| C | 58.1855329 | 44.7302642 | 59.5225246 |

| | | | |
|---|---|---|---|
| O | 59.0979395 | 44.2828985 | 60.2606050 |
| O | 56.9646346 | 44.8448491 | 59.7755717 |
| H | 59.6096115 | 43.2688760 | 57.8058176 |
| H | 58.0526852 | 43.4381921 | 56.9335033 |
| H | 59.5879956 | 45.7533289 | 58.2631426 |
| H | 57.9041382 | 45.8215514 | 57.6427547 |
| O | 59.1818109 | 44.5929417 | 54.8805156 |
| C | 59.6569589 | 38.4789236 | 52.6234839 |
| N | 59.2576305 | 39.9231483 | 52.5948366 |
| H | 58.8142813 | 37.9118653 | 52.2030856 |
| H | 59.7886757 | 38.1873407 | 53.6753971 |
| H | 59.2428522 | 40.2647373 | 51.6133995 |
| C | 57.8511172 | 40.0564120 | 53.1368878 |
| H | 57.9084053 | 39.7363738 | 54.1891312 |
| C | 57.3000725 | 41.4597080 | 52.9901027 |
| H | 57.2537701 | 39.3158466 | 52.5816010 |
| C | 60.2186134 | 40.8199711 | 53.3145854 |
| H | 60.1193788 | 40.6405776 | 54.3936168 |
| H | 61.2406467 | 40.5731723 | 52.9968552 |
| H | 60.0052796 | 41.8662543 | 53.0722529 |
| H | 57.3678458 | 41.8015113 | 51.9458190 |
| H | 57.8119113 | 42.1948688 | 53.6269799 |
| H | 56.2398347 | 41.4566100 | 53.2765040 |
| H | 66.9637328 | 46.7522107 | 53.2007082 |
| H | 62.1871020 | 45.4922602 | 48.9194232 |
| H | 62.5323425 | 51.0643810 | 54.2847982 |
| H | 60.5641338 | 38.2600969 | 52.0602650 |

MEL1-TS1N344A-meth

| | | | |
|---|---|---|---|
| N | 64.9123049 | 45.6561907 | 55.2163023 |
| C | 65.2370933 | 45.5258974 | 53.8780602 |
| C | 66.6224423 | 45.6803808 | 53.3444720 |
| N | 63.0352665 | 45.2211822 | 54.1789950 |
| C | 64.0473244 | 45.2507420 | 53.2457869 |
| C | 63.5908040 | 45.4733431 | 55.3582205 |
| H | 67.3452812 | 45.2417348 | 54.0451170 |
| H | 66.7242800 | 45.1234748 | 52.4010920 |
| H | 63.8587541 | 45.0894667 | 52.1882127 |
| H | 63.0581677 | 45.5152106 | 56.3029672 |
| H | 65.5430010 | 45.8839821 | 55.9974371 |
| C | 61.1714247 | 45.8705784 | 48.6840523 |
| C | 60.4653696 | 46.4507212 | 49.9151630 |
| C | 60.2259188 | 45.3935931 | 51.0067438 |
| O | 59.6440170 | 44.3502818 | 50.7324471 |
| O | 60.6953537 | 45.7275060 | 52.1684916 |
| H | 60.5669113 | 45.0296973 | 48.3094370 |
| H | 61.2617401 | 46.6128976 | 47.8789268 |
| H | 61.0037941 | 47.3110974 | 50.3387223 |
| H | 59.4690091 | 46.8041033 | 49.5971056 |
| N | 60.5140764 | 48.9147240 | 55.7084291 |
| C | 61.1443169 | 49.2930768 | 54.5343034 |
| C | 61.4653621 | 50.7283752 | 54.2410045 |

| | | | |
|---|---|---|---|
| N | 60.8113699 | 47.0602435 | 54.5757994 |
| C | 61.3225766 | 48.1141624 | 53.8451354 |
| C | 60.3296527 | 47.5767408 | 55.6940758 |
| H | 61.1081617 | 51.0209455 | 53.2413638 |
| H | 60.9302808 | 51.3704866 | 54.9567732 |
| H | 61.7889756 | 47.9552401 | 52.8770503 |
| H | 59.8392254 | 47.0379723 | 56.5005422 |
| H | 60.1958444 | 49.5327936 | 56.4504620 |
| Fe | 60.9741117 | 44.9284103 | 53.9158255 |
| O | 61.1688174 | 43.2040539 | 53.4094878 |
| C | 59.7271304 | 44.2882031 | 56.0330571 |
| O | 60.9800397 | 44.4665451 | 56.0815935 |
| C | 58.9656015 | 43.8898676 | 57.2605215 |
| C | 58.6396569 | 45.1394175 | 58.1031421 |
| C | 58.1597001 | 44.7377979 | 59.5215829 |
| O | 59.0653507 | 44.2916362 | 60.2684772 |
| O | 56.9420118 | 44.8817104 | 59.7731491 |
| H | 59.5972456 | 43.2235773 | 57.8676678 |
| H | 58.0411642 | 43.3606838 | 56.9840253 |
| H | 59.5648287 | 45.7244679 | 58.2361411 |
| H | 57.8859422 | 45.7643153 | 57.6024375 |
| O | 59.1516637 | 44.5287730 | 54.9177273 |
| C | 59.7480615 | 38.6506465 | 52.6149332 |
| N | 59.5117148 | 40.1445025 | 52.6775506 |
| H | 58.8427934 | 38.2021961 | 52.1756891 |
| H | 59.8570209 | 38.2928895 | 53.6485231 |
| H | 59.5637161 | 40.5393635 | 51.7142998 |
| C | 58.1076392 | 40.3953642 | 53.1870300 |
| H | 58.0852664 | 39.9766081 | 54.2055773 |
| C | 57.7220667 | 41.8580178 | 53.1572193 |
| H | 57.4529757 | 39.7823761 | 52.5507309 |
| C | 60.5381602 | 40.8327606 | 53.4999985 |
| H | 60.3145652 | 40.7117709 | 54.5680395 |
| H | 61.5544723 | 40.5127981 | 53.2317323 |
| H | 60.6315778 | 42.1740080 | 53.3496978 |
| H | 57.8539718 | 42.2953891 | 52.1562559 |
| H | 58.2973682 | 42.4707287 | 53.8644097 |
| H | 56.6606483 | 41.9432468 | 53.4312369 |
| H | 66.9269079 | 46.7155762 | 53.1904000 |
| H | 62.1859452 | 45.5296337 | 48.8903820 |
| H | 62.5179709 | 51.0088459 | 54.2786622 |
| H | 60.6217885 | 38.3700231 | 52.0267847 |

MEL1-TS1-ethN344A

| | | | |
|---|---|---|---|
| N | 64.8354877 | 45.7247518 | 55.2279883 |
| C | 65.1496386 | 45.6006447 | 53.8881456 |
| C | 66.5422161 | 45.7041690 | 53.3561050 |
| N | 62.9328768 | 45.4098038 | 54.1893602 |
| C | 63.9433448 | 45.3999520 | 53.2548729 |
| C | 63.5039656 | 45.6120930 | 55.3698128 |
| H | 67.2518175 | 45.2475331 | 54.0599667 |
| H | 66.6289617 | 45.1404706 | 52.4151899 |
| H | 63.7456148 | 45.2646107 | 52.1953313 |
| H | 62.9776579 | 45.6629891 | 56.3178877 |

| | | | |
|---|---|---|---|
| H | 65.4792941 | 45.9117160 | 56.0086867 |
| C | 61.1663858 | 45.9349062 | 48.6535667 |
| C | 60.4877278 | 46.5843743 | 49.8628772 |
| C | 60.2253214 | 45.5930084 | 51.0116927 |
| O | 59.7099911 | 44.5035404 | 50.7696393 |
| O | 60.5834098 | 46.0372243 | 52.1711568 |
| H | 60.5426045 | 45.0891521 | 48.3241930 |
| H | 61.2656046 | 46.6399028 | 47.8170125 |
| H | 61.0455224 | 47.4528961 | 50.2433362 |
| H | 59.4974178 | 46.9485656 | 49.5364943 |
| N | 60.4747683 | 49.1031747 | 55.6766949 |
| C | 61.1629018 | 49.4696326 | 54.5314479 |
| C | 61.5166997 | 50.8964815 | 54.2481523 |
| N | 60.8134177 | 47.2496545 | 54.5712774 |
| C | 61.3700873 | 48.2899887 | 53.8567082 |
| C | 60.2775915 | 47.7698094 | 55.6652511 |
| H | 61.1640480 | 51.2093151 | 53.2529830 |
| H | 61.0007672 | 51.5418343 | 54.9725382 |
| H | 61.8769096 | 48.1187705 | 52.9122516 |
| H | 59.7445262 | 47.2380029 | 56.4481601 |
| H | 60.1414891 | 49.7261408 | 56.4080057 |
| Fe | 60.8654333 | 45.2979402 | 53.9684131 |
| O | 60.9870331 | 43.5957116 | 53.4890852 |
| C | 59.6560037 | 44.5745366 | 56.0761995 |
| O | 60.9023846 | 44.7267163 | 56.1646137 |
| C | 58.8622818 | 44.1207760 | 57.2656068 |
| C | 58.5969498 | 45.3207819 | 58.1953393 |
| C | 58.1296794 | 44.8291373 | 59.5862931 |
| O | 59.0448153 | 44.3571532 | 60.3052970 |
| O | 56.9077889 | 44.9240995 | 59.8416961 |
| H | 59.4631398 | 43.3848223 | 57.8217990 |
| H | 57.9172553 | 43.6522335 | 56.9534751 |
| H | 59.5445274 | 45.8637463 | 58.3441224 |
| H | 57.8509613 | 46.0003393 | 57.7575851 |
| O | 59.0970405 | 44.8867830 | 54.9648095 |
| C | 59.9347683 | 38.7019670 | 52.3908486 |
| N | 60.0379668 | 40.2027588 | 52.4802033 |
| H | 58.9233017 | 38.4274613 | 52.0458395 |
| H | 60.0776473 | 38.3134886 | 53.4095755 |
| H | 59.9170735 | 40.6175378 | 51.5329121 |
| C | 58.9442969 | 40.7780504 | 53.3542406 |
| H | 59.1464438 | 40.4233369 | 54.3799673 |
| C | 58.8099152 | 42.2749431 | 53.2788164 |
| H | 58.0041683 | 40.3032336 | 53.0207046 |
| C | 61.3798661 | 40.6335498 | 52.9874909 |
| H | 61.5388699 | 40.1841395 | 53.9776082 |
| H | 62.1575664 | 40.2896172 | 52.2887137 |
| H | 61.4096252 | 41.7216764 | 53.0635206 |
| H | 58.5381778 | 42.7065494 | 52.3073457 |
| H | 59.9698672 | 43.0150094 | 53.4536718 |
| H | 58.2046717 | 42.6728934 | 54.0985217 |
| H | 66.8804656 | 46.7281790 | 53.1978080 |
| H | 62.1766678 | 45.5835187 | 48.8631476 |
| H | 62.5793138 | 51.1363835 | 54.2851047 |

| | | | |
|---|---|---|---|
| H | 60.6912059 | 38.2650838 | 51.7389266 |

MEL1-IM1-methN344A

| | | | |
|---|---|---|---|
| N | 64.8948238 | 45.7735331 | 55.2249706 |
| C | 65.2207976 | 45.6220621 | 53.8889743 |
| C | 66.6133160 | 45.7296528 | 53.3604092 |
| N | 63.0019374 | 45.4480263 | 54.1749723 |
| C | 64.0211981 | 45.4119210 | 53.2504205 |
| C | 63.5635918 | 45.6682596 | 55.3573347 |
| H | 67.3251732 | 45.2813451 | 54.0666649 |
| H | 66.7041653 | 45.1618590 | 52.4224679 |
| H | 63.8341817 | 45.2426915 | 52.1934266 |
| H | 63.0299496 | 45.7359675 | 56.3006556 |
| H | 65.5338399 | 45.9622883 | 56.0095122 |
| C | 61.1878986 | 45.8299418 | 48.6280469 |
| C | 60.4471776 | 46.5216594 | 49.7755596 |
| C | 60.2110898 | 45.5967454 | 50.9750003 |
| O | 59.7766067 | 44.4483824 | 50.7936841 |
| O | 60.5219785 | 46.1017990 | 52.1149584 |
| H | 60.5920184 | 44.9605402 | 48.3091903 |
| H | 61.3065646 | 46.4984793 | 47.7645220 |
| H | 60.9616661 | 47.4309849 | 50.1195790 |
| H | 59.4537623 | 46.8305021 | 49.4041307 |
| N | 60.5840091 | 49.1833453 | 55.7413042 |
| C | 61.1500511 | 49.5678499 | 54.5370009 |
| C | 61.5240913 | 50.9868349 | 54.2504476 |
| N | 60.7804243 | 47.3473641 | 54.5618418 |
| C | 61.2633469 | 48.4023914 | 53.8178249 |
| C | 60.3760811 | 47.8488789 | 55.7171209 |
| H | 61.1794271 | 51.2972044 | 53.2523458 |
| H | 61.0159402 | 51.6439662 | 54.9686017 |
| H | 61.6634704 | 48.2534302 | 52.8193534 |
| H | 59.9227782 | 47.2991584 | 56.5387301 |
| H | 60.3353178 | 49.7958439 | 56.5138602 |
| Fe | 60.9236293 | 45.2562601 | 53.8918629 |
| O | 61.0381318 | 43.5530172 | 53.0666829 |
| C | 59.6571727 | 44.4875322 | 55.9347772 |
| O | 60.9217617 | 44.6426178 | 55.9591436 |
| C | 58.9339213 | 44.0287869 | 57.1642208 |
| C | 58.6626019 | 45.2307594 | 58.0937418 |
| C | 58.1868594 | 44.7561976 | 59.4928697 |
| O | 59.0979140 | 44.3194141 | 60.2381193 |
| O | 56.9601527 | 44.8398764 | 59.7322057 |
| H | 59.5752638 | 43.3131785 | 57.7012422 |
| H | 57.9910973 | 43.5331326 | 56.8891132 |
| H | 59.6062317 | 45.7816561 | 58.2416432 |
| H | 57.9152761 | 45.9037426 | 57.6483882 |
| O | 59.0644672 | 44.8038726 | 54.8537975 |
| C | 59.5949586 | 38.3922454 | 52.4859054 |
| N | 59.2124068 | 39.8469274 | 52.5834518 |
| H | 58.7787063 | 37.8895377 | 51.9476231 |
| H | 59.6370122 | 37.9935779 | 53.5102268 |
| H | 59.2616182 | 40.2945622 | 51.6457482 |

| | | | |
|---|---|---|---|
| C | 57.7622090 | 39.9538715 | 53.0582998 |
| H | 57.7740096 | 39.6102965 | 54.1035835 |
| C | 57.2441892 | 41.3679095 | 52.9143042 |
| H | 57.1977384 | 39.2221466 | 52.4563275 |
| C | 60.0963024 | 40.5961942 | 53.4613288 |
| H | 60.1751067 | 40.2046443 | 54.4768732 |
| H | 60.4375947 | 41.5903361 | 53.1664233 |
| H | 60.4171515 | 43.6254944 | 52.3073632 |
| H | 57.2412289 | 41.6746820 | 51.8570975 |
| H | 57.8387827 | 42.0968247 | 53.4851683 |
| H | 56.2129547 | 41.4175410 | 53.2871632 |
| H | 66.9364525 | 46.7578478 | 53.1977285 |
| H | 62.1950796 | 45.5023318 | 48.8855840 |
| H | 62.5922872 | 51.2005559 | 54.2871614 |
| H | 60.5472611 | 38.2154297 | 51.9859821 |

MEL1-IM1-ethN344A

| | | | |
|---|---|---|---|
| N | 64.9399818 | 45.7936086 | 55.2185529 |
| C | 65.2628275 | 45.6667476 | 53.8807117 |
| C | 66.6566881 | 45.7638868 | 53.3535249 |
| N | 63.0390036 | 45.5622296 | 54.1568500 |
| C | 64.0573905 | 45.5150974 | 53.2356547 |
| C | 63.6030915 | 45.7309895 | 55.3439839 |
| H | 67.3663200 | 45.3152238 | 54.0613616 |
| H | 66.7480631 | 45.1951762 | 52.4162537 |
| H | 63.8692651 | 45.3843542 | 52.1732937 |
| H | 63.0695187 | 45.7854329 | 56.2880463 |
| H | 65.5806716 | 45.9507235 | 56.0079808 |
| C | 61.1762319 | 45.8547845 | 48.6480478 |
| C | 60.4417927 | 46.5359302 | 49.8045954 |
| C | 60.2509941 | 45.6193043 | 51.0199335 |
| O | 59.8486910 | 44.4555194 | 50.8689645 |
| O | 60.5671213 | 46.1673537 | 52.1381378 |
| H | 60.5790091 | 44.9856066 | 48.3296876 |
| H | 61.2837747 | 46.5289787 | 47.7873970 |
| H | 60.9409936 | 47.4597639 | 50.1316346 |
| H | 59.4348252 | 46.8187912 | 49.4489568 |
| N | 60.6175508 | 49.1335539 | 55.7579618 |
| C | 61.1605231 | 49.5388543 | 54.5492926 |
| C | 61.5134148 | 50.9652308 | 54.2701371 |
| N | 60.8375835 | 47.3161386 | 54.5665218 |
| C | 61.2872662 | 48.3836112 | 53.8179648 |
| C | 60.4374073 | 47.7969657 | 55.7333173 |
| H | 61.1572698 | 51.2792494 | 53.2773039 |
| H | 61.0047216 | 51.6109049 | 54.9988854 |
| H | 61.6761826 | 48.2450809 | 52.8145837 |
| H | 60.0074793 | 47.2298635 | 56.5546179 |
| H | 60.3571120 | 49.7360857 | 56.5348566 |
| Fe | 60.9516392 | 45.4042405 | 53.9061999 |
| O | 61.1189988 | 43.7289664 | 53.2120793 |
| C | 59.6991521 | 44.5438419 | 55.9206717 |
| O | 60.9606065 | 44.6815523 | 55.9428060 |
| C | 58.9776893 | 44.0548407 | 57.1466837 |

| | | | |
|---|---|---|---|
| C | 58.6881951 | 45.2408401 | 58.0890582 |
| C | 58.2067856 | 44.7574713 | 59.4824177 |
| O | 59.1143159 | 44.3133719 | 60.2285276 |
| O | 56.9800761 | 44.8454470 | 59.7222192 |
| H | 59.6223790 | 43.3370676 | 57.6757851 |
| H | 58.0395330 | 43.5548170 | 56.8631718 |
| H | 59.6270667 | 45.7970275 | 58.2508374 |
| H | 57.9405920 | 45.9138789 | 57.6443591 |
| O | 59.0891002 | 44.9130180 | 54.8678154 |
| C | 59.7324389 | 38.4106920 | 52.4425284 |
| N | 59.4184172 | 39.8725368 | 52.5458318 |
| H | 58.8591121 | 37.9289479 | 51.9784300 |
| H | 59.8340301 | 38.0287048 | 53.4691903 |
| H | 59.2495867 | 40.2725800 | 51.6024033 |
| C | 58.1327268 | 40.0416278 | 53.3476798 |
| H | 58.3532127 | 39.6523852 | 54.3562283 |
| C | 57.6689290 | 41.4467588 | 53.3879177 |
| H | 57.4049464 | 39.3620683 | 52.8623072 |
| C | 60.5390112 | 40.6788228 | 53.1246186 |
| H | 60.8562170 | 40.2210189 | 54.0727466 |
| H | 61.3734003 | 40.6577647 | 52.4077071 |
| H | 60.2048593 | 41.7077865 | 53.2961549 |
| H | 57.6637036 | 42.0560190 | 52.4816110 |
| H | 60.5354368 | 43.7422337 | 52.4213389 |
| H | 57.3286918 | 41.8942369 | 54.3226286 |
| H | 66.9781206 | 46.7926206 | 53.1908747 |
| H | 62.1868139 | 45.5270026 | 48.8916667 |
| H | 62.5794856 | 51.1904264 | 54.2992454 |
| H | 60.6438017 | 38.1726094 | 51.8940745 |

MEL2-RCN344A

| | | | |
|---|---|---|---|
| N | 64.7107866 | 46.5921807 | 55.0044903 |
| C | 65.1503171 | 45.9284037 | 53.8737900 |
| C | 66.5805535 | 45.6320941 | 53.5535550 |
| N | 62.9226568 | 46.2075350 | 53.7905512 |
| C | 64.0157257 | 45.6894068 | 53.1344299 |
| C | 63.3753509 | 46.7460270 | 54.9155872 |
| H | 67.1355937 | 45.3009842 | 54.4445351 |
| H | 66.6253217 | 44.8003722 | 52.8344379 |
| H | 63.9222892 | 45.2135645 | 52.1625311 |
| H | 62.7745702 | 47.2322984 | 55.6801412 |
| H | 65.2893835 | 46.9231472 | 55.7887567 |
| C | 61.5827097 | 45.5624531 | 48.1099746 |
| C | 61.0884000 | 46.8094589 | 48.8650631 |
| C | 60.9076231 | 46.6138383 | 50.3620311 |
| O | 59.7883689 | 46.6141362 | 50.9147753 |
| O | 61.9633293 | 46.4470587 | 51.0666711 |
| H | 60.8064899 | 44.7812447 | 48.1579343 |
| H | 61.7430550 | 45.8042814 | 47.0523572 |
| H | 61.7814867 | 47.6557783 | 48.7229850 |
| H | 60.1164886 | 47.1074291 | 48.4440831 |
| N | 60.7427660 | 50.3683446 | 53.5752548 |
| C | 61.8302157 | 50.4116584 | 52.7189532 |
| C | 62.5021441 | 51.6997216 | 52.3711439 |

| | | | |
|---|---|---|---|
| N | 61.1121619 | 48.2991943 | 52.9782995 |
| C | 62.0475434 | 49.1019883 | 52.3601783 |
| C | 60.3337267 | 49.0906242 | 53.7025162 |
| H | 62.3441370 | 51.9714281 | 51.3149682 |
| H | 62.0534665 | 52.5071273 | 52.9652693 |
| H | 62.8193622 | 48.6809634 | 51.7218298 |
| H | 59.4892069 | 48.7719699 | 54.3059638 |
| H | 60.3046483 | 51.1600465 | 54.0414610 |
| Fe | 60.9914717 | 46.2231049 | 52.8607181 |
| O | 60.9088797 | 44.6039438 | 52.7105758 |
| C | 59.7600986 | 46.0379298 | 55.4516450 |
| O | 60.5484077 | 45.1751368 | 55.8055693 |
| C | 58.9430779 | 46.8685929 | 56.4329374 |
| C | 58.2968555 | 46.1377976 | 57.6127636 |
| C | 57.8457376 | 47.2069690 | 58.6371957 |
| O | 58.7475001 | 47.6489818 | 59.3927442 |
| O | 56.6530519 | 47.5982233 | 58.5851903 |
| H | 58.1926449 | 47.4613524 | 55.8906514 |
| H | 59.6678756 | 47.5947323 | 56.8508065 |
| H | 57.4350212 | 45.5425566 | 57.2736178 |
| H | 59.0370801 | 45.4713435 | 58.0795073 |
| O | 59.6086594 | 46.4291031 | 54.1949399 |
| C | 59.1362909 | 39.6422902 | 52.4062052 |
| N | 58.6549938 | 40.8106209 | 53.2199548 |
| H | 59.4471237 | 40.0411392 | 51.4280163 |
| H | 58.2671749 | 38.9864784 | 52.2483432 |
| H | 59.4911919 | 41.3656668 | 53.4898589 |
| C | 57.7738088 | 41.7118250 | 52.3743880 |
| H | 56.8549301 | 41.1275300 | 52.2068583 |
| C | 57.5128627 | 43.0642017 | 53.0150553 |
| H | 58.2884558 | 41.8156424 | 51.4036605 |
| C | 57.9610828 | 40.4019875 | 54.4820441 |
| H | 57.8313229 | 41.2664088 | 55.1484249 |
| H | 56.9842576 | 39.9817413 | 54.2071698 |
| H | 58.5439356 | 39.6358840 | 55.0124310 |
| H | 56.8378355 | 43.6370299 | 52.3632729 |
| H | 58.4383140 | 43.6541891 | 53.1106950 |
| H | 57.0324688 | 42.9906894 | 54.0011644 |
| H | 67.1223501 | 46.4897657 | 53.1549035 |
| H | 62.5208180 | 45.1241841 | 48.4504746 |
| H | 63.5795666 | 51.7136615 | 52.5355632 |
| H | 59.9699654 | 39.1049591 | 52.8582330 |

MEL2-TS10-methN344A

| | | | |
|---|---|---|---|
| N | 64.7094284 | 46.4372032 | 55.0531927 |
| C | 65.1560491 | 45.8349956 | 53.8896430 |
| C | 66.5884182 | 45.5894352 | 53.5385928 |
| N | 62.9137309 | 45.9926583 | 53.8699829 |
| C | 64.0198134 | 45.5615042 | 53.1682682 |
| C | 63.3677649 | 46.5164172 | 55.0042241 |
| H | 67.1654686 | 45.2508328 | 54.4125363 |
| H | 66.6438817 | 44.7740873 | 52.8010281 |
| H | 63.9311590 | 45.1064627 | 52.1872255 |
| H | 62.7573672 | 46.9324607 | 55.8003654 |

| | | | | | | | |
|---|---|---|---|---|---|---|---|
| H | 65.2874475 | 46.7899016 | 55.8289468 | C | 57.7611114 | 47.1881841 | 58.7727081 |
| C | 61.5990953 | 45.4106690 | 48.2141069 | O | 58.6368212 | 47.6425869 | 59.5504959 |
| C | 61.0539543 | 46.5949004 | 49.0322827 | O | 56.5609164 | 47.5552313 | 58.7016273 |
| C | 60.9185549 | 46.3359131 | 50.5223120 | H | 58.0323207 | 47.4228742 | 55.9891145 |
| O | 59.8105770 | 46.3352291 | 51.1144007 | H | 59.5027959 | 47.6813995 | 56.9346942 |
| O | 61.9803230 | 46.1337660 | 51.1961376 | H | 57.4107898 | 45.5012387 | 57.4276121 |
| H | 60.8714349 | 44.5845291 | 48.2558253 | H | 59.0328019 | 45.5226024 | 58.1945966 |
| H | 61.7028333 | 45.7030593 | 47.1615613 | O | 59.4399424 | 46.2586724 | 54.3071984 |
| H | 61.7009374 | 47.4819070 | 48.9170746 | C | 58.8389523 | 39.9297632 | 53.0808511 |
| H | 60.0623270 | 46.8647289 | 48.6385218 | N | 58.7847755 | 41.4241051 | 52.8583972 |
| N | 60.7074900 | 50.2597918 | 53.6154183 | H | 57.9823940 | 39.4900144 | 52.5435269 |
| C | 61.8213744 | 50.2798069 | 52.7931897 | H | 58.7174548 | 39.7484824 | 54.1595646 |
| C | 62.4722467 | 51.5693189 | 52.4030227 | H | 59.0800449 | 41.6011433 | 51.8749776 |
| N | 61.1010077 | 48.1673584 | 53.1025738 | C | 57.3644835 | 41.9313904 | 52.9627699 |
| C | 62.0534992 | 48.9565367 | 52.4899586 | H | 57.0391648 | 41.7203597 | 53.9936467 |
| C | 60.2995646 | 48.9839405 | 53.7703853 | C | 57.2533094 | 43.4038935 | 52.6130162 |
| H | 62.2992532 | 51.8081367 | 51.3408285 | H | 56.7767012 | 41.2971536 | 52.2829303 |
| H | 62.0150615 | 52.3877957 | 52.9772933 | C | 59.6927572 | 42.1603524 | 53.7675206 |
| H | 62.8511825 | 48.5210342 | 51.8931873 | H | 60.1108290 | 43.3757190 | 53.2691555 |
| H | 59.4315923 | 48.6892524 | 54.3527952 | H | 59.2138535 | 42.4130860 | 54.7220752 |
| H | 60.2517093 | 51.0646890 | 54.0405015 | H | 60.6614074 | 41.6600849 | 53.8711483 |
| Fe | 60.9494906 | 45.9806761 | 53.0257570 | H | 56.1917926 | 43.6905917 | 52.6173060 |
| O | 60.8294604 | 44.1620378 | 52.8805373 | H | 57.6662000 | 43.6149856 | 51.6141249 |
| C | 59.6810579 | 46.0803704 | 55.5885325 | H | 57.7701172 | 44.0561331 | 53.3317905 |
| O | 60.5931241 | 45.3624994 | 55.9926418 | H | 67.1066699 | 46.4665940 | 53.1511982 |
| C | 58.8258530 | 46.8997288 | 56.5397938 | H | 62.5693370 | 45.0069448 | 48.5034251 |
| C | 58.2459880 | 46.1446861 | 57.7435109 | H | 63.5502316 | 51.6214178 | 52.5556706 |

| | | | |
|---|---|---|---|
| H | 59.7645650 | 39.4749975 | 52.7280106 |

MEL2-TS1N344A-eth-Cα

| | | | |
|---|---|---|---|
| N | 64.7152978 | 46.4196270 | 54.9887745 |
| C | 65.1772032 | 45.8025599 | 53.8390121 |
| C | 66.6172335 | 45.5817911 | 53.5043282 |
| N | 62.9297399 | 45.8805591 | 53.8278456 |
| C | 64.0469926 | 45.4767334 | 53.1274792 |
| C | 63.3712283 | 46.4540238 | 54.9445062 |
| H | 67.1882024 | 45.2571415 | 54.3874613 |
| H | 66.6959219 | 44.7658231 | 52.7707945 |
| H | 63.9687985 | 45.0081756 | 52.1512697 |
| H | 62.7486588 | 46.8689345 | 55.7322525 |
| H | 65.2846913 | 46.8010287 | 55.7576820 |
| C | 61.5689698 | 45.4701619 | 48.1777543 |
| C | 61.0090740 | 46.6077966 | 49.0507769 |
| C | 60.8853890 | 46.2861253 | 50.5302397 |
| O | 59.7810728 | 46.2390443 | 51.1271517 |
| O | 61.9559535 | 46.0856979 | 51.1906989 |
| H | 60.8379886 | 44.6458699 | 48.1528660 |
| H | 61.7078878 | 45.8205056 | 47.1479666 |
| H | 61.6473690 | 47.5047988 | 48.9738745 |
| H | 60.0120127 | 46.8821473 | 48.6746802 |
| N | 60.6678432 | 50.1553429 | 53.6146555 |
| C | 61.7854283 | 50.1935053 | 52.7981420 |
| C | 62.4136975 | 51.4986530 | 52.4155230 |
| N | 61.0934270 | 48.0669053 | 53.1007418 |
| C | 62.0374415 | 48.8726945 | 52.4941456 |
| C | 60.2771953 | 48.8728581 | 53.7635032 |
| H | 62.2309228 | 51.7455840 | 51.3563673 |
| H | 61.9485329 | 52.3057161 | 53.0012468 |
| H | 62.8403759 | 48.4496764 | 51.8947864 |
| H | 59.4075885 | 48.5679309 | 54.3377819 |
| H | 60.1968536 | 50.9534250 | 54.0370573 |
| Fe | 60.9526952 | 45.8386603 | 53.0221926 |
| O | 60.7844298 | 44.0059057 | 52.8160834 |
| C | 59.7525555 | 46.0147859 | 55.5561536 |
| O | 60.7117473 | 45.3324698 | 55.9178528 |
| C | 58.9347408 | 46.8364032 | 56.5369769 |
| C | 58.2850483 | 46.0693549 | 57.6964271 |
| C | 57.7944819 | 47.1067083 | 58.7312001 |
| O | 58.6750774 | 47.5766116 | 59.4919867 |
| O | 56.5859222 | 47.4517442 | 58.6824308 |
| H | 58.1714731 | 47.4196851 | 56.0022625 |
| H | 59.6436203 | 47.5599686 | 56.9799682 |
| H | 57.4412202 | 45.4640206 | 57.3315001 |
| H | 59.0337437 | 45.4131586 | 58.1646217 |
| O | 59.4338304 | 46.1494361 | 54.2885321 |
| C | 59.4188424 | 40.1155166 | 52.4895319 |
| N | 59.0956703 | 41.2739521 | 53.4134782 |
| H | 59.8277375 | 40.5340686 | 51.5595366 |
| H | 58.4689693 | 39.6093855 | 52.2509627 |
| H | 59.9746328 | 41.5017069 | 53.9163481 |
| C | 58.7401670 | 42.5431732 | 52.6476746 |
| H | 58.6653637 | 42.2957759 | 51.5747916 |

| | | | |
|---|---|---|---|
| C | 57.6385094 | 43.4070566 | 53.1888245 |
| H | 59.7804064 | 43.3082223 | 52.6757065 |
| C | 58.0864748 | 40.8654988 | 54.4501456 |
| H | 57.9795460 | 41.6456575 | 55.2163014 |
| H | 57.1308579 | 40.6800486 | 53.9449761 |
| H | 58.4200206 | 39.9368092 | 54.9385968 |
| H | 57.6073945 | 44.3134524 | 52.5686713 |
| H | 57.8057637 | 43.7259647 | 54.2272975 |
| H | 56.6522431 | 42.9139669 | 53.1194981 |
| H | 67.1259512 | 46.4650182 | 53.1180856 |
| H | 62.5302925 | 45.0526654 | 48.4771242 |
| H | 63.4916651 | 51.5704797 | 52.5600705 |
| H | 60.1449587 | 39.4309632 | 52.9279483 |

MEL2-TS1N344A-eth-Cβ

| | | | |
|---|---|---|---|
| N | 64.6875657 | 46.5168712 | 55.0076262 |
| C | 65.1246661 | 45.8782131 | 53.8622218 |
| C | 66.5570636 | 45.6052312 | 53.5295565 |
| N | 62.8819751 | 46.0779772 | 53.8291764 |
| C | 63.9813654 | 45.6111129 | 53.1436780 |
| C | 63.3448947 | 46.6232776 | 54.9468693 |
| H | 67.1186889 | 45.2683866 | 54.4144154 |
| H | 66.6098927 | 44.7833265 | 52.7998477 |
| H | 63.8871103 | 45.1441984 | 52.1669946 |
| H | 62.7457554 | 47.0810509 | 55.7304094 |
| H | 65.2700677 | 46.8648864 | 55.7813714 |

| | | | |
|---|---|---|---|
| C | 61.5827116 | 45.5547685 | 48.1240951 |
| C | 61.0586017 | 46.7661291 | 48.9122150 |
| C | 60.8017094 | 46.5197914 | 50.3924085 |
| O | 59.6651427 | 46.5817320 | 50.8950441 |
| O | 61.8244333 | 46.2694674 | 51.1292628 |
| H | 60.8173912 | 44.7615331 | 48.1344421 |
| H | 61.7543772 | 45.8335642 | 47.0772343 |
| H | 61.7571205 | 47.6172816 | 48.8375610 |
| H | 60.1069799 | 47.0874833 | 48.4631449 |
| N | 60.7018962 | 50.2632100 | 53.5712113 |
| C | 61.8156290 | 50.2969503 | 52.7473214 |
| C | 62.4709910 | 51.5943506 | 52.3915347 |
| N | 61.0872208 | 48.1864244 | 53.0128765 |
| C | 62.0446825 | 48.9809189 | 52.4136219 |
| C | 60.2871619 | 48.9892359 | 53.7022207 |
| H | 62.3022987 | 51.8599208 | 51.3349563 |
| H | 62.0136559 | 52.3993705 | 52.9847617 |
| H | 62.8351504 | 48.5512904 | 51.8039920 |
| H | 59.4265816 | 48.6791581 | 54.2866882 |
| H | 60.2526678 | 51.0608886 | 54.0175687 |
| Fe | 60.9065428 | 46.1525177 | 52.9157696 |
| O | 60.7151325 | 44.3978698 | 52.7336837 |
| C | 59.7267090 | 46.0149769 | 55.5204333 |
| O | 60.5516026 | 45.1932698 | 55.8905427 |
| C | 58.9114862 | 46.8613184 | 56.4876109 |
| C | 58.2552495 | 46.1399811 | 57.6674625 |
| C | 57.8052889 | 47.2160708 | 58.6823992 |

| | | | |
|---|---|---|---|
| O | 58.7108552 | 47.6708577 | 59.4250519 |
| O | 56.6095407 | 47.5998153 | 58.6351570 |
| H | 58.1645472 | 47.4524957 | 55.9389110 |
| H | 59.6369256 | 47.5854679 | 56.9063149 |
| H | 57.3924396 | 45.5477414 | 57.3261023 |
| H | 58.9911672 | 45.4741320 | 58.1414049 |
| O | 59.5298904 | 46.3363505 | 54.2501707 |
| C | 59.2205529 | 39.8549099 | 52.4206228 |
| N | 58.8870612 | 41.0713144 | 53.2497194 |
| H | 59.5638165 | 40.2148323 | 51.4385020 |
| H | 58.2843611 | 39.2935294 | 52.2707936 |
| H | 59.7871309 | 41.4658392 | 53.5861680 |
| C | 58.2284562 | 42.1562848 | 52.4138543 |
| H | 57.1766753 | 41.8351318 | 52.2992007 |
| C | 58.3314266 | 43.5394507 | 52.9971538 |
| H | 58.6817411 | 42.1014857 | 51.4094298 |
| C | 58.0605400 | 40.7262377 | 54.4512321 |
| H | 57.9695374 | 41.5926725 | 55.1225164 |
| H | 57.0704683 | 40.4106127 | 54.0959906 |
| H | 58.5190663 | 39.8942065 | 55.0055240 |
| H | 57.8215410 | 44.2854862 | 52.3754983 |
| H | 59.6192321 | 44.0406914 | 52.8800374 |
| H | 58.1000625 | 43.6584493 | 54.0640893 |
| H | 67.0936237 | 46.4703533 | 53.1400256 |
| H | 62.5229138 | 45.1200083 | 48.4633157 |
| H | 63.5488063 | 51.6374484 | 52.5481359 |
| H | 60.0006295 | 39.2442192 | 52.8751604 |

MEL2-IM1N344A-meth

| | | | |
|---|---|---|---|
| N | 64.9302767 | 46.4341621 | 54.9855683 |
| C | 65.3604740 | 45.8383771 | 53.8124292 |
| C | 66.7928410 | 45.6060577 | 53.4595699 |
| N | 63.1235572 | 46.0437289 | 53.8034961 |
| C | 64.2160448 | 45.6018339 | 53.0894048 |
| C | 63.5931175 | 46.5440658 | 54.9413264 |
| H | 67.3692084 | 45.2579539 | 54.3295307 |
| H | 66.8602798 | 44.8046326 | 52.7084384 |
| H | 64.1108438 | 45.1673901 | 52.1000940 |
| H | 62.9900607 | 46.9692286 | 55.7367297 |
| H | 65.5099953 | 46.7630468 | 55.7690874 |
| C | 61.6271066 | 45.4932878 | 48.2671011 |
| C | 61.1506187 | 46.7235646 | 49.0608456 |
| C | 61.0339657 | 46.5137443 | 50.5632321 |
| O | 59.9219090 | 46.4674285 | 51.1426580 |
| O | 62.1049246 | 46.3882793 | 51.2385727 |
| H | 60.8697059 | 44.6962340 | 48.3492346 |
| H | 61.7201215 | 45.7502851 | 47.2037061 |
| H | 61.8267018 | 47.5806106 | 48.8997458 |
| H | 60.1589898 | 47.0149386 | 48.6821968 |
| N | 60.7770053 | 50.4376187 | 53.5916615 |
| C | 61.9236531 | 50.4176955 | 52.8170916 |
| C | 62.5972229 | 51.6836309 | 52.3970325 |
| N | 61.1890917 | 48.3233372 | 53.1951802 |

| | | | |
|---|---|---|---|
| C | 62.1682037 | 49.0832202 | 52.5889434 |
| C | 60.3594276 | 49.1704584 | 53.7856517 |
| H | 62.4282470 | 51.9034777 | 51.3301486 |
| H | 62.1541504 | 52.5203149 | 52.9534482 |
| H | 62.9832375 | 48.6197171 | 52.0392555 |
| H | 59.4559485 | 48.9164930 | 54.3312079 |
| H | 60.3122791 | 51.2603700 | 53.9690712 |
| Fe | 61.1024316 | 46.1442794 | 53.1184391 |
| O | 60.7437795 | 44.2896156 | 52.7482096 |
| C | 59.6479558 | 46.4448307 | 55.2442455 |
| O | 60.8328251 | 46.0473214 | 55.4094002 |
| C | 58.8493041 | 47.0448665 | 56.3756955 |
| C | 58.4084465 | 46.1166095 | 57.5223544 |
| C | 57.8434135 | 47.0299383 | 58.6402861 |
| O | 58.6562386 | 47.4071343 | 59.5185678 |
| O | 56.6444350 | 47.3883233 | 58.5221339 |
| H | 57.9671955 | 47.5505742 | 55.9604138 |
| H | 59.5011281 | 47.8221058 | 56.8136713 |
| H | 57.6307441 | 45.4206877 | 57.1711743 |
| H | 59.2659957 | 45.5421549 | 57.9034685 |
| O | 59.1907614 | 46.4492523 | 54.0498906 |
| C | 58.5463687 | 39.6999532 | 53.2139764 |
| N | 58.4268750 | 41.2030332 | 53.1007345 |
| H | 57.7786906 | 39.2664704 | 52.5538248 |
| H | 58.3392270 | 39.4183634 | 54.2599178 |
| H | 58.8611112 | 41.4836282 | 52.1973037 |
| C | 56.9781799 | 41.6602738 | 53.0094433 |
| H | 56.5191937 | 41.4281408 | 53.9825396 |
| C | 56.9121207 | 43.1400634 | 52.6725620 |
| H | 56.5090484 | 41.0191082 | 52.2470203 |
| C | 59.1028488 | 41.8859899 | 54.1948077 |
| H | 59.8447403 | 44.2916350 | 52.3902382 |
| H | 58.6586396 | 41.7398557 | 55.1836617 |
| H | 59.8620422 | 42.6334673 | 53.9520308 |
| H | 55.8690959 | 43.4784737 | 52.6526650 |
| H | 57.3513944 | 43.3445774 | 51.6837684 |
| H | 57.4316719 | 43.7634186 | 53.4164492 |
| H | 67.2941231 | 46.5024168 | 53.0944349 |
| H | 62.5895591 | 45.0686498 | 48.5524693 |
| H | 63.6754240 | 51.7083067 | 52.5549584 |
| H | 59.5367328 | 39.3513955 | 52.9211394 |

MEL2-IM1N344A-eth-Cα

| | | | |
|---|---|---|---|
| N | 64.9380440 | 46.4184166 | 54.9917168 |
| C | 65.3717405 | 45.8317935 | 53.8158217 |
| C | 66.8052405 | 45.6040679 | 53.4636152 |
| N | 63.1324598 | 46.0188423 | 53.8087863 |
| C | 64.2278856 | 45.5923462 | 53.0912630 |
| C | 63.5983253 | 46.5195549 | 54.9483121 |
| H | 67.3823725 | 45.2589670 | 54.3342452 |
| H | 66.8735668 | 44.8029499 | 52.7127346 |
| H | 64.1239675 | 45.1737277 | 52.0947867 |
| H | 62.9907446 | 46.9345238 | 55.7469276 |
| H | 65.5175088 | 46.7446352 | 55.7768000 |

| | | | | | | | |
|---|---|---|---|---|---|---|---|
| C | 61.6259777 | 45.5539265 | 48.2452694 | O | 58.6593528 | 47.2620626 | 59.5004068 |
| C | 61.1495094 | 46.7601997 | 49.0751039 | O | 56.6344096 | 47.2383258 | 58.5294932 |
| C | 61.0172862 | 46.4951915 | 50.5685183 | H | 58.0965936 | 47.5263683 | 56.0281342 |
| O | 59.8985327 | 46.4133763 | 51.1303923 | H | 59.6349216 | 47.6330298 | 56.9160539 |
| O | 62.0829242 | 46.3581328 | 51.2512849 | H | 57.6038181 | 45.3425978 | 57.0942857 |
| H | 60.8577311 | 44.7634675 | 48.2819143 | H | 59.2132602 | 45.3531423 | 57.8986946 |
| H | 61.7520513 | 45.8458397 | 47.1951096 | O | 59.2008577 | 46.3893348 | 54.0818473 |
| H | 61.8349845 | 47.6154186 | 48.9504885 | C | 59.1559355 | 39.6869468 | 52.4053335 |
| H | 60.1629662 | 47.0700978 | 48.6982672 | N | 58.7018269 | 40.8480545 | 53.2546556 |
| N | 60.6868954 | 50.3669657 | 53.5608752 | H | 59.5295520 | 40.1092270 | 51.4604196 |
| C | 61.8568141 | 50.3619844 | 52.8208130 | H | 58.2598002 | 39.0873211 | 52.1858719 |
| C | 62.5149904 | 51.6369537 | 52.4013168 | H | 59.5668733 | 41.2897131 | 53.6361473 |
| N | 61.1547596 | 48.2563317 | 53.2047871 | C | 58.0234319 | 41.8738793 | 52.4371052 |
| C | 62.1347752 | 49.0297290 | 52.6172796 | H | 58.2996997 | 41.8236447 | 51.3756357 |
| C | 60.2907218 | 49.0933086 | 53.7592721 | C | 57.4773235 | 43.0807292 | 53.0978280 |
| H | 62.3408424 | 51.8583516 | 51.3351934 | H | 59.8951769 | 44.3155024 | 52.1641650 |
| H | 62.0666166 | 52.4684138 | 52.9622271 | C | 57.8600461 | 40.4204959 | 54.4255107 |
| H | 62.9729261 | 48.5765746 | 52.0945689 | H | 57.7315102 | 41.2576635 | 55.1261405 |
| H | 59.3781133 | 48.8233048 | 54.2818593 | H | 56.8905983 | 40.0907849 | 54.0290660 |
| H | 60.1953252 | 51.1831800 | 53.9185553 | H | 58.3456999 | 39.5906291 | 54.9607633 |
| Fe | 61.0930579 | 46.0630538 | 53.1268367 | H | 57.0331857 | 43.7410715 | 52.3417472 |
| O | 60.6921404 | 44.2380466 | 52.7094180 | H | 58.2675004 | 43.6707440 | 53.6058440 |
| C | 59.6891994 | 46.3382130 | 55.2620405 | H | 56.7060109 | 42.8528899 | 53.8508716 |
| O | 60.8650689 | 45.8928526 | 55.3830151 | H | 67.3040081 | 46.5009863 | 53.0964157 |
| C | 58.9354870 | 46.9351699 | 56.4234613 | H | 62.5770031 | 45.1132407 | 48.5442910 |
| C | 58.3998685 | 45.9817867 | 57.5064506 | H | 63.5937155 | 51.6741534 | 52.5530908 |
| C | 57.8365968 | 46.8838778 | 58.6330705 | H | 59.9469349 | 39.1036744 | 52.8766936 |

MEL2-IM1N344A-eth-Cβ

| | | | |
|---|---|---|---|
| N | 64.6524943 | 46.7008965 | 55.0545524 |
| C | 65.0485996 | 45.9760559 | 53.9465304 |
| C | 66.4661732 | 45.6447430 | 53.6011169 |
| N | 62.8092297 | 46.2230669 | 53.9533695 |
| C | 63.8822767 | 45.6830673 | 53.2768311 |
| C | 63.3116687 | 46.8377195 | 55.0180952 |
| H | 67.0252670 | 45.2950876 | 54.4831792 |
| H | 66.4751912 | 44.8133293 | 52.8798956 |
| H | 63.7495226 | 45.1513710 | 52.3388673 |
| H | 62.7434608 | 47.3912053 | 55.7632563 |
| H | 65.2614814 | 47.0705680 | 55.7971574 |
| C | 61.5675444 | 45.5599527 | 48.1826675 |
| C | 61.0626394 | 46.7800158 | 48.9672474 |
| C | 60.8015875 | 46.5613628 | 50.4508639 |
| O | 59.6778778 | 46.7410390 | 50.9580422 |
| O | 61.7960740 | 46.2340917 | 51.1947822 |
| H | 60.7995746 | 44.7694775 | 48.2115127 |
| H | 61.7225562 | 45.8315259 | 47.1311233 |
| H | 61.7706061 | 47.6235938 | 48.8837328 |
| H | 60.1158685 | 47.1146353 | 48.5174076 |
| N | 60.8404108 | 50.4154331 | 53.6412862 |
| C | 61.8476935 | 50.4281315 | 52.6917433 |
| C | 62.5394138 | 51.6979526 | 52.3187838 |
| N | 61.0752934 | 48.3422426 | 52.9976655 |
| C | 61.9818975 | 49.1148919 | 52.3029631 |
| C | 60.3956247 | 49.1550189 | 53.7961938 |
| H | 62.4045742 | 51.9382064 | 51.2525009 |
| H | 62.0858969 | 52.5266968 | 52.8786008 |
| H | 62.6851484 | 48.6722510 | 51.6021593 |
| H | 59.6011346 | 48.8544659 | 54.4722399 |
| H | 60.4646068 | 51.2220577 | 54.1353215 |
| Fe | 60.8450295 | 46.3228990 | 52.9592318 |
| O | 60.5635699 | 44.5119725 | 52.9423988 |
| C | 59.1638056 | 45.8481985 | 55.3412237 |
| O | 59.1881332 | 44.6215879 | 55.3097625 |
| C | 58.7081805 | 46.6785137 | 56.5277611 |
| C | 58.0846223 | 45.9676491 | 57.7236785 |
| C | 57.7320220 | 47.0424176 | 58.7752588 |
| O | 58.6600530 | 47.4200970 | 59.5333805 |
| O | 56.5638428 | 47.5107713 | 58.7421623 |
| H | 58.0195575 | 47.4484054 | 56.1383009 |
| H | 59.5930903 | 47.2508344 | 56.8621083 |
| H | 57.1768458 | 45.4274853 | 57.4184428 |
| H | 58.8010487 | 45.2489765 | 58.1473470 |
| O | 59.5534474 | 46.6149968 | 54.3422395 |
| C | 59.2114997 | 39.5235531 | 52.3056898 |
| N | 58.7572400 | 40.7559367 | 53.0294388 |
| H | 59.5281851 | 39.8438142 | 51.3014462 |
| H | 58.3317905 | 38.8721123 | 52.1995886 |
| H | 59.6028572 | 41.3274081 | 53.2229838 |
| C | 57.8294135 | 41.5752607 | 52.1128356 |
| H | 56.9457395 | 40.9291568 | 51.9884266 |

| | | | |
|---|---|---|---|
| C | 57.5169186 | 42.9276073 | 52.6337748 |
| H | 58.3637210 | 41.6266041 | 51.1459842 |
| C | 58.0942627 | 40.4799928 | 54.3426467 |
| H | 57.9534328 | 41.4201044 | 54.8949940 |
| H | 57.1233609 | 40.0078543 | 54.1409641 |
| H | 58.7062328 | 39.7943754 | 54.9438727 |
| H | 58.2736023 | 43.7160946 | 52.5852541 |
| H | 59.9818030 | 44.3900708 | 53.7314199 |
| H | 56.5656391 | 43.1453578 | 53.1236377 |
| H | 67.0355540 | 46.4816512 | 53.1968183 |
| H | 62.5119296 | 45.1239650 | 48.5084260 |
| H | 63.6126510 | 51.7074296 | 52.5088870 |
| H | 60.0394679 | 39.0020968 | 52.7859179 |

MEL-OH-RCwat

| | | | |
|---|---|---|---|
| N | 57.8849632 | 49.7784559 | 49.5411450 |
| C | 57.3696678 | 48.6194810 | 50.0917982 |
| C | 56.0290977 | 48.5324843 | 50.7503447 |
| N | 59.4576254 | 48.2702452 | 49.3513615 |
| C | 58.3668848 | 47.6799523 | 49.9501303 |
| C | 59.1325129 | 49.5348509 | 49.1154208 |
| H | 55.2523105 | 49.0582387 | 50.1751693 |
| H | 55.7191300 | 47.4772502 | 50.8021705 |
| H | 58.3646637 | 46.6332303 | 50.2433209 |
| H | 59.7693266 | 50.2684349 | 48.6319347 |
| H | 57.4304222 | 50.7000591 | 49.4813015 |
| C | 61.5632520 | 43.8322272 | 52.6086113 |
| C | 62.3812343 | 45.0984529 | 52.3119776 |
| C | 62.3904641 | 45.4384738 | 50.8126432 |
| O | 62.7724532 | 44.5996866 | 49.9920015 |
| O | 61.9555274 | 46.6204422 | 50.5383288 |
| H | 61.9821180 | 43.0031199 | 52.0168405 |
| H | 61.6430580 | 43.5420221 | 53.6673728 |
| H | 62.0310012 | 45.9682942 | 52.8869066 |
| H | 63.4261076 | 44.9041177 | 52.6106045 |
| N | 63.0094532 | 51.0896066 | 50.4293392 |
| C | 62.4986940 | 50.5341369 | 51.5940216 |
| C | 62.6053297 | 51.1901742 | 52.9375913 |
| N | 62.1043306 | 49.1864911 | 49.8341738 |
| C | 61.9348658 | 49.3492656 | 51.1932643 |
| C | 62.7604234 | 50.2529021 | 49.3968234 |
| H | 63.0439514 | 50.5069319 | 53.6816356 |
| H | 63.2851511 | 52.0506252 | 52.8688655 |
| H | 61.4238576 | 48.6033271 | 51.7923739 |
| H | 63.0751769 | 50.4481870 | 48.3723666 |
| H | 63.5252981 | 51.9644010 | 50.3528016 |
| Fe | 61.3258825 | 47.4744246 | 48.8854604 |
| O | 60.6831041 | 46.1188378 | 48.2637181 |
| C | 62.2180231 | 48.2061836 | 46.6256953 |
| O | 61.0951862 | 48.6511677 | 46.9875998 |
| C | 62.7379380 | 48.3340691 | 45.2173672 |
| C | 62.2216989 | 49.5072622 | 44.3857910 |
| C | 63.0882352 | 50.7869892 | 44.4602678 |

| | | | |
|---|---|---|---|
| O | 62.5583305 | 51.8577392 | 44.0974766 |
| O | 64.2897181 | 50.6478121 | 44.8166221 |
| H | 62.4874664 | 47.3627612 | 44.7531069 |
| H | 63.8341627 | 48.3751967 | 45.2503149 |
| H | 62.2411237 | 49.2190083 | 43.3231385 |
| H | 61.1729697 | 49.7464066 | 44.6117160 |
| O | 62.8850280 | 47.5441501 | 47.5014249 |
| C | 57.5286455 | 40.5040187 | 44.1148279 |
| C | 58.9627959 | 40.9016080 | 43.7426358 |
| C | 59.7023535 | 41.5302848 | 44.9344209 |
| C | 61.1175741 | 41.9651753 | 44.5645608 |
| N | 61.6931566 | 43.0026575 | 45.4997529 |
| H | 57.0725603 | 41.3190684 | 44.7020352 |
| H | 57.5247571 | 39.6127117 | 44.7660394 |
| H | 59.5211487 | 40.0247939 | 43.3680459 |
| H | 58.9703550 | 41.6400300 | 42.9227694 |
| H | 59.1130650 | 42.3970553 | 45.2758562 |
| H | 59.7463700 | 40.8237818 | 45.7817928 |
| H | 61.8127586 | 41.1109021 | 44.5795178 |
| H | 61.1434883 | 42.4167259 | 43.5591156 |
| H | 61.4313730 | 42.7783302 | 46.4899230 |
| C | 63.1951271 | 42.9481801 | 45.4414508 |
| H | 63.4898471 | 43.0352570 | 44.3842577 |
| C | 63.9074487 | 44.0182556 | 46.2470826 |
| H | 63.4478998 | 41.9453111 | 45.8138037 |
| C | 61.1304314 | 44.3608781 | 45.2374942 |
| H | 60.0358776 | 44.2928527 | 45.2400281 |
| H | 61.4500214 | 45.0444566 | 46.0293484 |
| H | 61.4854424 | 44.7194712 | 44.2601543 |
| H | 63.3929329 | 44.1623438 | 47.2186025 |
| H | 64.4984546 | 45.8568316 | 45.9938535 |
| H | 64.9171208 | 43.6353034 | 46.4881471 |
| O | 63.9735118 | 45.1995987 | 45.4901326 |
| O | 60.8340331 | 43.3188980 | 48.2327262 |
| H | 59.8577564 | 43.2195408 | 48.1467668 |
| H | 60.9597540 | 44.2832762 | 48.3710093 |
| H | 56.0114709 | 48.9711133 | 51.7480226 |
| H | 60.5036594 | 43.9508079 | 52.3821842 |
| H | 61.6644626 | 51.5386505 | 53.3635027 |
| H | 56.8506897 | 40.3154104 | 43.2824397 |

MEL-OH-TS1-Owat

| | | | |
|---|---|---|---|
| N | 57.8035962 | 49.7758494 | 49.4697353 |
| C | 57.3207585 | 48.6262971 | 50.0695897 |
| C | 55.9952602 | 48.5325332 | 50.7580803 |
| N | 59.4133778 | 48.3031802 | 49.3361143 |
| C | 58.3431434 | 47.7087922 | 49.9651223 |
| C | 59.0582373 | 49.5452946 | 49.0493112 |
| H | 55.1990809 | 49.0481195 | 50.2009471 |
| H | 55.6927476 | 47.4758118 | 50.8241370 |
| H | 58.3949377 | 46.6891084 | 50.3375222 |
| H | 59.6810530 | 50.2732271 | 48.5401636 |
| H | 57.3367203 | 50.6901398 | 49.4004230 |

| | | | |
|---|---|---|---|
| C | 61.5719850 | 43.9110194 | 52.4033372 |
| C | 62.3072557 | 45.2675397 | 52.3520820 |
| C | 62.0207396 | 46.0902367 | 51.1054262 |
| O | 62.8323289 | 46.1911096 | 50.1548728 |
| O | 60.9048345 | 46.6969626 | 51.0181813 |
| H | 61.9903901 | 43.2533893 | 51.6244898 |
| H | 61.7623838 | 43.4184124 | 53.3687954 |
| H | 62.0392996 | 45.8909751 | 53.2210119 |
| H | 63.3889847 | 45.0729814 | 52.3939649 |
| N | 63.2143935 | 50.8901762 | 50.5980677 |
| C | 62.5045990 | 50.5151506 | 51.7303554 |
| C | 62.5940374 | 51.2566707 | 53.0277098 |
| N | 62.1112600 | 49.0903717 | 50.0404434 |
| C | 61.8095639 | 49.3941857 | 51.3534340 |
| C | 62.9664033 | 50.0099327 | 49.6078695 |
| H | 63.0621537 | 50.6417852 | 53.8133843 |
| H | 63.2426075 | 52.1321761 | 52.8940199 |
| H | 61.1104747 | 48.7921702 | 51.9250600 |
| H | 63.4241403 | 50.0571468 | 48.6221208 |
| H | 63.8370316 | 51.6924284 | 50.5119769 |
| Fe | 61.3332598 | 47.4510968 | 49.0549484 |
| O | 60.6131926 | 46.0291614 | 48.3256368 |
| C | 62.1870039 | 48.2558688 | 46.7506843 |
| O | 61.0622596 | 48.7211341 | 47.0586663 |
| C | 62.7649170 | 48.3255972 | 45.3559167 |
| C | 62.2380715 | 49.4451552 | 44.4536855 |
| C | 63.0805567 | 50.7378437 | 44.4580124 |
| O | 62.5295063 | 51.7917718 | 44.0708579 |
| O | 64.2911263 | 50.6388832 | 44.7861549 |
| H | 62.5447872 | 47.3300468 | 44.9264045 |
| H | 63.8588027 | 48.3899666 | 45.4288839 |
| H | 62.2692971 | 49.0987551 | 43.4087508 |
| H | 61.1851691 | 49.6788535 | 44.6645076 |
| O | 62.8291308 | 47.6194475 | 47.6612328 |
| C | 57.5096705 | 40.4339072 | 43.9425287 |
| C | 58.8991172 | 40.8583694 | 43.4483177 |
| C | 59.7532477 | 41.3962861 | 44.5976808 |
| C | 61.0778895 | 41.9993749 | 44.1446878 |
| N | 61.6952636 | 42.8610752 | 45.2153012 |
| H | 57.0950209 | 41.2198716 | 44.5952041 |
| H | 57.5707570 | 39.5197180 | 44.5586484 |
| H | 59.4097122 | 40.0188614 | 42.9477369 |
| H | 58.8171007 | 41.6630026 | 42.6986054 |
| H | 59.1597795 | 42.1745633 | 45.0942136 |
| H | 59.9369822 | 40.6114258 | 45.3531585 |
| H | 61.8262470 | 41.2218696 | 43.9195230 |
| H | 60.9523652 | 42.6338860 | 43.2509627 |
| H | 61.8611124 | 42.2430882 | 46.0342657 |
| C | 63.0403637 | 43.4108829 | 44.8145932 |
| H | 62.8468504 | 44.3354257 | 44.2490621 |
| C | 63.9242400 | 43.6297690 | 46.0562127 |
| H | 63.5123386 | 42.6806964 | 44.1405957 |
| C | 60.7969779 | 43.9854225 | 45.6539125 |
| H | 60.0256861 | 43.5879510 | 46.3269178 |

| | | | |
|---|---|---|---|
| H | 61.4208924 | 44.7103152 | 46.1851689 |
| H | 60.3384649 | 44.4369308 | 44.7627269 |
| H | 64.2057311 | 42.6086052 | 46.4085575 |
| H | 62.4249098 | 43.9233119 | 48.0002826 |
| H | 64.8714816 | 44.0715794 | 45.6679603 |
| O | 63.3779950 | 44.3644954 | 47.0673229 |
| O | 61.6650575 | 43.7519246 | 48.7380098 |
| H | 60.9542337 | 43.2242360 | 48.3044188 |
| H | 61.0755887 | 45.1276374 | 48.5579388 |
| H | 55.9999042 | 48.9752723 | 51.7540861 |
| H | 60.4920618 | 43.9653981 | 52.2659474 |
| H | 61.6405889 | 51.5949751 | 53.4333614 |
| H | 56.7785877 | 40.2650220 | 43.1519195 |

MEL-OH-IM1-Owat

| | | | |
|---|---|---|---|
| N | 57.8201087 | 49.8074856 | 49.4184718 |
| C | 57.3402518 | 48.6759045 | 50.0487274 |
| C | 56.0168214 | 48.5838156 | 50.7416575 |
| N | 59.4264276 | 48.3204518 | 49.2894270 |
| C | 58.3617694 | 47.7550373 | 49.9553396 |
| C | 59.0707268 | 49.5546491 | 48.9843324 |
| H | 55.2232431 | 49.1085068 | 50.1896529 |
| H | 55.7092401 | 47.5276507 | 50.7975236 |
| H | 58.4222851 | 46.7592481 | 50.3897010 |
| H | 59.6942963 | 50.2678288 | 48.4544839 |
| H | 57.3595623 | 50.7246161 | 49.3467588 |
| C | 61.5639784 | 43.8507486 | 52.3907347 |
| C | 62.3124543 | 45.1914451 | 52.2305647 |
| C | 61.9956555 | 45.9634107 | 50.9592395 |
| O | 62.7841553 | 46.0201812 | 49.9797868 |
| O | 60.8919384 | 46.5917214 | 50.8856592 |
| H | 61.9614453 | 43.1331766 | 51.6562007 |
| H | 61.7607814 | 43.4279246 | 53.3875569 |
| H | 62.0813381 | 45.8635848 | 53.0740293 |
| H | 63.3937677 | 44.9884300 | 52.2505668 |
| N | 63.1108655 | 50.9789801 | 50.5284998 |
| C | 62.5523826 | 50.4817250 | 51.6962795 |
| C | 62.6303750 | 51.2028004 | 53.0068906 |
| N | 62.2001943 | 49.0596667 | 49.9824816 |
| C | 61.9845513 | 49.2867893 | 51.3272718 |
| C | 62.8836352 | 50.1004050 | 49.5265818 |
| H | 63.0740481 | 50.5659663 | 53.7886003 |
| H | 63.2963839 | 52.0702652 | 52.9029254 |
| H | 61.4079670 | 48.5906001 | 51.9289262 |
| H | 63.2180165 | 50.2574292 | 48.5022963 |
| H | 63.6316665 | 51.8482786 | 50.4304099 |
| Fe | 61.3490307 | 47.3526816 | 48.9137816 |
| O | 60.3528177 | 45.9340596 | 48.0784668 |
| C | 62.2561005 | 48.0914492 | 46.5925057 |
| O | 61.3327733 | 48.7673509 | 47.1238179 |
| C | 62.7257849 | 48.2980917 | 45.1725806 |
| C | 62.1752830 | 49.5007753 | 44.4073626 |
| C | 63.0621211 | 50.7703506 | 44.4453991 |

| | | | |
|---|---|---|---|
| O | 62.5293435 | 51.8474764 | 44.1077173 |
| O | 64.2749112 | 50.6122611 | 44.7442013 |
| H | 62.4824303 | 47.3501861 | 44.6591025 |
| H | 63.8255434 | 48.3646287 | 45.1886393 |
| H | 62.1152380 | 49.2401080 | 43.3394756 |
| H | 61.1499022 | 49.7526349 | 44.7113018 |
| O | 62.7617498 | 47.1408285 | 47.2914086 |
| C | 57.5351095 | 40.4094119 | 44.0014477 |
| C | 58.9565481 | 40.7642883 | 43.5424531 |
| C | 59.7725308 | 41.3624254 | 44.6947017 |
| C | 61.0775409 | 41.9988200 | 44.2289242 |
| N | 61.6792722 | 42.9513973 | 45.2435993 |
| H | 57.1343943 | 41.2329651 | 44.6164090 |
| H | 57.5387216 | 39.5147583 | 44.6476082 |
| H | 59.4640864 | 39.8798462 | 43.1226912 |
| H | 58.9277271 | 41.5157951 | 42.7356005 |
| H | 59.1390342 | 42.1321061 | 45.1535621 |
| H | 59.9707312 | 40.6098552 | 45.4773219 |
| H | 61.8592680 | 41.2454054 | 44.0347183 |
| H | 60.9189585 | 42.5860909 | 43.3082314 |
| H | 62.0264662 | 42.3794142 | 46.0439855 |
| C | 62.8601199 | 43.6583624 | 44.6422977 |
| H | 62.4783168 | 44.3874985 | 43.9064324 |
| C | 63.7289197 | 44.3190608 | 45.7139511 |
| H | 63.4501065 | 42.9004569 | 44.1064795 |
| C | 60.6893621 | 43.9373258 | 45.7801765 |
| H | 59.9583281 | 43.4186115 | 46.4127345 |
| H | 61.1948275 | 44.6852284 | 46.3954213 |
| H | 60.1849228 | 44.4260711 | 44.9333805 |
| H | 64.6155331 | 44.8060691 | 45.2502582 |
| H | 61.0931168 | 44.4238795 | 48.6977836 |
| H | 63.2449724 | 45.2086514 | 46.2151447 |
| O | 64.0889040 | 43.4819905 | 46.7093107 |
| O | 61.1539153 | 43.4746556 | 48.9571655 |
| H | 60.3921424 | 43.0866925 | 48.4673544 |
| H | 59.4800144 | 45.7385078 | 48.4542624 |
| H | 56.0233161 | 49.0150681 | 51.7426801 |
| H | 60.4833666 | 43.9104266 | 52.2611818 |
| H | 61.6792715 | 51.5532349 | 53.4077210 |
| H | 56.8159480 | 40.2487127 | 43.1982979 |

MEL-OH-TS2-Owat

| | | | |
|---|---|---|---|
| N | 57.6804084 | 49.7463623 | 49.3745243 |
| C | 57.2357952 | 48.6320049 | 50.0597761 |
| C | 55.9333318 | 48.5494643 | 50.7918057 |
| N | 59.3036434 | 48.2788679 | 49.2502315 |
| C | 58.2668594 | 47.7225861 | 49.9693979 |
| C | 58.9186179 | 49.4976224 | 48.9099528 |
| H | 55.1253842 | 49.0666089 | 50.2540915 |
| H | 55.6256535 | 47.4948608 | 50.8696576 |
| H | 58.3541068 | 46.7455149 | 50.4385191 |
| H | 59.5144375 | 50.1971819 | 48.3335920 |
| H | 57.2139956 | 50.6606594 | 49.2971092 |

| | | | |
|---|---|---|---|
| C | 61.5500249 | 43.8930085 | 52.3238575 |
| C | 62.2572869 | 45.2615674 | 52.2260225 |
| C | 61.9274941 | 46.0552405 | 50.9742571 |
| O | 62.7251547 | 46.1671169 | 50.0101122 |
| O | 60.7969019 | 46.6379028 | 50.8896443 |
| H | 61.9631385 | 43.2236847 | 51.5504536 |
| H | 61.7742096 | 43.4196152 | 53.2913541 |
| H | 61.9953284 | 45.8968832 | 53.0886700 |
| H | 63.3439939 | 45.0932154 | 52.2498433 |
| N | 63.1401104 | 50.9758012 | 50.5943474 |
| C | 62.5005879 | 50.5259533 | 51.7400151 |
| C | 62.5959172 | 51.2364566 | 53.0544492 |
| N | 62.0839722 | 49.1504429 | 50.0086500 |
| C | 61.8393342 | 49.3889735 | 51.3464734 |
| C | 62.8761648 | 50.1255005 | 49.5807730 |
| H | 63.0433274 | 50.5954112 | 53.8308488 |
| H | 63.2625312 | 52.1021922 | 52.9471750 |
| H | 61.1884434 | 48.7392401 | 51.9244872 |
| H | 63.2737920 | 50.2463767 | 48.5743996 |
| H | 63.7333305 | 51.8004459 | 50.5167898 |
| Fe | 61.2866754 | 47.4740255 | 48.9594206 |
| O | 60.3755431 | 45.8567318 | 48.1013987 |
| C | 62.1548214 | 48.1996527 | 46.6578396 |
| O | 61.1080063 | 48.7647567 | 47.0906040 |
| C | 62.6739510 | 48.3524034 | 45.2536962 |
| C | 62.1460046 | 49.5231425 | 44.4247920 |
| C | 63.0398433 | 50.7891122 | 44.4426671 |
| O | 62.5112372 | 51.8657964 | 44.0961269 |
| O | 64.2518422 | 50.6260182 | 44.7385085 |
| H | 62.4424868 | 47.3813896 | 44.7762517 |
| H | 63.7724775 | 48.4105667 | 45.2999495 |
| H | 62.1183245 | 49.2199201 | 43.3667930 |
| H | 61.1121985 | 49.7888230 | 44.6869511 |
| O | 62.7343370 | 47.3915083 | 47.4667422 |
| C | 57.5596456 | 40.4185862 | 43.8829662 |
| C | 58.8709319 | 41.0313064 | 43.3761957 |
| C | 60.0230421 | 40.8356120 | 44.3662859 |
| C | 61.2699051 | 41.6872334 | 44.0746587 |
| N | 61.5341542 | 42.6952864 | 45.1570601 |
| H | 57.1259246 | 41.0599236 | 44.6675094 |
| H | 57.7358812 | 39.4330614 | 44.3511193 |
| H | 59.1815039 | 40.5968454 | 42.4101269 |
| H | 58.7289798 | 42.1093318 | 43.1885416 |
| H | 59.6731234 | 41.0358274 | 45.3916034 |
| H | 60.3070238 | 39.7713768 | 44.3719304 |
| H | 62.1785210 | 41.0662022 | 44.0341001 |
| H | 61.1749216 | 42.2423096 | 43.1252955 |
| H | 61.5697925 | 42.1599846 | 46.0458211 |
| C | 62.9013770 | 43.3414447 | 45.0253165 |
| H | 62.8269509 | 44.0347792 | 44.1720075 |
| C | 63.3929877 | 43.9867923 | 46.3291207 |
| H | 63.6011555 | 42.5304245 | 44.7854319 |
| C | 60.4646619 | 43.7295063 | 45.2302614 |
| H | 59.4917665 | 43.2688659 | 45.4453505 |

| | | | |
|---|---|---|---|
| H | 60.6941932 | 44.4402809 | 46.0283082 |
| H | 60.4375041 | 44.2534156 | 44.2655212 |
| H | 63.3136519 | 45.0995241 | 46.3365808 |
| H | 61.0280297 | 44.8466809 | 48.2341825 |
| H | 62.4241248 | 43.8200422 | 47.4072120 |
| O | 64.4691557 | 43.4571842 | 46.8288320 |
| O | 61.6164325 | 43.7932850 | 48.3146691 |
| H | 60.9080241 | 43.1433102 | 48.0676415 |
| H | 59.4890120 | 45.6593564 | 48.4480348 |
| H | 55.9714701 | 48.9947591 | 51.7859511 |
| H | 60.4659379 | 43.9294163 | 52.2166340 |
| H | 61.6454078 | 51.5866707 | 53.4568780 |
| H | 56.8020828 | 40.2793698 | 43.1117435 |

MEL-OH-PC-Owat

| | | | |
|---|---|---|---|
| N | 57.7508573 | 49.8502109 | 49.6196142 |
| C | 57.2898632 | 48.6910348 | 50.2096482 |
| C | 55.9481623 | 48.5658692 | 50.8641322 |
| N | 59.4123092 | 48.4207849 | 49.4985188 |
| C | 58.3501098 | 47.8070650 | 50.1240650 |
| C | 59.0218285 | 49.6496690 | 49.2216737 |
| H | 55.1593306 | 49.0661231 | 50.2826939 |
| H | 55.6640528 | 47.5032026 | 50.9251452 |
| H | 58.4169255 | 46.7842294 | 50.4940666 |
| H | 59.6267213 | 50.4050616 | 48.7266875 |
| H | 57.2593836 | 50.7478246 | 49.5270343 |
| C | 61.4154312 | 43.7878978 | 52.6381708 |
| C | 62.2503051 | 45.0000818 | 52.1859473 |
| C | 62.1376722 | 45.2156419 | 50.6686130 |
| O | 62.3813949 | 44.2419854 | 49.9145868 |
| O | 61.7058199 | 46.3430609 | 50.2877067 |
| H | 61.8047651 | 42.9004948 | 52.1151260 |
| H | 61.5294200 | 43.5966334 | 53.7168180 |
| H | 61.9630626 | 45.9235954 | 52.7100280 |
| H | 63.3070378 | 44.7913094 | 52.4281298 |
| N | 63.0064183 | 51.1480998 | 50.5181206 |
| C | 62.5712459 | 50.4968891 | 51.6626402 |
| C | 62.6245075 | 51.1230222 | 53.0234133 |
| N | 62.2916608 | 49.1805064 | 49.8493673 |
| C | 62.1302975 | 49.2736498 | 51.2150692 |
| C | 62.8251925 | 50.3248285 | 49.4556899 |
| H | 63.0089692 | 50.4108573 | 53.7698500 |
| H | 63.3331985 | 51.9634203 | 53.0079313 |
| H | 61.6988100 | 48.4546937 | 51.7842225 |
| H | 63.0894005 | 50.5911811 | 48.4326022 |
| H | 63.4331781 | 52.0705368 | 50.4765157 |
| Fe | 61.2581970 | 47.5914614 | 48.7310585 |
| O | 59.6917154 | 46.2804953 | 47.5916835 |
| C | 62.2123697 | 48.0411263 | 46.3801504 |
| O | 61.3304038 | 48.7904822 | 46.8935755 |
| C | 62.7389513 | 48.2897898 | 44.9791250 |
| C | 62.2144446 | 49.5185939 | 44.2380632 |
| C | 63.0754631 | 50.7967498 | 44.3677249 |

| | | | |
|---|---|---|---|
| O | 62.5275766 | 51.8833139 | 44.0853924 |
| O | 64.2872190 | 50.6461697 | 44.6746073 |
| H | 62.5301526 | 47.3604727 | 44.4208749 |
| H | 63.8363350 | 48.3666065 | 45.0427542 |
| H | 62.2044124 | 49.3046053 | 43.1584975 |
| H | 61.1766166 | 49.7589668 | 44.5066019 |
| O | 62.6338891 | 47.0296974 | 47.0349467 |
| C | 57.6487667 | 40.5175058 | 43.9604949 |
| C | 59.0377690 | 40.9204264 | 43.4427342 |
| C | 59.8784688 | 41.5471639 | 44.5614189 |
| C | 61.1811663 | 42.1610308 | 44.0643868 |
| N | 61.8682957 | 43.0313723 | 45.1013643 |
| H | 57.2434941 | 41.3341071 | 44.5832776 |
| H | 57.7121764 | 39.6312824 | 44.6148666 |
| H | 59.5545319 | 40.0502600 | 43.0055746 |
| H | 58.9503524 | 41.6676367 | 42.6373273 |
| H | 59.2597374 | 42.3345845 | 45.0121113 |
| H | 60.0847980 | 40.8130534 | 45.3588322 |
| H | 61.9236847 | 41.3895564 | 43.8000108 |
| H | 61.0072158 | 42.7975107 | 43.1801995 |
| H | 62.2449504 | 42.4146790 | 45.8540388 |
| C | 63.0113861 | 43.7711708 | 44.4884630 |
| H | 62.6108284 | 44.6042635 | 43.8842278 |
| C | 63.9847123 | 44.2284710 | 45.5570040 |
| H | 63.5563441 | 43.1039101 | 43.7969327 |
| C | 60.9469176 | 44.0107433 | 45.7784818 |
| H | 60.2712517 | 43.4546516 | 46.4363131 |
| H | 61.5444494 | 44.7180810 | 46.3657565 |
| H | 60.3758081 | 44.5531302 | 45.0096390 |
| H | 64.6255051 | 45.1002903 | 45.3076744 |
| H | 59.8397342 | 45.4073093 | 48.0629048 |
| H | 60.9921475 | 44.0537759 | 49.1088956 |
| O | 64.0745930 | 43.6413331 | 46.6089151 |
| O | 60.0066523 | 44.0992301 | 48.9464742 |
| H | 59.7413444 | 43.3367773 | 48.3760470 |
| H | 58.7259442 | 46.4122347 | 47.7426580 |
| H | 55.9183655 | 49.0106604 | 51.8587879 |
| H | 60.3496651 | 43.8948351 | 52.4362448 |
| H | 61.6798485 | 51.4963702 | 53.4187585 |
| H | 56.9044075 | 40.3211730 | 43.1888408 |

MEL-OH-TS1-Cβwat

| | | | |
|---|---|---|---|
| N | 57.6587500 | 49.6834379 | 49.3755377 |
| C | 57.2429235 | 48.5696553 | 50.0790223 |
| C | 55.9456548 | 48.4874770 | 50.8180456 |
| N | 59.3037141 | 48.2447980 | 49.2541461 |
| C | 58.2871846 | 47.6733415 | 49.9860653 |
| C | 58.8970240 | 49.4490540 | 48.9020908 |
| H | 55.1335555 | 48.9914224 | 50.2742104 |
| H | 55.6435290 | 47.4331732 | 50.9158439 |
| H | 58.4063722 | 46.6946617 | 50.4466331 |
| H | 59.4745621 | 50.1477010 | 48.3062440 |
| H | 57.1801776 | 50.5900198 | 49.2924020 |

| | | | | | | | |
|---|---|---|---|---|---|---|---|
| C | 61.6292742 | 43.9437536 | 52.4011349 | O | 62.4943721 | 51.8198712 | 44.0299780 |
| C | 62.3543066 | 45.3037342 | 52.3144893 | O | 64.2264806 | 50.6260202 | 44.7600269 |
| C | 62.0619412 | 46.0809004 | 51.0296415 | H | 62.4323486 | 47.3442119 | 44.7520911 |
| O | 62.8595395 | 46.1055739 | 50.0550202 | H | 63.7602036 | 48.3802698 | 45.2635588 |
| O | 60.9633098 | 46.7035352 | 50.9577639 | H | 62.1789776 | 49.1742942 | 43.3003667 |
| H | 62.0465808 | 43.2744587 | 51.6290222 | H | 61.1041788 | 49.7248432 | 44.5748578 |
| H | 61.8299953 | 43.4603948 | 53.3698226 | O | 62.8426211 | 47.6417755 | 47.5215059 |
| H | 62.0692388 | 45.9520842 | 53.1589166 | C | 57.5008799 | 40.4685252 | 43.9829913 |
| H | 63.4377281 | 45.1216198 | 52.3922912 | C | 58.9073012 | 40.8591261 | 43.5075985 |
| N | 63.1419037 | 50.9783704 | 50.5322370 | C | 59.7514206 | 41.3752356 | 44.6734440 |
| C | 62.4841432 | 50.5474705 | 51.6748886 | C | 61.1292470 | 41.8959792 | 44.2784228 |
| C | 62.5947955 | 51.2443593 | 52.9947889 | N | 61.6617107 | 42.8475004 | 45.3316012 |
| N | 62.0717872 | 49.1524643 | 49.9567275 | H | 57.0904040 | 41.2684272 | 44.6226388 |
| C | 61.8128306 | 49.4160830 | 51.2847020 | H | 57.5338095 | 39.5596740 | 44.6092552 |
| C | 62.8774216 | 50.1112214 | 49.5282815 | H | 59.4058799 | 40.0046347 | 43.0200421 |
| H | 63.0600510 | 50.5983913 | 53.7568072 | H | 58.8567459 | 41.6611333 | 42.7523967 |
| H | 63.2536882 | 52.1145523 | 52.8835391 | H | 59.1847860 | 42.1949561 | 45.1380813 |
| H | 61.1546214 | 48.7764001 | 51.8641374 | H | 59.8624721 | 40.5928624 | 45.4449724 |
| H | 63.2872773 | 50.2054346 | 48.5246330 | H | 61.8699235 | 41.0844972 | 44.1919930 |
| H | 63.7348169 | 51.8014038 | 50.4457874 | H | 61.1004998 | 42.4558863 | 43.3286761 |
| Fe | 61.2489867 | 47.4237461 | 48.9115578 | H | 61.4012372 | 42.4388348 | 46.2464584 |
| O | 60.5209786 | 45.8890772 | 48.1959495 | C | 63.1673719 | 42.8921142 | 45.3119167 |
| C | 62.1268734 | 48.1999908 | 46.6160342 | H | 63.4709286 | 43.1761152 | 44.2938696 |
| O | 60.9607157 | 48.5501728 | 46.9408439 | C | 63.7682678 | 43.8654261 | 46.3136849 |
| C | 62.6655551 | 48.3215325 | 45.2121132 | H | 63.4829248 | 41.8611551 | 45.5257010 |
| C | 62.1528118 | 49.4783023 | 44.3578836 | C | 61.0191500 | 44.1971749 | 45.2257576 |
| C | 63.0235312 | 50.7536214 | 44.4095466 | H | 59.9323797 | 44.0614548 | 45.2246443 |

| | | | |
|---|---|---|---|
| H | 61.2917177 | 44.8156937 | 46.0851991 |
| H | 61.3465435 | 44.6658507 | 44.2861301 |
| H | 63.1502197 | 43.8395237 | 47.2651295 |
| H | 64.3821301 | 45.7500168 | 46.2534455 |
| H | 64.7682690 | 43.5120471 | 46.6245934 |
| O | 63.8166690 | 45.1303572 | 45.7350699 |
| O | 61.8681261 | 43.7190000 | 48.5399083 |
| H | 61.0894307 | 43.1787700 | 48.2177400 |
| H | 61.1755952 | 45.1498543 | 48.2652638 |
| H | 55.9878638 | 48.9497766 | 51.8042325 |
| H | 60.5481309 | 43.9925361 | 52.2714563 |
| H | 61.6475007 | 51.5798218 | 53.4168843 |
| H | 56.7786746 | 40.3011328 | 43.1839504 |

MEL-OH-TS2-Cβwat

| | | | |
|---|---|---|---|
| N | 57.6751635 | 49.6836069 | 49.3504967 |
| C | 57.2506381 | 48.5826940 | 50.0689328 |
| C | 55.9531276 | 48.5147565 | 50.8121696 |
| N | 59.2930674 | 48.2077265 | 49.1993746 |
| C | 58.2796808 | 47.6675229 | 49.9636789 |
| C | 58.8992237 | 49.4203734 | 48.8519794 |
| H | 55.1437515 | 49.0210121 | 50.2656444 |
| H | 55.6458764 | 47.4618189 | 50.9132130 |
| H | 58.3892980 | 46.6969908 | 50.4427963 |
| H | 59.4762469 | 50.1071863 | 48.2416120 |
| H | 57.2088372 | 50.5984310 | 49.2733072 |
| C | 61.5847994 | 43.9657124 | 52.4243644 |
| C | 62.3125075 | 45.3200634 | 52.2669744 |
| C | 62.0028410 | 46.0800625 | 50.9844939 |
| O | 62.8049763 | 46.1463784 | 50.0090179 |
| O | 60.8958861 | 46.6871588 | 50.8962720 |
| H | 61.9925602 | 43.2566553 | 51.6881331 |
| H | 61.7825307 | 43.5493386 | 53.4238943 |
| H | 62.0512193 | 45.9960362 | 53.0983357 |
| H | 63.3970939 | 45.1403236 | 52.3193216 |
| N | 63.1015795 | 51.0094535 | 50.5660463 |
| C | 62.4621659 | 50.5500997 | 51.7074070 |
| C | 62.5688074 | 51.2368365 | 53.0340733 |
| N | 62.0494560 | 49.1815739 | 49.9653721 |
| C | 61.8017245 | 49.4162314 | 51.3024262 |
| C | 62.8391490 | 50.1607538 | 49.5470655 |
| H | 63.0148452 | 50.5779531 | 53.7964514 |
| H | 63.2422738 | 52.0987430 | 52.9403210 |
| H | 61.1573395 | 48.7588167 | 51.8782741 |
| H | 63.2435394 | 50.2806132 | 48.5430623 |
| H | 63.6849933 | 51.8405136 | 50.4920410 |
| Fe | 61.3193990 | 47.4251654 | 48.8915516 |
| O | 60.7012445 | 45.8325872 | 48.0793080 |
| C | 62.1481229 | 48.2322263 | 46.6039832 |
| O | 60.9833980 | 48.5658711 | 46.9667308 |
| C | 62.6139991 | 48.3262309 | 45.1744399 |
| C | 62.1110784 | 49.5147764 | 44.3537624 |
| C | 63.0140611 | 50.7706450 | 44.4049685 |

| | | | |
|---|---|---|---|
| O | 62.5028004 | 51.8548419 | 44.0541896 |
| O | 64.2195637 | 50.6011957 | 44.7282017 |
| H | 62.2893738 | 47.3693702 | 44.7252291 |
| H | 63.7110679 | 48.3211519 | 45.1589224 |
| H | 62.0982452 | 49.2255317 | 43.2919912 |
| H | 61.0736377 | 49.7803573 | 44.6026575 |
| O | 62.9011920 | 47.7106556 | 47.4983538 |
| C | 57.4405816 | 40.4875473 | 43.9538154 |
| C | 58.8360741 | 40.8968710 | 43.4626843 |
| C | 59.6879692 | 41.4207164 | 44.6182826 |
| C | 61.0601835 | 41.9204542 | 44.1851099 |
| N | 61.7289499 | 42.7604612 | 45.2410852 |
| H | 57.0299098 | 41.2760883 | 44.6082028 |
| H | 57.4925779 | 39.5742675 | 44.5719655 |
| H | 59.3412890 | 40.0492812 | 42.9688800 |
| H | 58.7709617 | 41.6996146 | 42.7084958 |
| H | 59.1287230 | 42.2455509 | 45.0832297 |
| H | 59.8050288 | 40.6440589 | 45.3944732 |
| H | 61.7488389 | 41.0826875 | 43.9892759 |
| H | 60.9990164 | 42.5386757 | 43.2728829 |
| H | 61.6464047 | 42.2593865 | 46.1472221 |
| C | 63.2446894 | 42.8532110 | 44.9483791 |
| H | 63.3167789 | 43.1766993 | 43.8986791 |
| C | 64.0004641 | 43.7841627 | 45.7997883 |
| H | 63.5845784 | 41.8101872 | 45.0365845 |
| C | 61.1095709 | 44.1101429 | 45.3837769 |
| H | 60.0282587 | 43.9914870 | 45.5110479 |
| H | 61.5073595 | 44.6111979 | 46.2730375 |
| H | 61.3292710 | 44.6888662 | 44.4749783 |
| H | 61.3020653 | 44.4128089 | 48.8122310 |
| H | 64.5835138 | 45.6412477 | 45.9485903 |
| H | 64.2291978 | 43.5804844 | 46.8508286 |
| O | 64.0986053 | 45.0405052 | 45.3244698 |
| O | 61.4339392 | 43.4815459 | 49.1223126 |
| H | 60.8581259 | 42.9967446 | 48.4835367 |
| H | 59.7660423 | 45.7688597 | 47.8408732 |
| H | 55.9890131 | 48.9795895 | 51.7974156 |
| H | 60.5040798 | 44.0115557 | 52.2901605 |
| H | 61.6237574 | 51.5870912 | 53.4491266 |
| H | 56.7165084 | 40.3173146 | 43.1570684 |

MEL-OH-PC-Cβwat

| | | | |
|---|---|---|---|
| N | 57.6751635 | 49.6836069 | 49.3504967 |
| C | 57.2506381 | 48.5826940 | 50.0689328 |
| C | 55.9531276 | 48.5147565 | 50.8121696 |
| N | 59.2930674 | 48.2077265 | 49.1993746 |
| C | 58.2796808 | 47.6675229 | 49.9636789 |
| C | 58.8992237 | 49.4203734 | 48.8519794 |
| H | 55.1437515 | 49.0210121 | 50.2656444 |
| H | 55.6458764 | 47.4618189 | 50.9132130 |
| H | 58.3892980 | 46.6969908 | 50.4427963 |
| H | 59.4762469 | 50.1071863 | 48.2416120 |
| H | 57.2088372 | 50.5984310 | 49.2733072 |
| C | 61.5847994 | 43.9657124 | 52.4243644 |
| C | 62.3125075 | 45.3200634 | 52.2669744 |

| | | | | | | | |
|---|---|---|---|---|---|---|---|
| C | 62.0028410 | 46.0800625 | 50.9844939 | H | 62.2893738 | 47.3693702 | 44.7252291 |
| O | 62.8049763 | 46.1463784 | 50.0090179 | H | 63.7110679 | 48.3211519 | 45.1589224 |
| O | 60.8958861 | 46.6871588 | 50.8962720 | H | 62.0982452 | 49.2255317 | 43.2919912 |
| H | 61.9925602 | 43.2566553 | 51.6881331 | H | 61.0736377 | 49.7803573 | 44.6026575 |
| H | 61.7825307 | 43.5493386 | 53.4238943 | O | 62.9011920 | 47.7106556 | 47.4983538 |
| H | 62.0512193 | 45.9960362 | 53.0983357 | C | 57.4405816 | 40.4875473 | 43.9538154 |
| H | 63.3970939 | 45.1403236 | 52.3193216 | C | 58.8360741 | 40.8968710 | 43.4626843 |
| N | 63.1015795 | 51.0094535 | 50.5660463 | C | 59.6879692 | 41.4207164 | 44.6182826 |
| C | 62.4621659 | 50.5500997 | 51.7074070 | C | 61.0601835 | 41.9204542 | 44.1851099 |
| C | 62.5688074 | 51.2368365 | 53.0340733 | N | 61.7289499 | 42.7604612 | 45.2410852 |
| N | 62.0494560 | 49.1815739 | 49.9653721 | H | 57.0299098 | 41.2760883 | 44.6082028 |
| C | 61.8017245 | 49.4162314 | 51.3024262 | H | 57.4925779 | 39.5742675 | 44.5719655 |
| C | 62.8391490 | 50.1607538 | 49.5470655 | H | 59.3412890 | 40.0492812 | 42.9688800 |
| H | 63.0148452 | 50.5779531 | 53.7964514 | H | 58.7709617 | 41.6996146 | 42.7084958 |
| H | 63.2422738 | 52.0987430 | 52.9403210 | H | 59.1287230 | 42.2455509 | 45.0832297 |
| H | 61.1573395 | 48.7588167 | 51.8782741 | H | 59.8050288 | 40.6440589 | 45.3944732 |
| H | 63.2435394 | 50.2806132 | 48.5430623 | H | 61.7488389 | 41.0826875 | 43.9892759 |
| H | 63.6849933 | 51.8405136 | 50.4920410 | H | 60.9990164 | 42.5386757 | 43.2728829 |
| Fe | 61.3193990 | 47.4251654 | 48.8915516 | H | 61.6464047 | 42.2593865 | 46.1472221 |
| O | 60.7012445 | 45.8325872 | 48.0793080 | C | 63.2446894 | 42.8532110 | 44.9483791 |
| C | 62.1481229 | 48.2322263 | 46.6039832 | H | 63.3167789 | 43.1766993 | 43.8986791 |
| O | 60.9833980 | 48.5658711 | 46.9667308 | C | 64.0004641 | 43.7841627 | 45.7997883 |
| C | 62.6139991 | 48.3262309 | 45.1744399 | H | 63.5845784 | 41.8101872 | 45.0365845 |
| C | 62.1110784 | 49.5147764 | 44.3537624 | C | 61.1095709 | 44.1101429 | 45.3837769 |
| C | 63.0140611 | 50.7706450 | 44.4049685 | H | 60.0282587 | 43.9914870 | 45.5110479 |
| O | 62.5028004 | 51.8548419 | 44.0541896 | H | 61.5073595 | 44.6111979 | 46.2730375 |
| O | 64.2195637 | 50.6011957 | 44.7282017 | H | 61.3292710 | 44.6888662 | 44.4749783 |

| | | | |
|---|---|---|---|
| H | 61.3020653 | 44.4128089 | 48.8122310 |
| H | 64.5835138 | 45.6412477 | 45.9485903 |
| H | 64.2291978 | 43.5804844 | 46.8508286 |
| O | 64.0986053 | 45.0405052 | 45.3244698 |
| O | 61.4339392 | 43.4815459 | 49.1223126 |
| H | 60.8581259 | 42.9967446 | 48.4835367 |
| H | 59.7660423 | 45.7688597 | 47.8408732 |
| H | 55.9890131 | 48.9795895 | 51.7974156 |
| H | 60.5040798 | 44.0115557 | 52.2901605 |
| H | 61.6237574 | 51.5870912 | 53.4491266 |
| H | 56.7165084 | 40.3173146 | 43.1570684 |
